# Supplementary material for: Enantioselective de novo construction of 3‑oxindoles via organocatalyzed formal [3 + 2] annulation from simple arylamines
Source: Nat Commun. 2024 Jul 23;15:6183. doi: 10.1038/s41467-024-50400-2 (PMC11263680; doi:10.1038/s41467-024-50400-2)
Supplement: Supplementary file 1 — Supplementary Information [file 41467_2024_50400_MOESM1_ESM.pdf]

Supplementary Information for:

Enantioselective *de novo* construction of 3-oxindoles *via* organocatalyzed formal [3+2] annulation from arylamines

Yong Wang[1], Yanyan Li[1], Haohua Chen*[,2,3], Yu Lan[1,3], Chao Pi[1,3], Yangjie Wu[1,3], Xiuling Cui*[,1,3]

[1]Henan Key Laboratory of Chemical Biology and Organic Chemistry, College of Chemistry, Zhengzhou University, Zhengzhou, Henan 450052, P. R. China

[2]State Key Laboratory of Antiviral Drugs, Henan Normal University, Xinxiang, Henan 453007, P. R. China

[3]Pingyuan Laboratory, Henan, P. R. China

*Corresponding author. Email: cuixl@zzu.edu.cn; chenhaohua@htu.edu.cn

Table of Contents:

General Information 2

Optimization of the Reaction Conditions 2

Synthesis and Characterization of 2,3-Diketoesters **2** 4

General Procedure and Characterization of Products **4** 8

Hammett Plot Studies 29

Control Experiments 30

Gram-scale Synthesis and Further Chemical Transformations 30

Crystallographic Data for **4bq** (CCDC 2262643) 34

DFT Calculations 41

Copies of NMR Spectra 43

Copies of HPLC Spectra 127

References 168

General Information

Unless otherwise noted, substrates, catalysts and solvents were obtained from commercial suppliers and used without further purification. The amines **1b-1e**[1,2] were prepared according to the literatures. Products **3** and **4** were visualized by UV-light at 365 nm. Other products were visualized by UV-light at 254 nm. Flash chromatography was conducted on silica gel (200–300 mesh). The ^{1}H NMR, ^{13}C NMR and ^{19}F NMR spectra were obtained using a Bruker AVANCE III spectrometer at 400, 100 and 376 MHz, respectively. Chemical shifts are reported in units of parts per million (ppm) downfield from tetramethylsilane (TMS), and all coupling constants were reported in hertz. Peak multiplicity was indicated as follows: br = broad, s = singlet, d = doublet, t = triplet, q = quartet, quint = quintet, sext = sextet and m = multiplet. Enantiomeric excesses values were determined with HPLC on Chiral Daicel Chiralpak AS-H and AD-H (mobile phase: hexane/^{i}PrOH). Optical rotation were measured on a Anton Paar MCP 4100 polarimeter and reported as follows: $[\alpha]_{20}^{D}$ (c = g/100 mL, solvent). Melting points were uncorrected. X-ray analysis was performed with a single-crystal X-ray diffractometer (Gemini E) from Agilent. All high-resolution mass spectra were obtained on an TOF LC/MS equipped with an ESI source.

Optimization of the Reaction Conditions

Supplementary Table 1. Optimization of the reaction conditions*a*

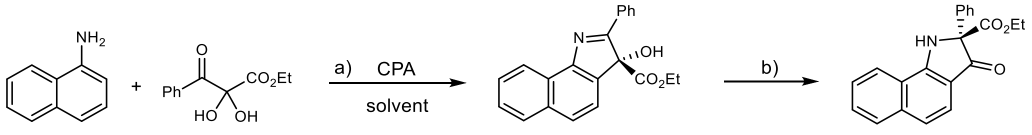

1a **2a** **3aa** **4aa**

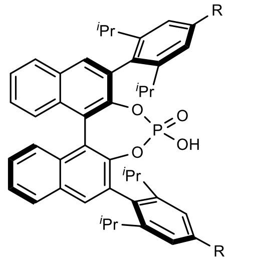

(*R*)-**5a**, R = ^{i}Pr
(*R*)-**5b**, R = 9-anthracenyl
(*R*)-**5c**, R = 2,4,6-$^{i}Pr_3C_6H_2$
(*R*)-**5f**, R = 1-Adamantyl

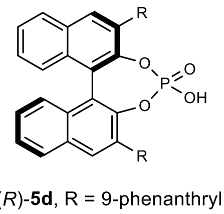

(*R*)-**5d**, R = 9-phenanthryl
(*R*)-**5e**, R = 9-anthryl
(*R*)-**5g**, R = 1-naphthyl
(*R*)-**5h**, R = 1-pyrenyl
(*R*)-**5i**, R = 2,4,6-(cyclohexyl)$_3C_6H_2$
(*R*)-**5j**, R = 3,5-$^{t}Bu_2C_6H_3$
(*R*)-**5k**, R = $SiPh_3$

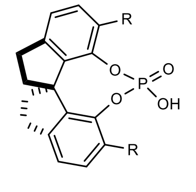

(*R*)-**6a**, R = 9-phenanthryl
(*R*)-**6b**, R = 9-anthryl
(*R*)-**6c**, R = 1-pyrenyl
(*R*)-**6d**, R = 1-naphthyl
(*R*)-**6e**, R = 2,4,6-(cyclohexyl)$_3C_6H_2$
(*R*)-**6f**, R = 3,5-$^{t}Bu_2C_6H_3$
(*R*)-**6g**, R = $SiPh_3$

entry	CPA	solvent	yield (**4aa**)%^{b}	ee (**4aa**)%^{c}
1	(*R*)-**5a**	toluene	95	-45
2	(*R*)-**5b**	toluene	84	-59
3	(*R*)-**5d**	toluene	90	28
4	(*R*)-**5e**	toluene	92	27
5	(*R*)-**5f**	toluene	96	-49
6	(*R*)-**5g**	toluene	89	20
7	(*R*)-**5h**	toluene	99	16

8	(*R*)-**5i**	toluene	99	-29
9	(*R*)-**5j**	toluene	99	-52
10	(*R*)-**5k**	toluene	72	21
11	(*R*)-**6a**	toluene	97	-52
12	(*R*)-**6b**	toluene	95	-68
13	(*R*)-**6c**	toluene	37	-2
14	(*R*)-**6e**	toluene	96	-26
15	(*R*)-**6f**	toluene	70	29
16	(*R*)-**6g**	toluene	64	1
17[d]	(*R*)-**5a**	cyclohexane	94	-46
18[d]	(*R*)-**5a**	$CHCl_3$	17	-39
19[d]	(*R*)-**5a**	PhCl	96	-66
20[d]	(*R*)-**5a**	benzene	99	-59
21[d]	(*R*)-**5a**	CH_2Cl_2	78	-68
22[d]	(*R*)-**5a**	PhF	74	-60
23[d]	(*R*)-**5a**	ODCB	87	-68
24[d]	(*R*)-**5a**	$PhCF_3$	96	-65
25[d]	(*R*)-**5a**	DCE	59	-61
26[d]	(*R*)-**5a**	*p*-xylene	66	-51
27[d]	(*R*)-**5a**	CCl_4	99	-67
28[d]	(*R*)-**5b**	CH_2Cl_2	71	-69
29[d]	(*R*)-**5b**	ODCB	67	-69
30[d]	(*R*)-**5c**	ODCB	66	-81
31[d]	(*R*)-**5c**	CH_2Cl_2	62	-81
32[d]	(*R*)-**5c**	CH_2Cl_2 : ODCB = 9 : 1	48	-81
33[d]	(*R*)-**5c**	CH_2Cl_2 : ODCB = 6 : 1	64	-84
34[d]	(*R*)-**5c**	CH_2Cl_2 : ODCB = 4 : 1	70	-88
35[d]	(*R*)-**5c**	CH_2Cl_2 : ODCB = 2 : 1	52	-83
36[d]	(*R*)-**5c**	CH_2Cl_2 : ODCB = 1 : 1	57	-79
37[d]	(*R*)-**5c**	CH_2Cl_2 : ODCB = 1 : 4	36	-79
38[defg]	(*R*)-**5c**	CH_2Cl_2 : ODCB = 4 : 1	59	-91
39[d]	(S)-**6b**	DCM	56	44
40[d]	(S)-**6b**	PhCl	84	43
41[d]	(S)-**6b**	ODCB	73	33
42[d]	(S)-**6b**	$CHCl_3$	76	-3

43[d]	(S)-**6b**	benzene	96	66
44[d]	(S)-**6b**	hexane	99	27
45[d]	(S)-**6b**	THF	18	62
46[d]	(S)-**6b**	cyclohexane	99	24
47[d]	(S)-**6b**	*p*-xylene	99	72
48[d]	(S)-**6b**	*o*-xylene	96	68
49[d]	(S)-**6b**	*m*-xylene	96	77
50[d]	(S)-**6b**	Et_2O	50	79
51[d]	(S)-**6b**	TBME	57	87
52[d]	(S)-**6b**	MeCN	26	72
53[d]	(S)-**6b**	EtOAc	54	77
54[def]	(S)-**6b**	AcO*t*Bu	99	87
55[def]	(S)-**6b**	AcO*i*Pr	99	88
56[def]	(S)-**6b**	methyl pivalate	98	87
57[defg]	(S)-**6b**	AcO*i*Pr	74	92

[a]Reaction conditions: a) **1a** (0.05 mmol), **2a** (0.055 mmol), CPA (10 mol %), solvent (2 mL), rt, 40 h. **3aa** was isolated for the second step. b) toluene (1mL), 100 °C, 20 h. Abbreviations: ODCB = 1,2-dichlorobenzene, TBME = *tert*-butyl methyl ether. [b]Isolated yields. [c]Determined by chiral HPLC analysis. [d]step b: TsOH·H_2O (1.0 equiv) was added in the second step at room temperature for 10 h. [e]**3aa** was not isolated and TsOH·H_2O was added directly in one-pot. [f]7 d for step a. [g]Solvent (4 mL).

Synthesis and Characterization of 2,3-Diketoesters 2

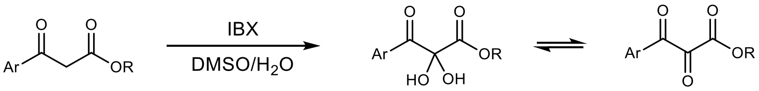

2

To the suspension of IBX (2.5 mmol) in the mixture of DMSO (22.5 mL) and water (7.5 mL), 1,3-dicarbonyl compounds (1 mmol) was added. The reaction mixture was stirred at 50 °C for 24 h, then filtered thourgh a pad of celite and washed with EA. The filtrate was washed with saturated $NaHCO_3$, water and brine, dried over Na_2SO_4, filtered, and concentrated under vacuum. The crude products were separated by flash column chromatography (PE : EA = 4 : 1) on silica gel to afford compounds **2**.

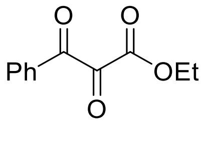

Ethyl 2,3-dioxo-3-phenylpropanoate (2a). [3]

Yellow oil. ^1H NMR ($CDCl_3$) δ: 8.02-7.98 (m, 2H), 7.74-7.68 (m, 1H), 7.59-7.51 (m, 2H), 4.43 (q, J = 7.2 Hz, 2H), 1.39(t, J = 7.2 Hz, 3H); ^{13}C NMR ($CDCl_3$) δ: 190.2, 183.8, 160.5, 135.5, 131.5,

130.0, 129.2, 63.3, 14.0.

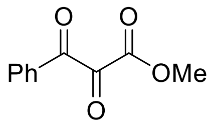

Methyl 2,3-dioxo-3-phenylpropanoate (2b). [4]

Yellow oil. ^{1}H NMR ($CDCl_3$) *δ*: 8.02-7.97 (m, 2H), 7.74-7.68 (m, 1H), 7.58-7.52 (m, 2H), 3.96(s, 3H); ^{13}C NMR ($CDCl_3$) *δ*: 190.0, 183.4, 160.8, 135.6, 131.8, 130.1, 129.2, 53.5.

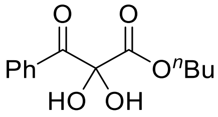

Butyl 2,2-dihydroxy-3-oxo-3-phenylpropanoate (2c).

Yellow oil. ^{1}H NMR ($CDCl_3$) *δ*: 8.10-8.06 (m, 2H), 7.63 (tt, *J* = 7.5, 1.2 Hz, 1H), 7.48 (t, *J* = 7.6 Hz, 2H), 5.31 (br, s, 2H), 4.16 (t, *J* = 6.5 Hz, 2H), 1.47-1.34 (m, 2H), 1.03 (sext, *J* = 7.4 Hz, 2H), 0.71 (t, *J* = 7.4 Hz, 3H); ^{13}C NMR ($CDCl_3$) *δ*: 191.5, 170.0, 134.7, 131.4, 130.1, 128.8, 91.5, 66.9, 30.1, 18.5, 13.3. HRMS (ESI) m/z calcd for $C_{13}H_{16}O_5Na^+$ $(M+Na)^+$ 275.0890, found 275.0891.

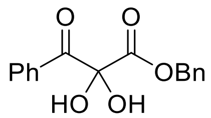

Ethyl 2,3-dioxo-3-phenylpropanoate (2d). [3]

Yellow oil. ^{1}H NMR ($CDCl_3$) *δ*: 7.99 (d, *J* = 7.4 Hz, 2H), 7.58 (t, *J* = 7.4 Hz, 1H), 7.38 (t, *J* = 7.5 Hz, 2H), 7.23 (d, *J* = 7.2 Hz, 1H), 7.21-7.15 (m, 2H), 7.01 (d, *J* = 7.2 Hz), 5.33 (br, s, 2H), 5.16 (s, 2H); ^{13}C NMR ($CDCl_3$) *δ*: 191.3, 169.6, 134.6, 134.0, 131.3, 130.1, 128.8, 128.5, 128.5, 128.1, 91.6, 68.5.

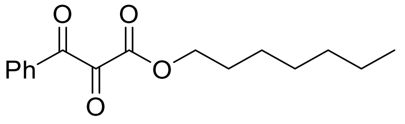

Heptyl 2,3-dioxo-3-phenylpropanoate (2e). [3]

Yellow oil. ^{1}H NMR ($CDCl_3$) *δ*: 8.02-7.97 (m, 2H), 7.74-7.67 (m, 1H), 7.55 (t, *J* = 7.6 Hz, 2H), 4.35 (t, *J* = 6.7 Hz, 2H), 1.73 (quint, *J* = 7.8 Hz, 2H), 1.38-1.22 (m, 8H), 0.88 (t, *J* = 7.1 Hz, 3H); ^{13}C NMR ($CDCl_3$) *δ*: 190.3, 183.9, 160.5, 135.5, 131.6, 130.0, 129.2, 67.3, 31.6, 28.7, 28.3, 25.6, 22.5, 14.0.

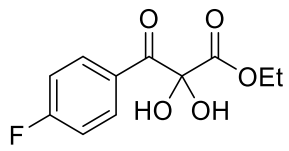

Ethyl 3-(4-fluorophenyl)-2,2-dihydroxy-3-oxopropanoate (2f). [3]

White solid. M.p. 96-98 °C.^{1}H NMR ($CDCl_3$) *δ*: 8.05-8.00 (m, 2H), 7.48-7.42 (m, 2H), 5.33 (br, s, 2H), 4.22 (q, *J* = 7.1 Hz, 2H), 1.11 (t, *J* = 7.1 Hz, 3H); ^{13}C NMR ($CDCl_3$) *δ*: 190.6, 169.7, 141.4,

131. 6, 129.7, 129.2, 91.7, 63.4, 13.7; ^{19}F NMR ($CDCl_3$) 101.5.

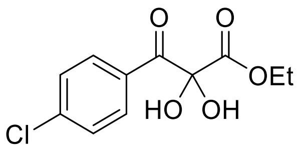

Ethyl 3-(4-chlorophenyl)-2,2-dihydroxy-3-oxopropanoate (2g). [5]
White solid. M.p.: 97-98 ^{o}C. ^{1}H NMR ($CDCl_3$) *δ*: 8.05-8.00 (m, 2H), 7.48-7.42 (m, 2H), 5.33 (br, s, 2H), 4.22 (q, *J* = 7.1 Hz, 2H), 1.11 (t, *J* = 7.1 Hz, 3H); ^{13}C NMR ($CDCl_3$) *δ*: 190.6, 169.7, 141.4, 131. 6, 129.7, 129.2, 91.7, 63.4, 13.7.

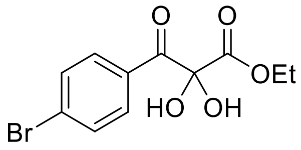

Ethyl 3-(4-bromophenyl)-2,2-dihydroxy-3-oxopropanoate (2h). [3]
White solid. M.p.: 104-105 ^{o}C. ^{1}H NMR ($CDCl_3$) *δ*: 7.97-7.92 (m, 2H), 7.65-7.60 (m, 2H), 5.26 (br, s, 2H), 4.22 (q, *J* = 7.1 Hz, 2H), 1.12 (t, *J* = 7.1 Hz, 3H); ^{13}C NMR ($CDCl_3$) *δ*: 190.8, 169.6, 132.2, 131.6, 130.3, 130.1, 91.6, 63.4, 13.7.

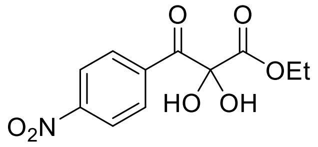

Ethyl 2,2-dihydroxy-3-(4-nitrophenyl)-3-oxopropanoate (2i).[3]
White solid. M.p.: 87-88 ^{o}C. ^{1}H NMR ($CDCl_3$) *δ*: 8.41-8.20 (m, 4H), 5.28 (br, s, 2H), 4.23 (q, *J* = 7.1 Hz, 2H), 1.12 (t, *J* = 7.1 Hz, 3H); ^{13}C NMR ($CDCl_3$) *δ*: 190.7, 169.1, 151.0, 136.1, 131.2, 123.9, 92.0, 63.6, 13.7.

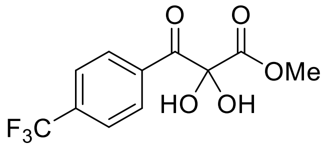

Methyl 2,2-dihydroxy-3-oxo-3-(4-(trifluoromethyl)phenyl)propanoate (2j).[3]
White solid. M.p.: 110-112 ^{o}C. ^{1}H NMR ($CDCl_3$) *δ*: 8.20 (d, *J* = 8.2 Hz, 2H), 7.75 (d, *J* = 8.3 Hz, 2H), 5.28 (br, s, 2H), 3.76 (s, 2H); ^{13}C NMR ($CDCl_3$) *δ*: 190.8, 169.9, 135.7 (q, *J* = 32.9 Hz), 134.1, 130.5, 125.9 (q, *J* = 3.7 Hz), 123.3 (q, *J* = 273.0 Hz), 91.9, 53.9; ^{19}F NMR ($CDCl_3$) 63.5.

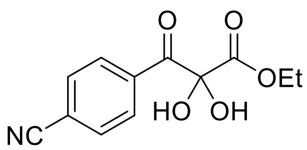

Ethyl 3-(4-cyanophenyl)-2,2-dihydroxy-3-oxopropanoate (2k).[4]
White solid. M.p.: 107-108 ^{o}C. ^{1}H NMR ($CDCl_3$) *δ*: 8.19 (d, *J* = 8.4 Hz, 2H), 7.78 (d, *J* = 8.4 Hz,

2H), 5.25 (br, s, 2H), 4.23 (q, *J* = 7.1 Hz, 2H), 1.11 (t, *J* = 7.2 Hz, 3H); ^{13}C NMR ($CDCl_3$) *δ*: 190.8, 169.1, 134.6, 132.5, 130.4, 117.7, 117.5, 91.9, 63.5, 13.7.

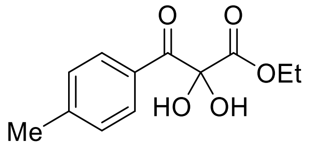

Ethyl 2,2-dihydroxy-3-oxo-3-(*p*-tolyl)propanoate (2l).[5]

White solid. M.p.: 91-92 °C. ^{1}H NMR ($CDCl_3$) *δ*: 7.97 (d, *J* = 8.3 Hz, 2H), 7.27 (d, *J* = 8.1 Hz, 2H), 5.33 (br, s, 2H), 4.21 (q, *J* = 7.2 Hz, 2H), 2.42 (s, 3H), 1.10 (t, *J* = 7.1 Hz, 3H); ^{13}C NMR ($CDCl_3$) *δ*: 191.0, 170.1, 146.0, 130.3, 129.5, 128.8, 91.5, 63.2, 21.9, 13.7.

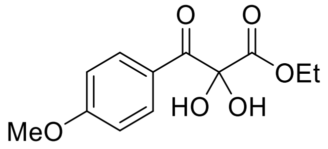

Ethyl 3-(4-methoxyphenyl)-2,3-dioxopropanoate (2m).[3]

Yellow oil. ^{1}H NMR ($CDCl_3$) *δ*: 8.09-8.04 (m, 2H), 6.96-6.91 (m, 2H), 5.38 (br, s, 2H), 4.22 (q, *J* = 7.2 Hz, 2H), 3.88 (s, 3H), 1.12 (t, *J* = 7.1 Hz, 3H); ^{13}C NMR ($CDCl_3$) *δ*: 189.8, 170.3, 164.8, 132.8, 124.1, 114.1, 91.5, 63.1, 55.6, 13.7.

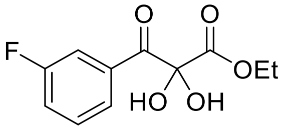

Ethyl 3-(3-fluorophenyl)-2,2-dihydroxy-3-oxopropanoate (2n).[4]

Yellow oil. ^{1}H NMR ($CDCl_3$) *δ*: 7.89-7.84 (m, 1H), 7.81-7.76 (m, 1H), 7.50-7.42 (m, 1H), 7.37-7.30 (m, 1H), 5.31 (br, s, 2H), 4.23 (q, *J* = 7.1 Hz, 2H), 1.10 (t, *J* = 7.1 Hz, 3H); ^{13}C NMR ($CDCl_3$) *δ*: 190.7 (d, *J* = 2.4 Hz), 169.5, 162.6 (d, *J* = 248.6 Hz), 133.4 (d, J = 6.8 Hz), 130.5 (d, *J* = 7.6 Hz), 126.0 (d, *J* = 3.1 Hz), 121.8 (d, *J* = 21.4 Hz), 116.8 (d, *J* = 23.3 Hz), 91.8, 63.4, 13.7; ^{19}F NMR ($CDCl_3$) -110.8.

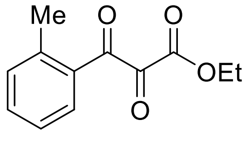

Ethyl 2,3-dioxo-3-(o-tolyl)propanoate (2o).[3]

Yellow oil. ^{1}H NMR ($CDCl_3$) *δ*: 7.69 (d, *J* = 7.8 Hz, 1H), 7.57-7.52 (m, 1H), 7.37-7.32 (m, 2H), 4.43 (q, *J* = 7.2 Hz, 2H), 2.63 (s, 3H), 1.39 (t, *J* = 7.1 Hz, 3H); ^{13}C NMR ($CDCl_3$) *δ*: 192.9, 183.6, 160.6, 141.7, 134.4, 133.1, 132.6, 132.6, 126.1, 63.3, 21.6, 14.0.

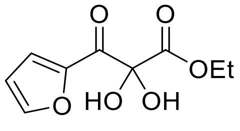

Ethyl 3-(furan-2-yl)-2,2-dihydroxy-3-oxopropanoate (2p).[6]

Yellow oil. ^{1}H NMR (CDCl$_3$) δ: 7.71-7.69 (m, 1H), 7.47-7.45 (m, 1H), 6.61 (dd, J = 3.7, 1.7 Hz, 1H), 5.29 (br, s, 2H), 4.27 (q, J = 7.2 Hz, 2H), 1.19 (t, J = 7.1 Hz, 3H); ^{13}C NMR (CDCl$_3$) δ: 180.1, 169.4, 148.6, 148.2, 122.7, 113.0, 91.2, 63.4, 13.8.

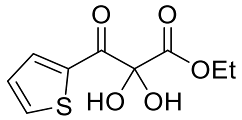

Ethyl 2,2-dihydroxy-3-oxo-3-(thiophen-2-yl)propanoate (2q).[5]

Yellow solid. M.p.: 170-171 °C. ^{1}H NMR (CDCl$_3$) δ: 7.94 (dd, J = 3.8, 0.6 Hz, 1H), 7.79 (dd, J = 4.9, 0.5 Hz, 1H), 7.16 (t, J = 4.2 Hz, 1H), 5.38 (br, s, 2H), 4.24 (q, J = 7.1 Hz, 2H), 1.15 (t, J = 7.1 Hz, 3H); ^{13}C NMR (CDCl$_3$) δ: 184.9, 169.7, 138.4, 136.6, 136.3, 128.7, 92.1, 63.4, 13.7.

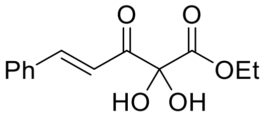

Ethyl (*E*)-2,2-dihydroxy-3-oxo-5-phenylpent-4-enoate (2r).[5]

Yellow oil. ^{1}H NMR (CDCl$_3$) δ: 7.92 (d, J = 16.0 Hz, 1H), 7.63-7.56 (m, 2H), 7.48-7.38 (m, 3H), 6.94 (d, J = 16.0 Hz, 1H), 5.18 (br, s, 2H), 4.30 (q, J = 7.2 Hz, 2H), 1.26 (t, J = 7.1 Hz, 3H); ^{13}C NMR (CDCl$_3$) δ: 191.1, 169.6, 148.1, 133.8, 131.7, 129.1, 129.0, 118.0, 92.3, 63.5, 13.9.

General Procedure and Characterization of Products 4

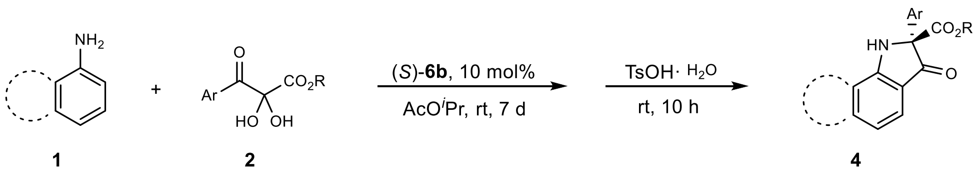

The amines **1** (0.05 mmol), 2,3-diketoesters **2** (0.055 mmol) and (*S*)-**6b** (10 mol %) were dissolved in AcO^{i}Pr. The reaction mixture was stirred at room temperature for 7 d. Then the TsOH·H$_2$O (1.0 equiv) was added. The reaction mixture was stirred at room temperature for 10 h. The solvent was removed in vacuo and the crude products was separated by flash column chromatography on silica gel to afford target products **4**.

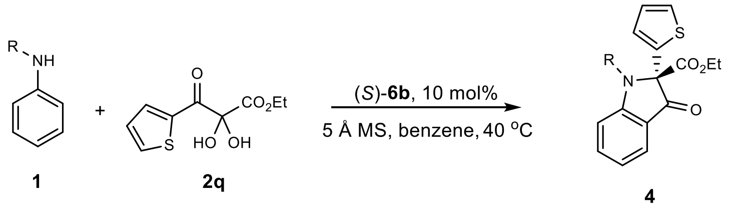

The amines **1** (0.05 mmol), 2,3-diketoesters **2q** (0.06 mmol), (*S*)-**6b** (10 mol %) and 5 Å MS (50 mg) were dissolved in benzene (1 mL). The reaction mixture was stirred at 40 °C for indicated time. The solvent was removed in vacuo and the crude products was separated by flash column

chromatography on silica gel to afford the target products **4**.

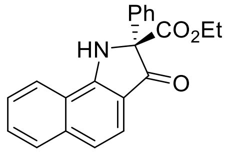

Ethyl (*R*)-3-oxo-2-phenyl-2,3-dihydro-1*H*-benzo[*g*]indole-2-carboxylate (4aa).
Product **4aa** was obtained by flash chromatography (DCM) in 74% yield as yellow solid. 92% ee. M.p.: 163-164 °C. ^{1}H NMR (CDCl$_3$) δ: 8.11 (d, J = 8.1 Hz, 1H), 7.88-7.82 (m, 3H), 7.71-7.65 (m, 1H), 7.62-7.56 (m, 1H), 7.54 (d, J = 8.6 Hz, 1H), 7.42-7.32 (m, 3H), 7.30 (d, J = 8.7 Hz, 1H), 6.37 (br, s, 1H), 4.37-4.23 (m, 2H), 1.31 (t, J = 7.1 Hz, 3H); ^{13}C NMR (CDCl$_3$) δ: 192.6, 167.7, 161.8, 138.3, 136.0, 130.5, 129.0, 128.6, 128.6, 126.4, 122.2, 121.5, 121.2, 120.4, 114.3, 75.7, 63.3, 14.0; HRMS (ESI) m/z calcd for $C_{21}H_{18}NO_3^+$ (M+H)$^+$ 332.1281, found 332.1281. $[\alpha]^{20}_D$ = -86.8 (c 0.1, DCM). Chiral HPLC: Chiralpak AS-H, hexane:^{i}PrOH = 80:20, 1.0 mL/min, 400 nm; tR = 9.3 min (major), 16.4 min (minor).

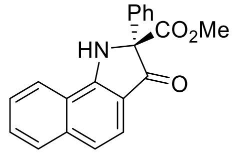

Methyl (*R*)-3-oxo-2-phenyl-2,3-dihydro-1*H*-benzo[*g*]indole-2-carboxylate (4ab).
Product **4ab** was obtained by flash chromatography (DCM) in 99% yield as yellow solid. 89% ee. M.p.: 219-221 °C. ^{1}H NMR (CDCl$_3$) δ: 8.11 (d, J = 8.1 Hz, 1H), 7.88-7.79 (m, 3H), 7.69 (d, J = 7.8 Hz, 1H), 7.59 (d, J = 7.2 Hz, 1H), 7.54 (d, J = 8.5 Hz, 1H), 7.43-7.34 (m, 3H), 7.31 (d, J = 8.6 Hz, 1H), 6.38 (br, s, 1H), 3.84 (s, 3H); ^{13}C NMR (CDCl$_3$) δ: 193.6, 168.2, 161.8, 138.4, 135.9, 130.6, 129.1, 128.7, 128.7, 126.4, 126.4, 122.2, 121.4, 121.3, 120.4, 114.3, 75.7, 53.9; HRMS (ESI) m/z calcd for $C_{20}H_{16}NO_3^+$ (M+H)$^+$ 318.1125, found 318.1126. $[\alpha]^{20}_D$ = -55.2 (c 0.2, DCM). Chiral HPLC: Chiralpak AS-H, hexane:^{i}PrOH = 80:20, 1.0 mL/min, 400 nm; tR = 9.9 min (major), 20.4 min (minor).

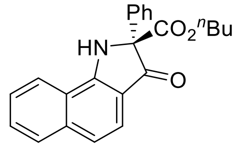

Butyl (*R*)-3-oxo-2-phenyl-2,3-dihydro-1*H*-benzo[*g*]indole-2-carboxylate (4ac).
Product **4ac** was obtained by flash chromatography (DCM) in 87% yield as yellow solid. 91% ee. M.p.: 172-173 °C. ^{1}H NMR (CDCl$_3$) δ: 8.12 (d, J = 8.1 Hz, 1H), 7.90-7.82 (m, 3H), 7.71-7.66 (m, 1H), 7.62-7.57 (m, 1H), 7.54 (d, J = 8.6 Hz, 1H), 7.42-7.28 (m, 4H), 6.36 (br, s, 1H), 4.28-4.17 (m, 2H), 1.65 (m, 2H), 1.35 (sext, J = 7.6 Hz, 2H), 0.89 (t, J = 7.4 Hz, 3H); ^{13}C NMR (CDCl$_3$) δ:192.6, 167.7, 161.8, 138.3, 136.0, 130.5, 129.0, 128.6, 128.5, 126.4, 126.4, 122.2, 121.5, 121.2, 120.4, 114.4, 75.7, 67.0, 30.4, 19.0, 13.6; HRMS (ESI) m/z calcd for $C_{23}H_{12}NO_3^+$ (M+H)$^+$ 360.1594, found 360.1592. $[\alpha]^{20}_D$ = -56.6 (c 0.1, DCM). Chiral HPLC: Chiralpak AS-H, hexane:^{i}PrOH = 80:20, 1.0 mL/min, 400 nm; tR = 8.1 min (major), 13.5 min (minor).

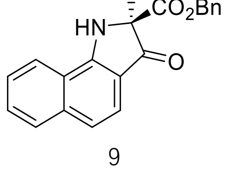

Benzyl (*R*)-3-oxo-2-phenyl-2,3-dihydro-1*H*-benzo[*g*]indole-2-carboxylate (4ad).

Product **4ad** was obtained by flash chromatography (DCM) in 94% yield as yellow solid. 86% ee. M.p.: 76-78 °C. ^{1}H NMR ($CDCl_3$) δ: 8.07 (d, J = 8.1 Hz, 1H), 7.86-7.78 (m, 3H), 7.70-7.64 (m, 1H), 7.59-7.50 (m, 2H), 7.40-7.26 (m, 9H), 6.37 (br, s, 1H), 5.26 (s, 2H); ^{13}C NMR ($CDCl_3$) δ: 192.5, 167.6, 161.7, 138.4, 135.8, 134.9, 130.5, 129.0, 128.6, 128.6, 128.4, 127.9, 126.5, 126.4, 122.2, 121.4, 121.2, 120.4, 114.3, 75.7, 68.5; HRMS (ESI) m/z calcd for $C_{26}H_{20}NO_3^+$ $(M+H)^+$ 394.1438, found 394.1437. $[\alpha]^{20}_D$ = -70.9 (*c* 0.04, DCM). Chiral HPLC: Chiralpak AS-H, hexane:^{i}PrOH = 80:20, 1.0 mL/min, 400 nm; tR = 9.9 min (major), 16.0 min (minor).

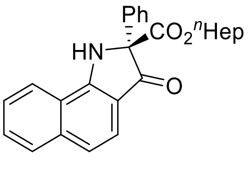

Heptyl (*R*)-3-oxo-2-phenyl-2,3-dihydro-1*H*-benzo[*g*]indole-2-carboxylate (4ae).

Product **4ae** was obtained by flash chromatography (DCM) in 72% yield as yellow solid. 91% ee. M.p.: 106-108 °C. ^{1}H NMR ($CDCl_3$) δ: 8.11 (d, J = 8.1 Hz, 1H), 7.93-7.81 (m, 3H), 7.69 (t, J = 7.8 Hz, 1H), 7.59 (t, J = 7.2 Hz, 1H), 7.54 (d, J = 8.6 Hz, 1H), 7.43-7.28 (m, 4H), 6.35 (br, s, 1H), 4.28-4.16 (m, 2H), 1.66 (quintet, J = 7.0 Hz, 2H), 1.36-1.12 (m, 8H), 0.85 (t, J = 7.1 Hz, 3H); ^{13}C NMR ($CDCl_3$) δ: 192.6, 167.7, 161.8, 138.3, 136.0, 130.5, 129.0, 128.6, 128.5, 126.4, 126.4, 122.2, 121.5, 121.2, 120.4, 114.4, 75.7, 67.3, 31.6, 28.7, 28.4, 25.6, 22.5, 14.1; HRMS (ESI) m/z calcd for $C_{26}H_{28}NO_3^+$ $(M+H)^+$ 402.2064, found 402.2065. $[\alpha]^{20}_D$ = -53.5 (*c* 0.1, DCM). Chiral HPLC: Chiralpak AS-H, hexane:^{i}PrOH = 80:20, 1.0 mL/min, 400 nm; tR = 5.4 min (major), 11.8 min (minor).

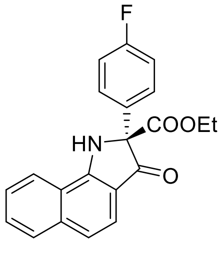

Ethyl (*R*)-2-(4-fluorophenyl)-3-oxo-2,3-dihydro-1*H*-benzo[*g*]indole-2-carboxylate (4af).

Product **4af** was obtained by flash chromatography (DCM) in 98% yield as yellow oil. 94% ee. ^{1}H NMR ($CDCl_3$) δ: 8.12 (d, J = 8.2 Hz, 1H), 7.96-7.82 (m, 3H), 7.69 (t, J = 7.4 Hz, 1H), 7.60 (t, J = 7.4 Hz, 1H), 7.53 (d, J = 8.6 Hz, 1H), 7.32 (d, J = 8.6 Hz, 1H), 7.10-7.03 (m, 2H), 6.37 (br, s, 1H), 4.36-4.22 (m, 2H), 1.30 (t, J = 7.1 Hz, 3H); ^{13}C NMR ($CDCl_3$) δ: 192.5, 167.5, 162.9 (d, J = 247.9 Hz), 161.8, 138.4, 131.6 (d, J = 3.1 Hz), 130.6, 129.1, 128.3 (d, J = 8.3 Hz), 126.5, 122.2, 121.6, 121.5, 120.3, 115.4 (d, J = 21.4 Hz), 114.3, 75.0, 63.4, 14.0; ^{19}F NMR ($CDCl_3$) δ: -113.8; HRMS (ESI) m/z calcd for $C_{21}H_{17}FNO_3^+$ $(M+H)^+$ 350.1187, found 350.1186. $[\alpha]^{20}_D$ = -103.5 (*c* 0.2, DCM). Chiral HPLC: Chiralpak AS-H, hexane:^{i}PrOH = 80:20, 1.0 mL/min, 400 nm; tR = 9.0 min (major), 13.3 min (minor).

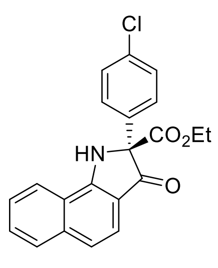

Ethyl (*R*)-2-(4-chlorophenyl)-3-oxo-2,3-dihydro-1*H*-benzo[*g*]indole-2-carboxylate (4ag).
Product **4ag** was obtained by flash chromatography (DCM) in 99% yield as yellow oil. 94% ee. ^{1}H NMR ($CDCl_3$) δ: 8.12 (d, J = 8.1 Hz, 1H), 7.90-7.83 (m, 3H), 7.73-7.67 (m, 1H), 7.63-7.58 (m, 1H), 7.52 (d, J = 8.6 Hz, 1H), 7.38-7.30 (m, 3H), 6.34 (br, s, 1H), 4.35-4.22 (m, 2H), 1.30 (t, J = 7.1 Hz, 3H); ^{13}C NMR ($CDCl_3$) δ: 192.2, 167.3, 161.9, 138.4, 134.7, 134.4, 130.7, 129.1, 128.7, 127.9, 126.5, 122.2, 121.6, 121.6, 120.3, 114.2, 75.1, 63.5, 14.0; HRMS (ESI) m/z calcd for $C_{21}H_{17}ClNO_3^+$ $(M+H)^+$ 366.0891, found 366.0891. $[\alpha]^{20}_D$ = -155.0 (*c* 0.2, DCM). Chiral HPLC: Chiralpak AS-H, hexane:^{i}PrOH = 80:20, 1.0 mL/min, 400 nm; tR = 9.3 min (major), 13.6 min (minor).

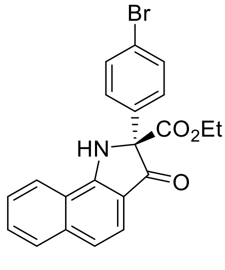

Ethyl (*R*)-2-(4-bromophenyl)-3-oxo-2,3-dihydro-1*H*-benzo[*g*]indole-2-carboxylate (4ah).
Product **4ah** was obtained by flash chromatography (DCM) in 98% yield as yellow oil. 94% ee. ^{1}H NMR ($CDCl_3$) δ: 8.12 (d, J = 8.1 Hz, 1H), 7.85 (d, J = 8.1 Hz, 1H), 7.83-7.78 (m, 2H), 7.72-7.66 (m, 1H), 7.62-7.56 (m, 1H), 7.54-7.47 (m, 3H), 7.32 (d, J = 8.6 Hz, 1H), 6.38 (br, s, 1H), 4.35-4.22 (m, 2H), 1.30 (t, J = 7.1 Hz, 3H); ^{13}C NMR ($CDCl_3$) δ: 192.1, 167.2, 161.9, 138.4, 134.9, 131.6, 130.7, 129.1, 128.3, 126.5, 122.9, 122.2, 121.5, 121.5, 120.3, 114.3, 75.1, 63.5, 14.0; HRMS (ESI) m/z calcd for $C_{21}H_{17}BrNO_3^+$ $(M+H)^+$ 410.0386, found 410.0387. $[\alpha]^{20}_D$ = -94.0 (*c* 0.2, DCM). Chiral HPLC: Chiralpak AS-H, hexane:^{i}PrOH = 80:20, 1.0 mL/min, 400 nm; tR = 9.8 min (major), 14.2 min (minor).

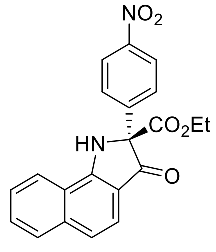

Ethyl (*R*)-2-(4-nitrophenyl)-3-oxo-2,3-dihydro-1*H*-benzo[*g*]indole-2-carboxylate (4ai).
Product **4ai** was obtained by flash chromatography (PE:EA = 3:1) in 99% yield as yellow solid. 96% ee. M.p.: 56-57 °C.^{1}H NMR ($CDCl_3$) δ: 8.26-8.13 (m, 5H), 7.88 (d, J = 8.1 Hz, 1H), 7.76-7.70 (m, 1H), 7.67-7.61 (m, 1H), 7.52 (d, J = 8.6 Hz, 1H), 7.37 (d, J = 8.6 Hz, 1H), 6.38 (br, s, 1H), 4.36-4.26 (m, 2H), 1.31 (t, J = 7.1 Hz, 3H); ^{13}C NMR ($CDCl_3$) δ: 191.1, 166.5, 162.1, 148.0, 142.5, 138.4, 130.9, 129.2, 127.7, 126.8, 123.5, 122.2, 122.1, 121.6, 120.2, 114.2, 75.3, 63.9, 14.0; HRMS (ESI) m/z calcd for $C_{21}H_{17}N_2O_5^+$ $(M+H)^+$ 377.1132, found 377.1132. $[\alpha]^{20}_D$ = -113.9 (*c* 0.1, DCM). Chiral HPLC: Chiralpak AS-H, hexane:^{i}PrOH = 80:20, 1.0 mL/min, 400 nm; tR = 18.4 min (major), 30.5 min (minor).

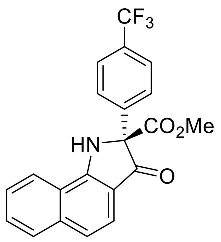

Methyl (*R*)-3-oxo-2-(4-(trifluoromethyl)phenyl)-2,3-dihydro-1*H*-benzo[*g*]indole-2-carboxylate (4aj).

Product **4aj** was obtained by flash chromatography (DCM) in 95% yield as yellow oil. 95% ee. ^{1}H NMR ($CDCl_3$) δ: 8.14 (d, J = 8.1 Hz, 1H), 8.06 (d, J = 8.3 Hz, 2H), 7.87 (d, J = 8.1 Hz, 1H), 7.74-7.68 (m, 1H), 7.67-7.59 (m, 3H), 7.53 (d, J = 8.6 Hz, 1H), 7.34 (d, J = 8.6 Hz, 1H), 6.39 (br, s, 1H), 3.85 (s, 3H); ^{13}C NMR ($CDCl_3$) δ: 191.7, 167.5, 162.0, 139.4, 138.4, 130.8, 130.8 (q, J = 32.4 Hz), 129.1, 127.0, 126.6, 125.4 (q, J = 3.7 Hz), 123.9 (q, J = 272.5 Hz), 122.2, 121.8, 121.5, 120.3, 114.1, 75.3, 54.2; ^{19}F NMR ($CDCl_3$) δ: -62.7; HRMS (ESI) m/z calcd for $C_{22}H_{15}F_3NO_3^+$ $(M+H)^+$ 386.0999, found 386.0999. $[\alpha]^{20}_D$ = -121.9 (c 0.2, DCM). Chiral HPLC: Chiralpak AS-H, hexane:^{i}PrOH = 80:20, 1.0 mL/min, 400 nm; tR = 6.8 min (major), 9.9 min (minor).

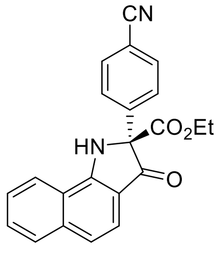

Ethyl (*R*)-2-(4-cyanophenyl)-3-oxo-2,3-dihydro-1*H*-benzo[*g*]indole-2-carboxylate (4ak).

Product **4ak** was obtained by flash chromatography (PE:EA = 3:1) in 99% yield as yellow oil. 95% ee. ^{1}H NMR ($CDCl_3$) δ: 8.17-8.09 (m, 3H), 7.87 (d, J = 8.1 Hz, 1H), 7.71 (t, J = 7.2 Hz, 1H), 7.67 (d, J = 8.4 Hz, 2H), 7.62 (t, J = 7.5 Hz, 1H), 7.51 (d, J = 8.6 Hz, 1H), 7.35 (d, J = 8.6 Hz, 1H), 6.40 (br, s, 1H), 4.30 (q, J = 7.1 Hz, 2H), 1.30 (t, J = 7.1 Hz, 3H); ^{13}C NMR ($CDCl_3$) δ: 191.3, 166.6, 162.1, 140.7, 138.4, 132.2, 130.9, 129.2, 129.1, 127.5, 126.7, 122.2, 122.0, 121.6, 120.2, 118.6, 114.1, 112.4, 75.2, 63.9, 14.0; HRMS (ESI) m/z calcd for $C_{22}H_{17}N_2O_3^+$ $(M+H)^+$ 357.1234, found 357.1233. $[\alpha]^{20}_D$ = -181.9 (c 0.2, DCM). Chiral HPLC: Chiralpak AS-H, hexane:^{i}PrOH = 80:20, 1.0 mL/min, 400 nm; tR = 12.6 min (major), 21.0 min (minor).

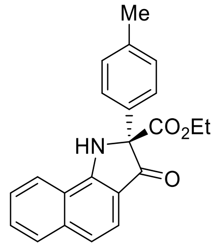

Ethyl (*R*)-3-oxo-2-(*p*-tolyl)-2,3-dihydro-1*H*-benzo[*g*]indole-2-carboxylate (4al).

Product **4al** was obtained by flash chromatography (DCM) in 88% yield as yellow solid. 94% ee. M.p.: 93-95 °C. ^{1}H NMR ($CDCl_3$) δ: 8.10 (d, J = 8.1 Hz, 1H), 7.84 (d, J = 8.1 Hz, 1H), 7.75-7.64 (m, 3H), 7.61-7.55 (m, 1H), 7.53 (d, J = 8.6 Hz, 1H), 7.30 (d, J = 8.6 Hz, 1H), 7.19 (d, J = 8.2 Hz, 2H), 6.35 (br, s, 1H), 4.36-4.23 (m, 2H), 2.33 (s, 3H), 1.30 (t, J = 7.1 Hz, 3H); ^{13}C NMR ($CDCl_3$) δ: 192.9, 167.8, 161.7, 138.4, 138.3, 133.1, 130.5, 129.3, 129.0, 128.7, 127.2, 126.3, 126.3, 122.2, 121.5, 121.1, 120.4, 114.3,75.6, 63.2, 21.1, 14.0; HRMS (ESI) m/z calcd for $C_{22}H_{20}NO_3^+$ $(M+H)^+$

346.1438, found 346.1438. $[\alpha]^{20}_{D}$ = -45.2 (*c* 0.2, DCM). Chiral HPLC: Chiralpak AS-H, hexane:^{i}PrOH = 80:20, 1.0 mL/min, 400 nm; tR = 9.5 min (major), 16.8 min (minor).

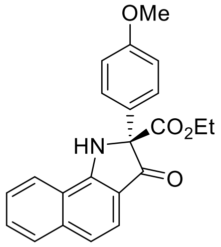

Ethyl (*R*)-2-(4-methoxyphenyl)-3-oxo-2,3-dihydro-1*H*-benzo[*g*]indole-2-carboxylate (4am).
Product **4am** was obtained by flash chromatography (DCM) in 97% yield as yellow oil. 78% ee. ^{1}H NMR ($CDCl_3$) δ: 8.10 (d, *J* = 8.2 Hz, 1H), 7.85 (d, *J* = 8.1 Hz, 1H), 7.80-7.74 (m, 2H), 7.71-7.65 (m, 1H), 7.61-7.55 (m, 1H), 7.53 (d, *J* = 8.6 Hz, 1H), 7.30 (d, *J* = 8.6 Hz, 1H), 6.93-6.87 (m, 2H), 6.36 (br, s, 1H), 4.36-4.22 (m, 2H), 3.79 (s, 3H), 1.30 (t, *J* = 7.1 Hz, 3H); ^{13}C NMR ($CDCl_3$) δ: 193.1, 167.9, 161.7, 159.8, 138.3, 130.5, 129.0, 128.1, 127.7, 126.3, 122.2, 121.5, 121.1, 120.4, 114.4, 114.0, 75.3, 63.2, 55.3, 14.0; HRMS (ESI) m/z calcd for $C_{22}H_{20}NO_4^+$ $(M+H)^+$ 362.1387, found 362.1386. $[\alpha]^{20}_{D}$ = +62.8 (*c* 0.2, DCM). Chiral HPLC: Chiralpak AS-H, hexane:^{i}PrOH = 80:20, 1.0 mL/min, 400 nm; tR = 12.0 min (major), 22.2 min (minor).

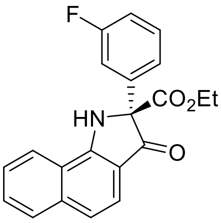

Ethyl (*R*)-2-(3-fluorophenyl)-3-oxo-2,3-dihydro-1*H*-benzo[*g*]indole-2-carboxylate (4an).
Product **4an** was obtained by flash chromatography (DCM) in 90% yield as yellow solid. 95% ee. M.p.: 177-179 °C. ^{1}H NMR ($CDCl_3$) δ: 8.12 (d, *J* = 8.1 Hz, 1H), 7.85 (d, *J* = 8.1 Hz, 1H), 7.74-7.63 (m, 3H), 7.63-7.57 (m, 1H), 7.53 (d, *J* = 8.6 Hz, 1H), 7.39-7.30 (m, 2H), 7.04 (tdd, *J* = 8.3, 2.6, 0.7 Hz, 1H), 6.39 (br, s, 1H), 4.37-4.23 (m, 2H), 1.31 (t, *J* = 7.1 Hz, 3H); ^{13}C NMR ($CDCl_3$) δ:192.0, 167.2, 162.8 (d, *J* = 245.5 Hz), 161.9, 138.4, 138.2 (d, *J* = 7.8 Hz), 130.7, 130.0 (d, *J* = 8.1 Hz), 129.1, 126.5, 122.2, 122.1 (d, *J* = 3.0 Hz), 121.5, 121.5, 120.3, 115.5 (d, *J* = 21.0 Hz), 114.2, 113.9 (d, *J* = 24.2 Hz), 75.1 (d, *J* = 1.8 Hz), 63.5, 14.0; ^{19}F NMR ($CDCl_3$) δ: -112.2. HRMS (ESI) m/z calcd for $C_{21}H_{17}FNO_3^+$ $(M+H)^+$ 350.1187, found 350.1186. $[\alpha]^{20}_{D}$ = -93.9 (*c* 0.1, DCM). Chiral HPLC: Chiralpak AS-H, hexane:^{i}PrOH = 80:20, 1.0 mL/min, 400 nm; tR = 6.8 min (major), 11.9 min (minor).

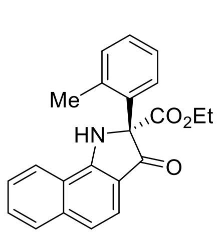

Ethyl (*S*)-3-oxo-2-(*o*-tolyl)-2,3-dihydro-1*H*-benzo[*g*]indole-2-carboxylate (4ao).
Product **4ao** was obtained by flash chromatography (PE:EA = 4:1) in 99% yield as yellow solid. 86% ee. M.p.: 107-110 °C. ^{1}H NMR ($CDCl_3$) δ: 7.99 (d, *J* = 8.1 Hz, 1H), 7.85 (d, *J* = 8.2 Hz, 1H), 7.67 (t, *J* = 7.7 Hz, 3H), 7.62 (d, *J* = 8.6 Hz, 1H), 7.54 (t, *J* = 7.9 Hz, 1H), 7.38-7.16 (m, 5H), 6.17 (br, s, 1H), 4.40-4.25 (m, 2H), 2.30 (s, 3H), 1.30 (t, *J* = 7.1 Hz, 3H); ^{13}C NMR ($CDCl_3$) δ: 193.9, 168.9,

161.6, 138.4, 137.5, 135.1, 132.1, 130.6, 129.0, 128.7, 128.0, 126.4, 126.3, 122.2, 121.4 121.3, 120.2, 115.1, 78.0, 63.3, 20.4, 14.0; HRMS (ESI) m/z calcd for $C_{22}H_{20}NO_3^+$ $(M+H)^+$ 346.1438, found 346.1438. $[\alpha]^{20}_D$ = -265.1 (*c* 0.2, DCM). Chiral HPLC: Chiralpak AS-H, hexane:^{i}PrOH = 80:20, 1.0 mL/min, 400 nm; tR = 8.6 min (minor), 11.4 min (major).

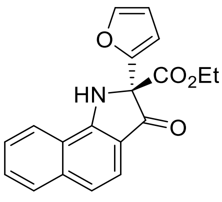

Ethyl (*R*)-2-(furan-2-yl)-3-oxo-2,3-dihydro-1*H*-benzo[*g*]indole-2-carboxylate (4ap).

Product **4ap** was obtained by flash chromatography (DCM) in 70% yield as yellow solid. 96% ee. M.p.: 198-200 °C. ^{1}H NMR ($CDCl_3$) δ: 8.03 (d, *J* = 8.3 Hz, 1H), 7.85 (d, *J* = 8.1 Hz, 1H), 7.71-7.65 (m, 1H), 7.61-7.54 (m, 2H), 7.43 (dd, *J* = 1.8, 0.7 Hz, 1H), 7.33 (d, *J* = 8.6 Hz, 1H), 6.57 (dd, *J* = 3.4, 0.6 Hz, 1H), 6.40 (dd, *J* = 3.4, 1.9 Hz, 1H), 6.27 (br, s, 1H), 4.39-4.25 (m, 2H), 1.29 (t, *J* = 7.1 Hz, 3H); ^{13}C NMR ($CDCl_3$) δ: 190.8, 166.2, 162.3, 148.7, 143.2, 138.5, 130.7, 129.0, 126.4, 122.3, 121.4, 121.4, 120.3, 114.1, 110.8, 108.9, 72.3, 63.5, 14.1; HRMS (ESI) m/z calcd for $C_{19}H_{16}NO_4^+$ $(M+H)^+$ 322.1074, found 322.1075. $[\alpha]^{20}_D$ = +156.9 (*c* 0.1, DCM). Chiral HPLC: Chiralpak AS-H, hexane:^{i}PrOH = 80:20, 1.0 mL/min, 400 nm; tR = 9.5 min (major), 10.9 min (minor).

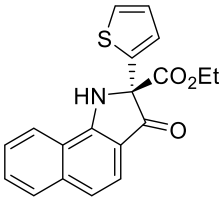

Ethyl (*R*)-3-oxo-2-(thiophen-2-yl)-2,3-dihydro-1*H*-benzo[*g*]indole-2-carboxylate (4aq).

Product **4aq** was obtained by flash chromatography (DCM) in 99% yield as yellow solid. 97% ee. M.p.: 184-185 °C. ^{1}H NMR ($CDCl_3$) δ: 8.11 (d, *J* = 8.2 Hz, 1H), 7.85 (d, *J* = 8.2 Hz, 1H), 7.72-7.66 (m, 1H), 7.63-7.57 (m, 1H), 7.56 (dd, *J* = 3.7, 1.2 Hz, 1H), 7.53 (d, *J* = 8.6 Hz, 1H), 7.34 (d, *J* = 8.6 Hz, 1H), 7.24 (dd, *J* = 5.1, 1.2 Hz, 1H), 7.04 (dd, *J* = 5.1, 3.7 Hz, 1H), 6.46 (br, s, 1H), 4.40-4.23 (m, 2H), 1.34 (t, *J* = 7.1 Hz, 3H); ^{13}C NMR ($CDCl_3$) δ: 191.4, 166.8, 162.1, 138.7, 138.4, 130.7, 129.0, 127.5, 126.5, 125.6, 125.5, 122.3, 121.9, 121.6, 120.4, 113.8, 74.0, 63.6, 14.0; HRMS (ESI) m/z calcd for $C_{19}H_{16}NO_3S^+$ $(M+H)^+$ 338.0845, found 338.0846. $[\alpha]^{20}_D$ = -110.6 (*c* 0.1, DCM). Chiral HPLC: Chiralpak AS-H, hexane:^{i}PrOH = 80:20, 1.0 mL/min, 400 nm; tR = 9.4 min (major), 12.2 min (minor).

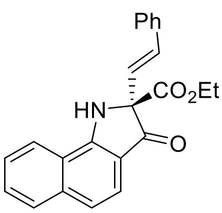

Ethyl (*R*,*E*)-3-oxo-2-styryl-2,3-dihydro-1*H*-benzo[*g*]indole-2-carboxylate (4ar).

Product **4ar** was obtained by flash chromatography (DCM) in 85% yield as yellow solid. 90% ee. M.p.: 192-193 °C. ^{1}H NMR ($CDCl_3$) δ: 8.11 (d, *J* = 8.2 Hz, 1H), 7.84 (d, *J* = 8.2 Hz, 1H), 7.71-7.65 (m, 1H), 7.61-7.55 (m, 1H), 7.53 (d, *J* = 8.6 Hz, 1H), 7.44-7.38 (m, 2H), 7.33-7.27 (m, 3H), 7.27-7.21 (m, 1H), 6.97 (d, *J* = 15.9 Hz, 1H), 6.78 (d, *J* = 15.9 Hz, 1H), 6.12 (br, s, 1H), 4.33 (q, *J* = 7.1 Hz, 2H), 1.35 (t, *J* = 7.1 Hz, 3H); ^{13}C NMR ($CDCl_3$) δ: 192.8, 167.5, 162.5, 138.3, 136.0, 130.7,

130.5, 129.0, 128.6, 128.4, 128.1, 127.9, 127.7, 127.5, 126.9, 126.4, 125.1, 122.2, 121.7, 121.4, 120.4, 113.8, 76.0, 63.3, 14.2; HRMS (ESI) m/z calcd for $C_{23}H_{19}NO_3Na^+$ $(M+Na)^+$ 380.1257, found 380.1261. $[\alpha]^{20}_D$ = -92.6 (*c* 0.1, DCM). Chiral HPLC: Chiralpak IE-3, hexane:^{i}PrOH = 80:20, 1.0 mL/min, 400 nm; tR = 15.9 min (minor), 27.2 min (major).

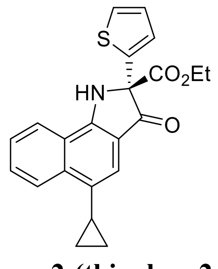

Ethyl (*R*)-5-cyclopropyl-3-oxo-2-(thiophen-2-yl)-2,3-dihydro-1H-benzo[*g*]indole-2-carboxylate (4bq).

Product **4bq** was obtained by flash chromatography (DCM) in 99% yield as yellow solid. 99% ee. M.p.: 164-166 °C. ^{1}H NMR ($CDCl_3$) *δ*: 8.45 (d, *J* = 8.4 Hz, 1H), 8.14 (d, *J* = 8.0 Hz, 1H), 7.81-7.74 (m, 1H), 7.68-7.60 (m, 1H), 7.55 (dd, *J* = 3.7, 1.2 Hz, 1H), 7.35 (s, 1H), 7.23 (dd, *J* = 5.1, 1.2 Hz, 1H), 7.03 (dd, *J* = 5.1, 3.7 Hz, 1H), 6.36 (br, s, 1H), 4.39-4.23 (m, 2H), 2.22-2.11 (m, 1H), 1.33 (t, *J* = 7.1 Hz, 3H), 1.06-0.97 (m, 2H), 0.73-0.65 (m, 2H); ^{13}C NMR ($CDCl_3$) *δ*: 191.6, 166.9, 161.4, 138.9, 138.6, 133.3, 130.6, 127.5, 126.3, 126.1, 125.6, 125.4, 122.7, 121.9, 118.2, 113.4, 74.0, 63.5, 14.0, 13.2, 6.2, 6.1; HRMS (ESI) m/z calcd for $C_{22}H_{20}NO_3S^+$ $(M+H)^+$ 378.1158, found 378.1159. $[\alpha]^{20}_D$ = -81.3 (*c* 0.2, DCM). Chiral HPLC: Chiralpak AS-H, hexane:^{i}PrOH = 80:20, 1.0 mL/min, 400 nm; tR = 10.5 min (major), 13.4 min (minor).

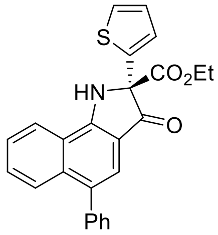

Ethyl (*R*)-3-oxo-5-phenyl-2-(thiophen-2-yl)-2,3-dihydro-1*H*-benzo[*g*]indole-2-carboxylate (4cq).

Product **4cq** was obtained by flash chromatography (DCM) in 99% yield as yellow solid. 96% ee. M.p.: 169-172 °C. ^{1}H NMR ($CDCl_3$) *δ*: 8.22-8.15 (m, 1H), 7.93-7.85 (m, 1H), 7.68-7.60 (m, 2H), 7.58 (dd, *J* = 3.7, 1.2 Hz, 1H), 7.51-7.44 (m, 3H), 7.44-7.37 (m, 3H), 7.27-7.23 (m, 1H), 7.05 (dd, *J* = 5.1, 3.7 Hz, 1H), 6.49 (br, s, 1H), 4.41-4.26 (m, 2H), 1.35 (t, *J* = 7.1 Hz, 3H); ^{13}C NMR ($CDCl_3$) *δ*: 191.5, 166.8, 161.5, 139.9, 138.8, 136.9, 134.5, 130.7, 130.1, 128.4, 127.6, 127.6, 127.4, 126.5, 125.6, 125.5, 122.5, 121.9, 120.8, 113.4, 74.1, 63.7, 14.0; HRMS (ESI) m/z calcd for $C_{25}H_{20}NO_3S^+$ $(M+H)^+$ 414.1158, found 414.1158. $[\alpha]^{20}_D$ = -63.9 (*c* 0.2, DCM). Chiral HPLC: Chiralpak AD-H, hexane:^{i}PrOH = 80:20, 1.0 mL/min, 400 nm; tR = 21.7 min (major), 27.7 min (minor).

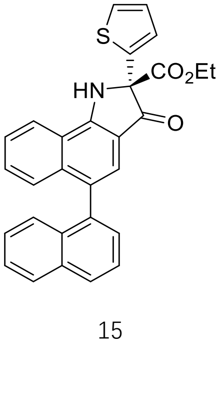

Ethyl (*R*)-5-(naphthalen-1-yl)-3-oxo-2-(thiophen-2-yl)-2,3-dihydro-1*H*-benzo[*g*]indole-2-carboxylate (4dq).

Product **4dq** was obtained by flash chromatography (DCM) in 82% yield as yellow solid. 1:1 dr. 97% ee. M.p.: >250 °C. ^{1}H NMR ($CDCl_3$) δ: 8.23 (d, J = 8.1 Hz, 1H), 7.97-7.88 (m, 2H), 7.65-7.52 (m, 4H), 7.52-7.34 (m, 5H), 7.34-7.22 (m, 2H), 7.10-7.02 (m, 1H), 6.57 (br, d, J = 1.9 Hz, 1H), 4.45-4.25 (m, 2H), 1.41-1.33 (m, 3H); ^{13}C NMR ($CDCl_3$) δ: 191.4, 166.8, 161.8, 138.8, 138.0, 137.4, 133.6, 132.9, 132.5, 130.7, 128.3, 128.2, 128.1, 127.6, 126.4, 126.0, 125.6, 125.5, 122.5, 121.9, 121.7, 113.5, 74.1, 63.7, 14.1; HRMS (ESI) m/z calcd for $C_{29}H_{22}NO_3S^+$ (M+H)$^+$ 464.1315, found 464.1315. $[\alpha]^{20}_D$ = -17.5 (*c* 0.2, DCM). Chiral HPLC: Chiralpak AD-H, hexane:^{i}PrOH = 80:20, 1.0 mL/min, 400 nm; tR = 10.1 min (major), 11.3 min (minor), 30.1 min (major), 67.7 min (minor).

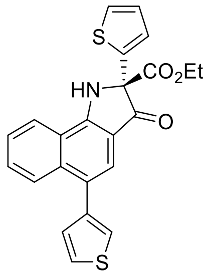

Ethyl (*R*)-3-oxo-2-(thiophen-2-yl)-5-(thiophen-3-yl)-2,3-dihydro-1*H*-benzo[*g*]indole-2-carboxylate (4eq).

Product **4eq** was obtained by flash chromatography (DCM) in 91% yield as yellow solid. 97% ee. M.p.: 174-176 °C. ^{1}H NMR ($CDCl_3$) δ: 8.17 (dd, J = 8.2, 0.7 Hz, 1H), 8.02 (d, J = 8.0 Hz, 1H), 7.70-7.64 (m, 1H), 7.64-7.59 (m, 1H), 7.57 (dd, J = 3.7, 1.2 Hz, 1H), 7.54 (s, 1H), 7.44 (dd, J = 4.9, 3.0 Hz, 1H), 7.32 (dd, J = 3.0, 1.2 Hz, 1H), 7.25 (dd, J = 5.1, 1.2 Hz, 1H), 7.21 (dd, J = 4.9, 1.2 Hz, 1H), 7.05 (dd, J = 5.1, 3.7 Hz, 1H), 6.52 (br, s, 1H), 4.41-4.25 (m, 2H), 1.35 (t, J = 7.1 Hz, 3H); ^{13}C NMR ($CDCl_3$) δ: 191.4, 166.8, 161.5, 140.2, 138.7, 137.0, 130.8, 129.4, 129.1, 127.5, 127.4, 126.5, 125.6, 125.6, 125.5, 123.6, 122.6, 121.8, 120.9, 113.3, 74.1, 63.7, 14.0; HRMS (ESI) m/z calcd for $C_{23}H_{18}NO_3S_2^+$ (M+H)$^+$ 420.0723, found 420.0722. $[\alpha]^{20}_D$ = -69.6 (*c* 0.2, DCM). Chiral HPLC: Chiralpak AD-H, hexane:^{i}PrOH = 80:20, 1.0 mL/min, 400 nm; tR = 26.9 min (major), 31.8 min (minor).

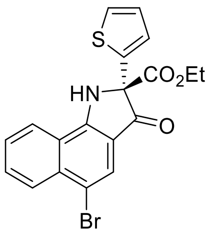

Ethyl (*R*)-5-bromo-3-oxo-2-(thiophen-2-yl)-2,3-dihydro-1*H*-benzo[*g*]indole-2-carboxylate (4fq).

Product **4fq** was obtained by flash chromatography (DCM) in 73% yield as yellow solid. 94% ee. M.p.: 193-195 °C. ^{1}H NMR ($CDCl_3$) δ: 8.27 (d, J = 8.4 Hz, 1H), 8.10 (d, J = 8.1 Hz, 1H), 7.86-7.77 (m, 2H), 7.68-7.62 (m, 1H), 7.53 (dd, J = 3.7, 1.2 Hz, 1H), 7.26 (dd, J = 5.1, 1.2 Hz, 1H), 7.04 (dd, J = 5.1, 3.7 Hz, 1H), 6.54 (br, s, 1H), 4.41-4.25 (m, 2H), 1.35 (t, J = 7.1 Hz, 3H); ^{13}C NMR ($CDCl_3$) δ: 190.1, 166.5, 161.3, 138.2, 135.9, 131.8, 128.8, 127.6, 127.4, 125.7, 125.6, 124.0, 122.6, 122.5, 115.1, 114.2, 74.1, 63.8, 14.0; HRMS (ESI) m/z calcd for $C_{19}H_{15}BrNO_3S^+$ (M+H)$^+$ 415.9951, found 415.9951. $[\alpha]^{20}_D$ = -89.0 (*c* 0.2, DCM). Chiral HPLC: Chiralpak AS-H, hexane:^{i}PrOH = 80:20, 1.0

mL/min, 400 nm; tR = 9.8 min (major), 12.4 min (minor).

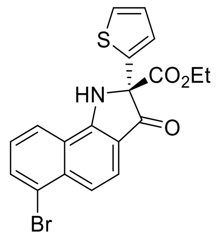

Ethyl (*R*)-6-bromo-3-oxo-2-(thiophen-2-yl)-2,3-dihydro-1*H*-benzo[*g*]indole-2-carboxylate (4gq).

Product **4gq** was obtained by flash chromatography (DCM) in 94% yield as yellow solid. 98% ee. M.p.: 132-135 °C. ^{1}H NMR ($CDCl_3$) δ: 8.08 (d, J = 8.2 Hz, 1H), 7.99 (d, J = 7.5 Hz, 1H), 7.78 (d, J = 8.9 Hz, 1H), 7.63 (d, J = 8.9 Hz, 1H), 7.54 (dd, J = 3.7, 1.0 Hz, 1H), 7.44 (t, J = 7.9 Hz, 1H), 7.28-7.23 (m, 1H), 7.05 (dd, J = 5.1, 3.7 Hz, 1H), 6.46 (br, s, 1H), 4.41-4.25 (m, 2H), 1.35 (t, J = 7.1 Hz, 3H); ^{13}C NMR ($CDCl_3$) δ: 191.2, 166.6, 161.7, 138.4, 136.8, 134.6, 127.6, 126.8, 125.7, 125.6, 124.1, 122.9, 121.9, 121.8, 121.0, 114.5, 74.3, 63.8, 14.0; HRMS (ESI) m/z calcd for $C_{19}H_{15}BrNO_3S^+$ $(M+H)^+$ 415.9951, found 415.9951. $[\alpha]^{20}_D$ = -67.5 (c 0.2, DCM). Chiral HPLC: Chiralpak AS-H, hexane:^{i}PrOH = 80:20, 1.0 mL/min, 400 nm; tR = 12.2 min (major), 17.6 min (minor).

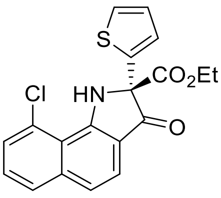

Ethyl (*R*)-9-chloro-3-oxo-2-(thiophen-2-yl)-2,3-dihydro-1*H*-benzo[*g*]indole-2-carboxylate (4hq).

Product **4hq** was obtained by flash chromatography (DCM) in 99% yield as yellow solid. 99% ee. M.p.: 177-179 °C. ^{1}H NMR ($CDCl_3$) δ: 7.75-7.66 (m, 2H), 7.58-7.45 (m, 4H), 7.29-7.21 (m, 2H), 7.04 (dd, J = 5.0, 3.8 Hz, 1H), 4.40-4.24 (m, 2H), 1.33 (t, J = 7.1 Hz, 3H); ^{13}C NMR ($CDCl_3$) δ: 191.0, 166.2, 161.1, 141.1, 138.0, 131.6, 130.0, 128.3, 127.4, 125.7, 125.5, 121.9, 121.4, 119.3, 114.4, 73.7, 63.5, 14.0; HRMS (ESI) m/z calcd for $C_{19}H_{15}ClNO_3S^+$ $(M+H)^+$ 372.0456, found 372.0457. $[\alpha]^{20}_D$ = -237.3 (c 0.2, DCM). Chiral HPLC: Chiralpak AS-H, hexane:^{i}PrOH = 80:20, 1.0 mL/min, 400 nm; tR = 9.5 min (major), 10.7 min (minor).

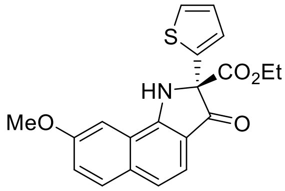

Ethyl (*R*)-8-methoxy-3-oxo-2-(thiophen-2-yl)-2,3-dihydro-1*H*-benzo[*g*]indole-2-carboxylate (4iq).

Product **4iq** was obtained by flash chromatography (DCM) in 61% yield as yellow solid. 98% ee. M.p.: 118-120 °C. ^{1}H NMR ($CDCl_3$) δ: 7.80-7.76 (m, 1H), 7.57 (dd, J = 3.7, 1.2 Hz, 1H), 7.42 (d, J = 8.5 Hz, 1H), 7.37-7.30 (m, 3H), 7.25 (dd, J = 5.1, 1.2 Hz, 1H), 7.05 (dd, J = 5.1, 3.7 Hz, 1H), 6.26 (br, s, 1H), 4.41-4.25 (m, 2H), 4.02 (s, 3H), 1.35 (t, J = 7.1 Hz, 3H); ^{13}C NMR ($CDCl_3$) δ: 191.8, 166.9, 161.1, 158.3, 138.9, 133.4, 130.5, 127.5, 125.6, 125.4, 122.7, 122.4, 121.9, 118.0, 114.7,

101.2, 74.0, 63.6, 55.7, 14.0; HRMS (ESI) m/z calcd for $C_{20}H_{18}NO_4S^+$ $(M+H)^+$ 368.0951, found 368.0951. $[\alpha]^{20}_D$ = -100.9 (*c* 0.1, DCM). Chiral HPLC: Chiralpak AS-H, hexane:^{i}PrOH = 80:20, 1.0 mL/min, 400 nm; tR = 9.9 min (major), 20.3 min (minor).

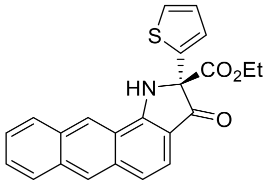

Ethyl (*R*)-3-oxo-2-(thiophen-2-yl)-2,3-dihydro-1*H*-naphtho[2,3-*g*]indole-2-carboxylate (4jq). Product **4jq** was obtained by flash chromatography (PE:EA = 10:1) in 93% yield as orange-yellow solid. 97% ee. M.p.: 159-162 °C. ^{1}H NMR ($CDCl_3$) δ: 8.62 (s, 1H), 8.26 (s, 1H), 7.98 (t, J = 7.9 Hz, 2H), 7.62-7.50 (m, 3H), 7.39 (d, J = 8.9 Hz, 1H), 7.33 (d, J = 8.9 Hz, 1H), 7.28-7.25 (m, 1H), 7.05 (dd, J = 5.1, 3.7 Hz, 1H), 6.90 (br, s, 1H), 4.43-4.28 (m, 2H), 1.36 (t, J = 7.1 Hz, 3H); ^{13}C NMR ($CDCl_3$) δ: 190.8, 167.0, 164.0, 138.7, 134.5, 134.4, 131.3, 128.9, 128.2, 127.7, 127.5, 127.5, 126.5, 125.7, 125.5, 122.4, 121.9, 120.5, 119.0, 112.0, 74.2, 63.7, 14.0; HRMS (ESI) m/z calcd for $C_{23}H_{18}NO_3S^+$ $(M+H)^+$ 388.1002, found 388.1003. $[\alpha]^{20}_D$ = -211.1 (*c* 0.2, DCM). Chiral HPLC: Chiralpak AS-H, hexane:^{i}PrOH = 80:20, 1.0 mL/min, 400 nm; tR = 14.0 min (major), 16.3 min (minor).

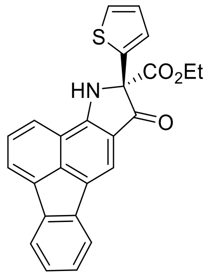

Ethyl (*S*)-6-oxo-5-(thiophen-2-yl)-5,6-dihydro-4*H*-fluoreno[9,1-*fg*]indole-5-carboxylate (4kq). Product **4kq** was obtained by flash chromatography (PE:EA = 4:1) in 99% yield as yellow solid. 96% ee. M.p.: 109-112 °C. ^{1}H NMR ($CDCl_3$) δ: 7.91 (s, 1H), 7.86 (d, J = 8.1 Hz, 1H), 7.82-7.77 (m, 2H), 7.74 (d, J = 7.0 Hz, 1H), 7.56 (dd, J = 3.7, 1.1 Hz, 1H), 7.46 (t, J = 7.8 Hz, 1H), 7.38 (td, J = 7.3, 1.0 Hz, 1H), 7.33 (td, J = 7.5, 0.9 Hz, 1H), 7.28 (dd, J = 5.1, 1.1 Hz, 1H), 7.11 (br, s, 1H), 7.05 (dd, J = 5.1, 3.8 Hz, 1H), 4.45-4.31 (m, 2H), 1.37 (t, J = 7.1 Hz, 3H); ^{13}C NMR ($CDCl_3$) δ: 191.0, 166.9, 163.1, 138.8, 138.5, 138.3, 138.2, 137.4, 130.4, 128.1, 127.4, 126.9, 125.7, 123.4, 121.5, 121.2, 121.1, 118.4, 115.6, 115.3, 74.7, 63.8, 14.0; HRMS (ESI) m/z calcd for $C_{25}H_{18}NO_3S^+$ $(M+H)^+$ 412.1002, found 412.1002. $[\alpha]^{20}_D$ = -177.7 (*c* 0.2, DCM). Chiral HPLC: Chiralpak AD-H, hexane:^{i}PrOH = 80:20, 1.0 mL/min, 400 nm; tR = 17.5 min (minor), 21.3 min (major).

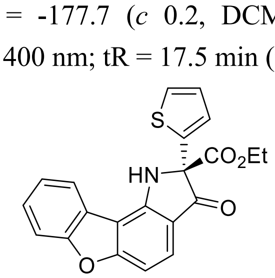

Ethyl (*R*)-3-oxo-2-(thiophen-2-yl)-2,3-dihydro-1*H*-benzofuro[2,3-*g*]indole-2-carboxylate (4lq). Product **4lq** was obtained by flash chromatography (DCM) in 56% yield as yellow solid. 96% ee. M.p.: 198-201 °C. ^{1}H NMR ($CDCl_3$) δ: 7.99 (dd, J = 7.5, 0.6 Hz, 1H), 7.71 (d, J = 8.6 Hz, 1H), 7.62 (d, J = 8.1 Hz, 1H), 7.56 (dd, J = 3.7, 1.2 Hz, 1H), 7.51 (td, J = 7.4, 1.3 Hz, 1H), 7.44 (td, J = 7.5,

1.0 Hz, 1H), 7.26 (dd, J = 5.1, 1.2 Hz, 1H), 7.18 (d, J = 8.6 Hz, 1H), 7.05 (dd, J = 5.1, 3.7 Hz, 1H), 6.37 (br, s, 1H), 4.42-4.27 (m, 2H), 1.35 (t, J = 7.1 Hz, 3H); ^{13}C NMR ($CDCl_3$) δ: 190.3, 166.9, 162.6, 156.9, 156.0, 138.7, 127.5, 127.1, 125.7, 125.6, 124.9, 123.9, 122.2, 121.4, 114.0, 111.9, 110.2, 106.4, 74.2, 63.7, 14.0; HRMS (ESI) m/z calcd for $C_{23}H_{18}NO_3S^+$ (M+H)$^+$ 388.1002, found 388.1003. $[\alpha]^{20}_D$ = -144.0 (c 0.03, DCM). Chiral HPLC: Chiralpak AS-H, hexane:^{i}PrOH = 80:20, 1.0 mL/min, 400 nm; tR = 11.3 min (major), 13.2 min (minor).

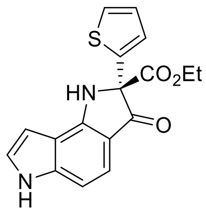

Ethyl (*R*)-3-oxo-2-(thiophen-2-yl)-1,2,3,6-tetrahydropyrrolo[2,3-*e*]indole-2-carboxylate (4mq). Product **4mq** was obtained by flash chromatography (PE:EA = 2:1) in 81% yield as yellow solid. 97% ee. M.p.: 234-236 °C. ^{1}H NMR (acetone-d_6) δ: 10.86 (br, s, 1H), 7.88 (br, s, 1H), 7.47 (dd, J = 3.6, 1.0 Hz, 1H), 7.39-7.33 (m, 2H), 7.20 (d, J = 8.6 Hz, 1H), 7.05-6.97 (m, 2H), 6.89 (t, J = 2.4 Hz, 1H), 4.19 (q, J = 7.1 Hz, 2H), 1.20 (t, J = 7.1 Hz, 3H); ^{13}C NMR (acetone-d_6) δ: 190.5, 167.2, 158.4, 141.7, 139.9, 126.7, 125.3, 125.1, 124.3, 117.6, 113.3, 108.3, 105.7, 101.3, 73.9, 62.1, 13.4; HRMS (ESI) m/z calcd for $C_{17}H_{15}N_2O_3S^+$ (M+H)$^+$ 327.0798, found 327.0798. $[\alpha]^{20}_D$ = +22.2 (c 0.1, DCM). Chiral HPLC: Chiralpak AS-H, hexane:^{i}PrOH = 80:20, 1.0 mL/min, 400 nm; tR = 10.8 min (major), 12.5 min (minor).

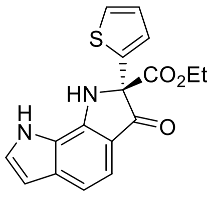

Ethyl (*R*)-3-oxo-2-(thiophen-2-yl)-1,2,3,8-tetrahydropyrrolo[3,2-*g*]indole-2-carboxylate (4nq). Product **4nq** was obtained by flash chromatography (PE:EA = 2:1) in 22% yield as yellow solid. 62% ee. M.p.: 218-220 °C. ^{1}H NMR (DMSO-d_6) δ: 11.28 (br, s, 1H), 8.17 (br, s, 1H), 7.65 (d, J = 2.8 Hz, 1H), 7.47 (dd, J = 5.1, 1.3 Hz, 1H), 7.35 (dd, J = 3.6, 1.3 Hz, 1H), 7.09-7.05 (m, 2H), 7.02 (d, J = 8.4 Hz, 1H), 6.58 (dd, J = 2.8, 2.0 Hz, 1H), 4.20 (q, J = 7.1 Hz, 2H), 1.18 (t, J = 7.1 Hz, 3H); ^{13}C NMR (DMSO-d_6) δ: 191.0, 167.5, 151.1, 139.7, 134.7, 129.8, 127.6, 126.2, 125.7, 121.1, 115.3, 113.6, 109.7, 104.5, 73.7, 62.8, 14.4; HRMS (ESI) m/z calcd for $C_{17}H_{15}N_2O_3S^+$ (M+H)$^+$ 327.0798, found 327.0799. $[\alpha]^{20}_D$ = -70.4 (c 0.04, DCM). Chiral HPLC: Chiralpak AS-H, hexane:^{i}PrOH = 80:20, 1.0 mL/min, 400 nm; tR = 11.9 min (major), 14.1 min (minor).

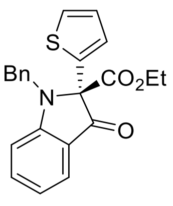

Ethyl (*R*)-1-benzyl-3-oxo-2-(thiophen-2-yl)indoline-2-carboxylate (4pq).
Product **4pq** was obtained by flash chromatography (DCM) in 82% yield as yellow oil. 90% ee. ^{1}H NMR ($CDCl_3$) δ: 7.68-7.64 (m, 1H), 7.46-7.39 (m, 1H), 7.33-7.28 (m, 2H), 7.28-7.23 (m, 2H), 7.23-7.19 (m, 2H), 7.17 (dd, J = 3.7, 1.2 Hz, 1H), 7.00 (dd, J = 5.1, 3.7 Hz, 1H), 6.87-6.81 (m, 1H), 6.65

(d, J = 8.4 Hz, 1H), 4.66 (q, J = 17.1 Hz, 2H), 4.20-4.10 (m, 1H), 4.09-3.98 (m, 1H), 1.16 (t, J = 7.2 Hz, 3H); ^{13}C NMR (CDCl$_3$) δ: 193.2, 166.7, 160.9, 138.0, 136.8, 136.0, 128.6, 127.5, 127.3, 127.0, 126.9, 126.6, 126.1, 119.0, 118.2, 110.1, 77.8, 62.8, 49.0, 13.9; HRMS (ESI) m/z calcd for $C_{22}H_{20}NO_3S^+$ (M+H)$^+$ 378.1158, found 378.1159. $[\alpha]^{20}_D$ = -171.6 (c 0.1, DCM). Chiral HPLC: Chiralpak IE-3, hexane:^{i}PrOH = 90:10, 1.0 mL/min, 400 nm; tR = 19.9 min (minor), 21.6 min (major).

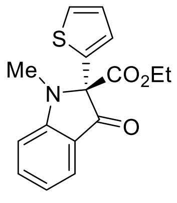

Ethyl (*R*)-1-methyl-3-oxo-2-(thiophen-2-yl)indoline-2-carboxylate (4qq).

Product **4qq** was obtained by flash chromatography (DCM) in 99% yield as yellow oil. 92% ee. ^{1}H NMR (CDCl$_3$) δ: 7.59 (d, J = 7.8 Hz, 1H), 7.58-7.52 (m, 1H), 7.29 (dd, J = 5.1, 0.9 Hz, 1H), 7.26-7.24 (m, 1H), 7.03 (dd, J = 5.0, 3.8 Hz, 1H), 6.89 (d, J = 8.3 Hz, 1H), 6.82 (t, J = 7.4 Hz, 1H), 4.37-4.19 (m, 2H), 3.09 (s, 3H), 1.27 (t, J = 7.1 Hz, 3H); ^{13}C NMR (CDCl$_3$) δ: 192.4, 165.9, 161.3, 138.2, 135.9, 127.2, 126.7, 126.2, 126.2, 118.6, 117.4, 108.6, 78.2, 62.8, 30.5, 14.1; HRMS (ESI) m/z calcd for $C_{16}H_{16}NO_3S^+$ (M+H)$^+$ 302.0845, found 302.0849. $[\alpha]^{20}_D$ = -250.9 (c 0.1, DCM). Chiral HPLC: Chiralpak AD-H, hexane:^{i}PrOH = 80:20, 1.0 mL/min, 400 nm; tR = 7.9 min (minor), 9.3 min (major).

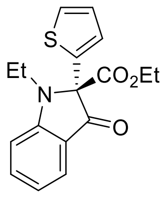

Ethyl (*R*)-1-ethyl-3-oxo-2-(thiophen-2-yl)indoline-2-carboxylate (4rq).

Product **4rq** was obtained by flash chromatography (DCM) in 93% yield as yellow oil. 91% ee. ^{1}H NMR (CDCl$_3$) δ: 7.62 (d, J = 7.7 Hz, 1H), 7.58-7.52 (m, 1H), 7.31 (dd, J = 5.1, 0.8 Hz, 1H), 7.19 (dd, J = 3.6, 0.8 Hz, 1H), 7.02 (dd, J = 4.9, 3.9 Hz, 1H), 6.91 (d, J = 8.4 Hz, 1H), 6.83 (t, J = 7.5 Hz, 1H), 4.29 (q, J = 7.1 Hz, 2H), 3.60-3.46 (m, 2H), 1.28 (t, J = 7.1 Hz, 3H), 1.23 (t, J = 7.2 Hz, 3H); ^{13}C NMR (CDCl$_3$) δ: 193.3, 167.0, 160.6, 138.1, 136.4, 127.1, 127.0, 126.4, 126.3, 118.3, 117.7, 108.9, 77.6, 62.7, 39.3, 14.0, 13.3; HRMS (ESI) m/z calcd for $C_{17}H_{17}NO_3SNa^+$ (M+Na)$^+$ 338.0821, found 338.0818. $[\alpha]^{20}_D$ = -255.0 (c 0.1, DCM). Chiral HPLC: Chiralpak AD-H, hexane:^{i}PrOH = 80:20, 1.0 mL/min, 400 nm; tR = 6.4 min (minor), 7.1 min (major).

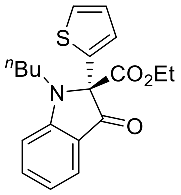

Ethyl (*R*)-1-butyl-3-oxo-2-(thiophen-2-yl)indoline-2-carboxylate (4sq).

Product **4sq** was obtained by flash chromatography (DCM) in 99% yield as yellow oil. 91% ee. ^{1}H NMR (CDCl$_3$) δ: 7.60 (dd, J = 7.7, 0.5 Hz, 1H), 756-7.49 (m, 1H), 7.29 (dd, J = 5.1, 1.2 Hz, 1H), 7.15 (dd, J = 3.7, 1.2 Hz, 1H), 7.0 (dd, J = 5.1, 3.7 Hz, 1H), 6.86 (d, J = 8.4 Hz, 1H), 6.80 (t, J = 7.6 Hz, 1H), 4.33-4.20 (m, 2H), 3.48-3.31 (m, 2H), 1.63-1.52 (m, 2H), 1.36-1.28 (m, 2H), 1.26 (t, J =

7.1 Hz, 3H), 0.91 (t, J = 7.3 Hz, 3H); ^{13}C NMR ($CDCl_3$) δ: 193.4, 167.1, 161.0, 138.1, 136.3, 127.2, 127.0, 126.4, 126.3, 118.3, 117.6, 109.0, 77.6, 62.7, 45.2, 30.2, 20.4, 14.0, 13.8; HRMS (ESI) m/z calcd for $C_{19}H_{22}NO_3S^+$ $(M+H)^+$ 344.1315, found 344.1319. $[\alpha]^{20}_D$ = -288.8 (c 0.2, DCM). Chiral HPLC: Chiralpak IE-3, hexane:^{i}PrOH = 90:10, 1.0 mL/min, 400 nm; tR = 15.2 min (minor), 15.9 min (major).

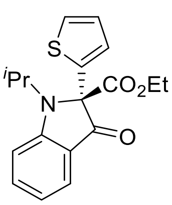

Ethyl (*R*)-1-isopropyl-3-oxo-2-(thiophen-2-yl)indoline-2-carboxylate (4tq).

Product **4tq** was obtained by flash chromatography (DCM) in 89% yield as yellow oil. 84% ee. ^{1}H NMR ($CDCl_3$) δ: 7.62 (dd, J = 7.7, 0.7 Hz, 1H), 7.51-7.46 (m, 1H), 7.28 (dd, J = 5.1, 1.2 Hz, 1H), 7.18 (dd, J = 3.7, 1.2 Hz, 1H), 7.02-6.96 (m, 2H), 6.78 (t, J = 7.2 Hz, 1H), 4.36-4.19 (m, 2H), 3.95-3.83 (m, 1H), 1.39 (d, J = 6.9 Hz, 3H), 1.32 (d, J = 6.9 Hz, 3H), 1.26 (t, J = 7.1 Hz, 3H); ^{13}C NMR ($CDCl_3$) δ: 194.0, 167.4, 159.2, 137.7, 136.8, 127.4, 126.8, 126.6, 126.2, 118.5, 117.9, 110.9, 77.2, 62.6, 48.0, 20.5, 19.5, 14.0; HRMS (ESI) m/z calcd for $C_{18}H_{20}NO_3S^+$ $(M+H)^+$ 330.1158, found 330.1158. $[\alpha]^{20}_D$ = -154.5 (c 0.1, DCM). Chiral HPLC: Chiralpak AD-H, hexane:^{i}PrOH = 98:2, 1.0 mL/min, 400 nm; tR = 19.2 min (minor), 21.1 min (major).

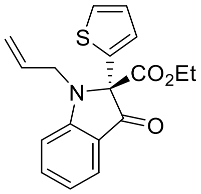

Ethyl (*R*)-1-allyl-3-oxo-2-(thiophen-2-yl)indoline-2-carboxylate (4uq).

Product **4uq** was obtained by flash chromatography (DCM) in 91% yield as yellow oil. 91% ee. ^{1}H NMR ($CDCl_3$) δ: 7.61 (dd, J = 7.7, 0.6 Hz, 1H), 7.55-7.47 (m, 1H), 7.29 (dd, J = 5.1, 1.2 Hz, 1H), 7.21 (dd, J = 3.7, 1.2 Hz, 1H), 7.01 (dd, J = 5.1, 3.7 Hz, 1H), 6.92 (t, J = 8.4 Hz, 1H), 6.83 (t, J = 7.7 Hz, 1H), 5.96-5.83 (m, 1H), 5.31-5.24 (m, 1H), 5.23-5.17 (m, 1H), 4.25 (q, J = 7.1 Hz, 2H), 4.10-4.05 (m, 2H), 1.26 (t, J = 7.1 Hz, 3H); ^{13}C NMR ($CDCl_3$) δ: 192.8, 166.6, 160.8, 137.9, 136.2, 133.4, 127.1, 127.1, 126.3, 126.1, 118.8, 117.9, 117.6, 109.9, 77.8, 62.7, 48.2, 14.0; HRMS (ESI) m/z calcd for $C_{18}H_{18}NO_3S^+$ $(M+H)^+$ 328.1002, found 328.1003. $[\alpha]^{20}_D$ = -177.9 (c 0.1, DCM). Chiral HPLC: Chiralpak AD-H, hexane:^{i}PrOH = 90:10, 1.0 mL/min, 400 nm; tR = 8.7 min (minor), 9.5 min (major).

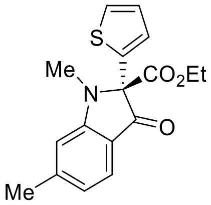

Ethyl (*R*)-1,6-dimethyl-3-oxo-2-(thiophen-2-yl)indoline-2-carboxylate (4vq).

Product **4vq** was obtained by flash chromatography (DCM) in 99% yield as yellow oil. 96% ee. ^{1}H NMR ($CDCl_3$) δ: 7.47 (d, J = 7.9 Hz, 1H), 7.27 (dd, J = 5.1, 1.2 Hz, 1H), 7.25 (dd, J = 3.7, 1.2 Hz, 1H), 7.02 (dd, J = 5.1, 3.7 Hz, 1H), 6.68 (s, 1H), 6.64 (d, J = 7.9 Hz, 1H), 4.35-4.19 (m, 2H), 3.07 (s, 3H), 2.42 (s, 3H), 1.27 (t, J = 7.1 Hz, 3H); ^{13}C NMR ($CDCl_3$) δ: 191.7, 166.1, 161.7, 150.1, 136.2,

127.2, 126.7, 126.0, 126.0, 120.3, 115.2, 108.8, 78.5, 62.7, 30.4, 22.8, 14.1; HRMS (ESI) m/z calcd for $C_{17}H_{18}NO_3S^+$ $(M+H)^+$ 316.1002, found 316.1006. $[\alpha]^{20}_D$ = -275.2 (*c* 0.2, DCM). Chiral HPLC: Chiralpak IE-3, hexane:^{i}PrOH = 80:20, 1.0 mL/min, 400 nm; tR = 15.3 min (minor), 17.8 min (major).

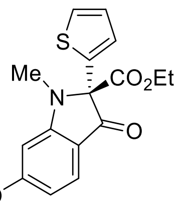

Ethyl (*R*)-6-methoxy-1-methyl-3-oxo-2-(thiophen-2-yl)indoline-2-carboxylate (4wq).

Product **4wq** was obtained by flash chromatography (DCM) in 99% yield as yellow oil. 96% ee. ^1H NMR ($CDCl_3$) δ: 7.50 (d, J = 8.6 Hz, 1H), 7.28 (dd, J = 5.1, 1.2 Hz, 1H), 7.24 (dd, J = 3.7, 1.2 Hz, 1H), 7.02 (dd, J = 5.1, 3.7 Hz, 1H), 6.39 (dd, J = 8.6, 2.0 Hz, 1H), 6.25 (d, J = 1.9 Hz, 1H), 4.37-4.19 (m, 2H), 3.92 (s, 3H), 3.07 (s, 3H), 1.28 (t, J = 7.1 Hz, 3H); ^{13}C NMR ($CDCl_3$) δ: 190.0, 168.4, 166.1, 163.5, 136.3, 127.8, 127.1, 126.6, 126.0, 110.8, 107.8, 91.7, 78.8, 62.7, 55.7, 30.5, 14.2; HRMS (ESI) m/z calcd for $C_{17}H_{18}NO_4S^+$ $(M+H)^+$ 332.0951, found 332.0950. $[\alpha]^{20}_D$ = -194.3 (*c* 0.2, DCM). Chiral HPLC: Chiralpak IE-3, hexane:^{i}PrOH = 80:20, 1.0 mL/min, 400 nm; tR = 25.3 min (minor), 31.0 min (major).

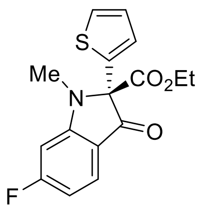

Ethyl (*R*)-6-fluoro-1-methyl-3-oxo-2-(thiophen-2-yl)indoline-2-carboxylate (4xq).

Product **4xq** was obtained by flash chromatography (DCM) in 95% yield as yellow oil. 93% ee. ^1H NMR ($CDCl_3$) δ: 7.61-7.55 (m, 1H), 7.30 (dd, J = 5.1, 1.2 Hz, 1H), 7.23 (dd, J = 3.7, 1.2 Hz, 1H), 7.03 (dd, J = 5.1, 3.7 Hz, 1H), 6.56-6.47 (m, 2H), 4.38-4.21 (m, 2H), 3.07 (s, 3H), 1.28 (t, J = 7.1 Hz, 3H); ^{13}C NMR ($CDCl_3$) δ: 190.5, 170.2 (d, J = 257.4 Hz), 165.6, 162.9 (d, J = 14.3 Hz), 135.4, 128.6 (d, J = 12.7 Hz), 127.2, 126.8, 126.4, 113.9, 107.1 (d, J = 24.8 Hz), 95.5 (d, J = 27.0 Hz), 78.8, 63.0, 30.6, 14.1; ^{19}F NMR ($CDCl_3$) δ: -97.2; HRMS (ESI) m/z calcd for $C_{16}H_{15}FNO_3S^+$ $(M+H)^+$ 320.0751, found 320.0754. $[\alpha]^{20}_D$ = -285.6 (*c* 0.1, DCM). Chiral HPLC: Chiralpak AD-H, hexane:^{i}PrOH = 80:20, 1.0 mL/min, 400 nm; tR = 6.7 min (minor), 8.3 min (major).

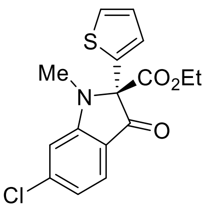

Ethyl (*R*)-6-chloro-1-methyl-3-oxo-2-(thiophen-2-yl)indoline-2-carboxylate (4yq).

Product **4yq** was obtained by flash chromatography (DCM) in 99% yield as yellow oil. 91% ee. ^1H NMR ($CDCl_3$) δ: 7.50 (d, J = 8.2 Hz, 1H), 7.30 (dd, J = 5.1, 1.2 Hz, 1H), 7.23 (dd, J = 3.7, 1.2 Hz, 1H), 7.03 (dd, J = 5.1, 3.7 Hz, 1H), 6.88 (d, J = 1.5 Hz, 2H), 6.78 (d, J = 8.2, 1.6 Hz, 1H), 4.38-4.21 (m, 2H), 3.07 (s, 3H), 1.28 (t, J = 7.1 Hz, 3H); ^{13}C NMR ($CDCl_3$) δ: 191.0, 165.5, 161.5, 144.9, 135.3, 127.2, 127.2, 126.8, 126.4, 119.3, 116.0, 108.8, 78.6, 63.0, 30.5, 14.1; HRMS (ESI) m/z calcd for $C_{16}H_{15}ClNO_3S^+$ $(M+H)^+$ 336.0456, found 336.0461. $[\alpha]^{20}_D$ = -330.0 (*c* 0.2, DCM). Chiral HPLC:

Chiralpak AD-H, hexane:^{i}PrOH = 80:20, 1.0 mL/min, 400 nm; tR = 6.3 min (minor), 9.9 min (major).

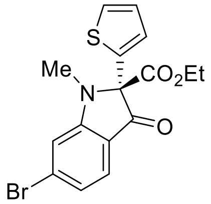

Ethyl (*R*)-6-bromo-1-methyl-3-oxo-2-(thiophen-2-yl)indoline-2-carboxylate (4zq).

Product **4zq** was obtained by flash chromatography (DCM) in 96% yield as yellow oil. 89% ee. ^{1}H NMR ($CDCl_3$) *δ*: 7.43 (d, *J* = 8.2 Hz, 1H), 7.30 (dd, *J* = 5.1, 0.9 Hz, 1H), 7.23 (dd, *J* = 3.6, 1.0 Hz, 1H), 7.07 (d, *J* = 1.1 Hz, 1H), 7.03 (dd, *J* = 5.0, 3.8 Hz, 1H), 6.94 (d, *J* = 8.2, 1.2 Hz, 1H), 4.37-4.20 (m, 2H), 3.07 (s, 3H), 1.28 (t, *J* = 7.1 Hz, 3H); ^{13}C NMR ($CDCl_3$) *δ*: 191.2, 165.4, 161.4, 135.2, 133.9, 127.2, 127.2, 126.8, 126.4, 122.1, 116.3, 111.9, 78.4, 63.0, 30.5, 14.1; HRMS (ESI) m/z calcd for $C_{16}H_{15}BrNO_3S^+$ (M+H)$^+$ 379.9951, found 379.9953. $[\alpha]^{20}_D$ = - 270.0 (*c* 0.2, DCM). Chiral HPLC: Chiralpak AS-H, hexane:^{i}PrOH = 80:20, 1.0 mL/min, 400 nm; tR = 6.3 min (minor), 10.8 min (major).

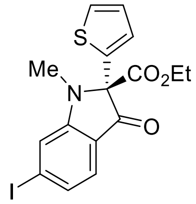

Ethyl (*R*)-6-iodo-1-methyl-3-oxo-2-(thiophen-2-yl)indoline-2-carboxylate (4Aq).

Product **4Aq** was obtained by flash chromatography (DCM) in 99% yield as yellow oil. 89% ee. ^{1}H NMR ($CDCl_3$) *δ*: 7.32 (d, *J* = 0.8 Hz, 1H), 7.30 (dd, *J* = 5.1, 1.2 Hz, 1H), 7.27 (d, *J* = 8.0 Hz, 1H), 7.22 (dd, *J* = 3.7, 1.2 Hz, 1H), 7.16 (dd, *J* = 8.1, 1.2 Hz, 1H), 7.02 (dd, *J* = 5.1, 3.7 Hz, 1H), 4.37-4.20 (m, 2H), 3.06 (s, 3H), 1.28 (t, *J* = 7.1 Hz, 3H); ^{13}C NMR ($CDCl_3$) *δ*: 191.6, 165.4, 161.1, 135.2, 128.0, 127.2, 126.9, 126.8, 126.4, 118.1, 116.9, 107.4, 78.2, 63.0, 30.5, 14.1; HRMS (ESI) m/z calcd for $C_{16}H_{15}INO_3S^+$ (M+H)$^+$ 427.9812, found 427.9816. $[\alpha]^{20}_D$ = - 253.8 (*c* 0.2, DCM). Chiral HPLC: Chiralpak AD-H, hexane:^{i}PrOH = 80:20, 1.0 mL/min, 400 nm; tR = 6.7 min (minor), 12.4 min (major).

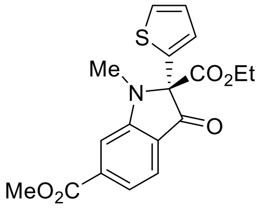

2-Ethyl 6-methyl (R)-1-methyl-3-oxo-2-(thiophen-2-yl)indoline-2,6-dicarboxylate (4Bq).

Product **4Bq** was obtained by flash chromatography (DCM) in 74% yield as yellow oil. 84% ee. ^{1}H NMR ($CDCl_3$) *δ*: 7.63 (d, *J* = 8.0 Hz, 1H), 7.56 (s, 1H), 7.46 (dd, *J* = 8.0, 1.1 Hz, 1H), 7.30 (dd, *J* = 5.1, 1.2 Hz, 1H), 7.25 (dd, *J* = 3.7, 1.2 Hz, 1H), 7.04 (dd, *J* = 5.1, 3.7 Hz, 1H), 4.37-4.20 (m, 2H), 3.97 (s, 3H), 3.14 (s, 3H), 1.27 (t, *J* = 7.1 Hz, 3H); ^{13}C NMR ($CDCl_3$) *δ*: 192.3, 166.4, 165.5, 160.7, 138.6, 135.3, 127.3, 126.9, 126.4, 126.1, 120.5, 119.3, 109.8, 78.6, 63.0, 52.7, 30.6, 14.1; HRMS (ESI) m/z calcd for $C_{18}H_{18}NO_5S^+$ (M+H)$^+$ 360.0900, found 360.0902. $[\alpha]^{20}_D$ = -96.5 (*c* 0.05, DCM). Chiral HPLC: Chiralpak AD-H, hexane:^{i}PrOH = 80:20, 1.0 mL/min, 400 nm; tR = 9.8 min (minor), 12.5 min (major).

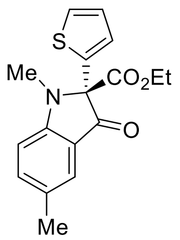

Ethyl (*R*)-1,5-dimethyl-3-oxo-2-(thiophen-2-yl)indoline-2-carboxylate (4Cq).

Product **4Cq** was obtained by flash chromatography (DCM) in 80% yield as yellow oil. 82% ee. ^{1}H NMR (CDCl$_3$) *δ*: 7.41-7.35 (m, 2H), 7.30-7.23 (m, 1H), 7.02 (dd, *J* = 5.0, 3.7 Hz, 1H), 6.81 (d, *J* = 8.9 Hz, 1H), 4.35-4.18 (m, 2H), 3.07 (s, 3H), 2.28 (s, 3H), 1.26 (t, *J* = 7.1 Hz, 3H); ^{13}C NMR (CDCl$_3$) *δ*: 192.5, 166.1, 159.9, 139.6, 136.2, 129.7, 128.1, 127.2, 126.7, 126.0, 125.6, 117.5, 108.5, 78.5, 62.7, 30.6, 20.4, 14.2; HRMS (ESI) m/z calcd for $C_{17}H_{18}NO_3S^+$ $(M+H)^+$ 316.1002, found 316.1002. $[\alpha]^{20}_D$ = -165.8 (*c* 0.1, DCM). Chiral HPLC: Chiralpak AD-H, hexane:^{i}PrOH = 80:20, 1.0 mL/min, 400 nm; tR = 7.0 min (minor), 9.4 min (major).

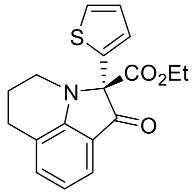

Ethyl (*R*)-1-oxo-2-(thiophen-2-yl)-1,2,5,6-tetrahydro-4*H*-pyrrolo[3,2,1-ij]quinoline-2-carboxylate (4Fq).

Product **4Fq** was obtained by flash chromatography (DCM) in 75% yield as yellow oil. 89% ee. ^{1}H NMR (CDCl$_3$) *δ*: 7.37 (d, *J* = 7.8 Hz, 1H), 7.29 (dd, *J* = 3.7, 1.1 Hz, 1H), 7.27 (dd, *J* = 5.2, 1.1 Hz, 1H), 7.21 (dd, *J* = 7.1, 0.7 Hz, 1H), 7.03 (dd, *J* = 5.1, 3.7 Hz, 1H), 6.68 (t, *J* = 7.5 Hz, 1H), 4.36-4.20 (m, 2H), 3.54-3.41 (m, 2H), 2.92-2.77 (m, 2H), 2.21-2.08 (m, 2H), 1.28 (t, *J* = 7.1 Hz, 3H); ^{13}C NMR (CDCl$_3$) *δ*: 192.3, 165.9, 159.0, 136.2, 135.5, 127.4, 126.5, 126.0, 125.8, 123.6, 120.9, 118.4, 114.7, 77.5, 62.7, 41.5, 24.6, 21.6, 14.2; HRMS (ESI) m/z calcd for $C_{18}H_{18}NO_3S^+$ $(M+H)^+$ 328.1002, found 328.1005. $[\alpha]^{20}_D$ = -300.6 (*c* 0.1, DCM). Chiral HPLC: Chiralpak AD-H, hexane:^{i}PrOH = 80:20, 1.0 mL/min, 400 nm; tR = 6.2 min (major), 6.8 min (minor).

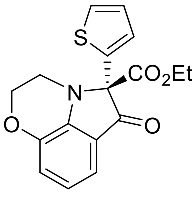

Ethyl (*R*)-6-oxo-5-(thiophen-2-yl)-2,3,5,6-tetrahydro-[1,4]oxazino[2,3,4-hi]indole-5-carboxylate (4Gq).

Product **4Gq** was obtained by flash chromatography (DCM) in 86% yield as yellow oil. 87% ee. ^{1}H NMR (CDCl$_3$) *δ*: 7.34 (dd, *J* = 3.7, 1.1 Hz, 1H), 7.28 (dd, *J* = 5.1, 1.1 Hz, 1H), 7.17 (dd, *J* = 7.9, 0.6 Hz, 1H), 7.06 (dd, *J* = 5.1, 3.7 Hz, 1H), 6.98 (dd, *J* = 7.7, 0.6 Hz, 1H), 6.72 (t, *J* = 7.8 Hz, 1H), 4.52-4.67 (m, 2H), 4.36-4.21 (m, 2H), 3.76-3.68 (m, 1H), 3.56 (dt, *J* = 11.7, 3.5 Hz, 1H), 1.29 (t, *J* = 7.1 Hz, 3H); ^{13}C NMR (CDCl$_3$) *δ*: 191.5, 165.4, 149.7, 142.6, 135.8, 127.7, 126.6, 125.8, 121.1, 119.7, 117.9, 116.7, 77.4, 65.8, 63.0, 40.9, 14.1; HRMS (ESI) m/z calcd for $C_{17}H_{15}NO_4SNa^+$ $(M+Na)^+$ 352.0614, found 352.0623. $[\alpha]^{20}_D$ = -267.4 (*c* 0.1, DCM). Chiral HPLC: Chiralpak IE-3, hexane:^{i}PrOH = 95:5, 1.0 mL/min, 400 nm; tR = 30.5 min (minor), 31.8 min (major).

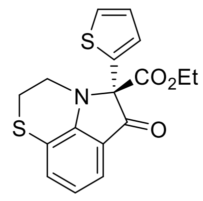

Ethyl (*R*)-6-oxo-5-(thiophen-2-yl)-2,3,5,6-tetrahydro-[1,4]thiazino[2,3,4-hi]indole-5-carboxylate (4Hq).

Product **4Hq** was obtained by flash chromatography (DCM) in 93% yield as yellow oil. 84% ee. ^{1}H NMR ($CDCl_3$) *δ*: 7.34 (dd, *J* = 7.8, 1.0 Hz, 1H), 7.31-7.26 (m, 3H), 7.04 (dd, *J* = 5.0, 3.8 Hz, 1H), 6.74 (t, *J* = 7.7 Hz, 1H), 4.39-4.23 (m, 2H), 3.78 (t, *J* = 4.9 Hz, 2H), 3.27 (dt, *J* = 13.3, 5.3 Hz, 1H), 3.10 (dt, *J* = 8.5, 4.5 Hz, 1H), 1.30 (t, *J* = 7.1 Hz, 3H); ^{13}C NMR ($CDCl_3$) *δ*: 191.4, 165.5, 154.5, 135.4, 134.1, 127.5, 126.9, 126.2, 122.4, 119.3, 116.2, 115.7, 77.7, 63.0, 42.5, 25.0, 14.1; HRMS (ESI) m/z calcd for $C_{17}H_{16}NO_3S_2^+$ $(M+H)^+$ 346.0566, found 346.0571. $[\alpha]^{20}_{D}$ = -374.9 (*c* 0.1, DCM). Chiral HPLC: Chiralpak AD-H, hexane:^{i}PrOH = 80:20, 1.0 mL/min, 400 nm; tR = 7.8 min (major), 9.0 min (minor).

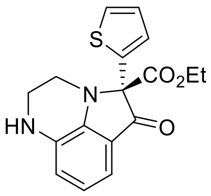

Ethyl (*R*)-6-oxo-5-(thiophen-2-yl)-2,3,5,6-tetrahydro-1*H*-pyrrolo[1,2,3-de]quinoxaline-5-carboxylate (4Iq).

Product **4Iq** was obtained by flash chromatography (PE:EA = 2:1) in 54% yield as yellow oil. 85% ee. ^{1}H NMR ($CDCl_3$) *δ*: 7.32 (dd, *J* = 3.7, 1.2 Hz, 1H), 7.27 (dd, *J* = 5.1, 1.2 Hz, 1H), 7.04 (dd, *J* = 5.1, 3.7 Hz, 1H), 6.97 (dd, *J* = 7.3, 1.5 Hz, 1H), 6.68-6.60 (m, 2H), 4.36-4.21 (m, 2H), 4.01 (br, s, 1H), 3.72-3.60 (m, 2H), 3.59-3.50 (m, 2H), 1.29 (t, *J* = 7.1 Hz, 3H); ^{13}C NMR ($CDCl_3$) *δ*: 192.3, 165.7, 150.7, 136.2, 132.3, 127.5, 126.5, 125.7, 119.7, 117.4, 115.2, 114.6, 78.2, 62.8, 41.1, 40.8, 14.2; HRMS (ESI) m/z calcd for $C_{17}H_{17}N_2O_3S^+$ $(M+H)^+$ 329.0954, found 329.0956. $[\alpha]^{20}_{D}$ = -252.9 (*c* 0.1, DCM). Chiral HPLC: Chiralpak IE-3, hexane:^{i}PrOH = 80:20, 1.0 mL/min, 400 nm; tR = 17.7 min (minor), 18.7 min (major).

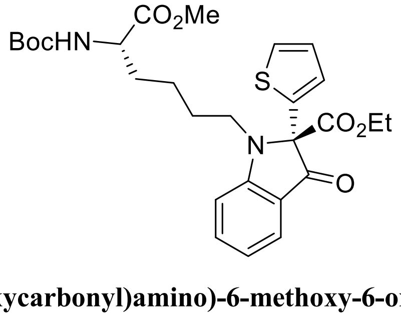

Ethyl (*R*)-1-((*S*)-5-((*tert*-butoxycarbonyl)amino)-6-methoxy-6-oxohexyl)-3-oxo-2-(thiophen-2-yl)indoline-2-carboxylate (4Jq).

Product **4Jq** was obtained by flash chromatography (PE:EA = 3:1) in 82% yield as yellow oil. 84% ee. ^{1}H NMR ($CDCl_3$) *δ*: 7.60 (d, *J* = 7.6 Hz, 1H), 7.57-7.50 (m, 1H), 7.30 (dd, *J* = 5.1, 1.0 Hz, 1H), 7.13 (dd, *J* = 3.6, 1.0 Hz, 1H), 7.01 (dd, *J* = 5.0, 3.8 Hz, 1H), 6.89-6.77 (m, 2H), 4.99 (d, *J* = 8.0 Hz, 1H), 4.34-4.19 (m, 3H), 3.72 (s, 3H), 3.48-3.26 (m, 2H), 1.84-1.51 (m, 4H), 1.44 (s, 9H), 1.34-1.27 (m, 2H), 1.26 (t, *J* = 7.1 Hz, 3H); ^{13}C NMR ($CDCl_3$) *δ*: 193.3, 173.2, 166.9, 160.7, 155.3, 138.2,

136.2, 127.3, 127.0, 126.5, 126.3, 118.5, 117.6, 109.0, 80.1, 77.6, 62.8, 53.2, 52.4, 45.2, 32.6, 28.3, 27.6, 22.9, 14.1; HRMS (ESI) m/z calcd for $C_{27}H_{34}N_2O_7SNa^+$ $(M+Na)^+$ 553.1979, found 553.1984. $[\alpha]^{20}_D$ = -60.5 (*c* 0.2, DCM). Chiral HPLC: Chiralpak AD-H, hexane:^{i}PrOH = 80:20, 1.0 mL/min, 400 nm; tR = 21.6 min (minor), 27.1 min (major).

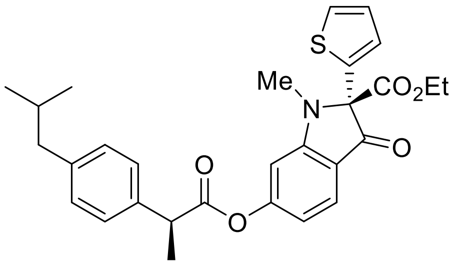

Ethyl (*R*)-6-(((*S*)-2-(4-isobutylphenyl)propanoyl)oxy)-1-methyl-3-oxo-2-(thiophen-2-yl)indoline-2-carboxylate (4Kq).

Product **4Kq** was obtained by flash chromatography (DCM) in 83% yield as yellow oil. 13:1 dr, 91% ee. ^{1}H NMR ($CDCl_3$) δ: 7.54 (d, *J* = 8.4 Hz, 1H), 7.33-7.27 (m, 3H), 7.23-7.19 (m, 1H), 7.16 (d, *J* = 8.0 Hz, 2H), 7.01 (dd, *J* = 4.9, 3.8 Hz, 1H), 6.56 (s, 1H), 6.43 (dt, *J* = 8.4, 1.5 Hz, 1H), 4.36-4.18 (m, 2H), 3.95 (q, *J* = 7.2 Hz, 1H), 3.02 (s, 3H), 2.48 (d, *J* = 7.2 Hz, 2H), 1.94-1.79 (m, 1H), 1.63 (d, *J* = 7.1 Hz, 3H), 1.26 (t, *J* = 7.1 Hz, 3H), 0.91 (d, *J* = 6.6 Hz, 6H); ^{13}C NMR ($CDCl_3$) δ: 191.0, 172.5, 165.7, 162.2, 159.3, 141.1, 136.7, 135.5, 129.6, 127.4, 127.2, 127.2, 126.8, 126.3, 114.9, 112.6, 101.5, 78.6, 62.9, 45.4, 45.1, 30.5, 30.2, 22.4, 18.4, 14.1; HRMS (ESI) m/z calcd for $C_{29}H_{31}NO_5SNa^+$ $(M+Na)^+$ 528.1815, found 528.1818. $[\alpha]^{20}_D$ = -158.5 (*c* 0.1, DCM). Chiral HPLC: Chiralpak AD-H, hexane:^{i}PrOH = 80:20, 1.0 mL/min, 400 nm; tR = 6.8 min (minor), 13.3 min (major).

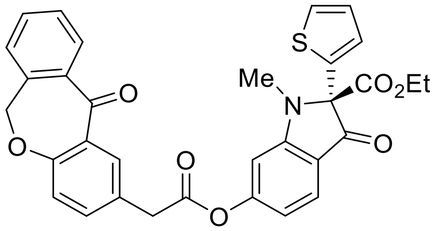

Ethyl (*R*)-1-methyl-3-oxo-6-(2-(11-oxo-6,11-dihydrodibenzo[*b*,*e*]oxepin-2-yl)acetoxy)-2-(thiophen-2-yl)indoline-2-carboxylate (4Lq).

Product **4Lq** was obtained by flash chromatography (DCM:EA = 50:1) in 99% yield as yellow oil. 91% ee. ^{1}H NMR ($CDCl_3$) δ: 8.24 (d, *J* = 2.3 Hz, 1H), 7.91 (dd, *J* = 7.7, 0.9 Hz, 1H), 7.62-7.55 (m, 2H), 7.55-7.46 (m, 2H), 7.39 (d, *J* = 7.1 Hz, 1H), 7.29 (dd, *J* = 5.1, 1.1 Hz, 1H), 7.22 (dd, *J* = 3.7, 1.1 Hz, 1H), 7.10 (d, *J* = 8.5 Hz, 1H), 7.02 (d, *J* = 5.1, 3.7 Hz, 1H), 6.67 (d, *J* = 1.7 Hz, 1H), 6.52 (dd, *J* = 8.4, 1.8 Hz, 1H), 5.22 (s, 2H), 4.36-4.19 (m, 2H), 3.92 (s, 2H), 3.05 (s, 3H), 1.27 (t, *J* = 7.1 Hz, 3H); ^{13}C NMR ($CDCl_3$) δ: 191.0, 190.8, 169.1, 165.7, 162.2, 160.8, 159.0, 140.4, 136.3, 135.5, 135.5, 132.9, 132.7, 129.5, 129.4, 127.9, 127.5, 127.2, 126.8, 126.7, 126.4, 125.3, 121.4, 115.1, 112.5, 101.6, 78.6, 76.7, 73.7, 62.9, 40.4, 30.5, 14.1; HRMS (ESI) m/z calcd for $C_{32}H_{25}NO_7SNa^+$ $(M+Na)^+$ 590.1244, found 590.1250. $[\alpha]^{20}_D$ = -190.3 (*c* 0.3, DCM). Chiral HPLC: Chiralpak AD-H, hexane:^{i}PrOH = 80:20, 1.0 mL/min, 400 nm; tR = 55.2 min (minor), 105.4 min (major).

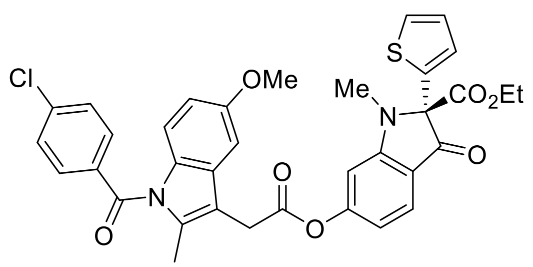

Ethyl (*R*)-6-(2-(1-(4-chlorobenzoyl)-5-methoxy-2-methyl-1*H*-indol-3-yl)acetoxy)-1-methyl-3-oxo-2-(thiophen-2-yl)indoline-2-carboxylate (4Mq).

Product **4Mq** was obtained by flash chromatography (PE:EA = 3:1) in 95 % yield as yellow oil. 91 % ee. ^{1}H NMR ($CDCl_3$) δ: 7.72-7.66 (m, 2H), 7.56 (d, J = 8.4 Hz, 1H), 7.52-7.45 (m, 2H), 7.29 (dd, *J* = 5.1, 1.1 Hz, 1H), 7.22 (dd, *J* = 3.7, 1.2 Hz, 1H), 7.06 (d, *J* = 2.4 Hz, 1H), 7.02 (dd, *J* = 5.1, 3.7 Hz, 1H), 6.89 (d, *J* = 9.0 Hz, 1H), 6.71 (dd, *J* = 9.0, 2.5 Hz, 1H), 6.64 (d, *J* = 1.7 Hz, 1H), 6.50 (dd, J = 8.4, 1.8 Hz, 1H), 4.36-4.19 (m, 2H), 3.94 (s, 2H), 3.85 (s, 3H), 3.04 (s, 3H), 2.48 (s, 3H), 1.27 (t, *J* = 7.1 Hz, 3H); ^{13}C NMR ($CDCl_3$) δ: 190.9, 168.5, 168.3, 165.6, 162.2, 159.0, 156.2, 139.5, 136.4, 135.4, 133.7, 131.2, 130.9, 130.4, 129.2, 127.5, 127.2, 126.8, 126.4, 115.1, 112.5, 111.8, 111.5, 101.5, 101.3, 78.7, 62.9, 55.8, 30.7, 30.5, 14.1, 13.5; HRMS (ESI) m/z calcd for $C_{35}H_{29}ClN_2O_7SNa^+$ $(M+Na)^+$ 679.1276, found 679.1274. $[\alpha]^{20}_{D}$ = -150.0 (*c* 0.3, DCM). Chiral HPLC: Chiralpak AD-H, hexane:^{i}PrOH = 80:20, 1.0 mL/min, 400 nm; tR = 39.4 min (minor), 63.9 min (major).

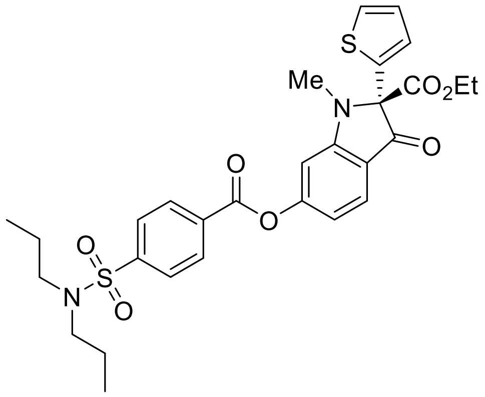

Ethyl (R)-6-((4-(*N*,*N*-dipropylsulfamoyl)benzoyl)oxy)-1-methyl-3-oxo-2-(thiophen-2-yl)indoline-2-carboxylate (4Nq).

Product **4Nq** was obtained by flash chromatography (PE:EA = 3:1) in 91 % yield as yellow oil. 91 % ee. ^{1}H NMR ($CDCl_3$) δ: 8.33 (d, *J* = 8.5 Hz, 2H), 7.98 (d, *J* = 8.5 Hz, 2H), 7.66 (d, *J* = 8.4 Hz, 1H), 7.32 (dd, *J* = 5.1, 1.1 Hz, 1H), 7.26 (dd, *J* = 3.7, 1.2 Hz, 1H), 7.04 (d, *J* = 5.1, 3.8 Hz, 1H), 6.80 (d, *J* = 1.7 Hz, 1H), 6.67 (dd, *J* = 8.4, 1.8 Hz, 1H), 4.39-4.22 (m, 2H), 3.18-3.12 (m, 4H), 3.10 (s, 3H), 1.64-1.52 (m, 4H), 1.30 (t, *J* = 7.1 Hz, 3H), 0.89 (t, *J* = 7.4 Hz, 6H); ^{13}C NMR ($CDCl_3$) δ: 190.9, 165.6, 163.2, 162.2, 158.9, 145.4, 135.4, 132.2, 130.9, 127.7, 127.3, 127.2, 126.8, 126.4, 115.4, 112.5, 101.7, 78.7, 63.0, 49.9, 30.6, 22.0, 14.2, 11.1; HRMS (ESI) m/z calcd for $C_{29}H_{33}N_2O_7S_2^+$ $(M+H)^+$ 585.1724, found 585.1728. $[\alpha]^{20}_{D}$ = -156.5 (*c* 0.3, DCM). Chiral HPLC: Chiralpak AD-H, hexane:^{i}PrOH = 80:20, 1.0 mL/min, 400 nm; tR = 24.5 min (minor), 28.9 min (major).

Hammett Plot Studies

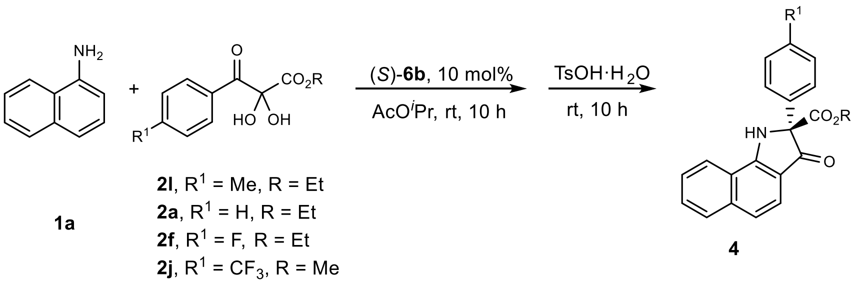

The 1-naphthylamine **1a** (0.05 mmol), 2,3-diketoester **2** (**2l**, **2a**, **2f**, **2j**) (0.055 mmol) and (*S*)-**6b** (10 mol %) were dissolved in AcO^{i}Pr (4 mL). The reaction mixture was stirred at room temperature for 10 h. Then the TsOH·H_2O (1.0 equiv) was added. The reaction mixture was stirred at room temperature for 10 h. The solvent was removed in vacuo and the crude products were separated by flash column chromatography on silica gel to afford the target products **4**.

Supplementary Table 2. Hammett plot studies.

	k_X	k_X/k_H	$\log(k_X/k_H)$	σ
Me	0.2	0.555556	-0.25527	-0.17
H	0.36	1	0	0
Cl	0.55	1.52777	0.184058	0.23
CF_3	0.73	2.027778	0.30702	0.54

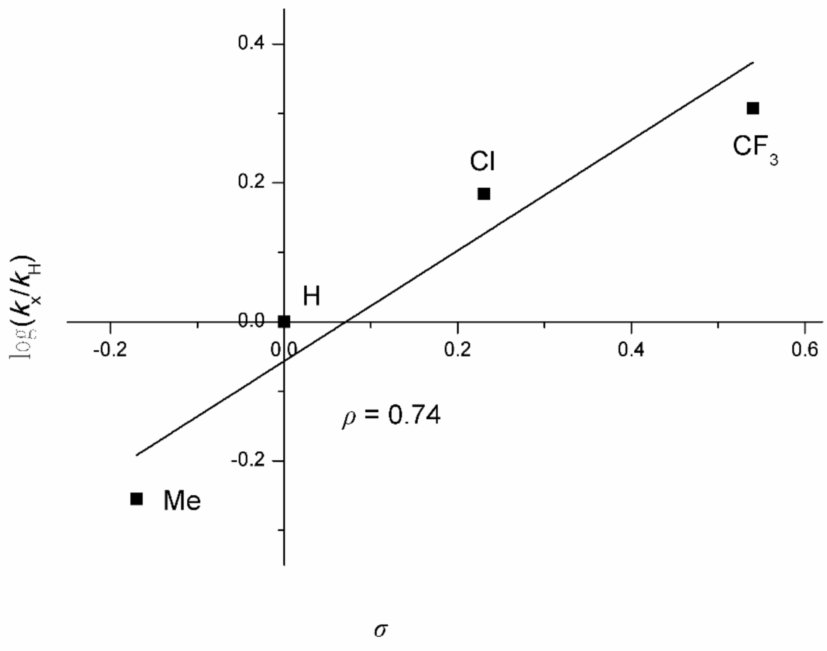

Supplementary Fig. 1. Hammett plot studies.

Control Experiments

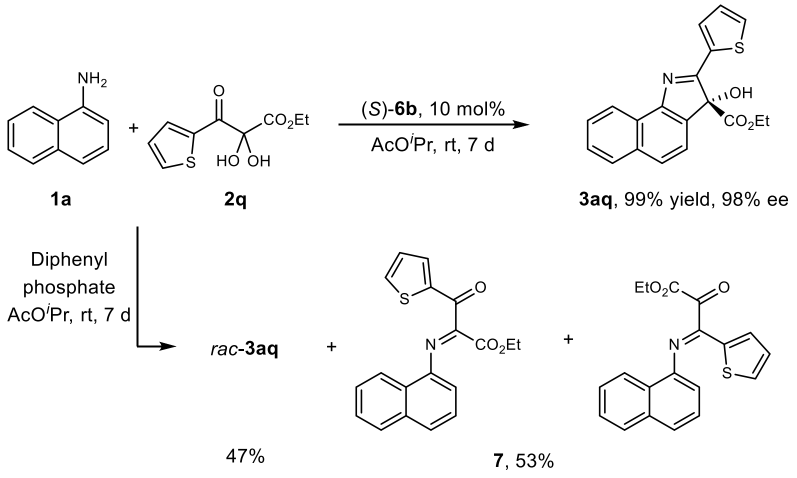

The 1-naphthylamine **1a** (0.05 mmol), **2q** (0.055 mmol) and (*S*)-**6b** (10 mol %) was dissolved in AcO^{i}Pr (4 mL). The reaction mixture was stirred at room temperature for 7 d. The solvent was removed in vacuo and the crude product was separated by flash column chromatography (DCM) on silica gel to afford compound **3aq** as yellow oil in 99% yield and 98% ee. ^{1}H NMR ($CDCl_3$) δ: 8.62 (d, J = 8.3 Hz, 1H), 7.88 (d, J = 8.2 Hz, 1H), 7.74 (d, J = 8.2 Hz, 1H), 7.68 (dd, J = 3.8, 0.9 Hz, 1H), 7.64-7.51 (m, 3H), 7.44 (d, J = 8.2 Hz, 1H), 7.15 (dd, J = 5.0, 3.8 Hz, 1H), 4.44 (br, s, 1H), 4.23-4.13 (m, 1H), 4.11-4.02 (m, 1H), 0.96 (t, J = 7.1 Hz, 3H); ^{13}C NMR ($CDCl_3$) δ: 172.0, 170.5, 151.8, 136.0, 135.3, 133.2, 131.0, 130.4, 128.3, 128.1, 127.0, 126.9, 126.9, 126.7, 124.0, 118.7, 86.7, 63.5, 13.7; HRMS (ESI) m/z calcd for $C_{19}H_{16}NO_3S^+$ $(M+H)^+$ 338.0845, found 338.0846. $[\alpha]^{20}_D$ = +93.7 (*c* 0.1, DCM). Chiral HPLC: Chiralpak AD-H, hexane:^{i}PrOH = 80:20, 1.0 mL/min, 400 nm; tR = 14.4 min (major), 15.6 min (minor).

The 1-naphthylamine **1a** (0.05 mmol), **2q** (0.055 mmol) and dipehyl phosphate (10 mol %) were dissolved in AcO^{i}Pr (4 mL). The reaction mixture was stirred at room temperature for 7 d. The solvent was removed in vacuo and the crude product was separated by flash column chromatography on silica gel to afford ketimine **7** and compound *rac*-**3aq** (47% yield). Product **7** was obtained by flash chromatography (PE:EA = 6:1) in 53% yield as yellow oil. ^{1}H NMR ($CDCl_3$) δ: 8.39 (dd, J = 3.9, 1.2 Hz, 1H), 8.08-8.05 (m, 1H), 7.89-7.85 (m, 1H), 7.82 (dd, J = 4.9, 1.2 Hz, 1H), 7.77 (d, J = 8.2 Hz, 1H), 7.60-7.50 (m, 2H), 7.42 (t, J = 7.5 Hz, 1H), 7.24-7.21 (m, 1H), 7.04 (dd, J = 7.3, 0.7 Hz, 1H), 4.17 (q, J = 7.1 Hz, 2H), 1.00 (t, J = 7.1 Hz, 3H); ^{13}C NMR ($CDCl_3$) δ: 179.5, 163.0, 157.4, 143.8, 137.7, 137.7, 137.0, 133.9, 127.9, 127.9, 127.6, 127.1, 126.9, 126.6, 125.3, 123.9, 113.5, 62.0, 13.8. HRMS (ESI) m/z calcd for $C_{19}H_{16}NO_3S^+$ $(M+H)^+$ 338.0845, found 338.0846.

Gram-scale Synthesis and Further Chemical Transformations

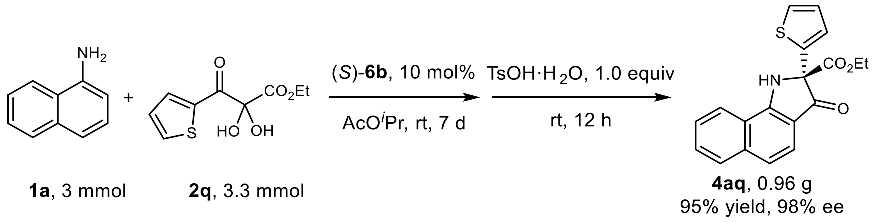

The 1-naphthylamine **1a** (2 mmol), **2q** (3.3 mmol) and (*S*)-**6b** (10 mol %) were dissolved in AcO^{i}Pr

(240 mL). The reaction mixture was stirred at room temperature for 7 d. Then the TsOH·H_2O (1.0 equiv) was added. The reaction mixture was stirred at room temperature for 10 h. The solvent was removed in vacuo and the crude product was separated by flash column chromatography on silica gel (PE:EA = 3:1) to afford product **4aq** in 95% yield and 98% ee.

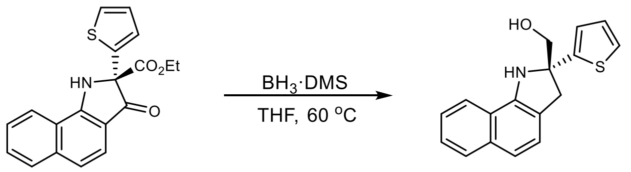

A solution of **4aq** (0.5 mmol) in THF (2 mL) was treated with borane-dimethyl sulfide complex (2 M in THF, 1.3 equiv) at 0 °C under N_2. The resulting mixture was heated at 60 °C for 4 h and then cooled to 0 °C. The reaction was then quenched with MeOH (0.5 mL) at 0 °C. The solvent was removed under reduced pressure, and the crude product was purified by silica gel column chromatography (PE:EA = 3:1) to afford **8** in 51% yield. ^{1}H NMR ($CDCl_3$) δ: 7.82-7.77 (m, 1H), 7.73-7.65 (m, 1H), 7.45-7.37 (m, 2H), 7.34 (d, *J* = 8.2 Hz, 1H), 7.27-7.23 (m, 1H), 7.19 (dd, *J* = 5.0, 1.0 Hz, 1H), 7.02 (dd, *J* = 3.5, 1.1 Hz, 1H), 6.96 (dd, *J* = 5.1, 3.6 Hz, 1H), 4.00 (d, *J* = 11.1 Hz, 1H), 3.86 (d, *J* = 11.1 Hz, 1H), 3.53 (d, *J* = 15.9 Hz, 1H), 3.41 (d, *J* = 15.8 Hz, 1H); ^{13}C NMR ($CDCl_3$) δ: 149.8, 144.3, 133.6, 128.6, 127.3, 125.3, 125.2, 124.6, 123.3, 123.0, 121.5, 121.1, 120.1, 69.2, 68.4, 42.5; HRMS (ESI) m/z calcd for $C_{17}H_{16}NOS^+$ $(M+H)^+$ 282.0947, found 282.0947. $[\alpha]^{20}_D$ = -140.8 (*c* 0.1, DCM). Chiral HPLC: Chiralpak AD-H, hexane:^{i}PrOH = 80:20, 1.0 mL/min, 254 nm; tR = 9.9 min (major), 11.9 min (minor).

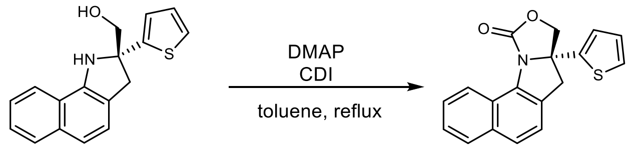

Compound **8** (0.1 mmol) was dissolved in dry toluene (2 mL) under N_2, treated with DMAP (1 mol%) and CDI (2.0 equiv) and refluxed for 3 h. After cooling to room temperture, the mixture was purified by silica gel column chromatography (PE:EA = 3:1) to afford compound **9** in 86% yield and 92% ee as white solid. M.p.: 188-190 °C. ^{1}H NMR ($CDCl_3$) δ: 8.50 (d, *J* = 8.5 Hz, 1H), 7.82 (d, *J* = 8.3 Hz, 1H), 7.68 (d, *J* = 8.3 Hz, 1H), 7.61-7.55 (m, 1H), 7.51-7.44 (m, 1H), 7.32 (d, *J* = 8.3 Hz, 1H), 7.17 (dd, *J* = 5.1, 1.1 Hz, 1H), 7.09 (dd, *J* = 3.6, 1.1 Hz, 1H), 6.91 (dd, *J* = 5.0, 3.7 Hz, 1H), 4.81 (d, *J* = 8.9 Hz, 1H), 4.73 (d, *J* = 8.9 Hz, 1H), 3.90 (d, *J* = 15.4 Hz, 1H), 3.63 (d, *J* = 15.5 Hz, 1H); ^{13}C NMR ($CDCl_3$) δ: 157.1, 148.1, 136.8, 134.1, 128.3, 128.0, 127.3, 127.1, 126.7, 126.2, 125.1, 125.0, 125.0, 123.7, 122.2, 76.1, 73.0, 45.4; HRMS (ESI) m/z calcd for $C_{18}H_{14}NO_2S^+$ $(M+H)^+$ 308.0740, found 308.0741. $[\alpha]^{20}_D$ = +57.2 (*c* 0.06, DCM). Chiral HPLC: Chiralpak AD-H, hexane:^{i}PrOH = 80:20, 1.0 mL/min, 254 nm; tR = 11.9 min (minor), 14.5 min (major).

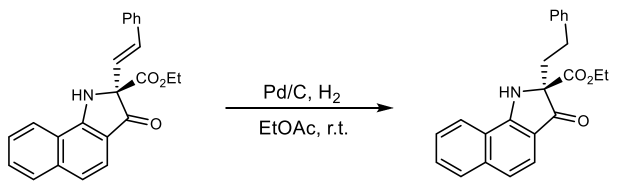

4ar, 90% ee, 0.05 mmol **10**, 86% yield, 94% ee

To a solution of **4ar** (0.05 mmol) in EtOAc was added palladium on activated charcoal (10% Pd). The reaction mixture was stirred at room temperature under H_2 atmosphere. After completion of the reaction, the reaction mixture was filtered through Celite and concentrated. The residue was purified by column chromatography on silica gel (PE:EA = 3:1) to give the compound **10** as colorless oil in 86% yield with 94% ee. ^{1}H NMR ($CDCl_3$) δ: 7.94 (d, J = 8.2 Hz, 1H), 7.83 (d, J = 8.1 Hz, 1H), 7.69-7.63 (m, 1H), 7.58-7.50 (m, 2H), 7.32-7.23 (m, 3H), 7.22-7.14 (m, 3H), 5.87 (br, s, 1H), 4.34-4.15 (m, 2H), 2.80-2.64 (m, 2H), 2.64-2.53 (m, 1H), 2.42-2.31 (m, 1H), 1.31 (t, J = 7.1 Hz, 3H); ^{13}C NMR ($CDCl_3$) δ: 194.3, 168.4, 162.7, 140.8, 138.3, 130.5, 129.0, 128.5, 126.3, 126.2, 122.2, 121.6, 121.1, 120.1, 115.0, 75.1, 62.8, 38.1, 30.7, 14.2; HRMS (ESI) m/z calcd for $C_{23}H_{22}NO_3^+$ $(M+H)^+$ 360.1594, found 360.1596. $[\alpha]^{20}_D$ = +276.7 (c 0.1, DCM). Chiral HPLC: Chiralpak AD-H, hexane:^{i}PrOH = 80:20, 1.0 mL/min, 400 nm; tR = 8.7 min (minor), 11.7 min (major).

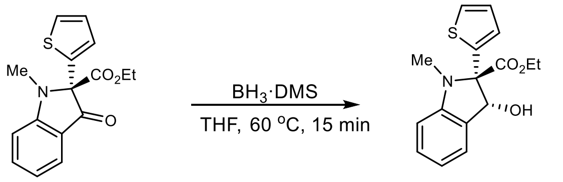

4qq, 0.05 mmol **11**, 54% yield, 8:1 dr, 90% ee

A solution of **4qq** (0.05 mmol) in THF (0.5 mL) was treated with borane-dimethyl sulfide complex (2 M in THF, 1.3 equiv) at 0 °C under N_2. The resulting mixture was heated at 60 °C for 15 min and then cooled to 0 °C. The reaction was then quenched with MeOH (0.5 mL) at 0 °C. The solvent was removed under reduced pressure, and the crude product was purified by silica gel column chromatography (PE:EA = 5:1) to afford **11** in 54% yield with 90% ee as colorless oil. ^{1}H NMR ($CDCl_3$) δ: 7.34-7.27 (m, 2H), 7.27-7.23 (m, 1H), 7.13 (dd, J = 3.6, 1.0 Hz, 1H), 7.04 (dd, J = 5.1, 3.7 Hz, 1H), 6.78 (t, J = 7.2 Hz, 1H), 6.56 (d, J = 7.9 Hz, 1H), 5.37 (d, J = 7.8 Hz, 1H), 4.35-4.21 (m, 2H), 2.89 (s, 3H), 1.77 (d, J = 7.9 Hz, 1H), 1.30 (t, J = 7.1 Hz, 3H); ^{13}C NMR ($CDCl_3$) δ: 171.9, 150.7, 136.7, 130.4, 127.8, 127.0, 126.8, 126.5, 125.4, 118.5, 106.9, 80.6, 78.1, 76.7, 62.0, 32.0, 14.1; HRMS (ESI) m/z calcd for $C_{16}H_{18}NO_3S^+$ $(M+H)^+$ 304.1002, found 304.1004. $[\alpha]^{20}_D$ = -140.0 (c 0.05, DCM). Chiral HPLC: Chiralpak AD-H, hexane:^{i}PrOH = 80:20, 1.0 mL/min, 254 nm; tR = 9.4 min (major), 15.0 min (minor).

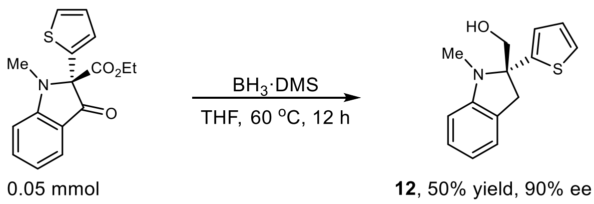

4qq, 0.05 mmol **12**, 50% yield, 90% ee

A solution of **4qq** (0.05 mmol) in THF (0.5 mL) was treated with borane-dimethyl sulfide complex (2 M in THF, 2.3 equiv) at 0 °C under N_2. The resulting mixture was heated at 60 °C for 12 h and

then cooled to 0 °C. The reaction was then quenched with MeOH (0.5 mL) at 0 °C. The solvent was removed under reduced pressure, and the crude product was purified by silica gel column chromatography (PE:EA = 5:1) to afford **12** in 50% yield as colorless oil. ^{1}H NMR ($CDCl_3$) *δ*: 7.18-7.07 (m, 3H), 6.92 (dd, *J* = 5.0, 3.6 Hz, 1H), 6.86 (dd, *J* = 3.6, 1.1 Hz, 1H), 6.78-6.72 (m, 1H), 6.46 (d, *J* = 7.8 Hz, 1H), 4.17 (d, *J* = 11.2 Hz, 1H), 3.95 (t, *J* = 10.6 Hz, 1H), 3.65 (d, *J* = 16.0 Hz, 1H), 3.18 (d, *J* = 16.0 Hz, 1H), 2.60 (s, 3H), 1.99 (d, *J* = 9.6 Hz, 1H); ^{13}C NMR ($CDCl_3$) *δ*: 151.6, 145.0, 127.8, 127.5, 126.4, 124.3, 124.1, 123.4, 118.6, 107.2, 72.2, 63.4, 40.9, 29.8; HRMS (ESI) m/z calcd for $C_{14}H_{15}NOSNa^+$ $(M+Na)^+$ 268.0767, found 268.0766. $[\alpha]^{20}_{D}$ = +65.8 (*c* 0.04, DCM). Chiral HPLC: Chiralpak AD-H, hexane:^{i}PrOH = 80:20, 1.0 mL/min, 254 nm; tR = 7.9 min (major), 8.8 min (minor).

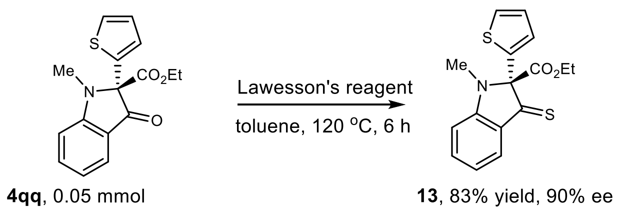

A solution of the compound **4qq** (0.05 mmol) and Lawesson's reagent (0.06 mmol) in toluene (1.0 mL) was refluxed under N_2 for 6 h. Then the mixture was concentrated in vacuo, and the residue was purified via column chromatography on silica gel (PE:EA = 5:1) to afford **13** in 83% yield with 90% ee as red oil. ^{1}H NMR ($CDCl_3$) *δ*: 7.73-7.68 (m, 1H), 7.60-7.53 (m, 1H), 7.31-7.23 (m, 2H), 6.99 (dd, *J* = 5.1, 3.7 Hz, 1H), 6.87 (d, *J* = 8.4 Hz, 1H), 6.81-6.75 (m, 1H), 4.33-4.15 (m, 2H), 3.10 (s, 3H), 1.22 (t, J = 7.1 Hz, 3H); ^{13}C NMR ($CDCl_3$) *δ*: 222.7, 165.0, 160.3, 138.7, 137.4, 132.3, 127.0, 126.8, 126.6, 126.2, 119.6, 109.1, 88.2, 77.4, 63.0, 30.9, 14.0; HRMS (ESI) m/z calcd for $C_{16}H_{15}NO_2S_2Na^+$ $(M+Na)^+$ 340.0436, found 340.0437. Chiral HPLC: Chiralpak AD-H, hexane:^{i}PrOH = 80:20, 1.0 mL/min, 520 nm; tR = 5.4 min (minor), 5.8 min (major).

Crystallographic Data for 4bq (CCDC 2262643)

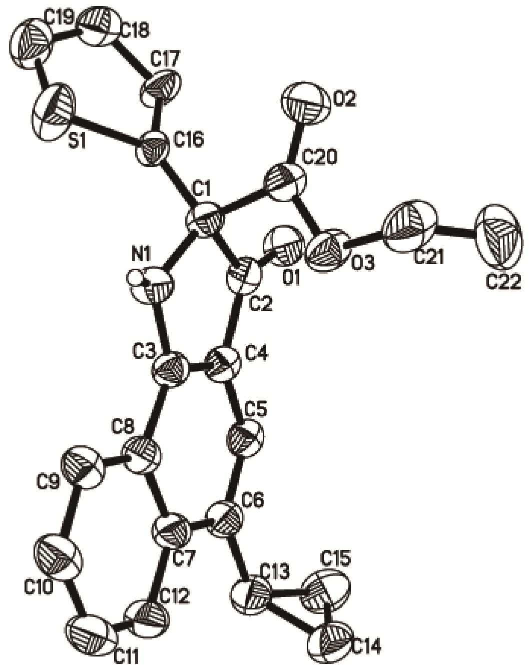

Supplementary Table 3. Crystal data and structure refinement for 4bq (CCDC 2262643).

Identification code	202305102_auto
Empirical formula	$C_{22}H_{19}NO_3S$
Formula weight	377.44
Temperature/K	293(2)
Crystal system	orthorhombic
Space group	$P2_12_12_1$
a/Å	8.90719(12)
b/Å	11.48656(18)
c/Å	19.0578(3)
α/°	90
β/°	90
γ/°	90
Volume/Å^3	1949.87(5)
Z	4
ρ_{calc}g/cm^3	1.286
μ/mm^{-1}	1.650
F(000)	792.0
Crystal size/mm^3	0.18 × 0.12 × 0.1
Radiation	CuKα (λ = 1.54184)
2Θ range for data collection/°	8.988 to 140.714
Index ranges	$-10 \leq h \leq 9$, $-13 \leq k \leq 13$, $-20 \leq l \leq 23$
Reflections collected	6641
Independent reflections	3649 [R_{int} = 0.0351, R_{sigma} = 0.0528]
Data/restraints/parameters	3649/41/279
Goodness-of-fit on F^2	1.034
Final R indexes [I>=2σ (I)]	R_1 = 0.0628, wR_2 = 0.1619
Final R indexes [all data]	R_1 = 0.0759, wR_2 = 0.1787
Largest diff. peak/hole / e Å^{-3}	0.22/-0.22
Flack parameter	-0.06(4)

Supplementary Table 4. Fractional Atomic Coordinates (×10[4]) and Equivalent Isotropic Displacement Parameters (Å[2]×10[3]) for 4bq (CCDC 2262643). U_{eq} is defined as 1/3 of of the trace of the orthogonalised U_{IJ} tensor.

Atom	*x*	*y*	*z*	U(eq)
S1	1857(4)	4477(5)	2810(3)	115.8(15)
S1A	1412(11)	2137(8)	2446(8)	115.8(15)
O1	4303(5)	896(3)	2721(2)	84.7(10)
O2	4075(7)	3125(17)	1336(4)	118(3)
O2A	3770(40)	3080(100)	1440(20)	118(3)
O3	6319(5)	3145(7)	1833(3)	83.9(17)
O3A	6080(30)	2520(30)	1659(16)	83.9(17)
N1	5075(5)	3859(3)	3043(2)	67.3(9)
C1	4279(5)	3016(4)	2605(2)	63.3(10)
C2	4792(5)	1821(4)	2917(2)	65.1(10)
C3	6075(5)	3285(4)	3464(2)	62.6(10)
C4	5921(5)	2080(4)	3427(2)	62.3(10)
C5	6858(6)	1339(4)	3825(2)	68.6(11)
C6	7971(6)	1781(5)	4237(2)	73.1(12)
C7	8155(5)	3037(5)	4272(2)	72.7(12)
C8	7182(5)	3801(4)	3898(2)	66.5(10)
C9	7380(6)	5026(5)	3943(3)	74.4(12)
C10	8502(7)	5467(6)	4338(3)	89.2(16)
C11	9480(8)	4739(7)	4701(3)	97.1(19)
C12	9308(7)	3553(6)	4669(3)	88.4(16)
C13	9032(8)	1027(6)	4630(3)	95.5(18)
C14	10497(9)	707(8)	4319(4)	110(2)
C15	9345(10)	-184(8)	4405(5)	122(3)
C16	2615(14)	3073(12)	2655(18)	57(3)
C16A	2560(20)	3360(20)	2620(30)	57(3)
C17	1340(15)	2401(13)	2500(11)	81(4)
C17A	1596(19)	4300(20)	2683(15)	81(4)
C18	-15(12)	2958(12)	2522(7)	91(3)
C18A	80(20)	3990(20)	2586(12)	91(3)
C19	191(18)	4038(14)	2801(7)	111(5)
C19A	-130(30)	2820(20)	2680(15)	111(5)
C20	4826(5)	3066(5)	1845(3)	71.8(11)
C21	7021(11)	3259(11)	1158(5)	114(4)
C21A	6480(50)	2680(60)	900(30)	114(4)
C22	7591(19)	2205(12)	858(5)	148(5)
C22A	7580(100)	1690(60)	1050(30)	148(5)

Supplementary Table 5. Anisotropic Displacement Parameters ($Å^2×10^3$) for 4bq (CCDC 2262643). The Anisotropic displacement factor exponent takes the form: $-2π^2[h^2a^{*2}U_{11}+2hka^*b^*U_{12}+…]$.

Atom	U_{11}	U_{22}	U_{33}	U_{23}	U_{13}	U_{12}
S1	80.0(19)	99(2)	168(4)	-34(2)	25(2)	-1.7(15)
S1A	80.0(19)	99(2)	168(4)	-34(2)	25(2)	-1.7(15)
O1	88(2)	60.7(17)	105(2)	-10.7(17)	-11(2)	-1.1(17)
O2	69(3)	215(7)	70(3)	-2(4)	4(3)	3(6)
O2A	69(3)	215(7)	70(3)	-2(4)	4(3)	3(6)
O3	58(2)	104(4)	89(3)	17(3)	7(2)	4(2)
O3A	58(2)	104(4)	89(3)	17(3)	7(2)	4(2)
N1	65(2)	56(2)	81(2)	5.2(17)	-11.3(19)	-3.3(16)
C1	55(2)	64(2)	71(2)	0.0(19)	-3.8(18)	1.3(18)
C2	61(2)	61(2)	73(2)	-3(2)	6.1(19)	2.6(19)
C3	54(2)	71(2)	63(2)	2.4(19)	1.1(18)	-0.5(19)
C4	59(2)	64(2)	64(2)	6.7(18)	3.6(18)	1.1(19)
C5	67(3)	70(2)	68(2)	9(2)	8(2)	5(2)
C6	69(3)	91(3)	59(2)	7(2)	1(2)	11(3)
C7	62(2)	101(4)	56(2)	0(2)	-0.4(18)	2(3)
C8	59(2)	80(3)	60(2)	-1(2)	3.1(19)	-7(2)
C9	77(3)	77(3)	68(2)	-3(2)	0(2)	-11(2)
C10	91(4)	100(4)	77(3)	-13(3)	-1(3)	-21(3)
C11	87(4)	126(5)	79(3)	-10(3)	-15(3)	-22(4)
C12	74(3)	123(5)	68(3)	-5(3)	-10(2)	3(3)
C13	94(4)	124(5)	69(3)	9(3)	-8(3)	31(4)
C14	91(4)	140(6)	100(4)	9(4)	-13(4)	33(4)
C15	121(6)	115(5)	130(6)	21(5)	-25(5)	38(5)
C16	63(2)	46(8)	63(4)	2(9)	1(2)	10(3)
C16A	63(2)	46(8)	63(4)	2(9)	1(2)	10(3)
C17	40(4)	79(7)	122(8)	-14(6)	-9(4)	2(4)
C17A	40(4)	79(7)	122(8)	-14(6)	-9(4)	2(4)
C18	55(4)	135(7)	82(6)	12(5)	9(3)	-12(5)
C18A	55(4)	135(7)	82(6)	12(5)	9(3)	-12(5)
C19	108(7)	130(7)	97(8)	7(6)	25(5)	28(6)
C19A	108(7)	130(7)	97(8)	7(6)	25(5)	28(6)
C20	65(2)	76(3)	74(3)	8(2)	-1(2)	4(2)
C21	75(5)	159(9)	109(6)	40(6)	24(4)	4(5)
C21A	75(5)	159(9)	109(6)	40(6)	24(4)	4(5)
C22	197(11)	159(12)	87(7)	9(6)	34(7)	42(11)

C22A	197(11)	159(12)	87(7)	9(6)	34(7)	42(11)

Supplementary Table 6. Bond Lengths for 4bq (CCDC 2262643).

Atom	Atom	Length/Å	Atom	Atom	Length/Å
S1	C16	1.773(13)	C5	C6	1.363(7)
S1	C19	1.566(16)	C6	C7	1.453(9)
S1A	C16A	1.76(2)	C6	C13	1.485(7)
S1A	C19A	1.64(2)	C7	C8	1.425(7)
O1	C2	1.208(6)	C7	C12	1.407(8)
O2	C20	1.181(9)	C8	C9	1.420(8)
O2A	C20	1.22(3)	C9	C10	1.349(8)
O3	C20	1.333(7)	C10	C11	1.391(10)
O3	C21	1.437(10)	C11	C12	1.372(10)
O3A	C20	1.33(2)	C13	C14	1.480(10)
O3A	C21A	1.49(6)	C13	C15	1.482(11)
N1	C1	1.462(6)	C14	C15	1.459(12)
N1	C3	1.368(6)	C16	C17	1.404(16)
C1	C2	1.563(6)	C16A	C17A	1.39(2)
C1	C16	1.487(14)	C17	C18	1.367(15)
C1	C16A	1.58(2)	C17A	C18A	1.408(19)
C1	C20	1.530(7)	C18	C19	1.363(17)
C2	C4	1.430(7)	C18A	C19A	1.37(2)
C3	C4	1.393(7)	C21	C22	1.431(14)
C3	C8	1.417(6)	C21A	C22A	1.53(3)
C4	C5	1.412(6)			

Supplementary Table 7. Bond Angles for 4bq (CCDC 2262643).

Atom	Atom	Atom	Angle/°	Atom	Atom	Atom	Angle/°
C19	S1	C16	93.8(7)	C3	C8	C9	122.4(5)
C19A	S1A	C16A	93.0(14)	C9	C8	C7	120.3(5)
C20	O3	C21	117.2(6)	C10	C9	C8	119.9(6)
C20	O3A	C21A	113(3)	C9	C10	C11	121.0(6)
C3	N1	C1	109.3(4)	C12	C11	C10	120.3(6)
N1	C1	C2	102.9(3)	C11	C12	C7	121.6(6)
N1	C1	C16	114.7(10)	C14	C13	C6	120.3(5)
N1	C1	C16A	107.2(14)	C14	C13	C15	59.0(5)
N1	C1	C20	111.2(4)	C15	C13	C6	121.4(6)

C2	C1	C16A	119.6(14)	C15	C14	C13	60.6(6)
C16	C1	C2	107.9(8)	C14	C15	C13	60.4(6)
C16	C1	C20	112.1(14)	C1	C16	S1	115.5(9)
C20	C1	C2	107.4(4)	C17	C16	S1	103.1(9)
C20	C1	C16A	108(2)	C17	C16	C1	140.2(15)
O1	C2	C1	123.3(4)	C1	C16A	S1A	111.1(15)
O1	C2	C4	130.3(5)	C17A	C16A	S1A	106.4(14)
C4	C2	C1	106.3(4)	C17A	C16A	C1	142.4(18)
N1	C3	C4	112.6(4)	C18	C17	C16	116.8(13)
N1	C3	C8	126.4(4)	C16A	C17A	C18A	112(2)
C4	C3	C8	120.9(4)	C19	C18	C17	108.6(13)
C3	C4	C2	108.1(4)	C19A	C18A	C17A	111(2)
C3	C4	C5	120.8(4)	C18	C19	S1	115.1(11)
C5	C4	C2	131.0(5)	C18A	C19A	S1A	109(2)
C6	C5	C4	121.0(5)	O2	C20	O3	123.1(6)
C5	C6	C7	118.6(4)	O2	C20	C1	126.9(5)
C5	C6	C13	122.5(6)	O2A	C20	O3A	119(3)
C7	C6	C13	118.9(5)	O2A	C20	C1	111(3)
C8	C7	C6	121.3(4)	O3	C20	C1	109.7(4)
C12	C7	C6	121.8(5)	O3A	C20	C1	120.2(13)
C12	C7	C8	116.9(5)	C22	C21	O3	115.8(9)
C3	C8	C7	117.2(4)	O3A	C21A	C22A	83(4)

Supplementary Table 8. Hydrogen Bonds for 4bq (CCDC 2262643).

D	H	A	d(D-H)/Å	d(H-A)/Å	d(D-A)/Å	D-H-A/°
N1	H1	O1[1]	0.86(3)	1.99(3)	2.811(5)	160(5)

[1]1-X,1/2+Y,1/2-Z

Supplementary Table 9. Hydrogen Atom Coordinates (Å×10^4) and Isotropic Displacement Parameters (Å^2×10^3) for 4bq (CCDC 2262643).

Atom	*x*	*y*	*z*	U(eq)
H1	5240(60)	4560(30)	2910(30)	79(16)
H5	6712	538	3806	82
H9	6738	5522	3701	89
H10	8623	6270	4368	107
H11	10254	5058	4966	116

H12	9970	3080	4916	106
H13	9046	1152	5139	115
H14A	10711	975	3847	133
H14B	11361	677	4629	133
H15A	9495	-765	4768	147
H15B	8845	-466	3986	147
H17	1415	1615	2388	97
H17A	1917	5055	2780	97
H18	-926	2651	2371	109
H18A	-680	4515	2472	109
H19	-599	4483	2975	134
H19A	-996	2468	2848	134
H21A	6297	3590	834	137
H21B	7844	3807	1199	137
H21C	6944	3423	792	137
H21D	5682	2490	576	137
H22A	8197	2387	456	221
H22B	6768	1718	717	221
H22C	8189	1802	1199	221
H22D	8527	2007	1204	221
H22E	7736	1243	631	221
H22F	7184	1194	1412	221

Supplementary Table 10. Atomic Occupancy for 4bq (CCDC 2262643).

**Atom**	***Occupancy***	**Atom**	***Occupancy***	**Atom**	***Occupancy***
S1	0.606(6)	S1A	0.394(6)	O2	0.836(12)
O2A	0.164(12)	O3	0.836(12)	O3A	0.164(12)
C16	0.606(6)	C16A	0.394(6)	C17	0.606(6)
H17	0.606(6)	C17A	0.394(6)	H17A	0.394(6)
C18	0.606(6)	H18	0.606(6)	C18A	0.394(6)
H18A	0.394(6)	C19	0.606(6)	H19	0.606(6)
C19A	0.394(6)	H19A	0.394(6)	C21	0.836(12)
H21A	0.836(12)	H21B	0.836(12)	C21A	0.164(12)
H21C	0.164(12)	H21D	0.164(12)	C22	0.836(12)
H22A	0.836(12)	H22B	0.836(12)	H22C	0.836(12)
C22A	0.164(12)	H22D	0.164(12)	H22E	0.164(12)
H22F	0.164(12)				

DFT Calculations

1. Computational Methods

All density functional theory (DFT) calculations were performed using the Gaussian 16 program[7]. The geometry optimizations were conducted using the M06-2X functional and 6-31G(d) basis set in diethylether solvent (**4aq** was obtained in 71% yield with 96% ee). To confirm whether each optimized stationary point was an energy minimum or a transition state, as well as to evaluate the zero-point vibrational energy and the thermal corrections at 298 K, the vibrational frequencies were computed at the same level of theory as for the geometry optimizations. Based on the optimized structures, the M06-2X functional and 6-311+G(d,p) basis set was used to calculate the solvation single-point energies to give more accurate energy information. The solvent effects of geometry optimizations and single-point calculations were considered by the SMD solvation model in diethylether solvent.

2. Absolute Calculation Energies, Enthalpies, and Free Energies.

Geometry	$E_{(elec\text{-}M06\text{-}2X)}$[a]	$G_{(corr\text{-}M06\text{-}2X)}$[b]	$H_{(corr\text{-}M06\text{-}2X)}$[c]	$E_{(M06\text{-}2X)}$[d]	IF[e]
**CPA**	-2375.037826	0.577822	0.683426	-2375.605397	-
**1a**	-441.060716	0.133725	0.175461	-441.186036	-
**2q**	-1046.542578	0.116554	0.174756	-1046.773852	-
**Int4**	-2816.117708	0.735116	0.86108	-2816.810628	-
**Int5**	-3862.694822	0.88027	1.037144	-3863.614291	-
**TS6-*RS***	-3862.684333	0.881836	1.033759	-3863.604215	376.24*i*
**TS6-*RR***	-3862.683743	0.884094	1.034105	-3863.602008	361.56*i*
**TS6-*SR***	-3862.674244	0.878543	1.032587	-3863.594446	741.60*i*
**TS6-*SS***	-3862.673311	0.882825	1.03395	-3863.593354	466.61*i*
**Int7-*RS***	-3862.70194	0.885212	1.037929	-3863.621798	-
**TS8-*R***	-3862.68466	0.879607	1.033134	-3863.605633	1088.23*i*
**Int9-*R***	-3862.698391	0.883188	1.038665	-3863.62253	-

**Int10-*R***	-3862.709467	0.883384	1.038491	-3863.634042	-
**TS11-*R***	-3862.697669	0.885106	1.036621	-3863.621668	94.64*i*
**Int12-*R***	-3862.714793	0.890849	1.040397	-3863.637073	-
**TS13-*R***	-3862.711053	0.884389	1.035066	-3863.635984	840.37*i*
**Int14-*R***	-1487.649012	0.280508	0.353432	-1488.007588	-
**TsOH**	-895.125319	0.106874	0.155705	-895.309819	-
**3aq**	-1411.233578	0.251929	0.323948	-1411.555543	-
**Int15**	-2306.391151	0.38319	0.481595	-2306.89727	-
**TS16**	-2306.386426	0.38192	0.477804	-2306.892497	613.24*i*
**Int17**	-2306.348238	0.388966	0.482348	-2306.896021	-
**TS18**	-1792.342183	0.382199	0.47803	-2306.853645	472.29*i*
**4aq**	-1411.253284	0.253351	0.324528	-1411.572603	-

[a]The electronic energy calculated by M06-2X in diethylether solvent. [b]The thermal correction to Gibbs free energy calculated by M06-2X in diethylether solvent. [c]The thermal correction to enthalpy calculated by M06-2X in diethylether solvent. [d]The electronic energy calculated by M06-2X in diethylether solvent. [e]The M06-2X calculated imaginary frequencies for the transition states.

Copies of NMR Spectra

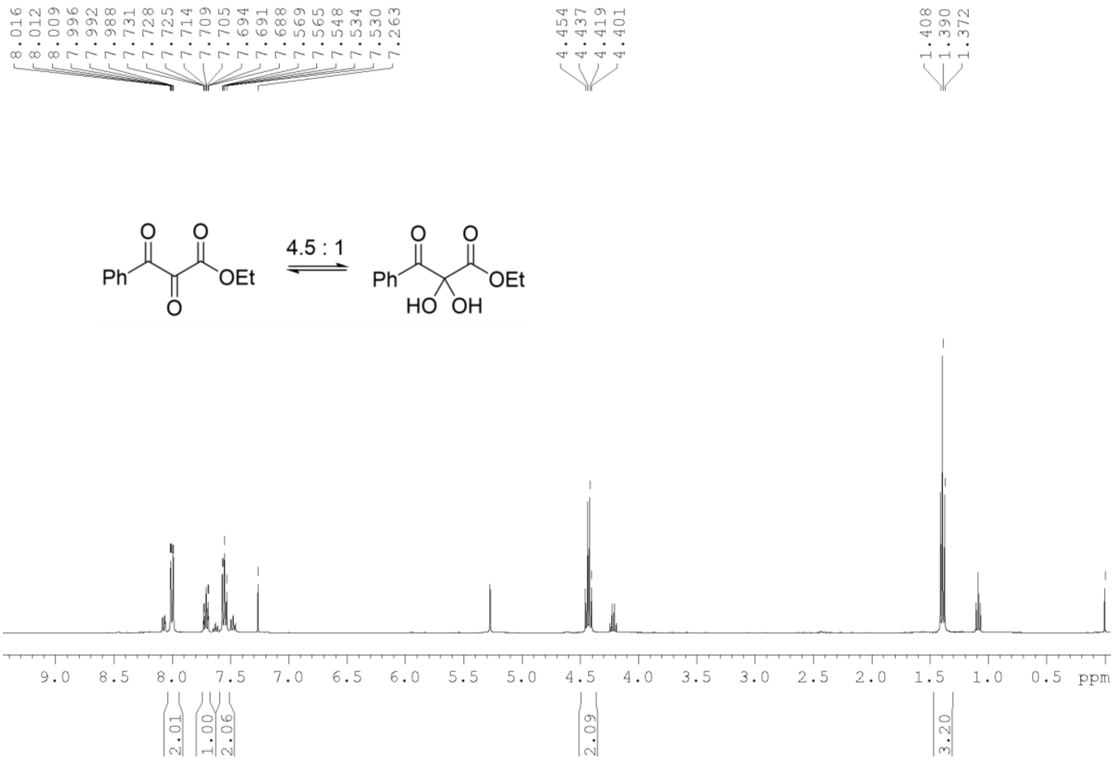

^{1}H NMR (400 MHz, $CDCl_3$) of **2a**

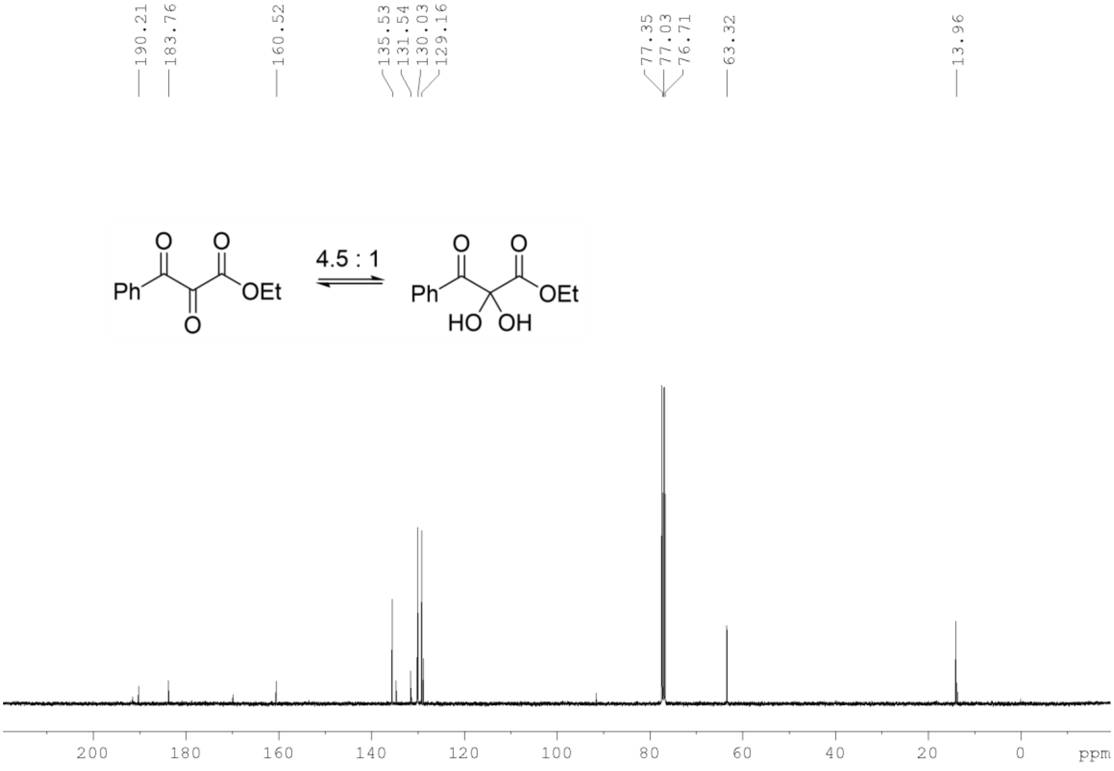

^{13}C NMR (100 MHz, $CDCl_3$) of **2a**

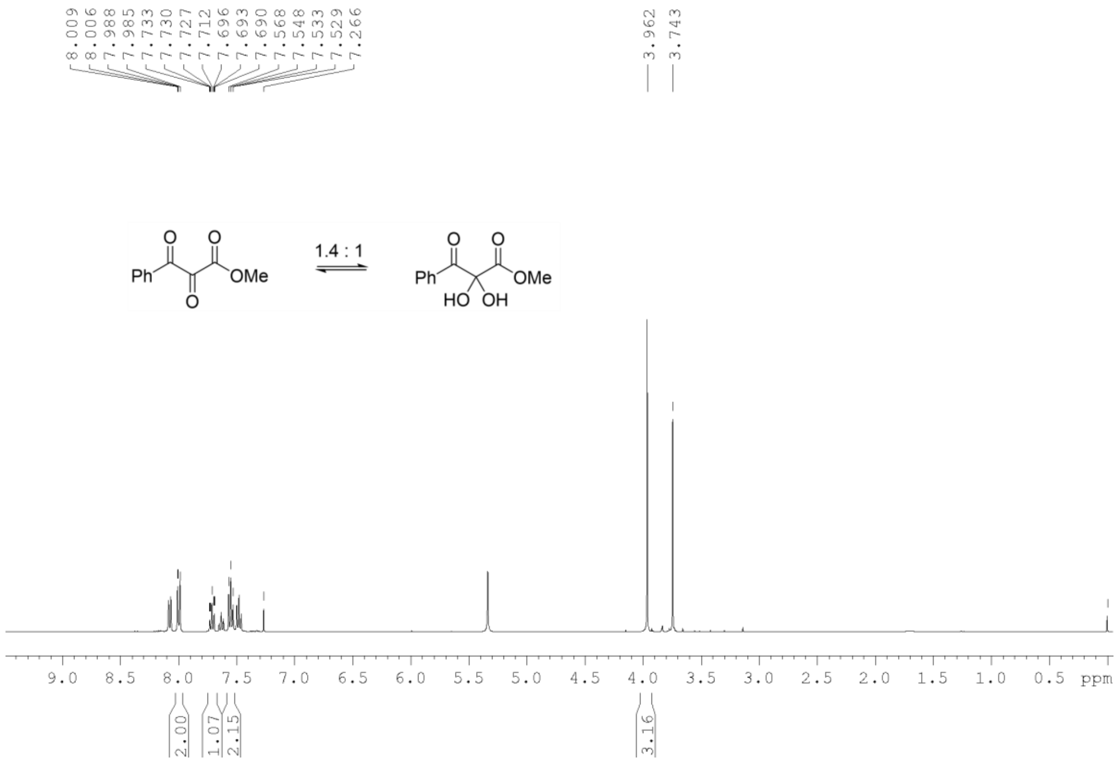

^{1}H NMR (400 MHz, $CDCl_3$) of **2b**

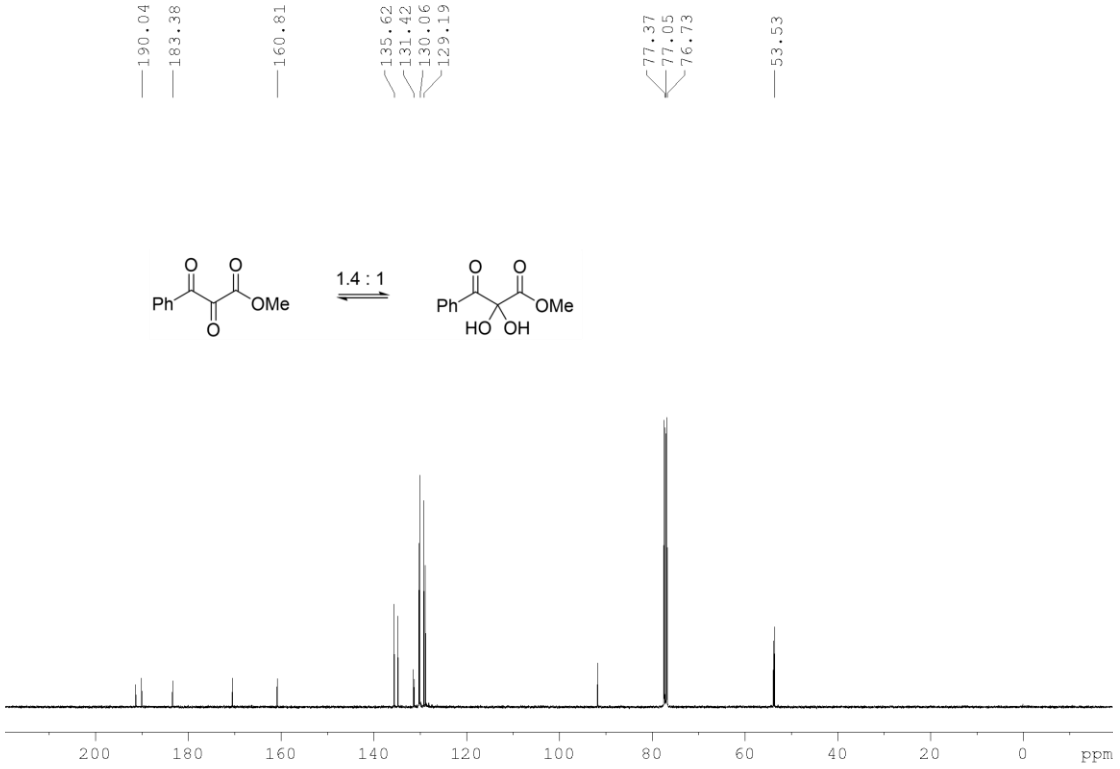

^{13}C NMR (100 MHz, $CDCl_3$) of **2b**

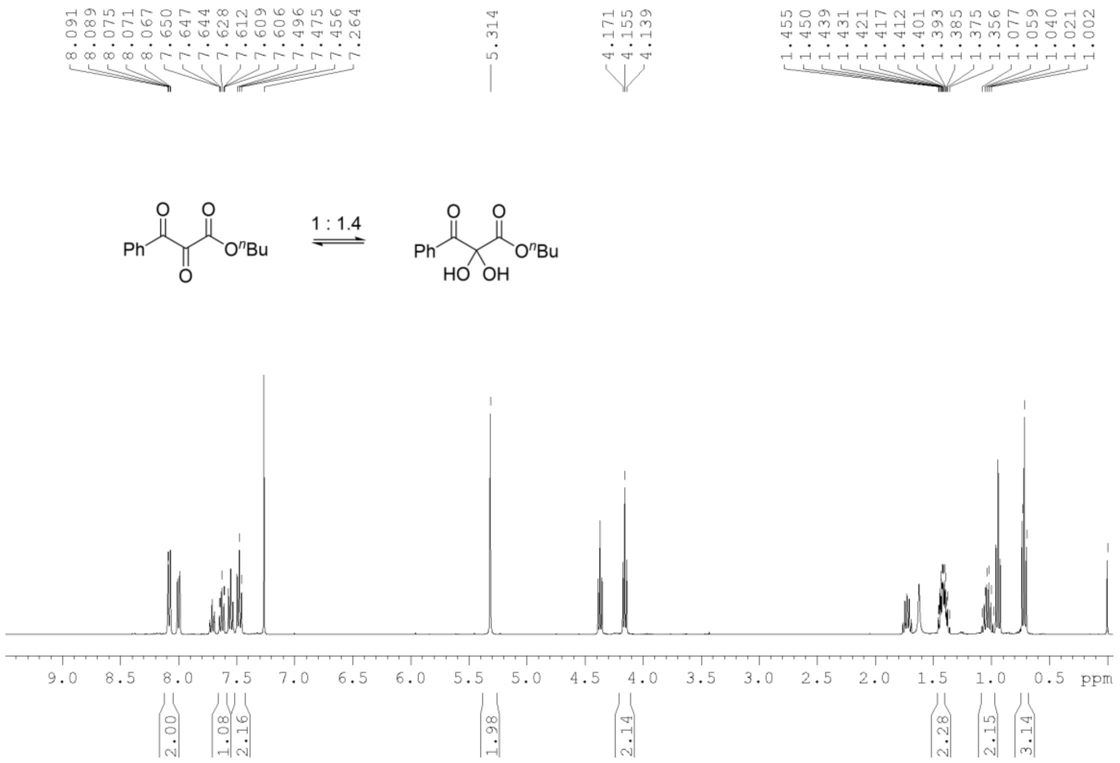

^{1}H NMR (400 MHz, CDCl3) of **2c**

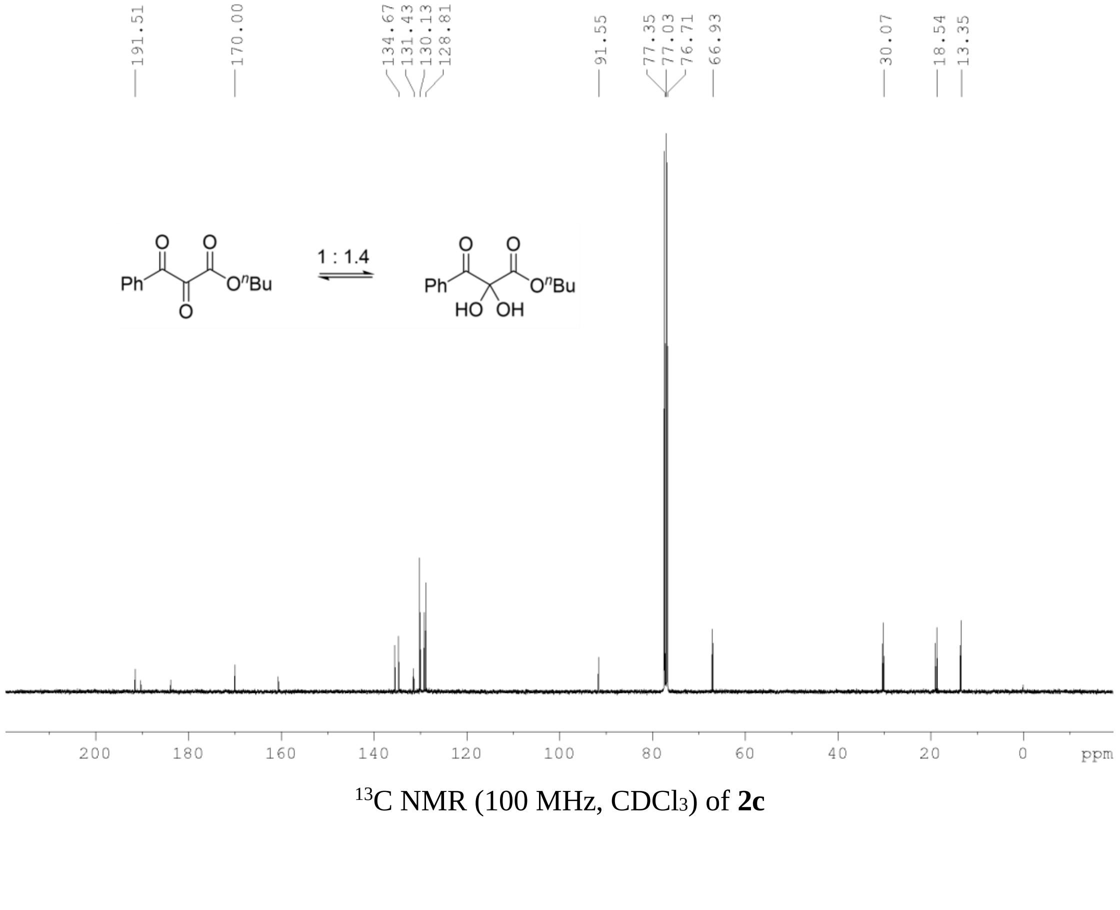

^{13}C NMR (100 MHz, CDCl3) of **2c**

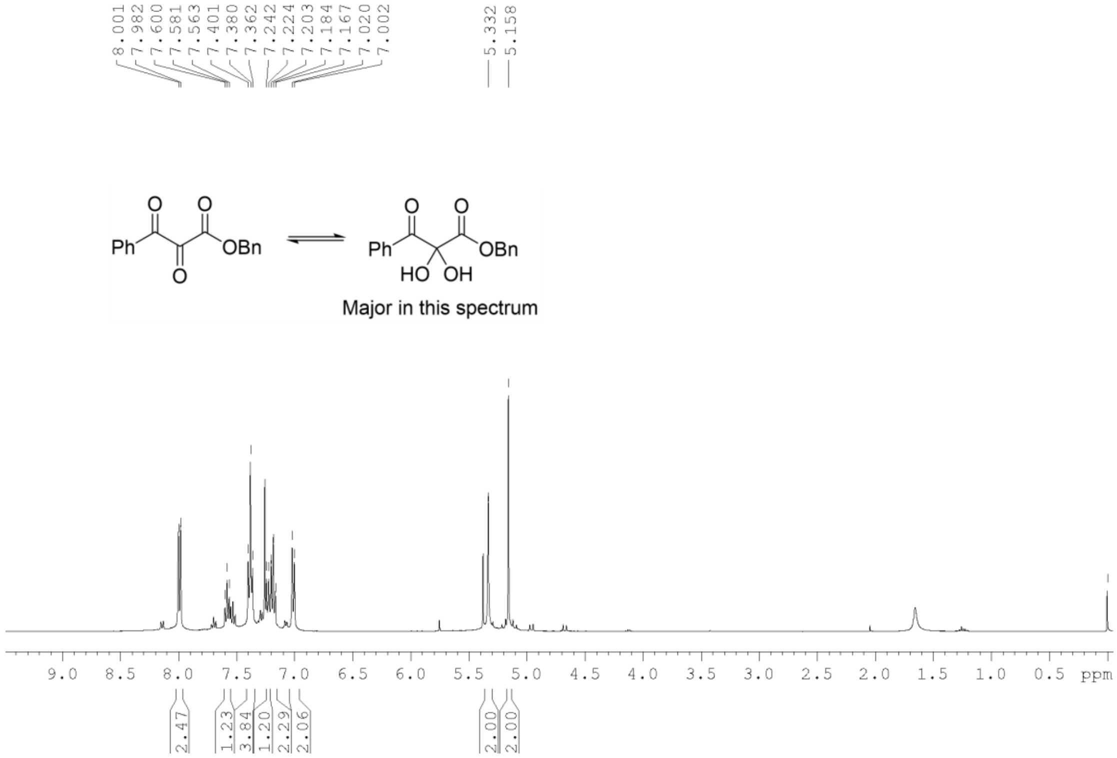

^{1}H NMR (400 MHz, $CDCl_3$) of **2d**

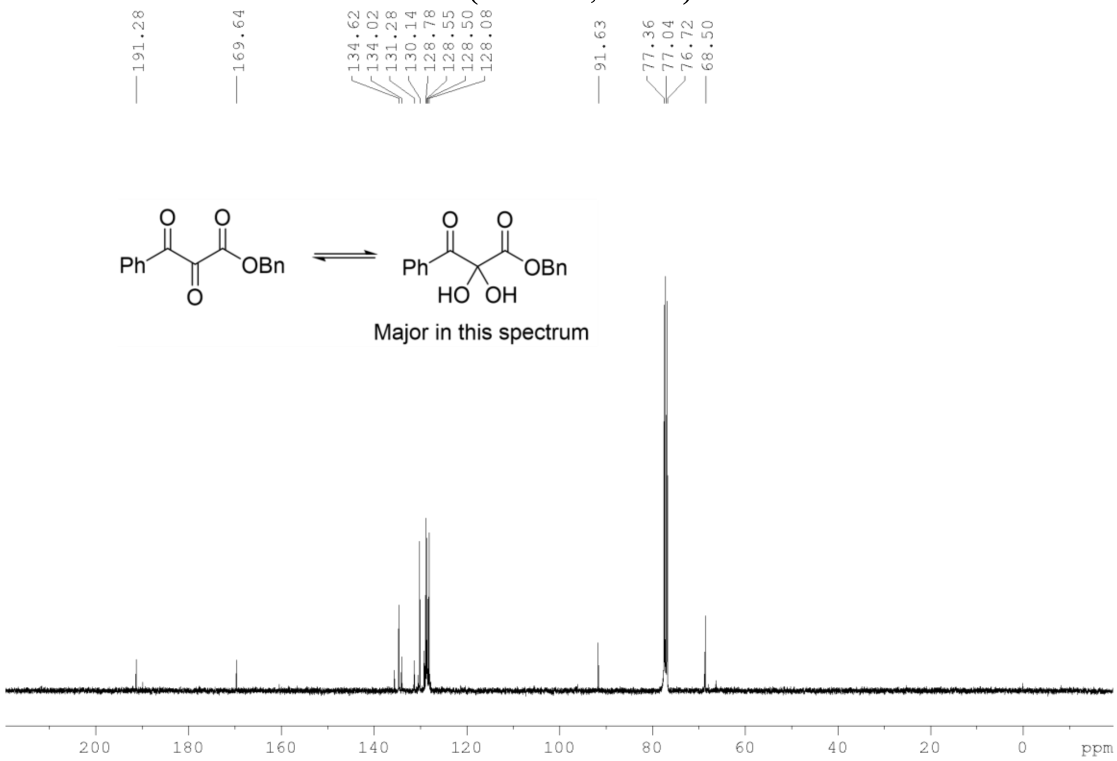

^{13}C NMR (100 MHz, $CDCl_3$) of **2d**

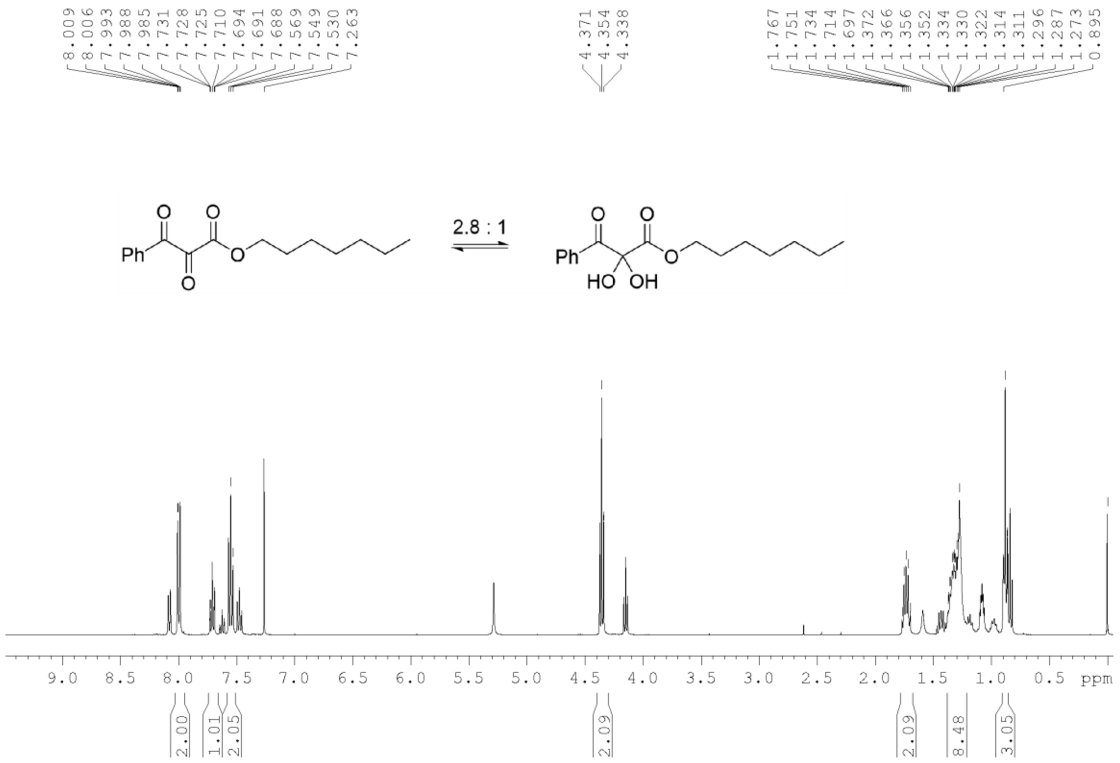

^{1}H NMR (400 MHz, CDCl3) of **2e**

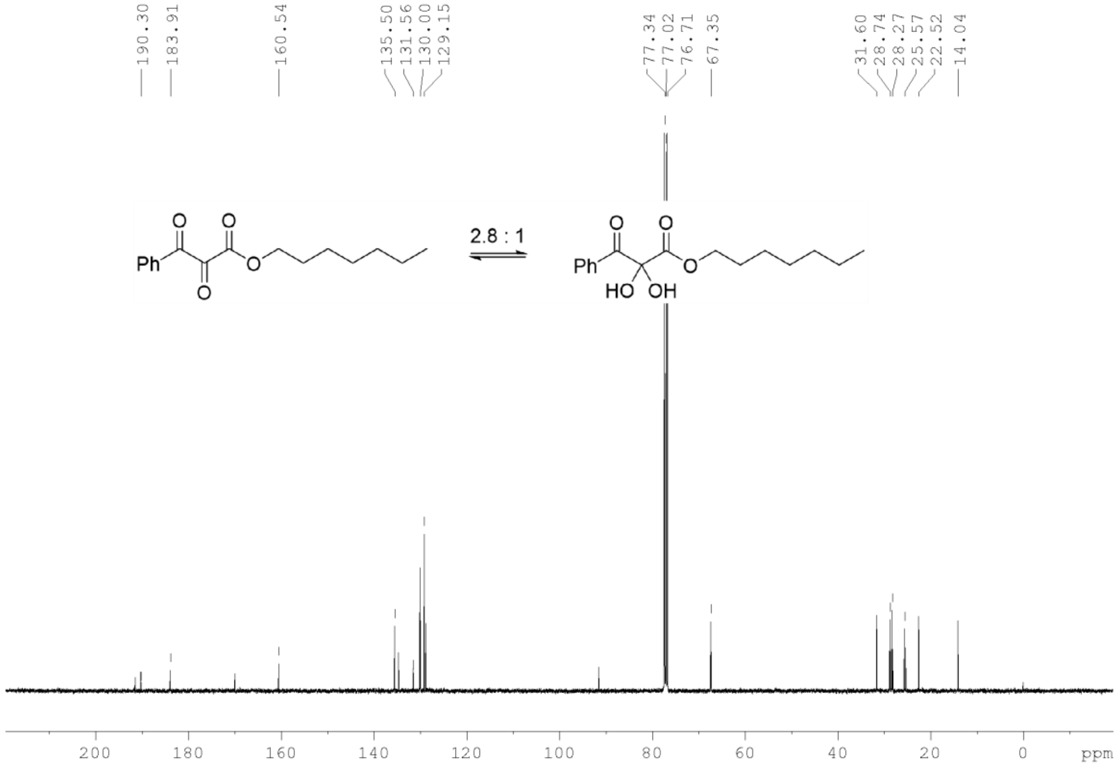

^{13}C NMR (100 MHz, CDCl3) of **2e**

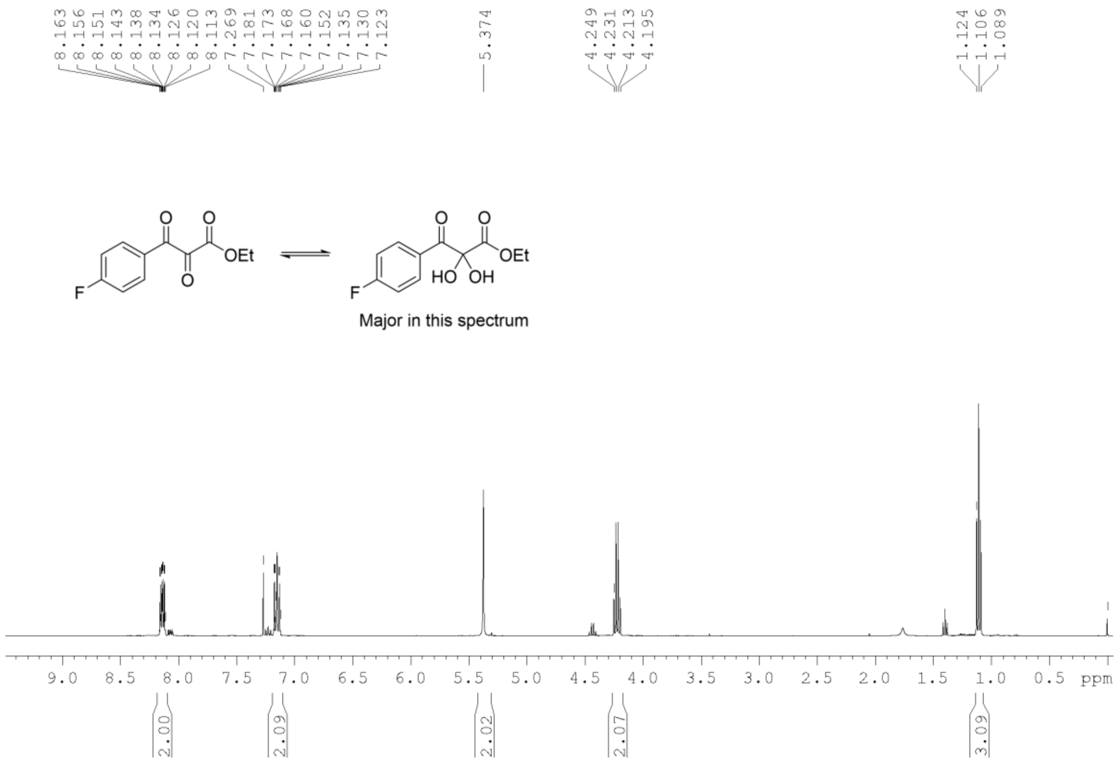

^{1}H NMR (400 MHz, $CDCl_3$) of **2f**

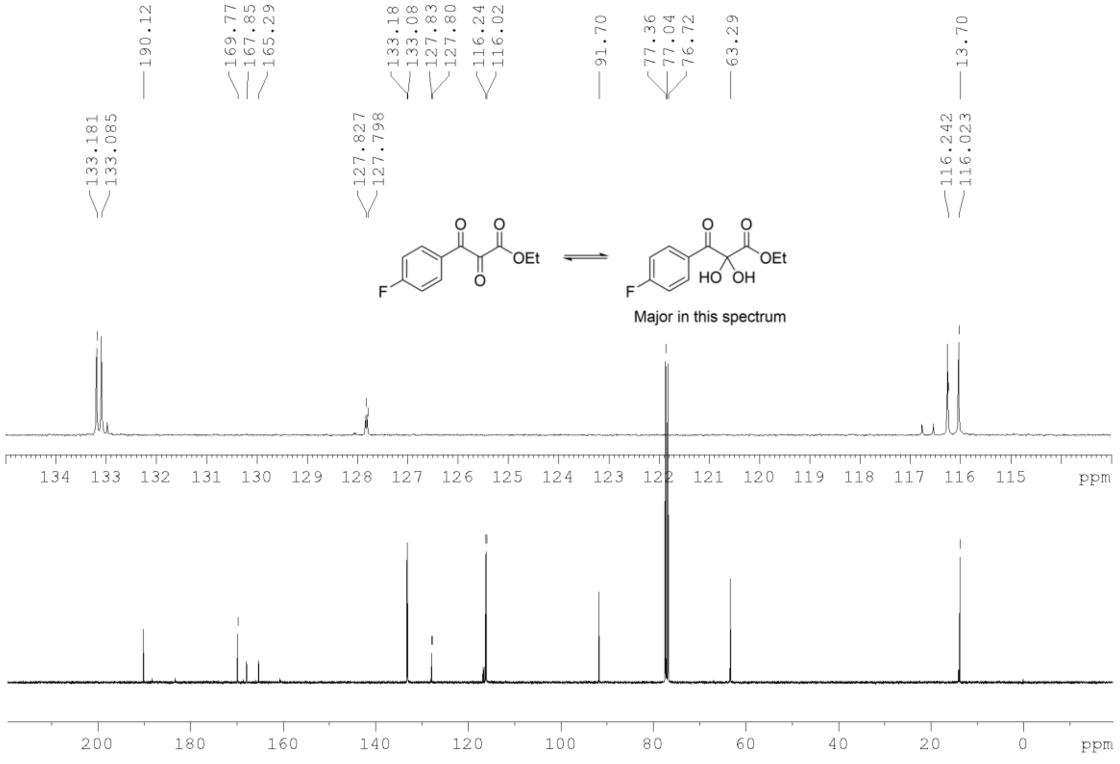

^{13}C NMR (100 MHz, $CDCl_3$) of **2f**

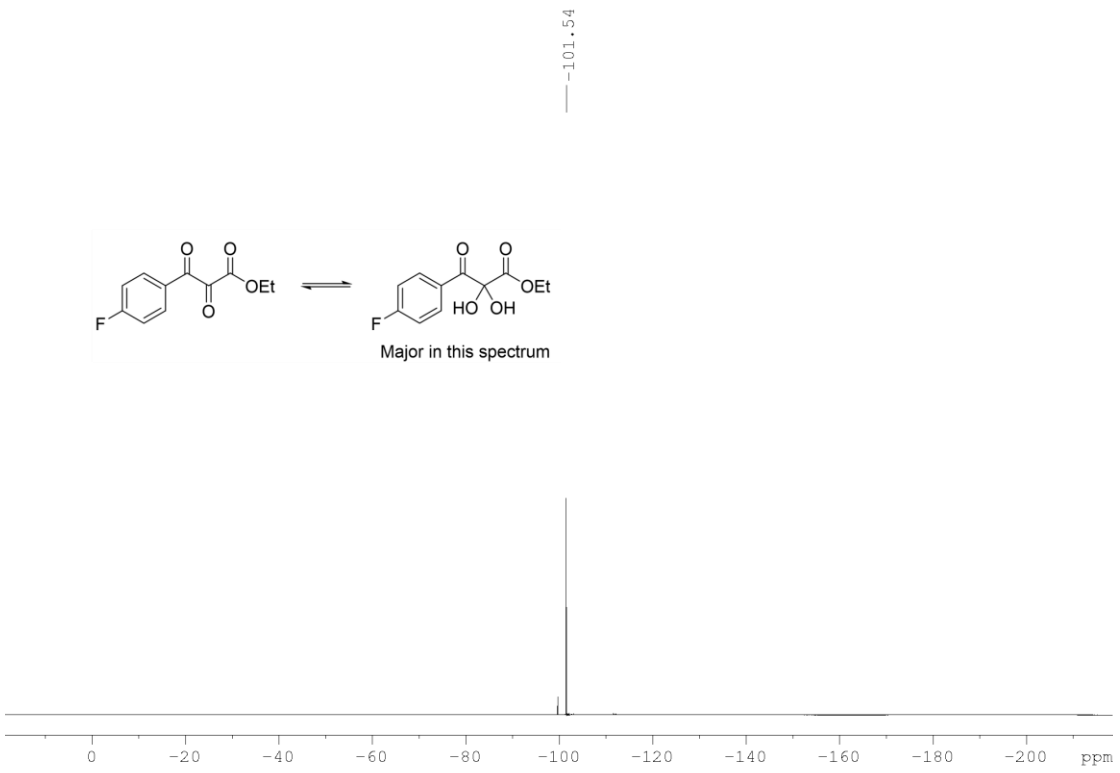

^{19}F NMR (376 MHz, CDCl$_3$) of **2f**

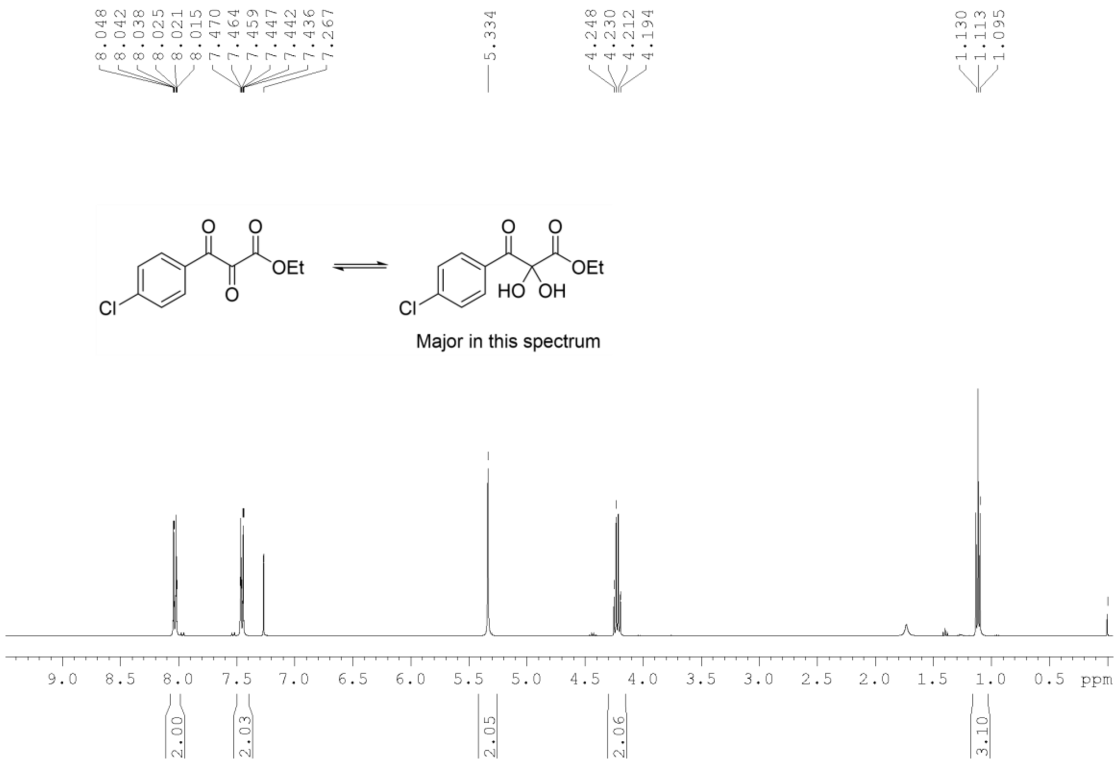

^{1}H NMR (400 MHz, CDCl$_3$) of **2g**

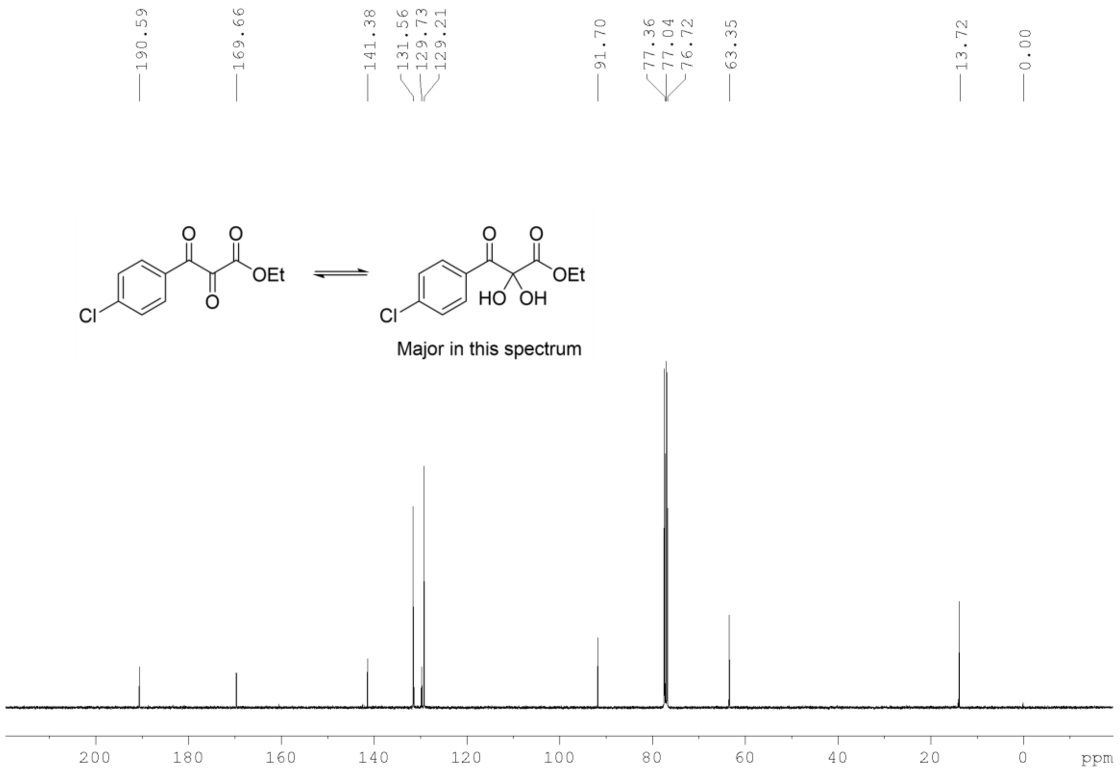

^{13}C NMR (100 MHz, $CDCl_3$) of **2g**

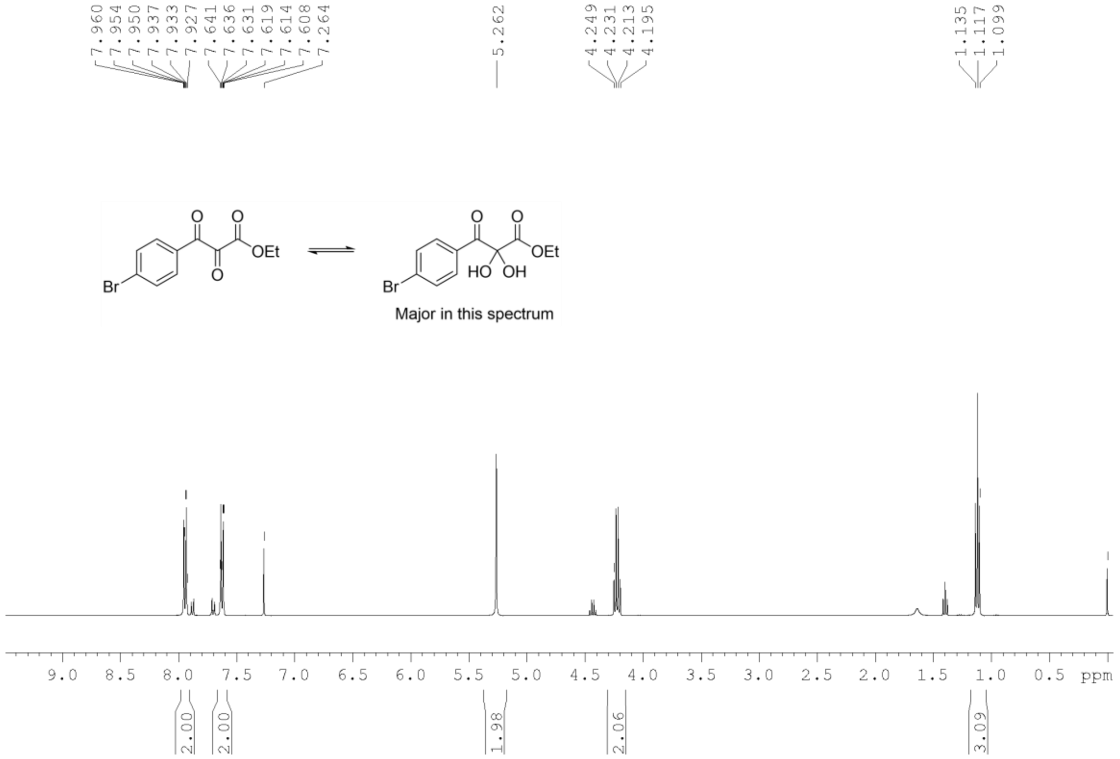

^{1}H NMR (400 MHz, $CDCl_3$) of **2h**

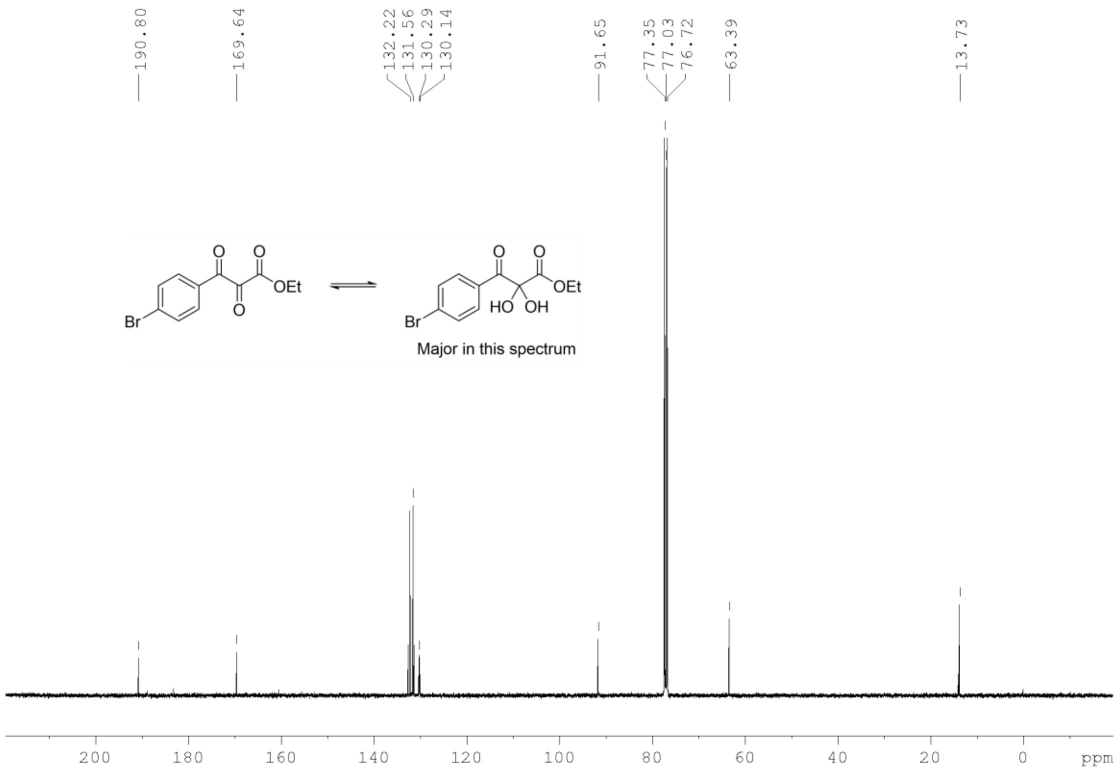

^{13}C NMR (100 MHz, CDCl$_3$) of **2h**

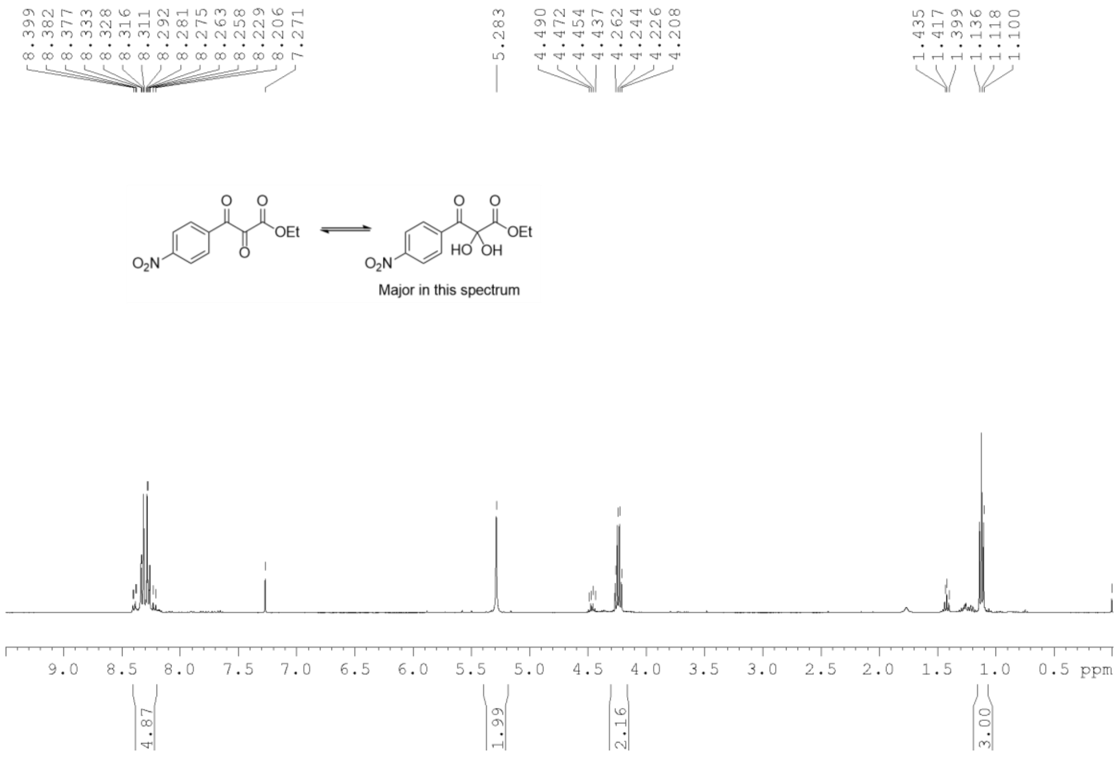

^{1}H NMR (400 MHz, CDCl$_3$) of **2i**

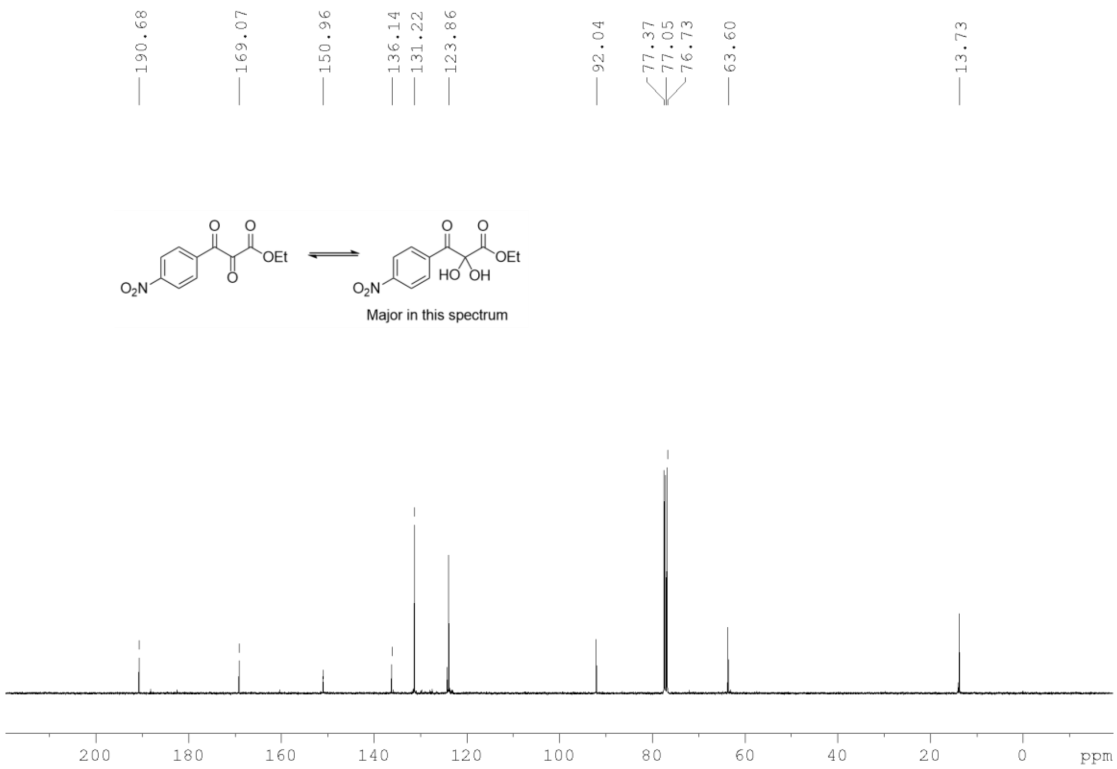

^{13}C NMR (100 MHz, CDCl$_3$) of **2i**

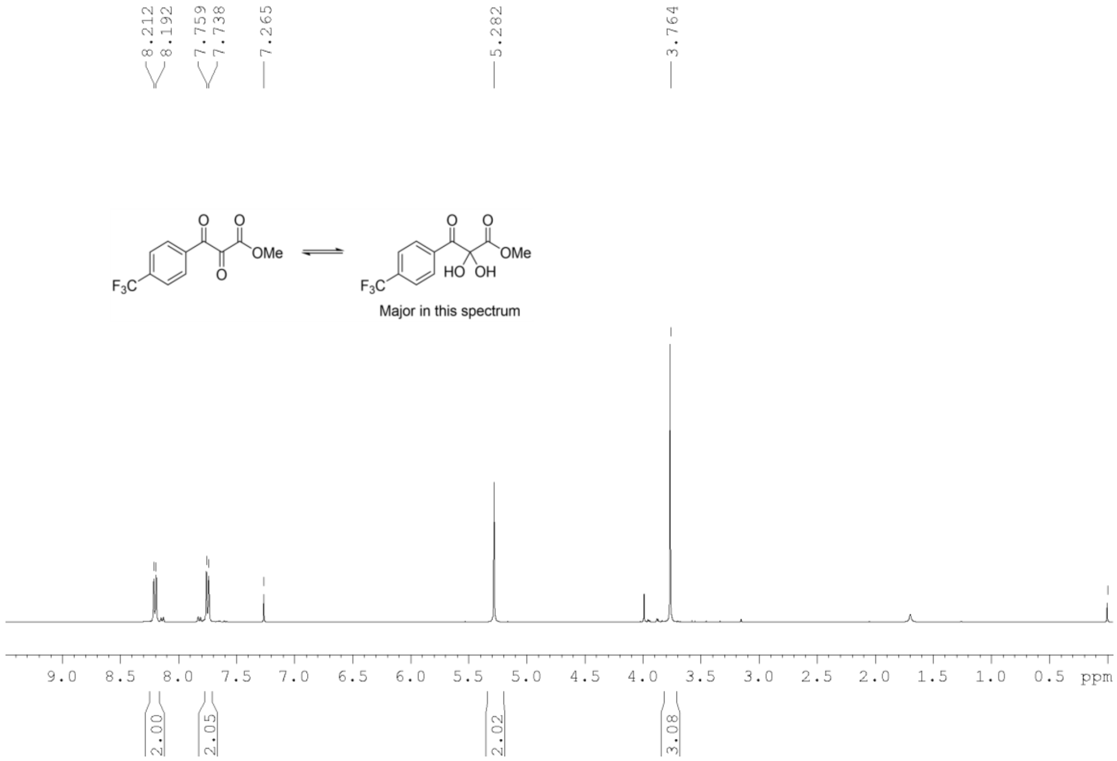

^{1}H NMR (400 MHz, CDCl$_3$) of **2j**

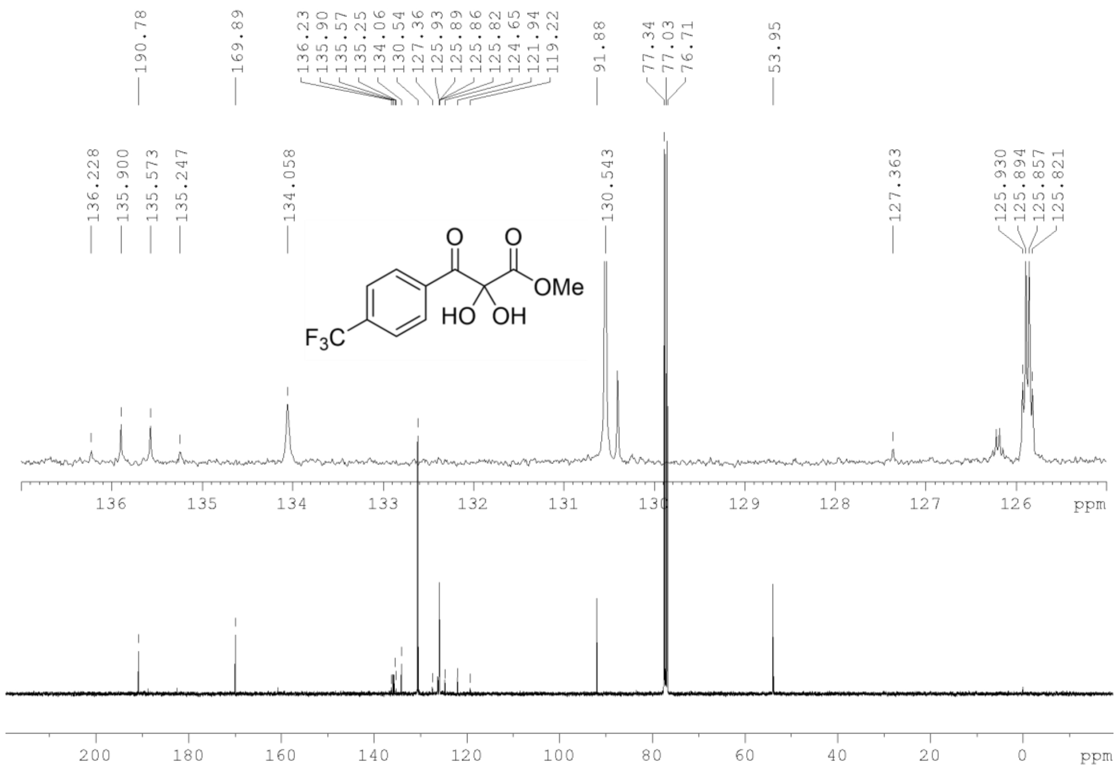

^{13}C NMR (100 MHz, CDCl$_3$) of **2j**

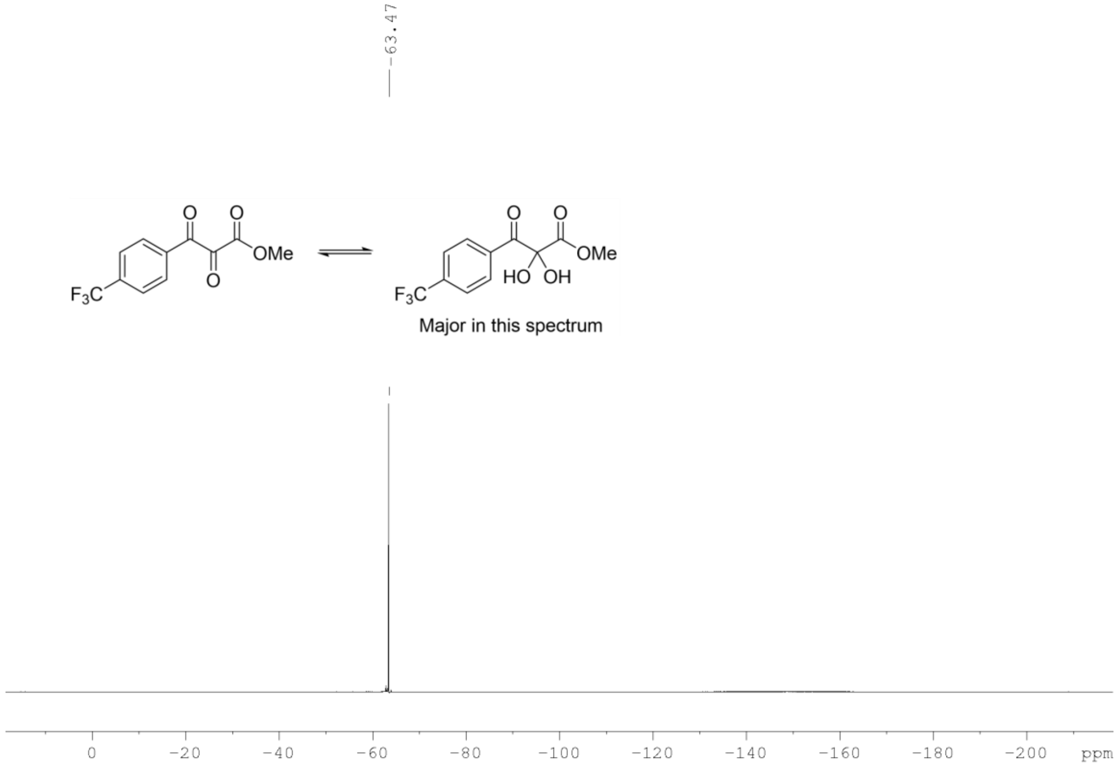

^{19}F NMR (376 MHz, CDCl$_3$) of **2j**

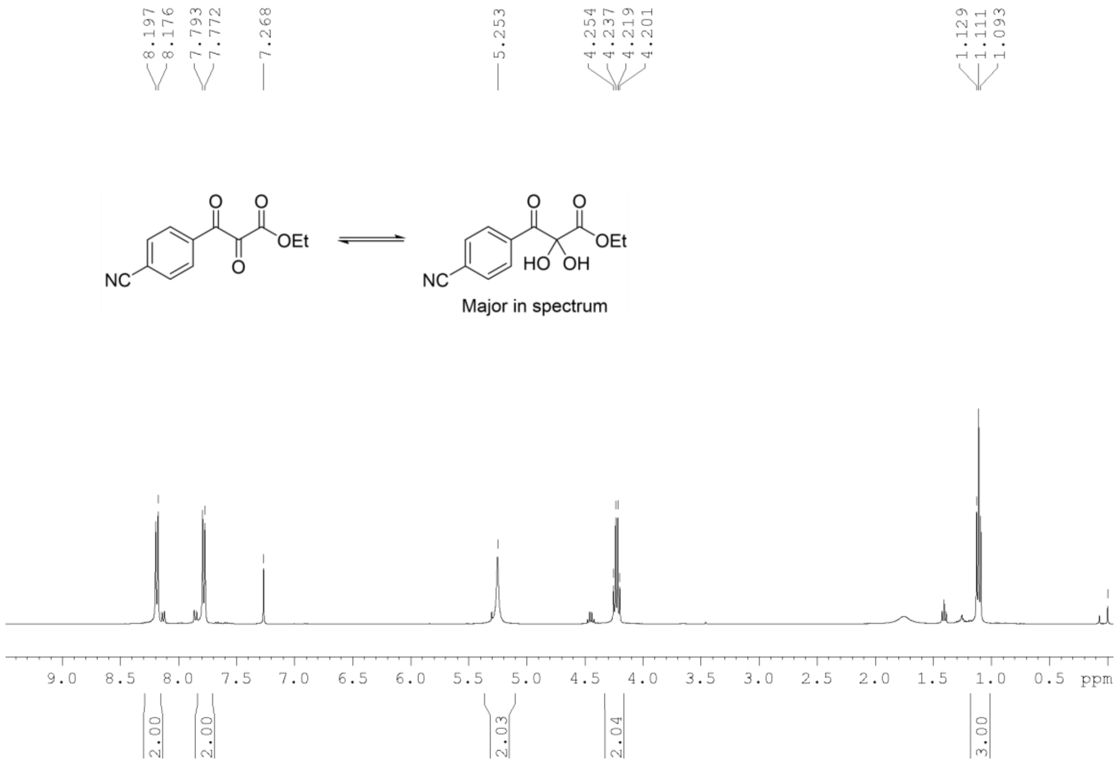

^{1}H NMR (400 MHz, CDCl3) of **2k**

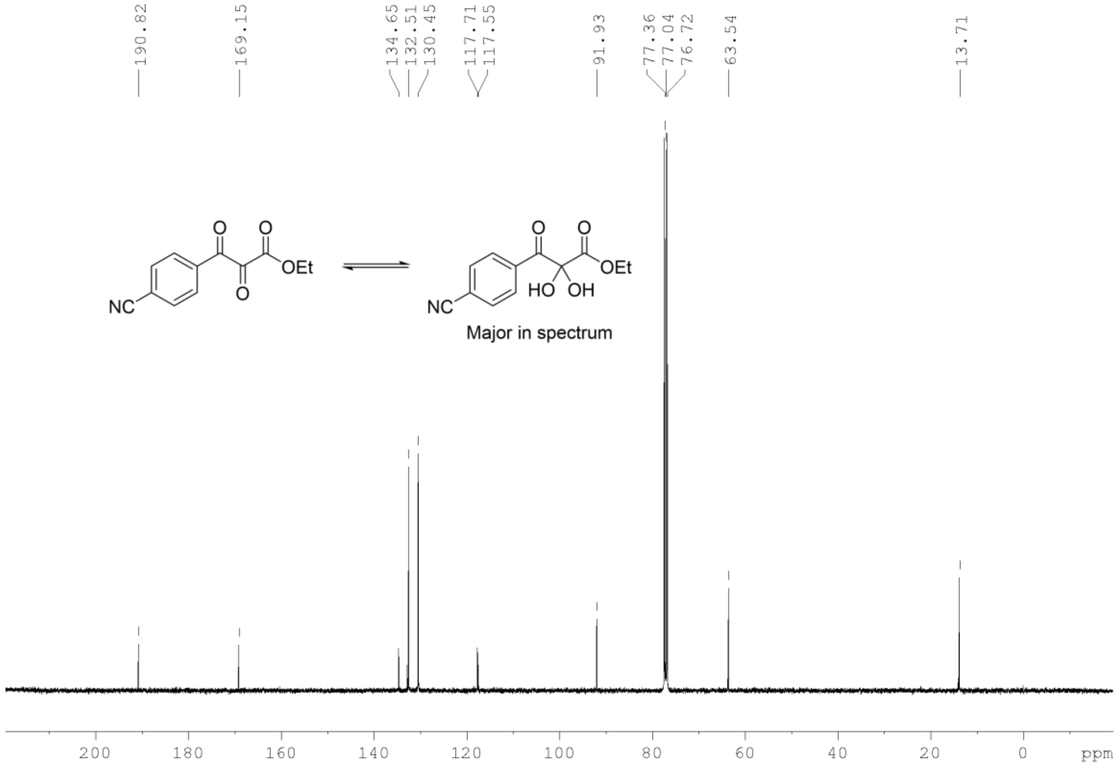

^{13}C NMR (100 MHz, CDCl3) of **2k**

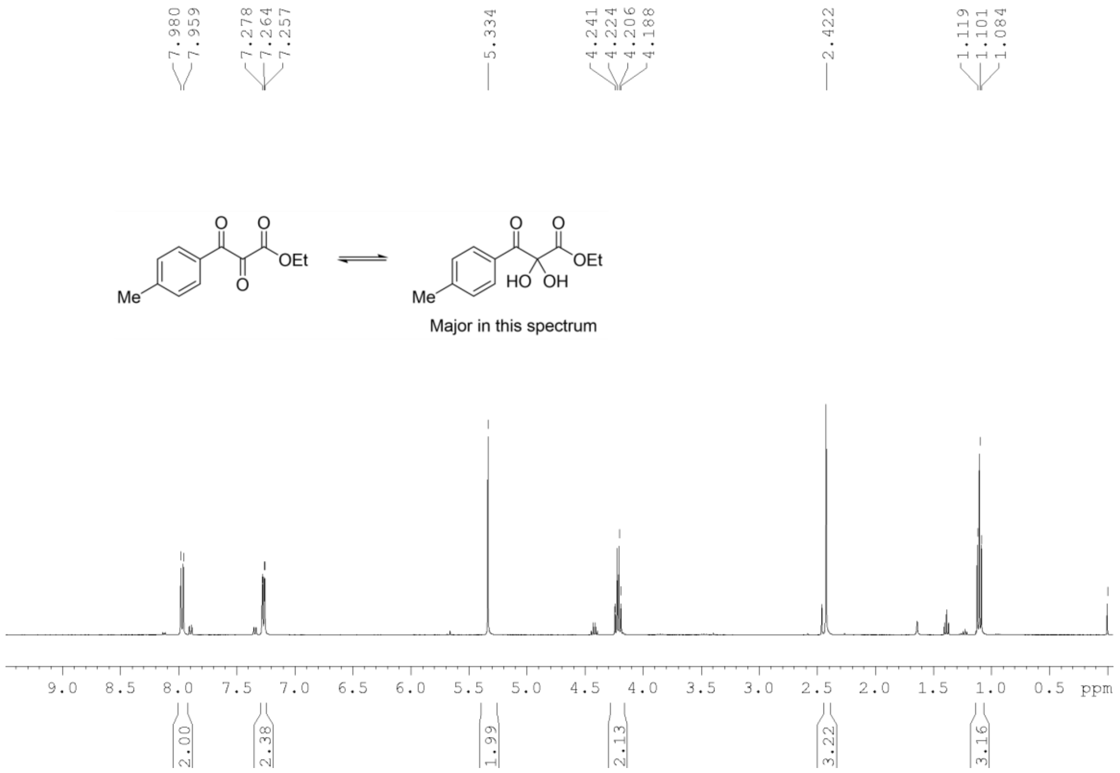

^{1}H NMR (400 MHz, $CDCl_3$) of **2l**

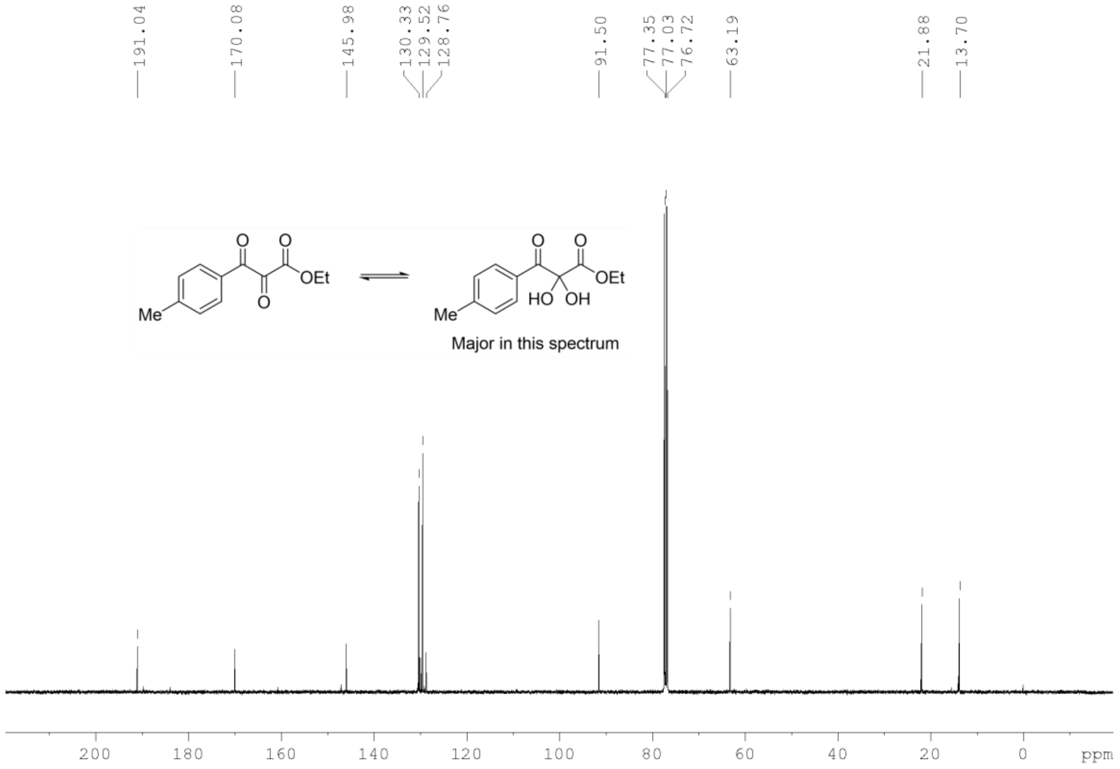

^{13}C NMR (100 MHz, $CDCl_3$) of **2l**

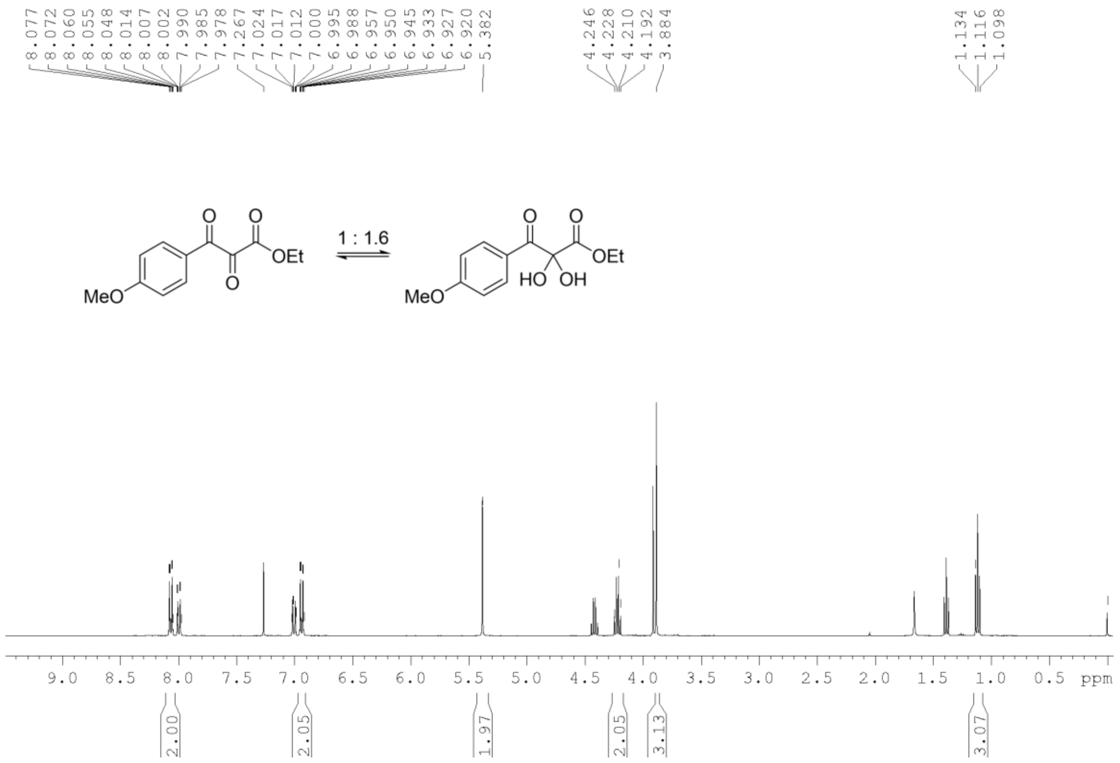

^{1}H NMR (400 MHz, $CDCl_3$) of **2m**

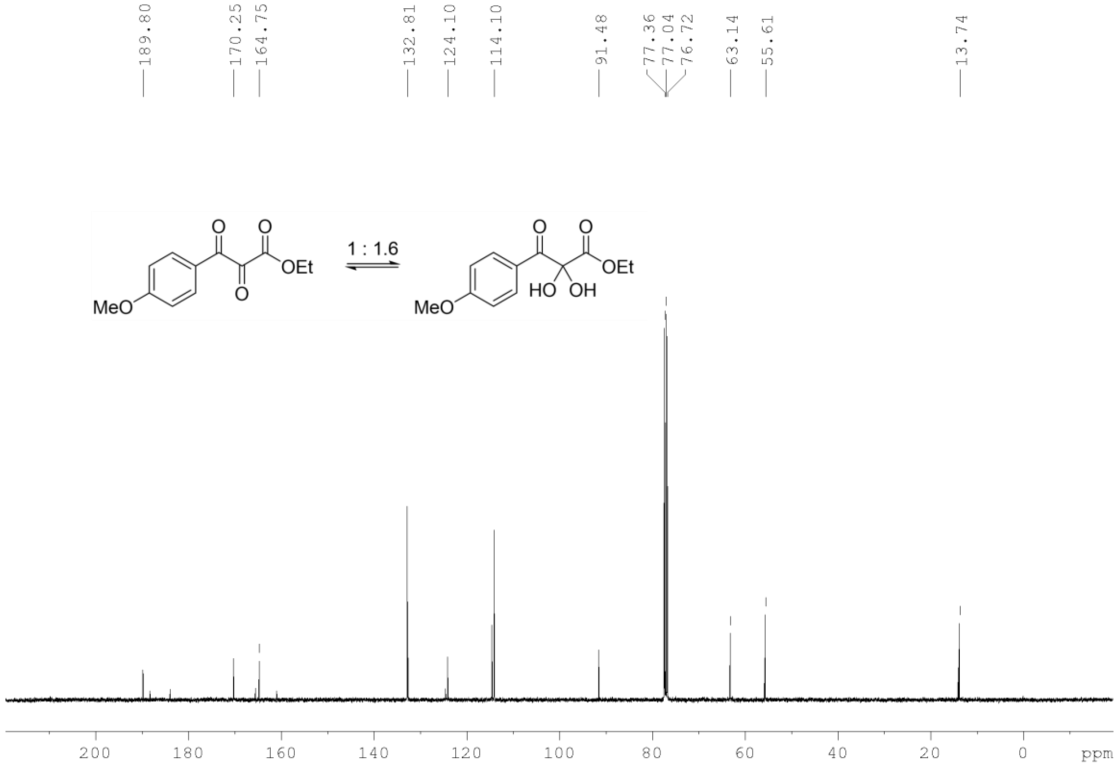

^{13}C NMR (100 MHz, $CDCl_3$) of **2m**

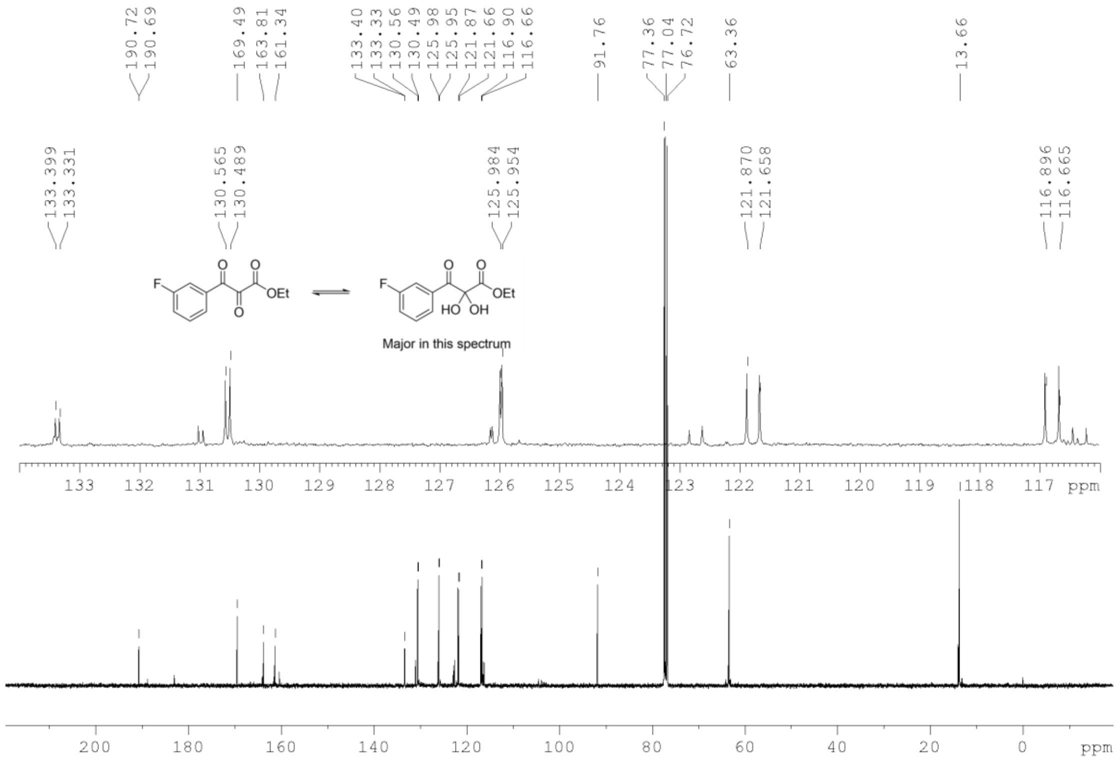

^{1}H NMR (400 MHz, $CDCl_3$) of **2n**

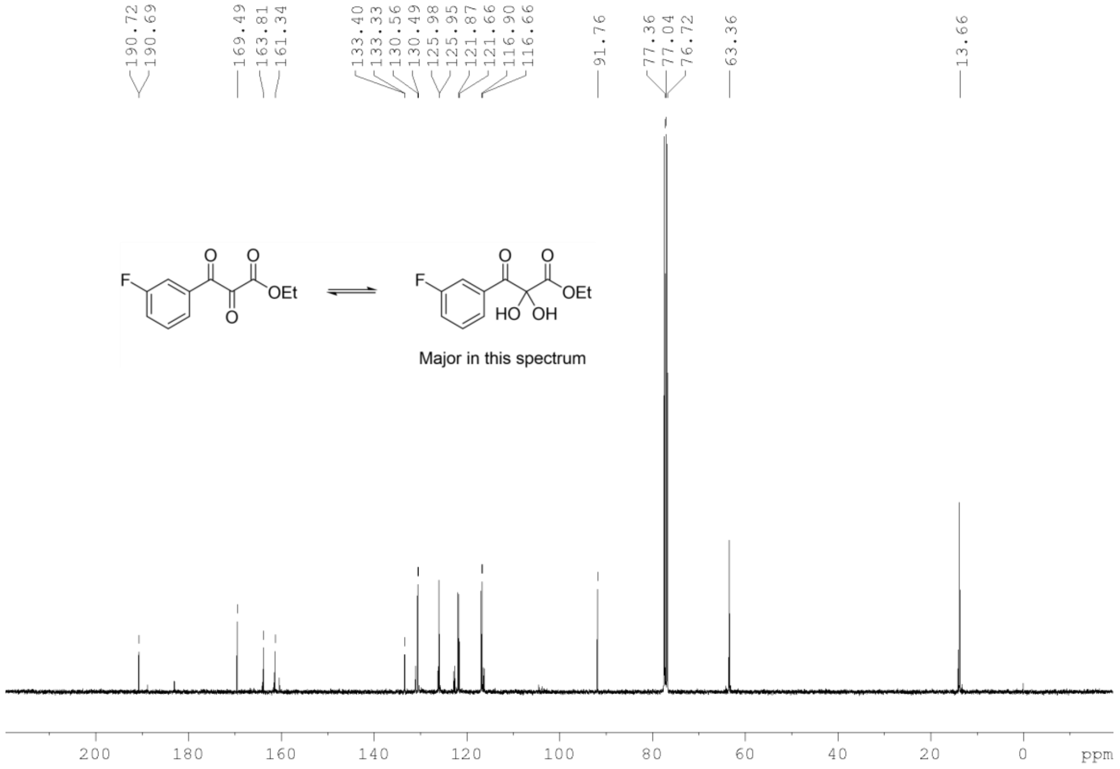

^{13}C NMR (100 MHz, $CDCl_3$) of **2n**

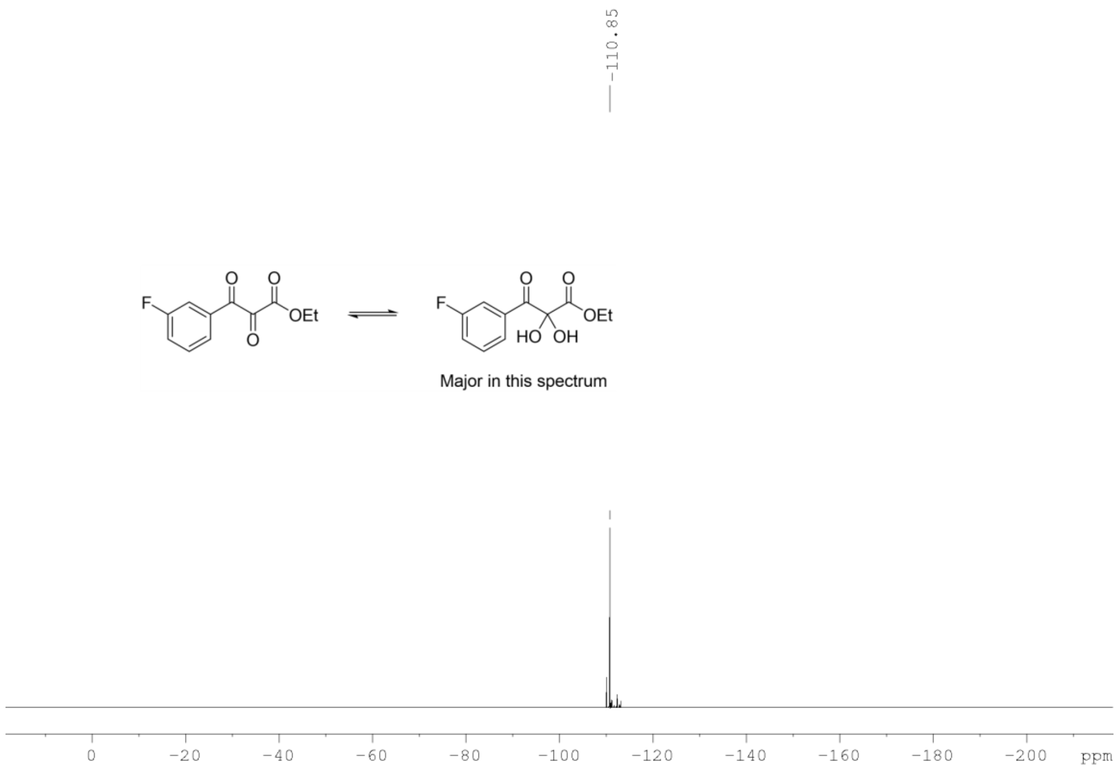

^{19}F NMR (376 MHz, $CDCl_3$) of **2n**

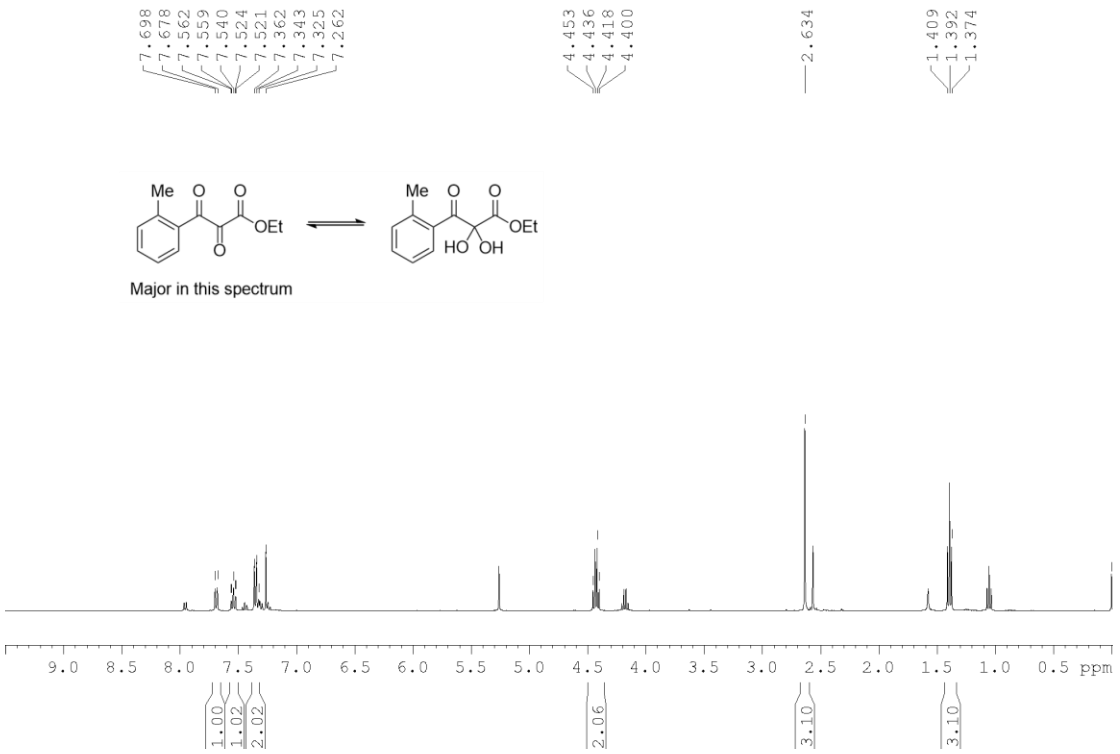

^{1}H NMR (400 MHz, $CDCl_3$) of **2o**

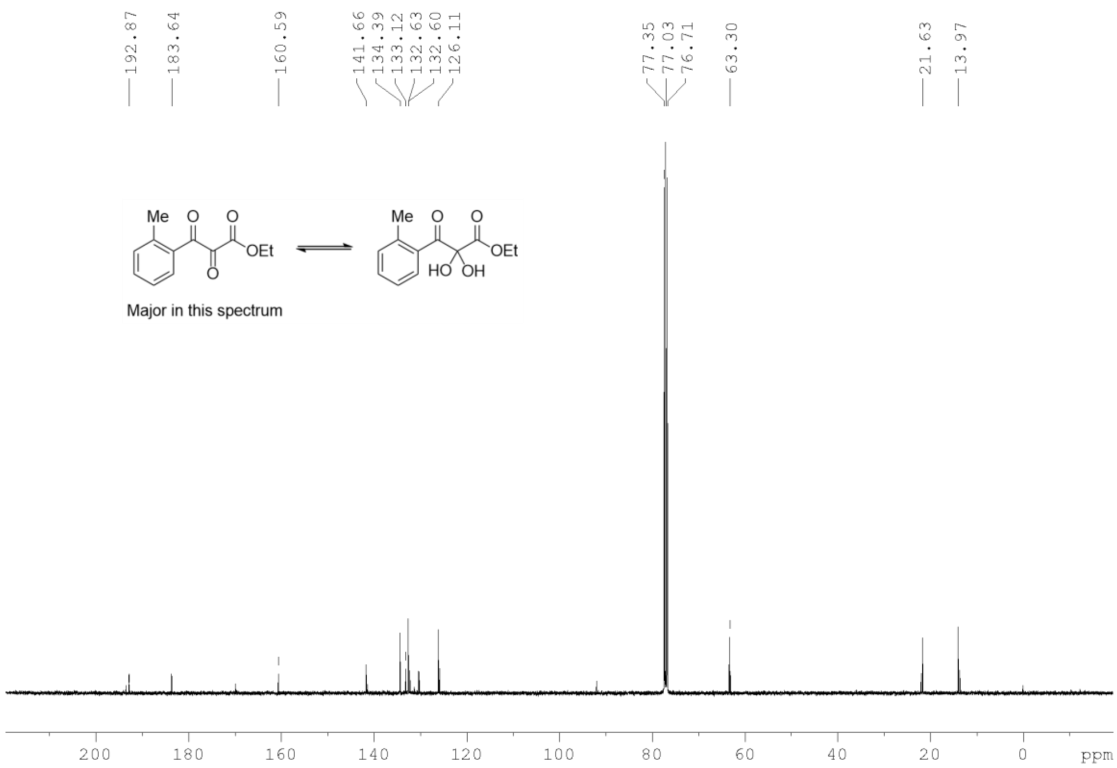

^{13}C NMR (100 MHz, CDCl$_3$) of **2o**

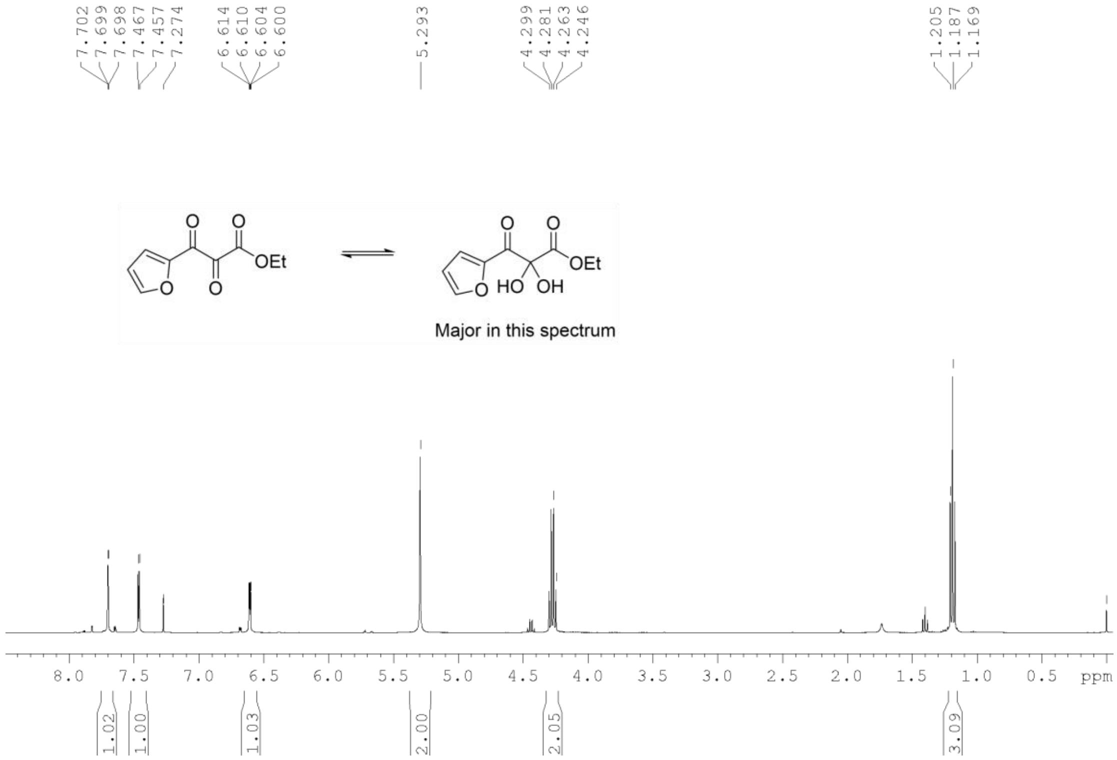

^{1}H NMR (400 MHz, CDCl$_3$) of **2p**

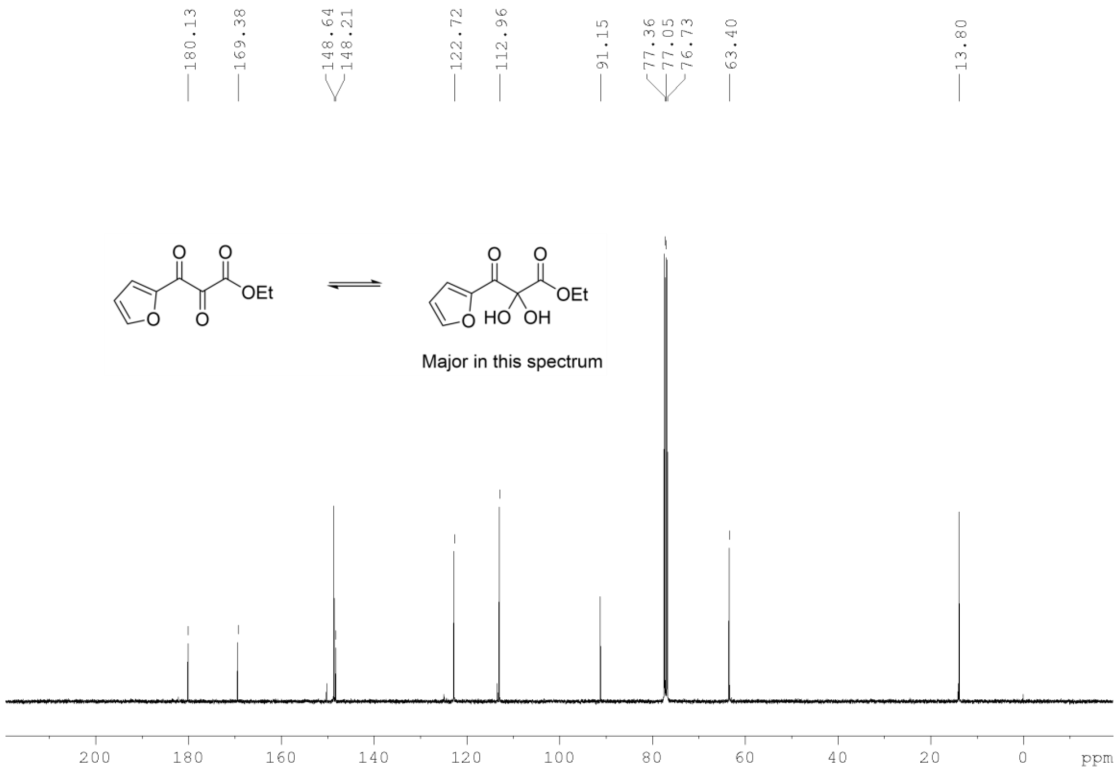

^{13}C NMR (100 MHz, $CDCl_3$) of **2p**

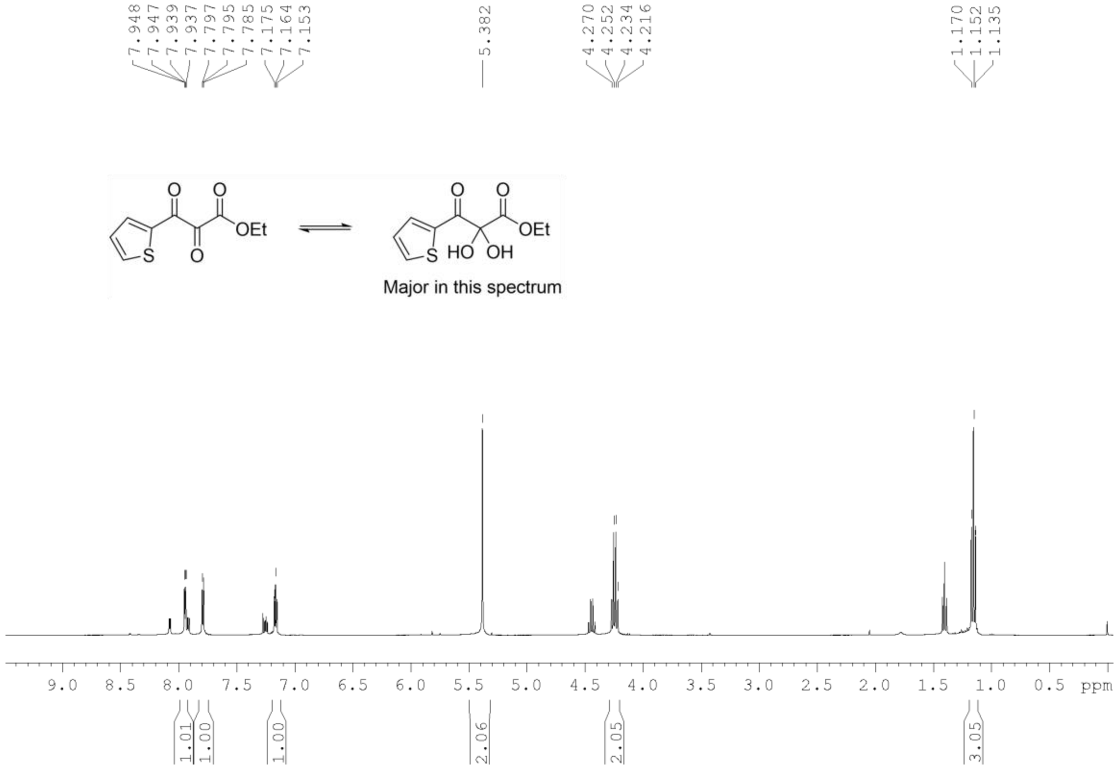

^{1}H NMR (400 MHz, $CDCl_3$) of **2q**

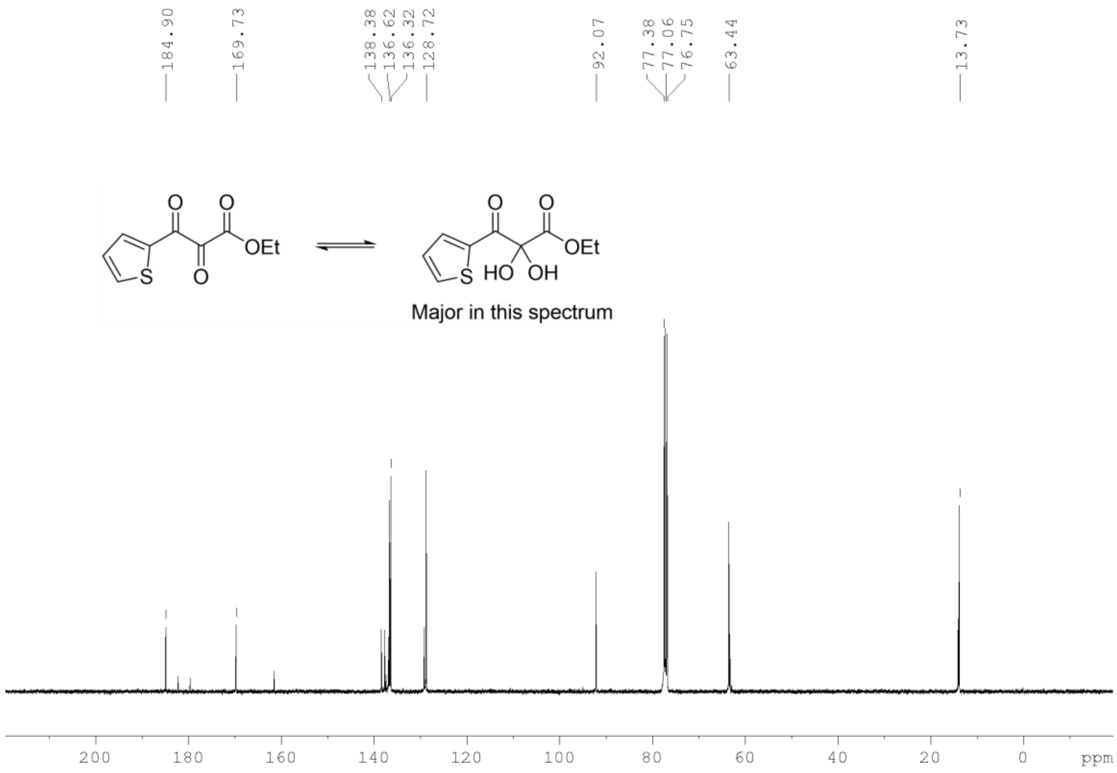

^{13}C NMR (100 MHz, CDCl$_3$) of **2q**

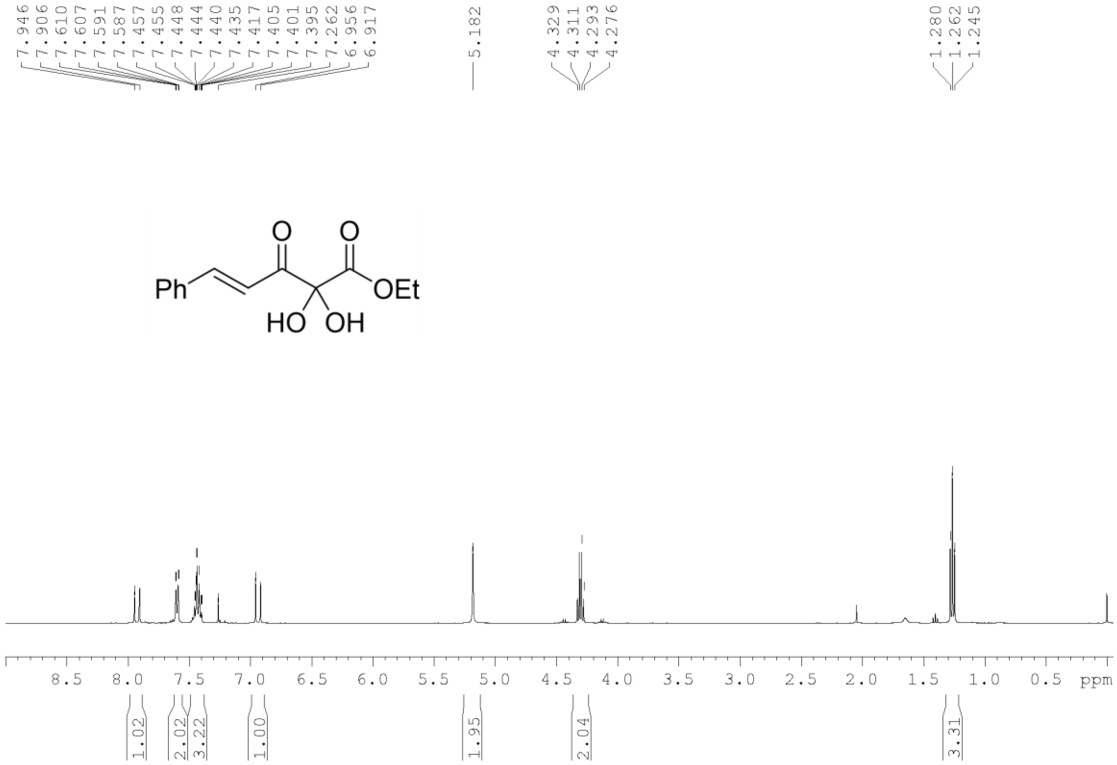

^{1}H NMR (400 MHz, CDCl$_3$) of **2r**

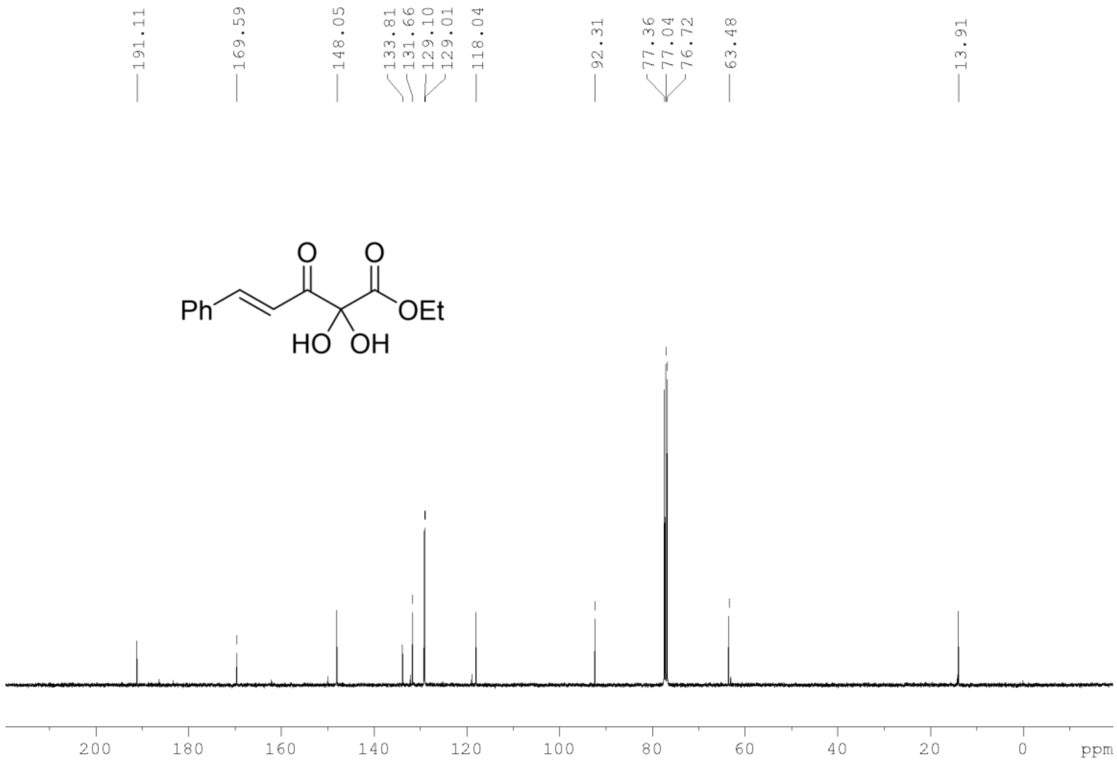

^{13}C NMR (100 MHz, CDCl$_3$) of **2r**

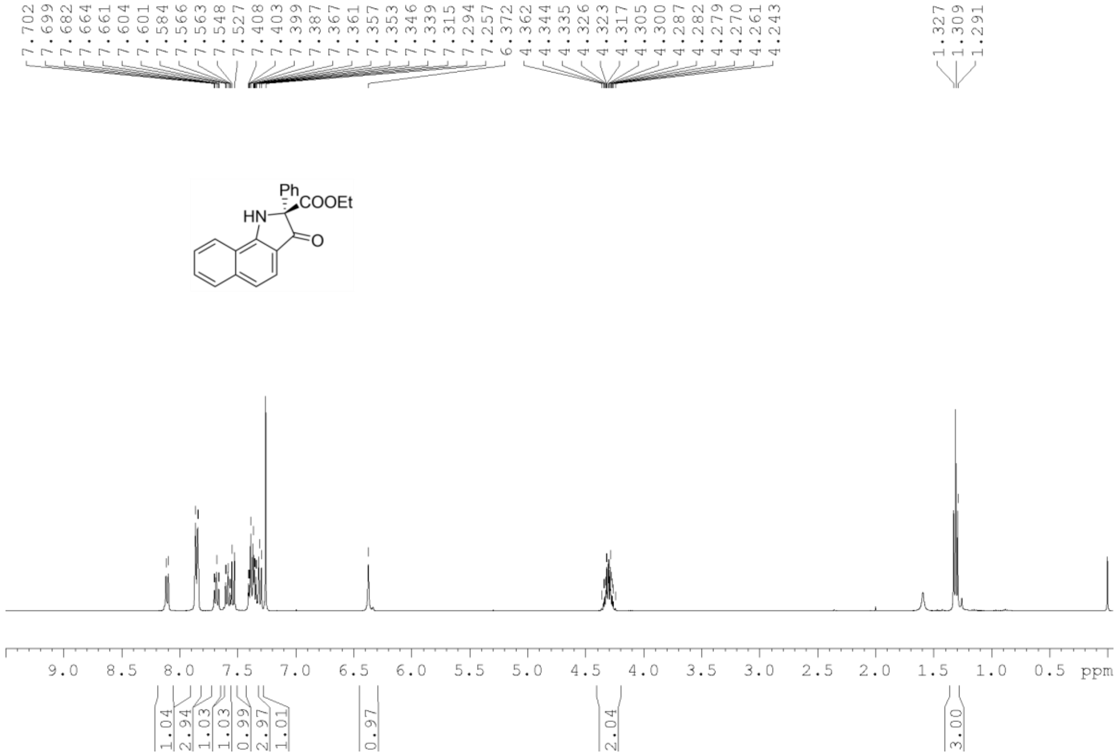

^{1}H NMR (400 MHz, CDCl$_3$) of **4aa**

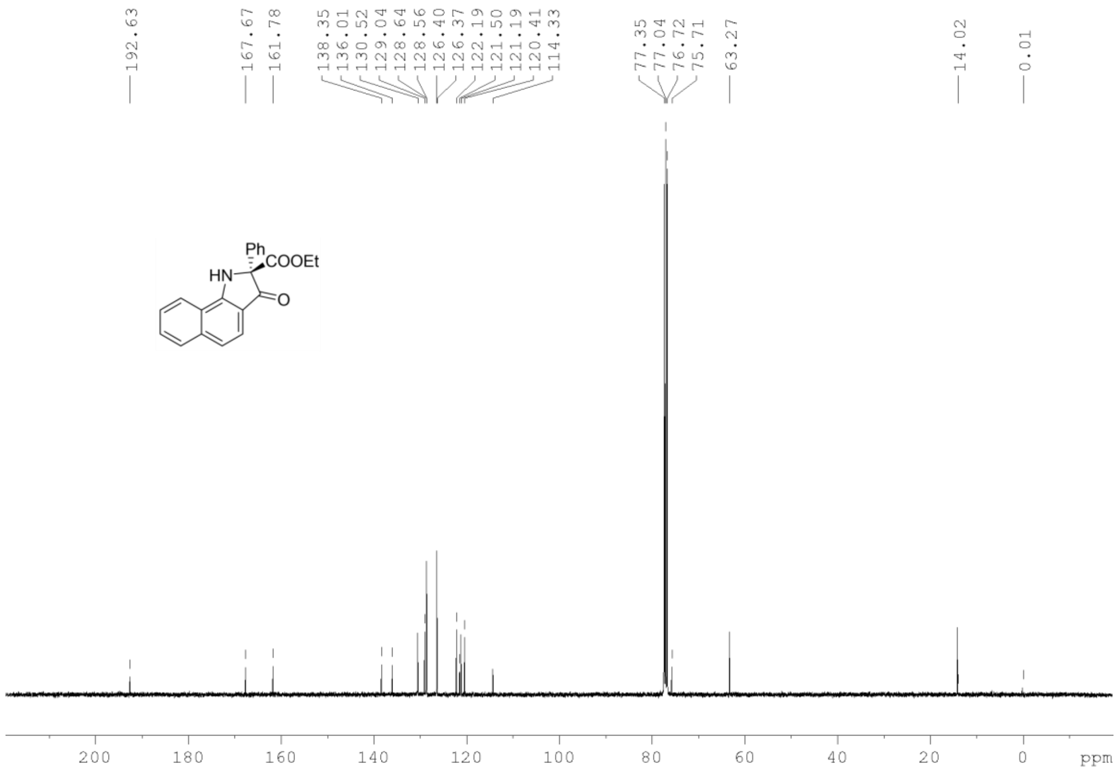

^{13}C NMR (100 MHz, $CDCl_3$) of **4aa**

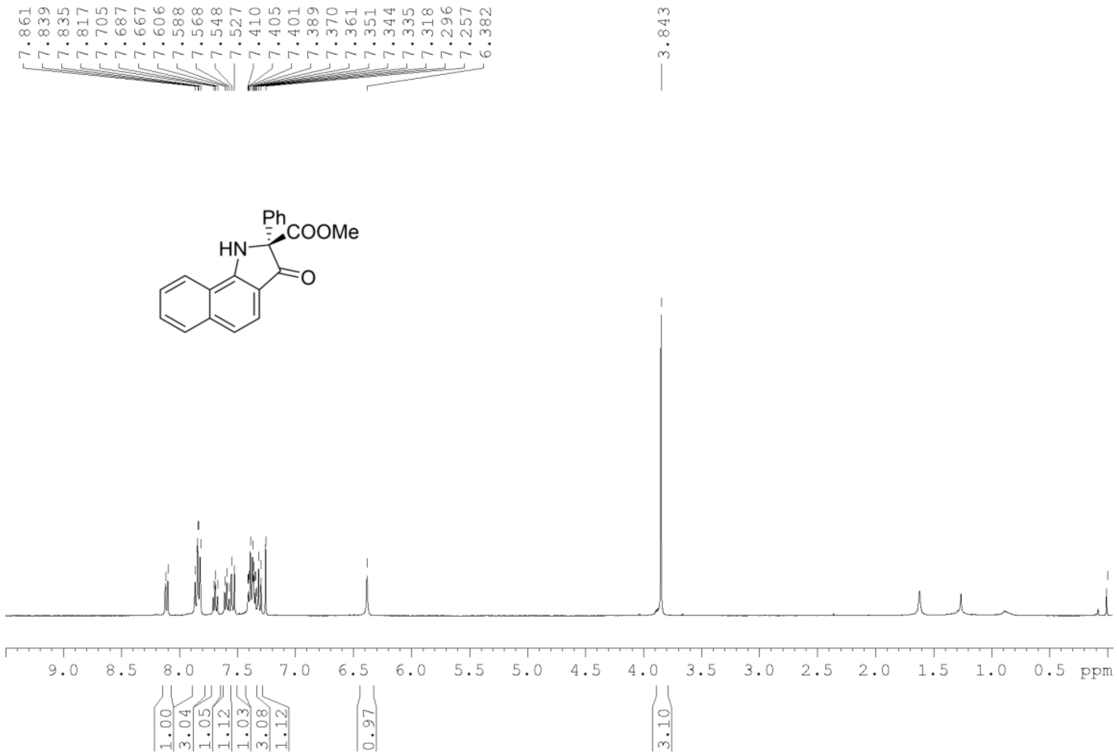

^{1}H NMR (400 MHz, $CDCl_3$) of **4ab**

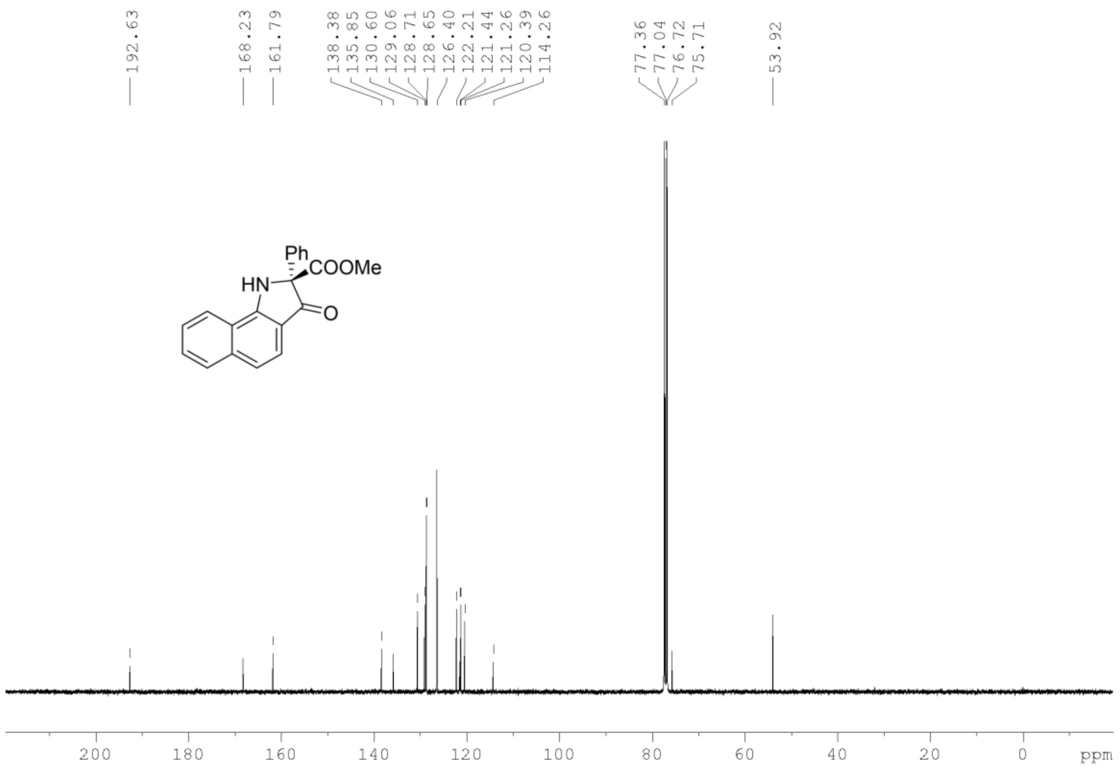

^{13}C NMR (100 MHz, $CDCl_3$) of **4ab**

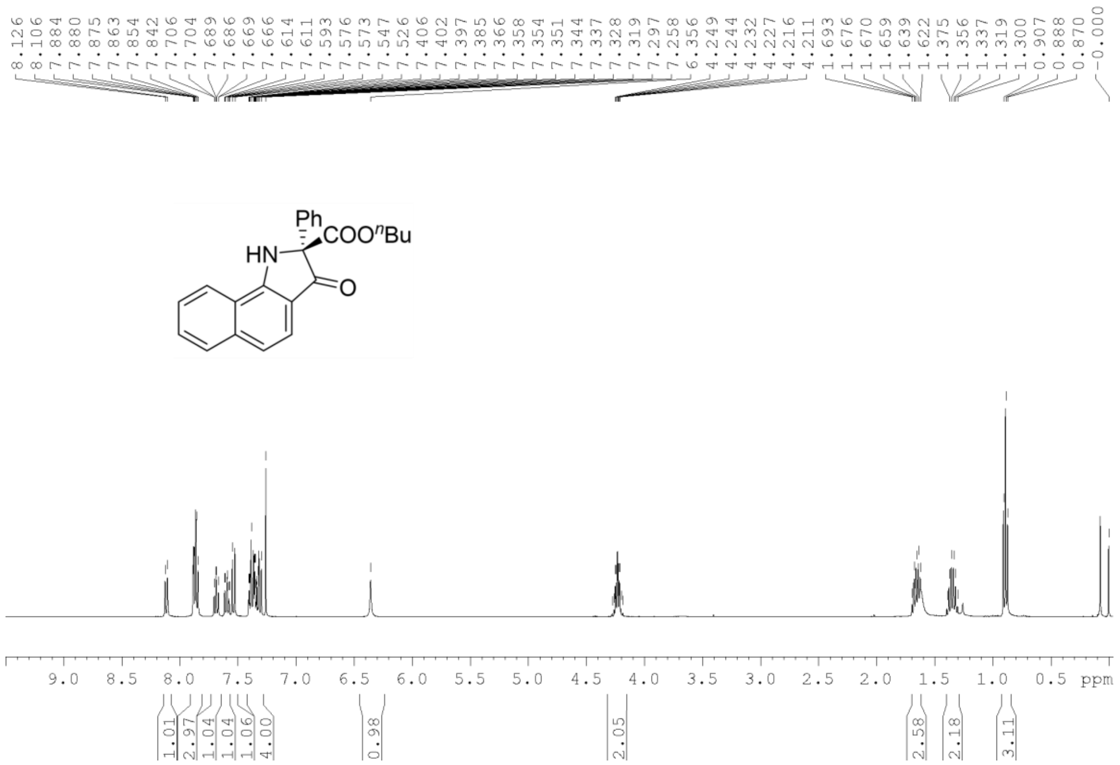

^{1}H NMR (400 MHz, $CDCl_3$) of **4ac**

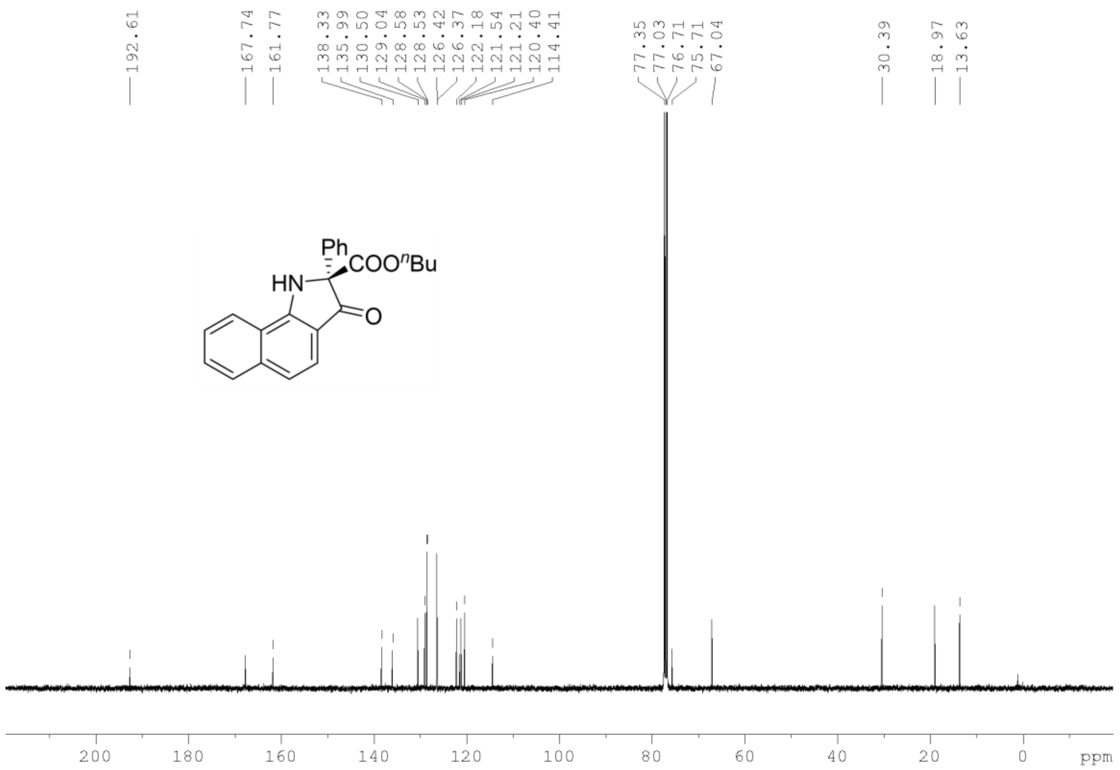

^{13}C NMR (100 MHz, $CDCl_3$) of **4ac**

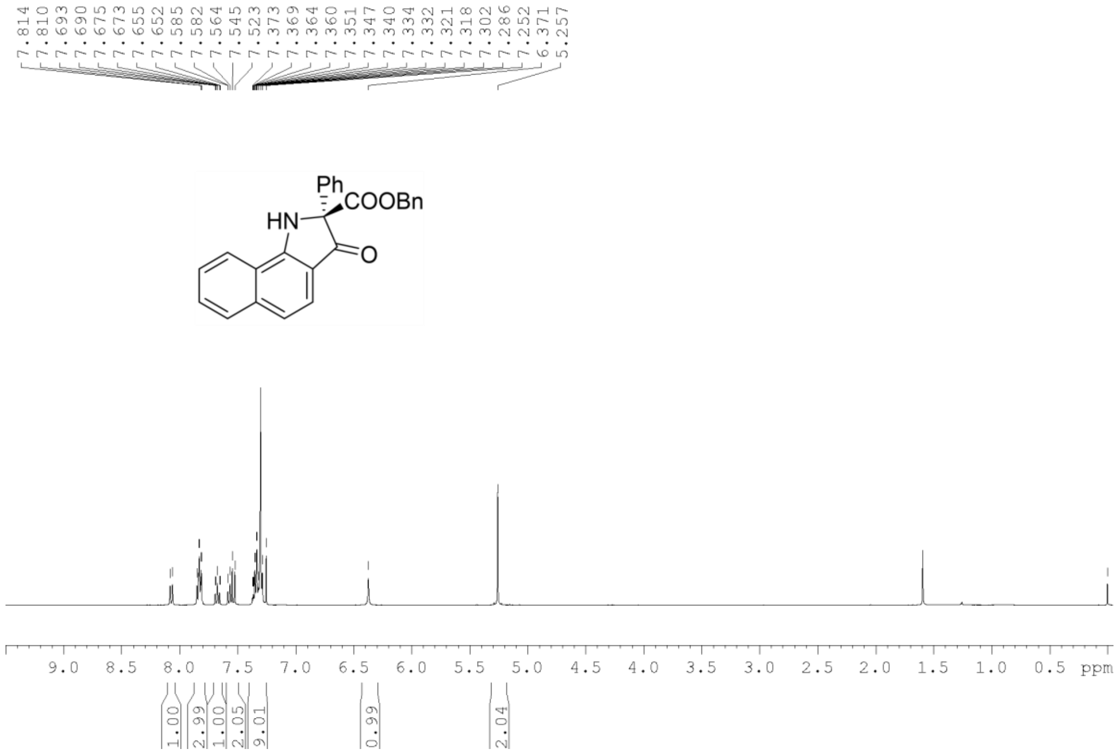

^{1}H NMR (400 MHz, $CDCl_3$) of **4ad**

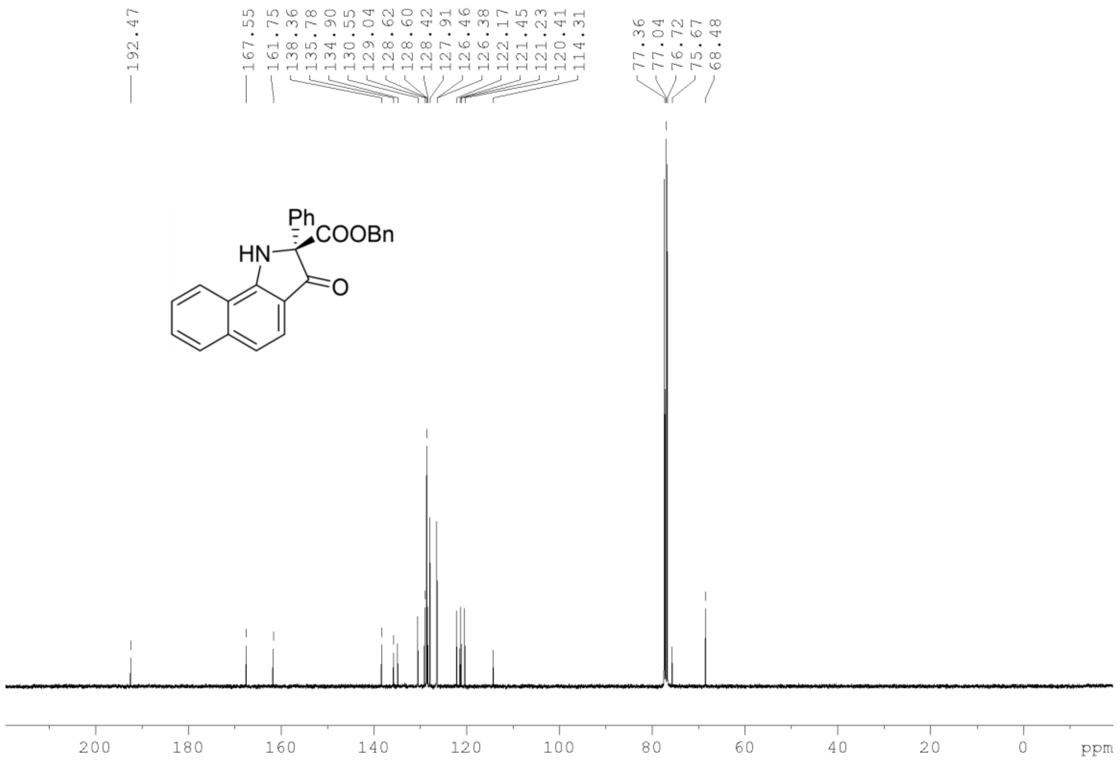

^{13}C NMR (100 MHz, $CDCl_3$) of **4ad**

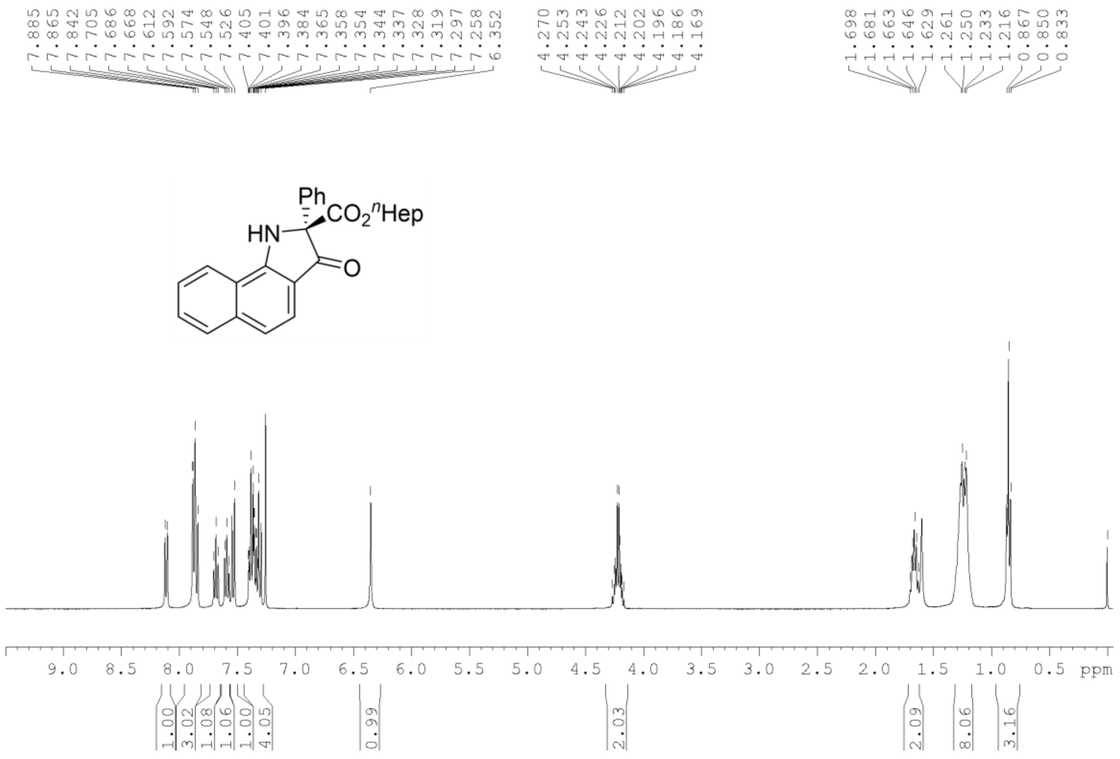

^{1}H NMR (400 MHz, $CDCl_3$) of **4ae**

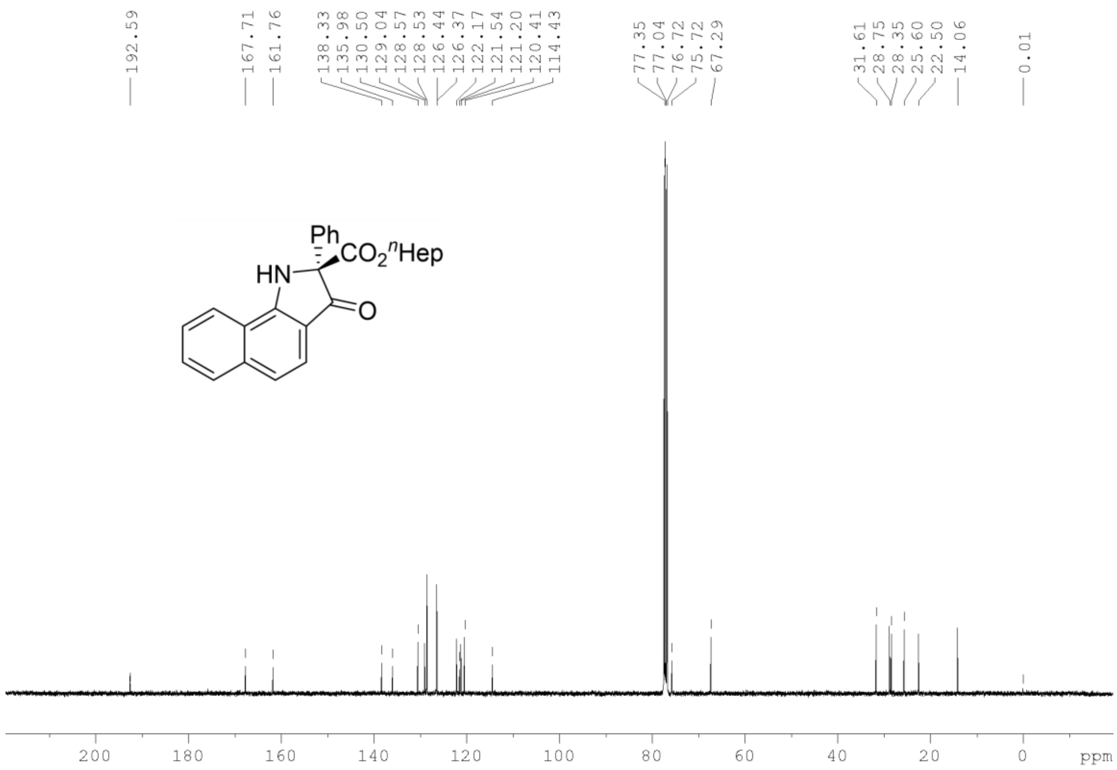

^{13}C NMR (100 MHz, $CDCl_3$) of **4ae**

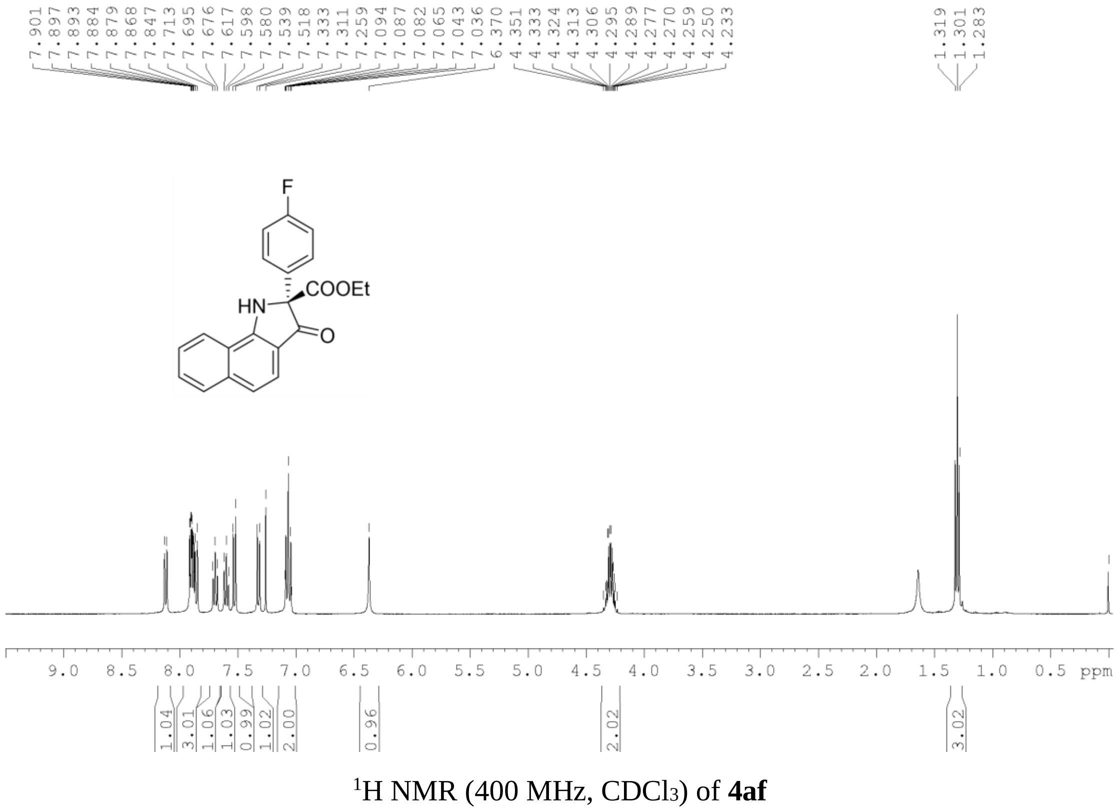

^{1}H NMR (400 MHz, $CDCl_3$) of **4af**

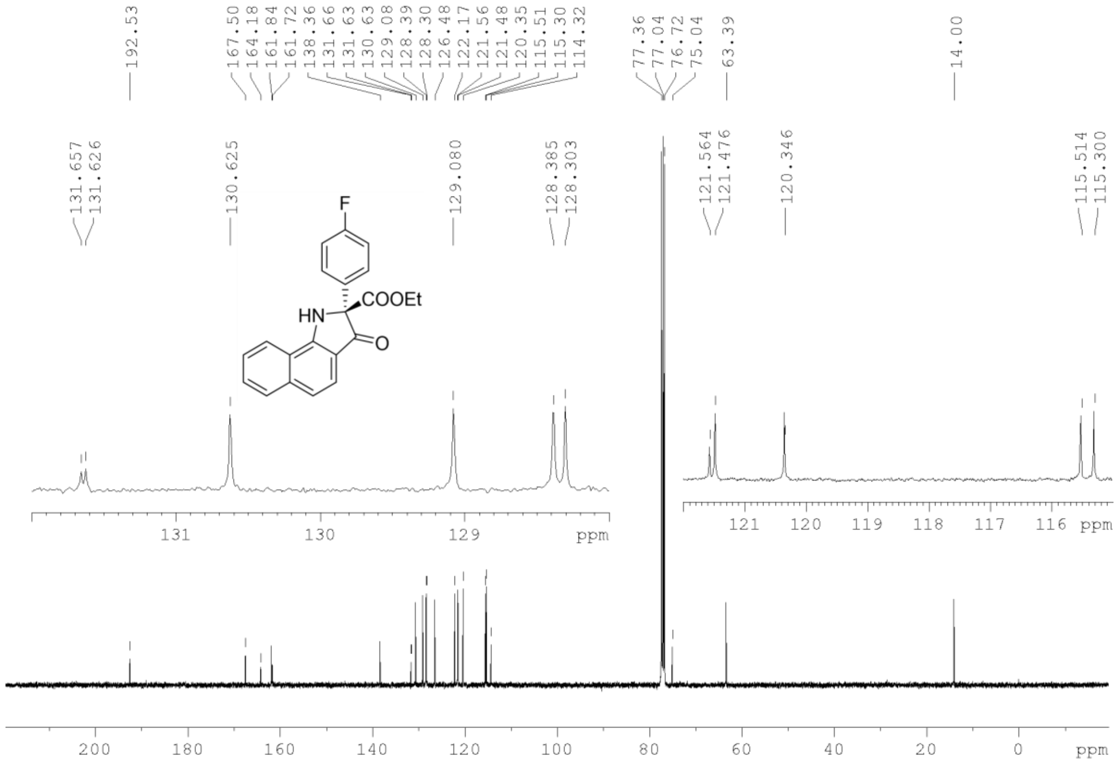

^{13}C NMR (100 MHz, $CDCl_3$) of **4af**

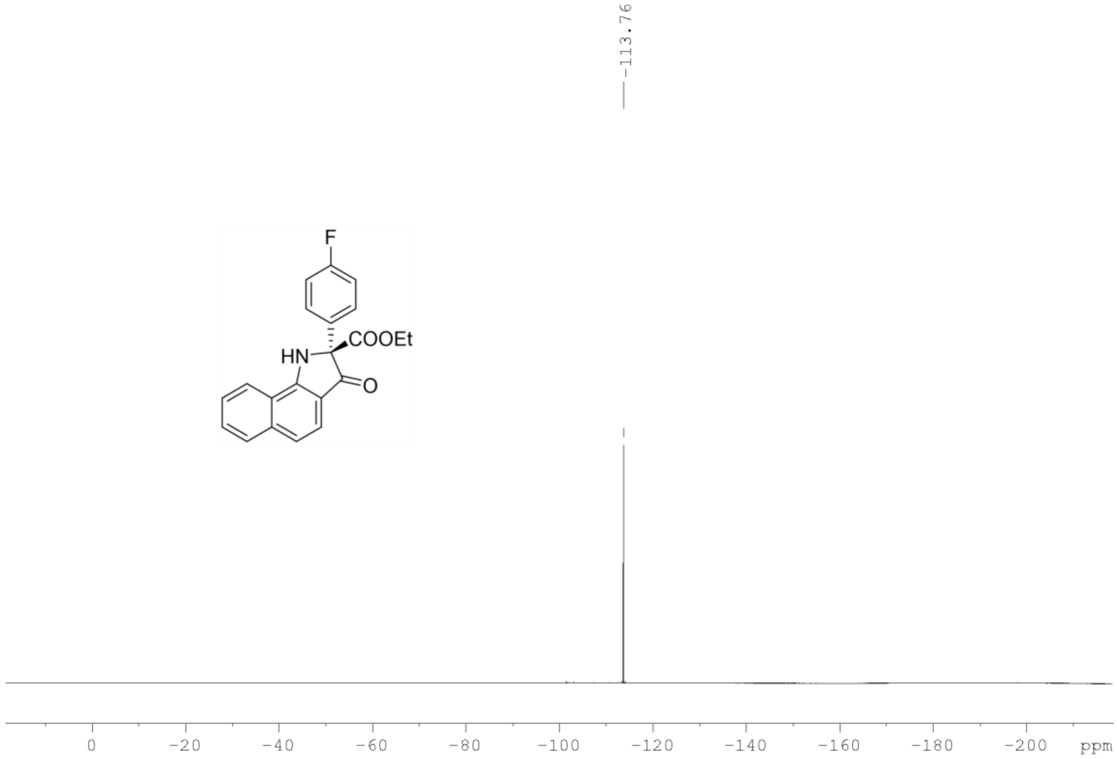

^{19}F NMR (376 MHz, $CDCl_3$) of **4af**

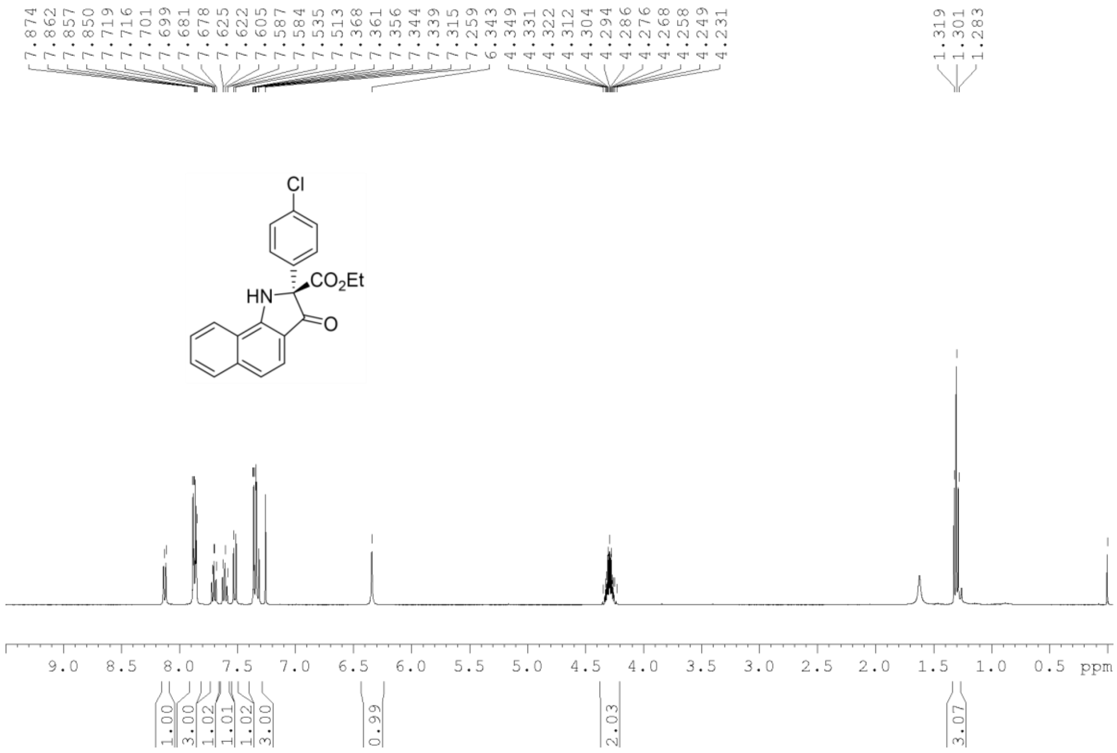

^{1}H NMR (400 MHz, CDCl3) of **4ag**

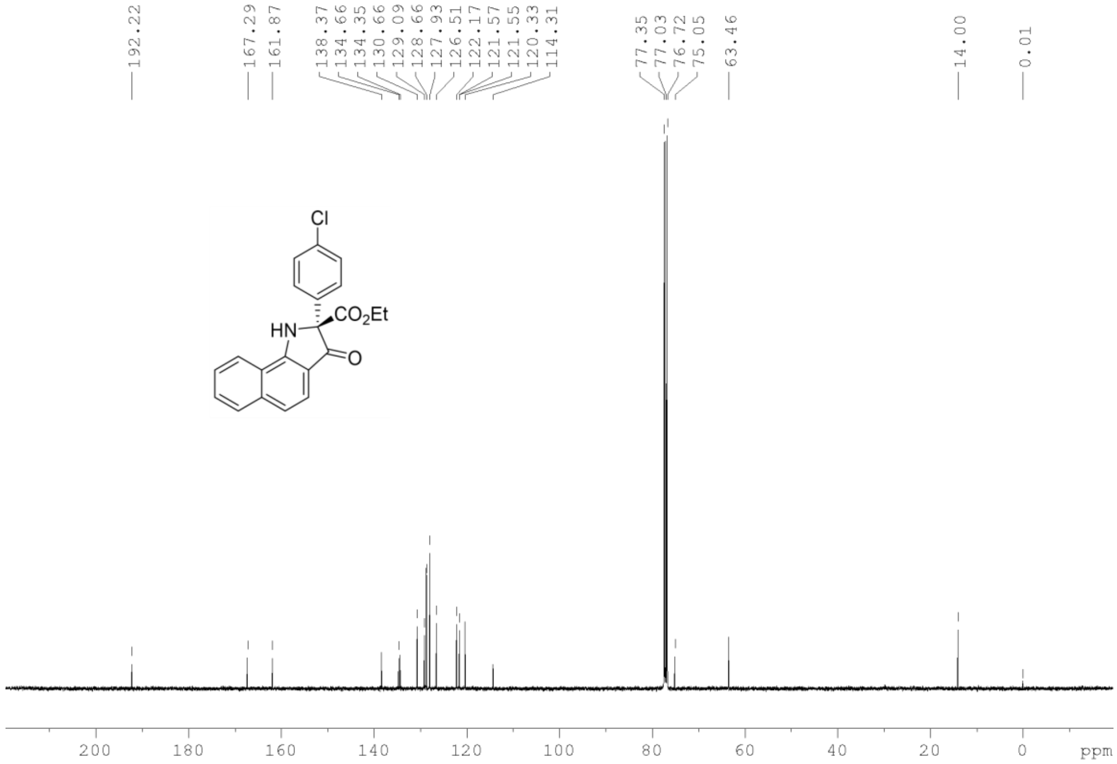

^{13}C NMR (100 MHz, CDCl3) of **4ag**

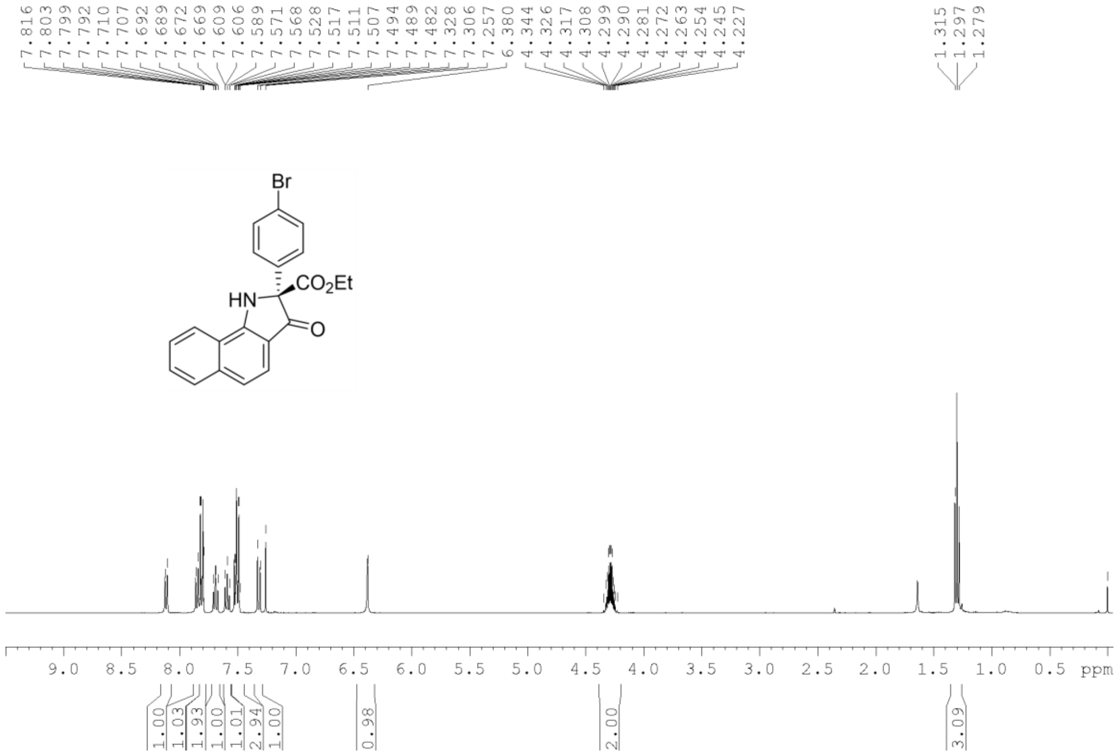

^{1}H NMR (400 MHz, $CDCl_3$) of **4ah**

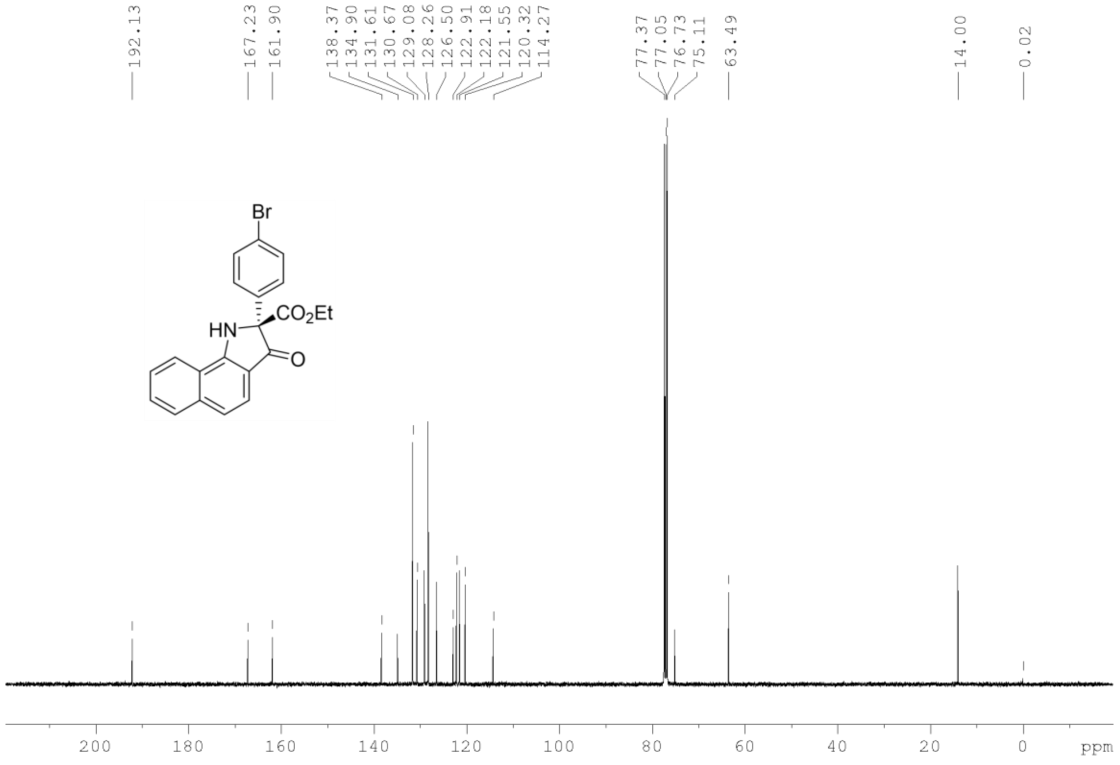

^{13}C NMR (100 MHz, $CDCl_3$) of **4ah**

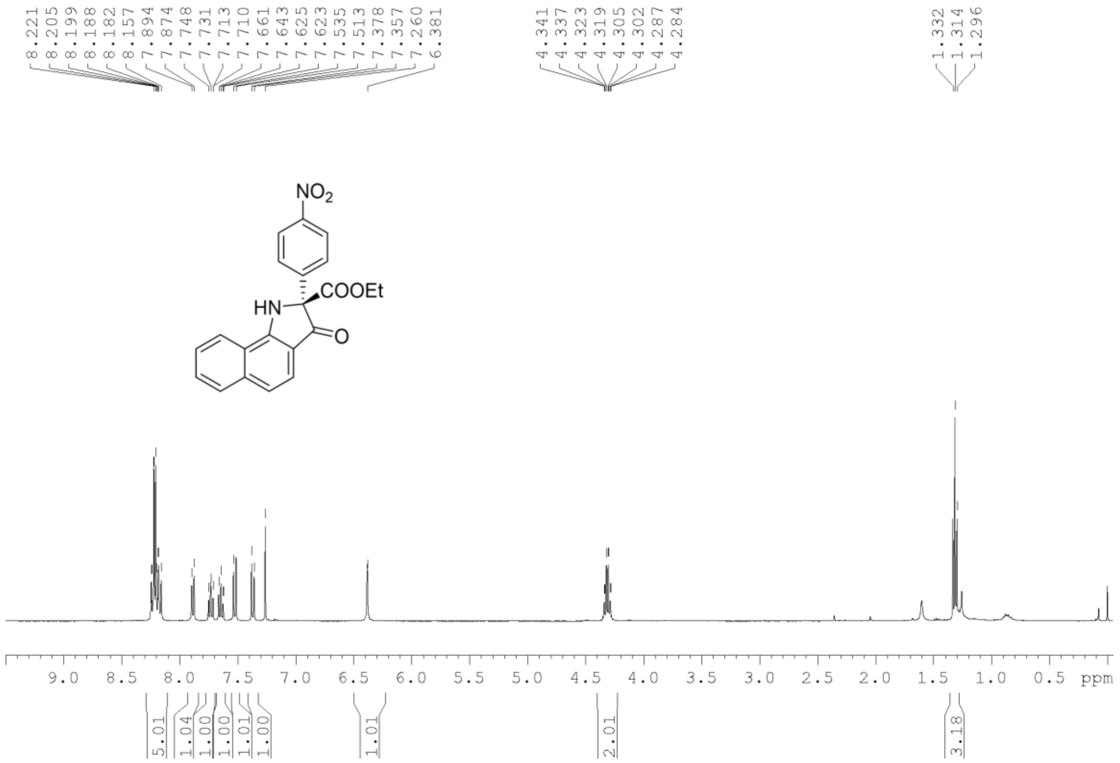

^{1}H NMR (400 MHz, $CDCl_3$) of **4ai**

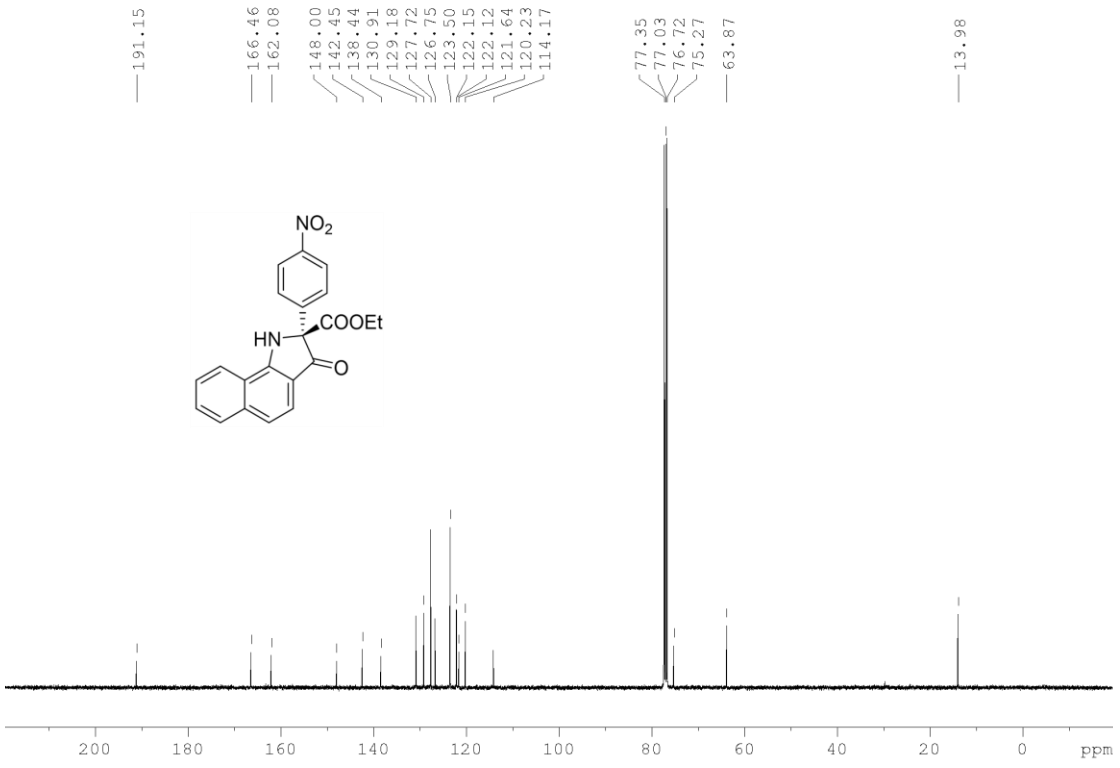

^{13}C NMR (100 MHz, $CDCl_3$) of **4ai**

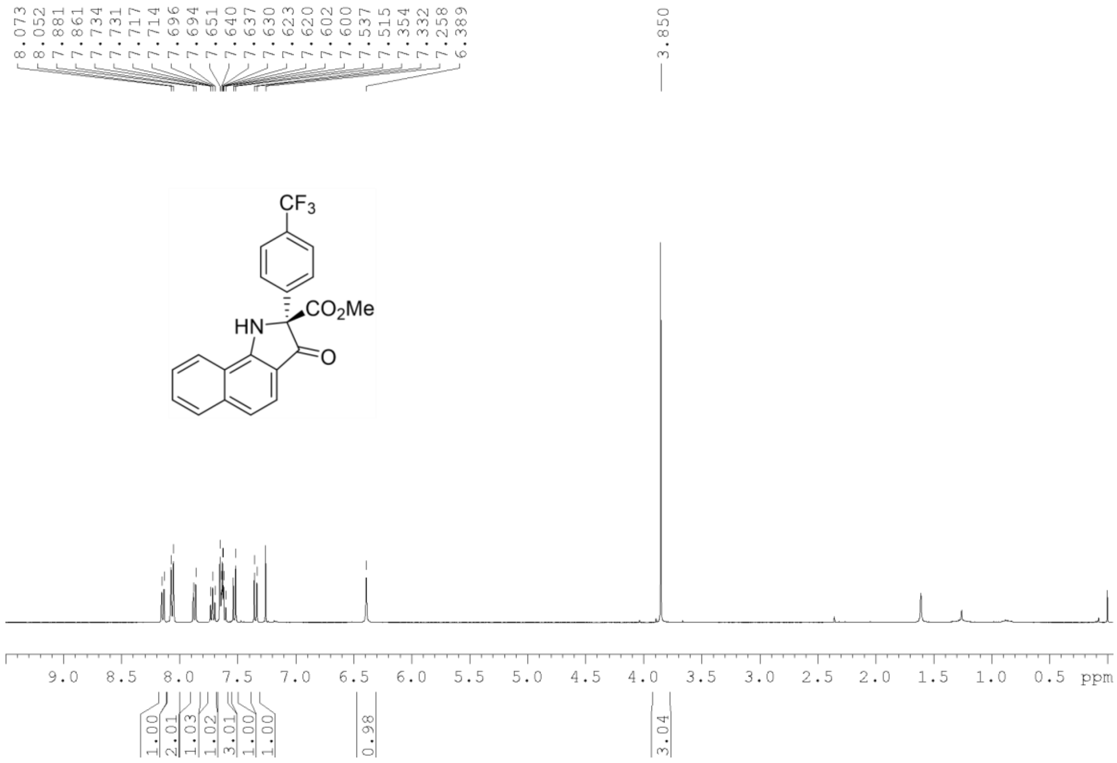

^{1}H NMR (400 MHz, $CDCl_3$) of **4aj**

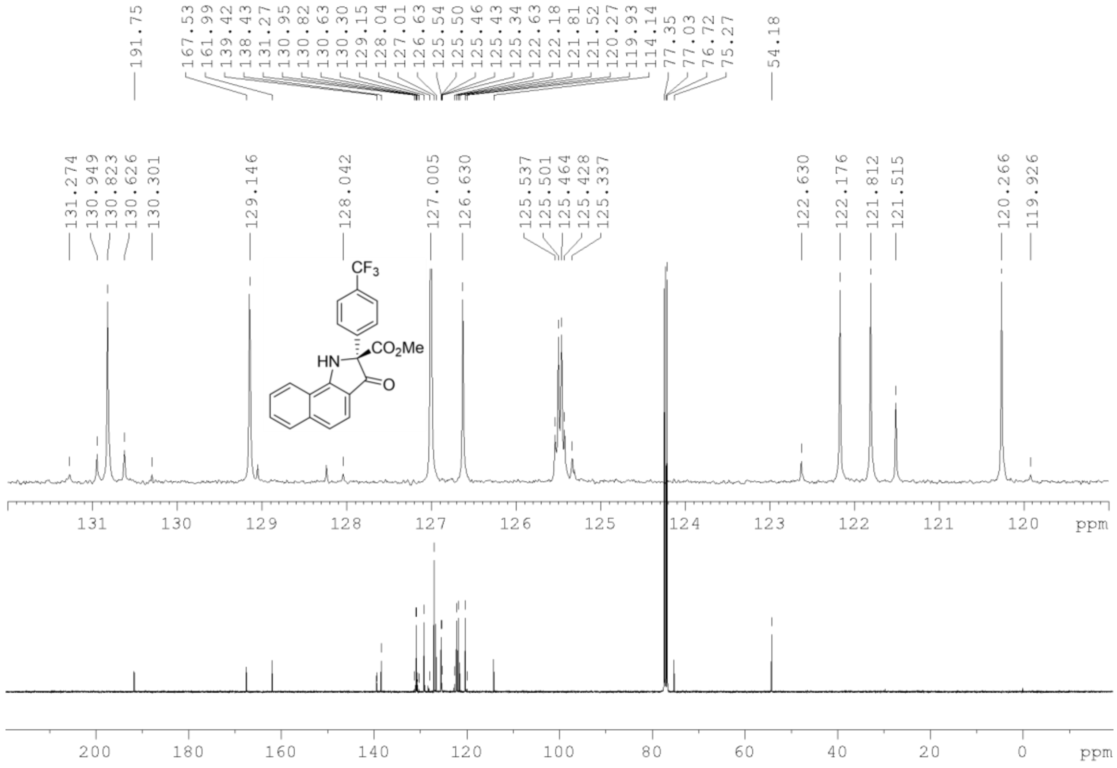

^{13}C NMR (100 MHz, $CDCl_3$) of **4aj**

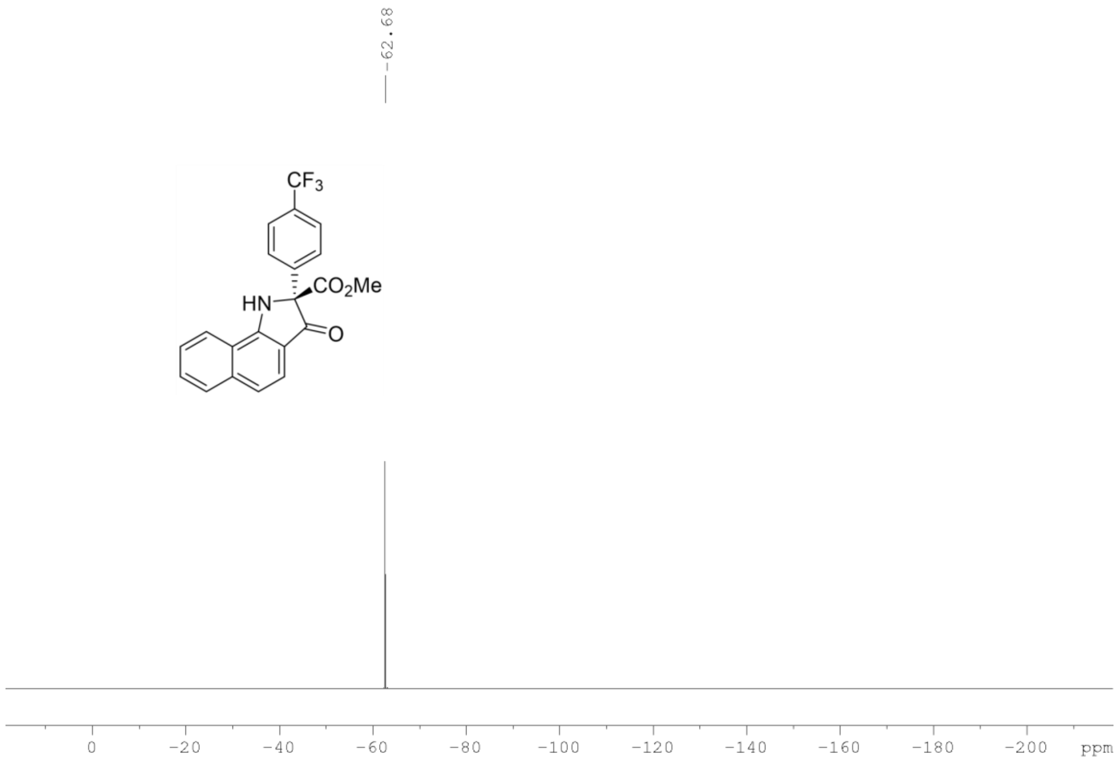

^{19}F NMR (376 MHz, CDCl$_3$) of **4aj**

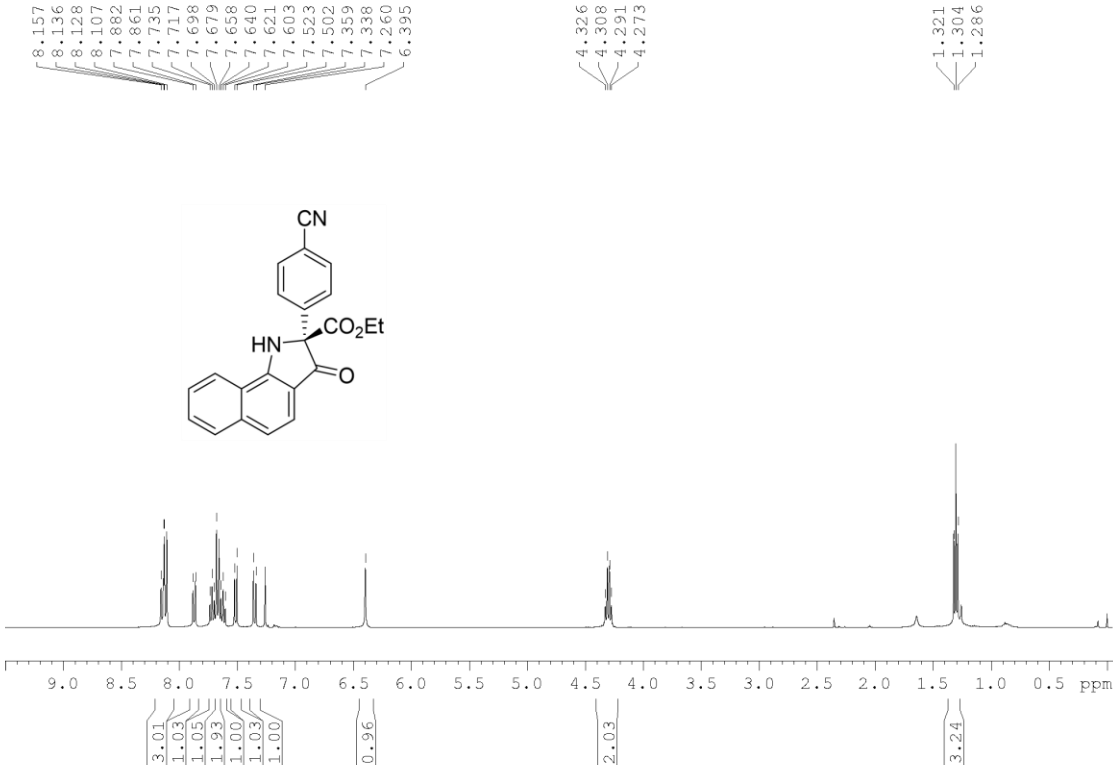

^{1}H NMR (400 MHz, CDCl$_3$) of **4ak**

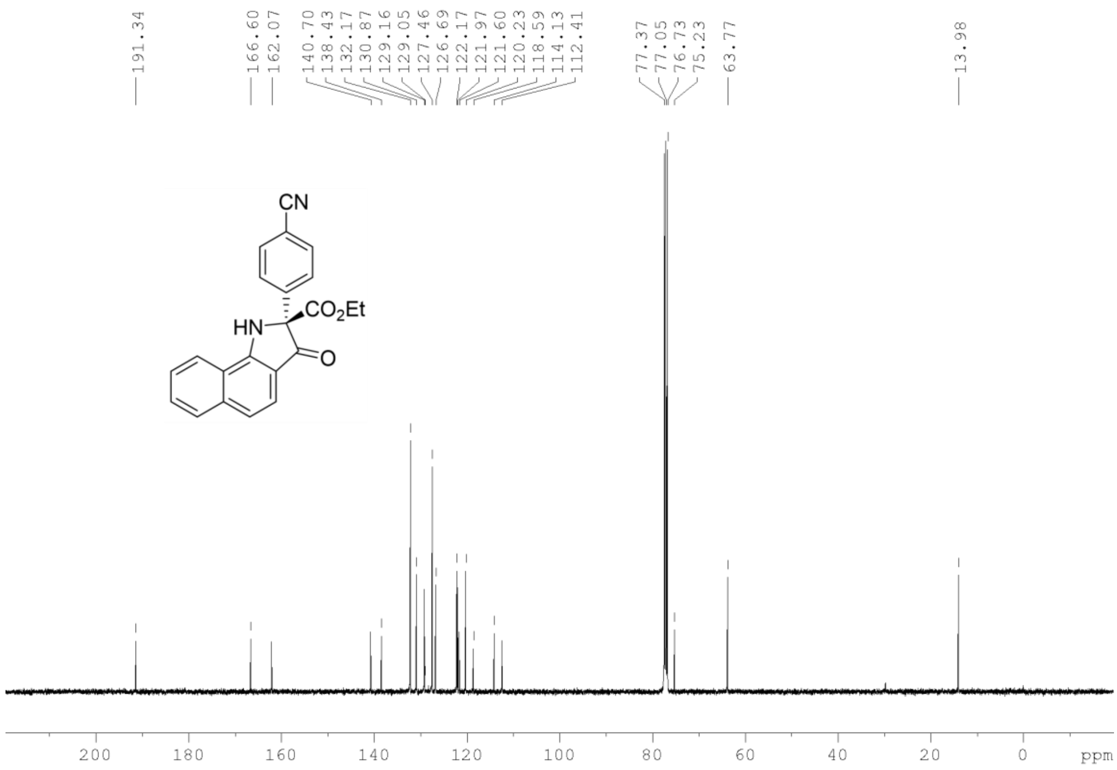

^{13}C NMR (100 MHz, $CDCl_3$) of **4ak**

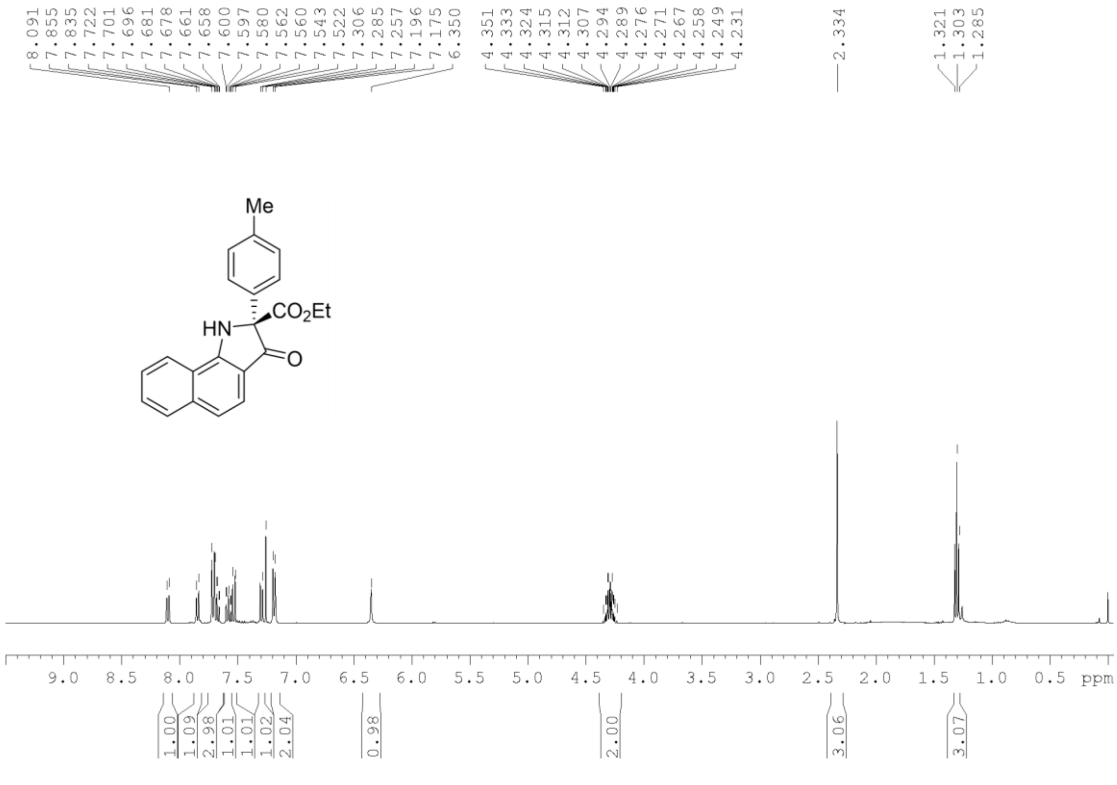

^{1}H NMR (400 MHz, $CDCl_3$) of **4al**

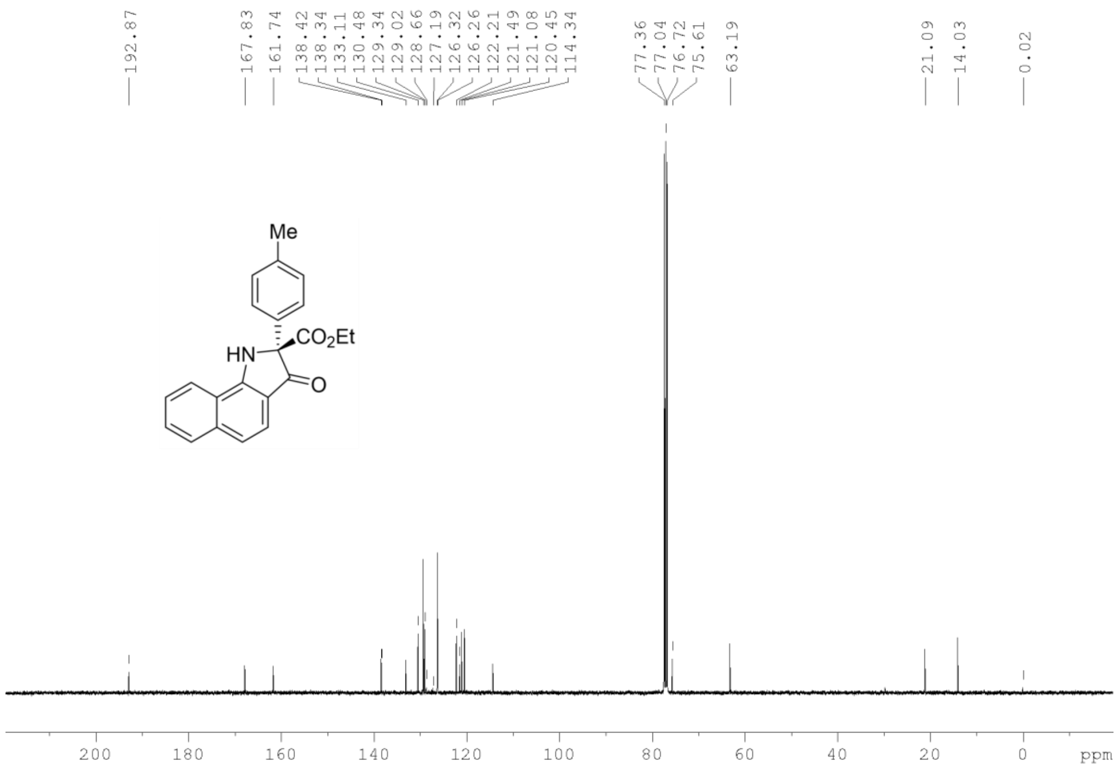

^{13}C NMR (100 MHz, $CDCl_3$) of **4al**

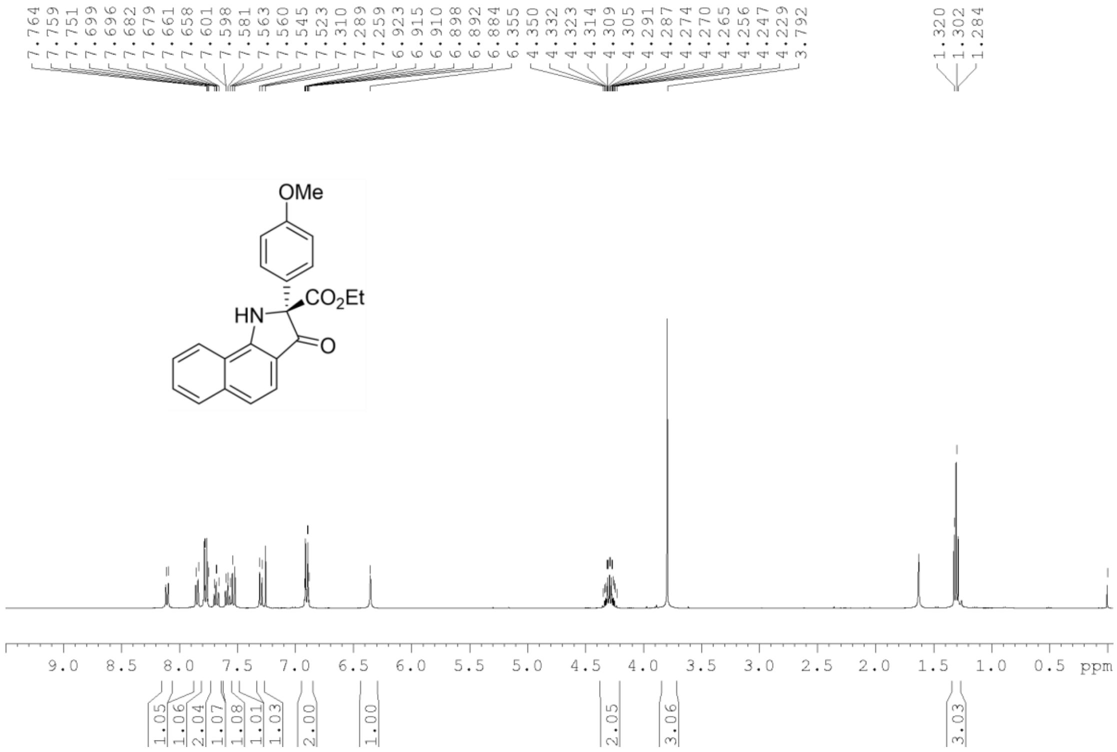

^{1}H NMR (400 MHz, $CDCl_3$) of **4am**

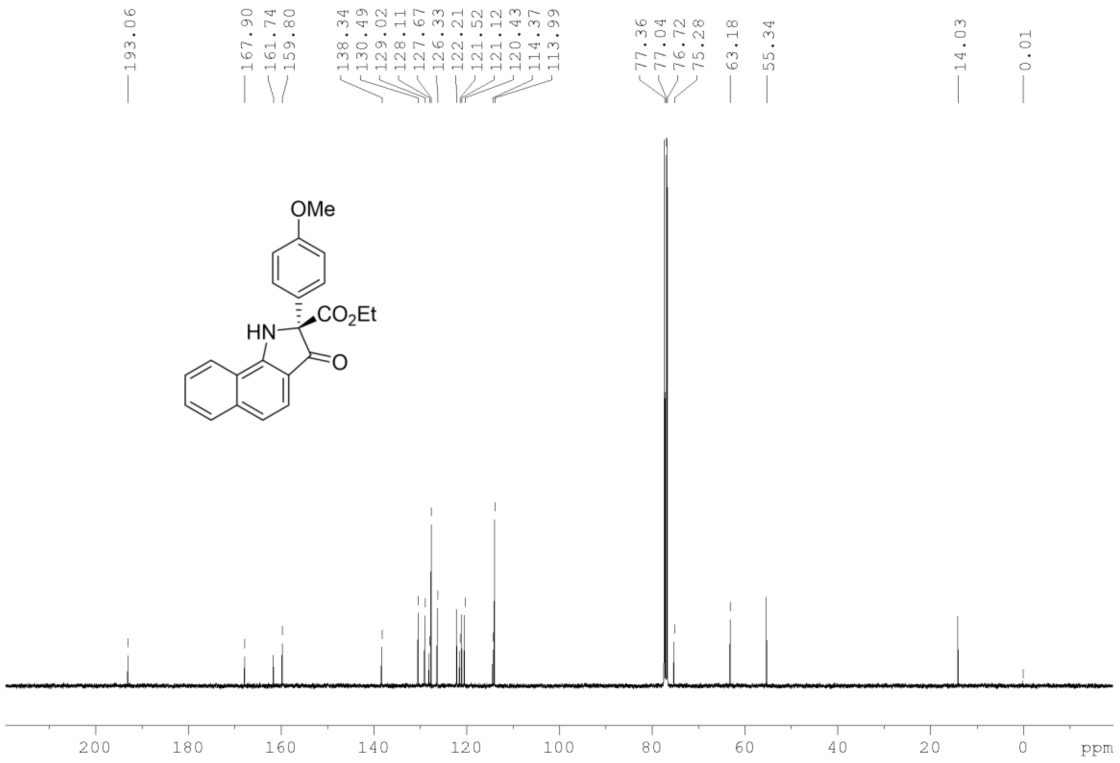

^{13}C NMR (100 MHz, $CDCl_3$) of **4am**

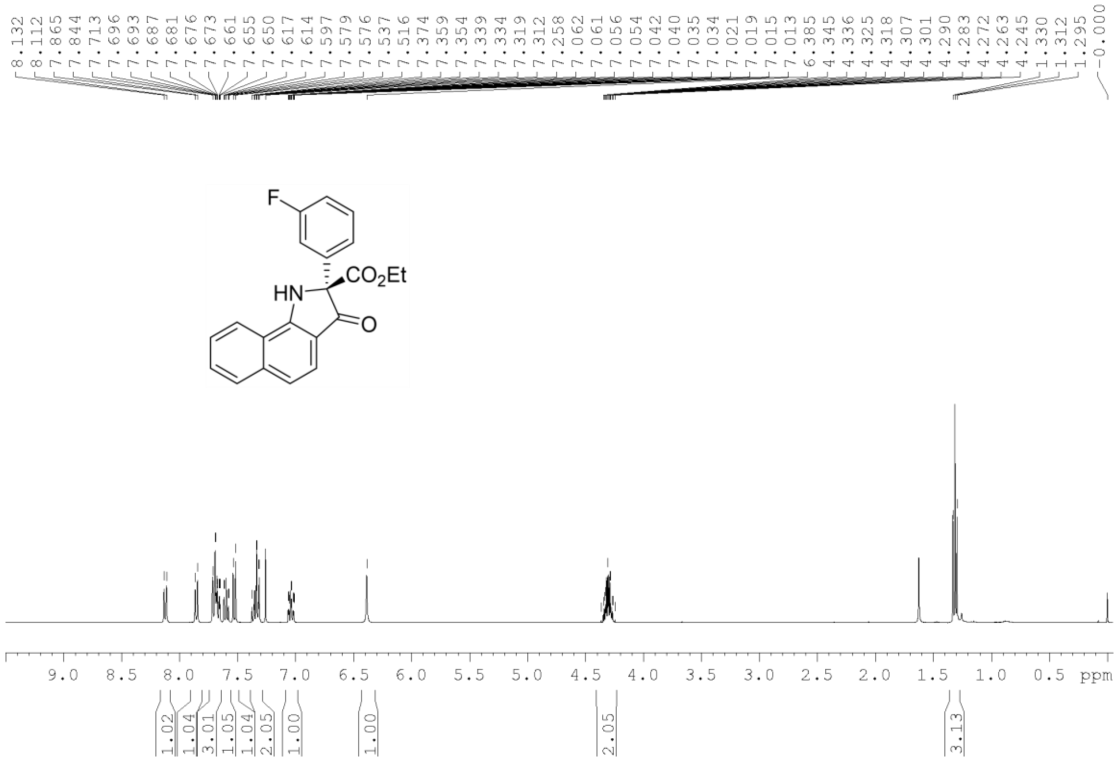

^{1}H NMR (400 MHz, $CDCl_3$) of **4an**

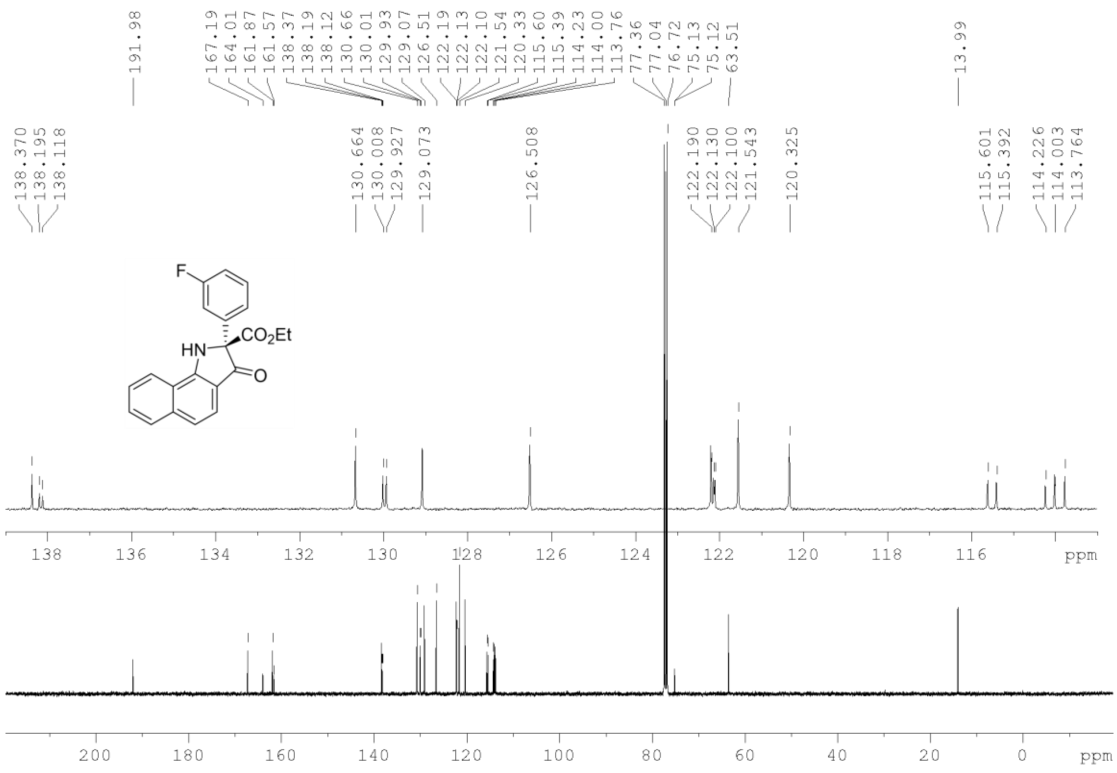

^{13}C NMR (100 MHz, $CDCl_3$) of **4an**

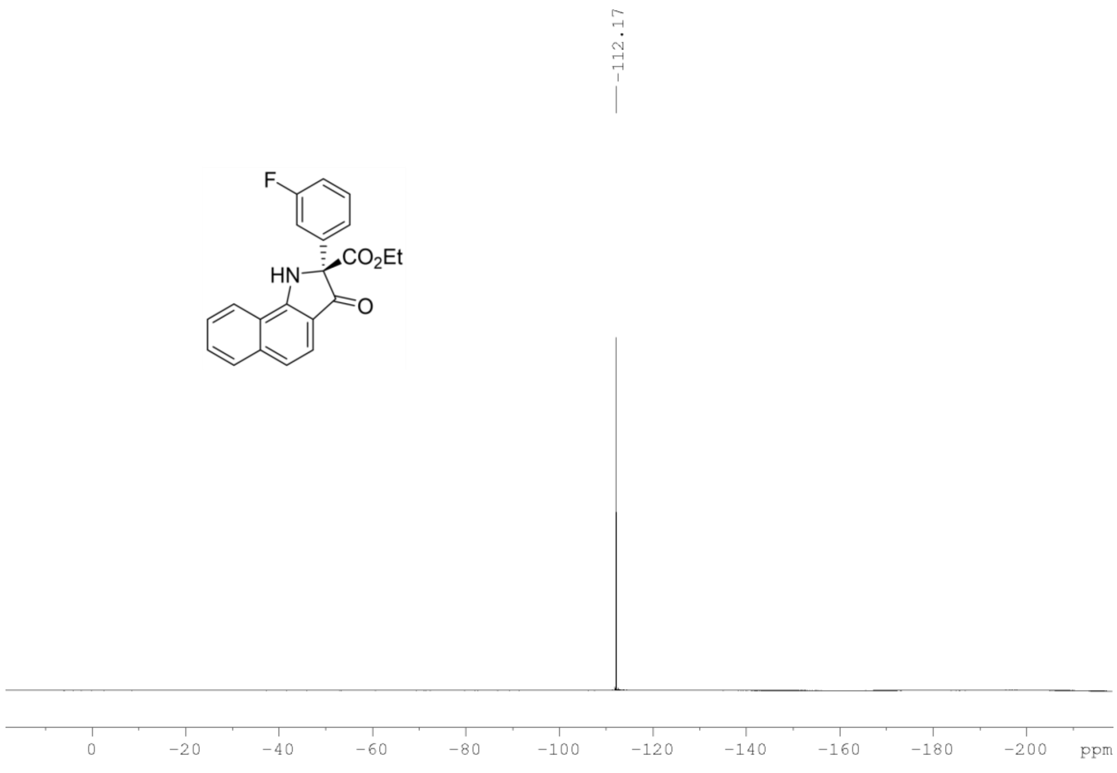

^{19}F NMR (376 MHz, $CDCl_3$) of **4an**

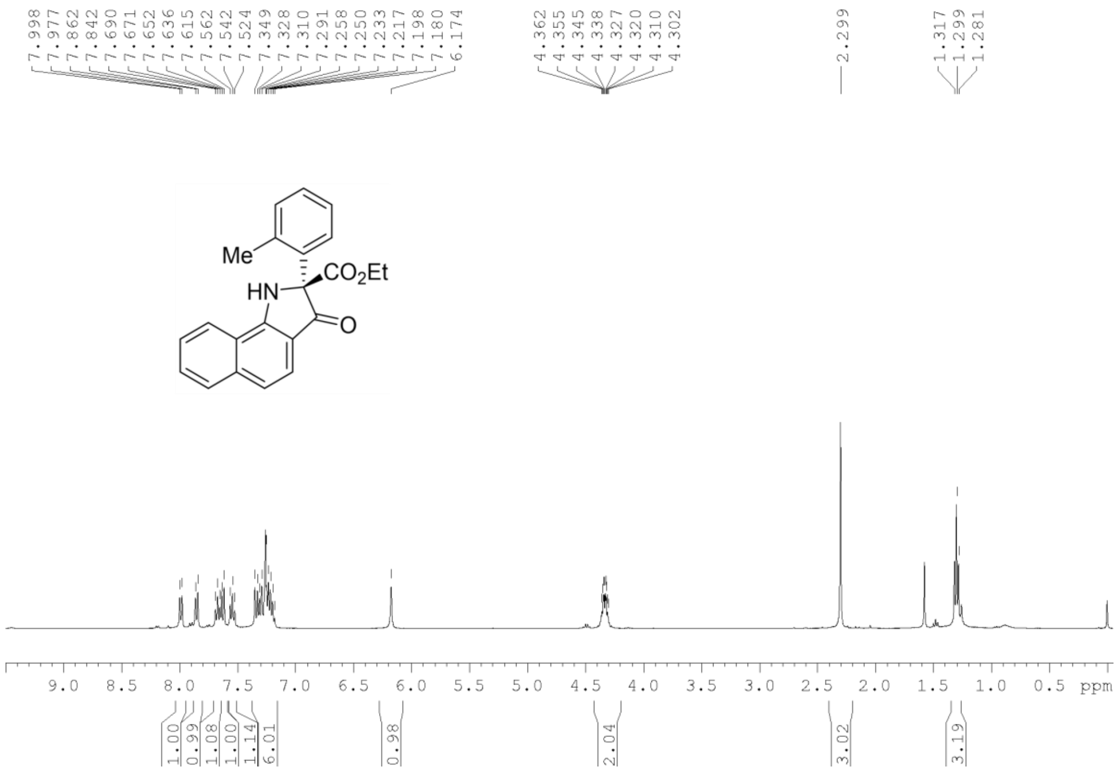

^{1}H NMR (400 MHz, $CDCl_3$) of **4ao**

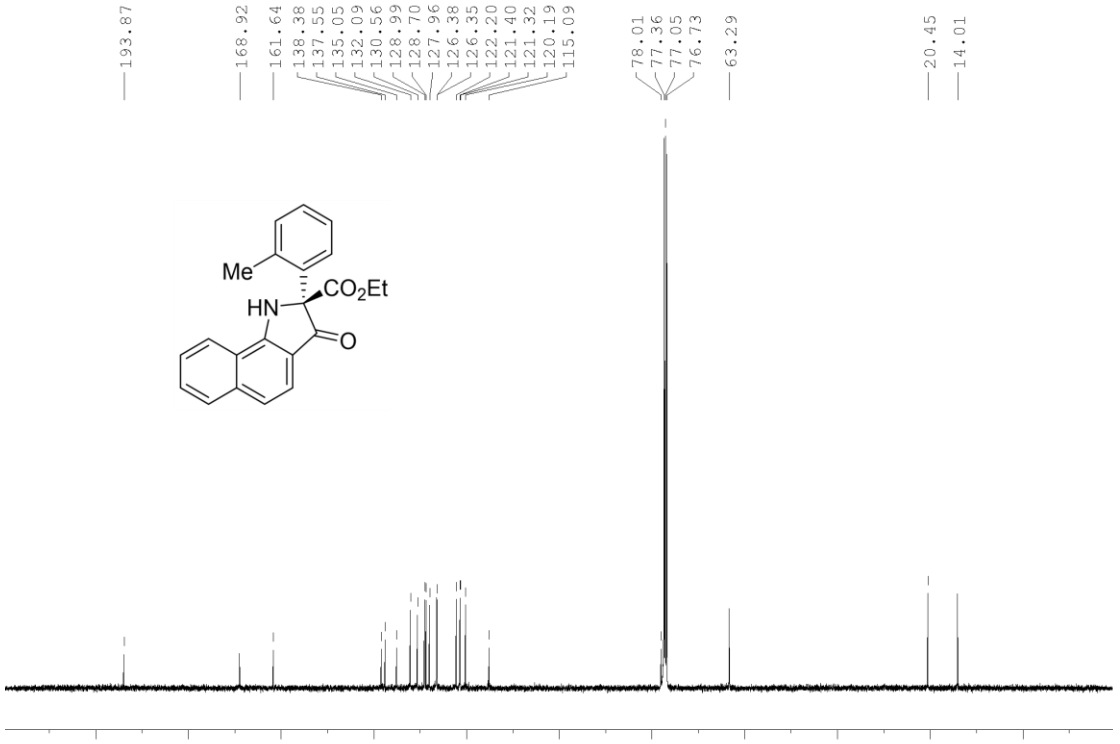

^{13}C NMR (100 MHz, $CDCl_3$) of **4ao**

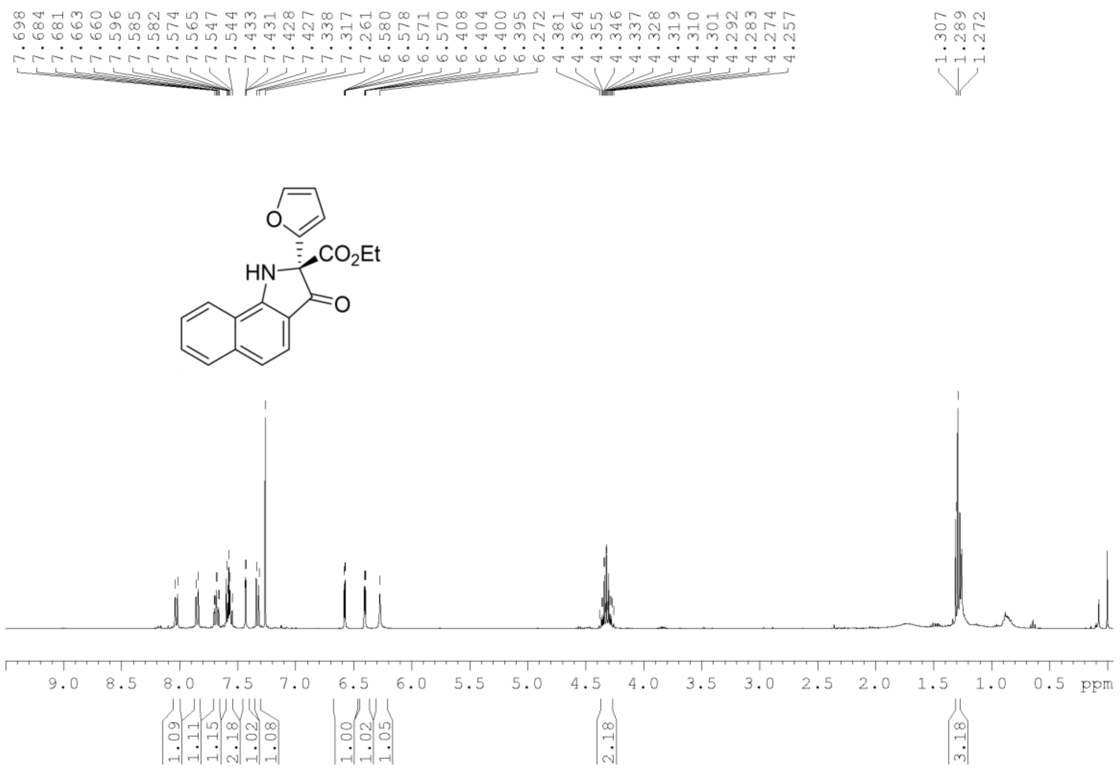

^{1}H NMR (400 MHz, CDCl3) of **4ap**

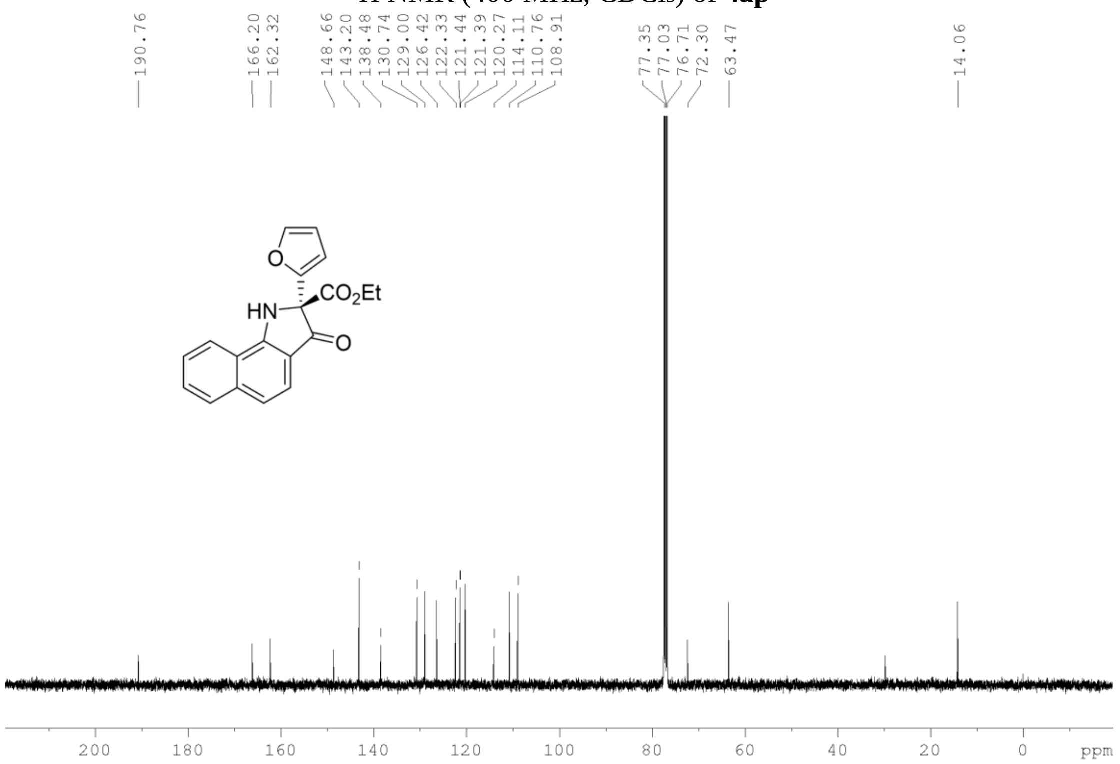

^{13}C NMR (100 MHz, CDCl3) of **4ap**

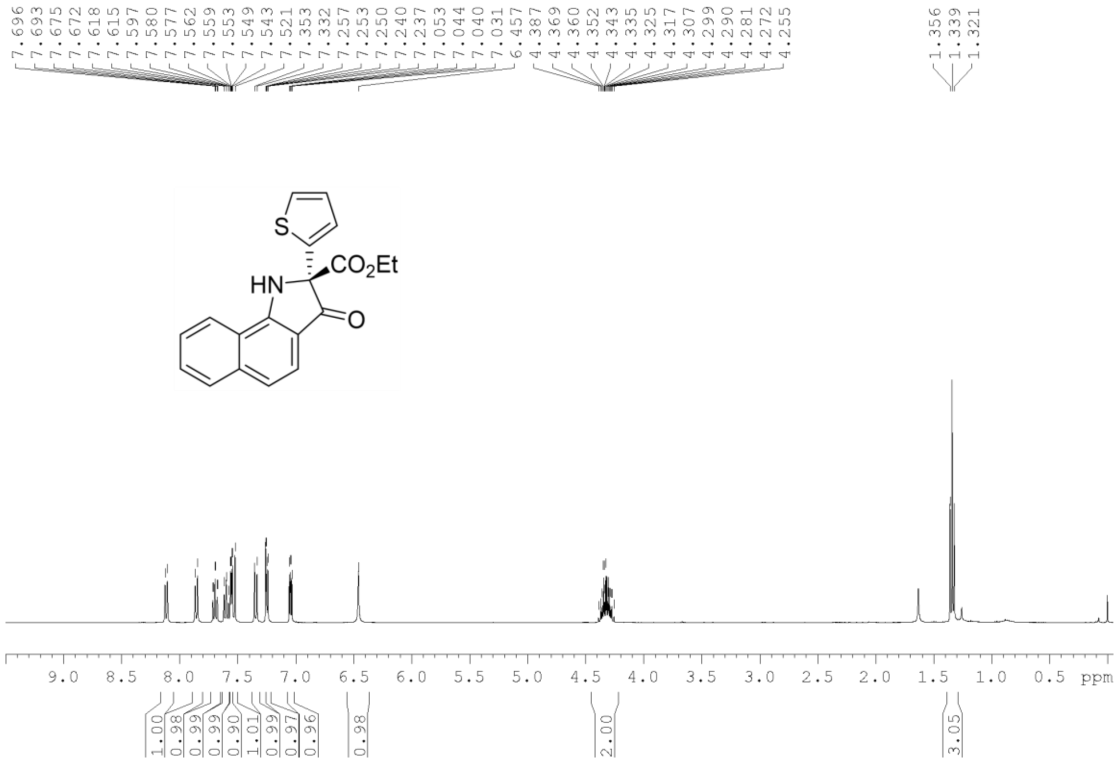

^{1}H NMR (400 MHz, $CDCl_3$) of **4aq**

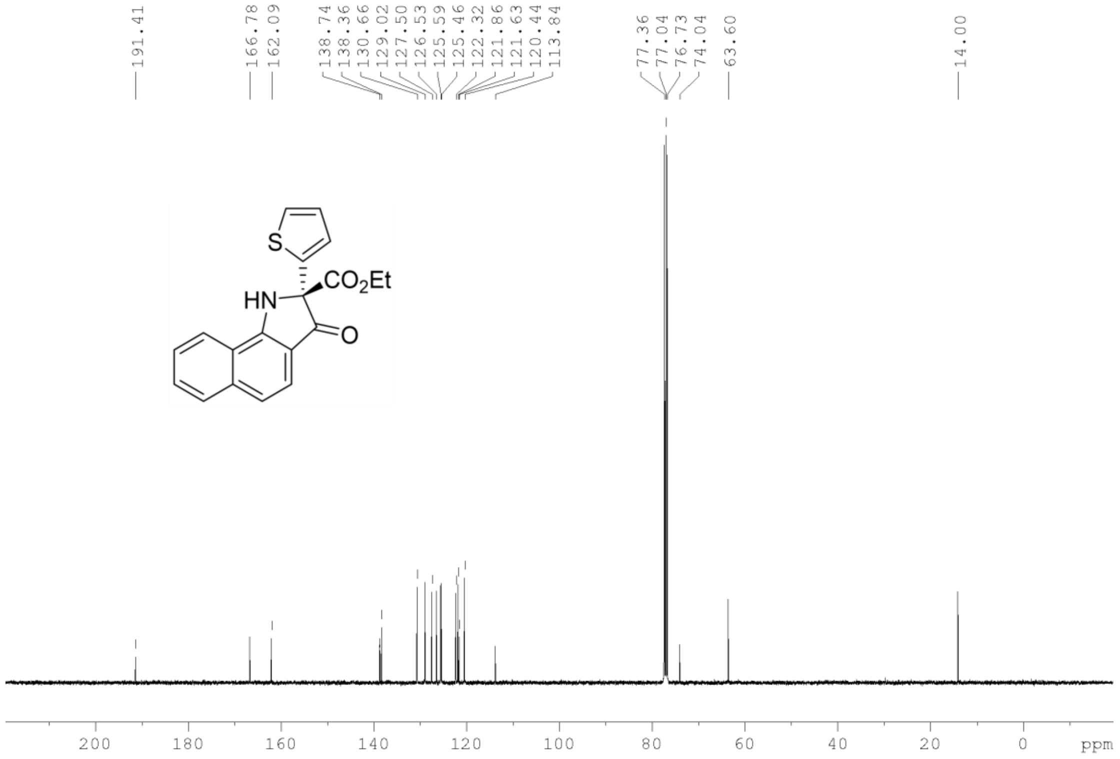

^{13}C NMR (100 MHz, $CDCl_3$) of **4aq**

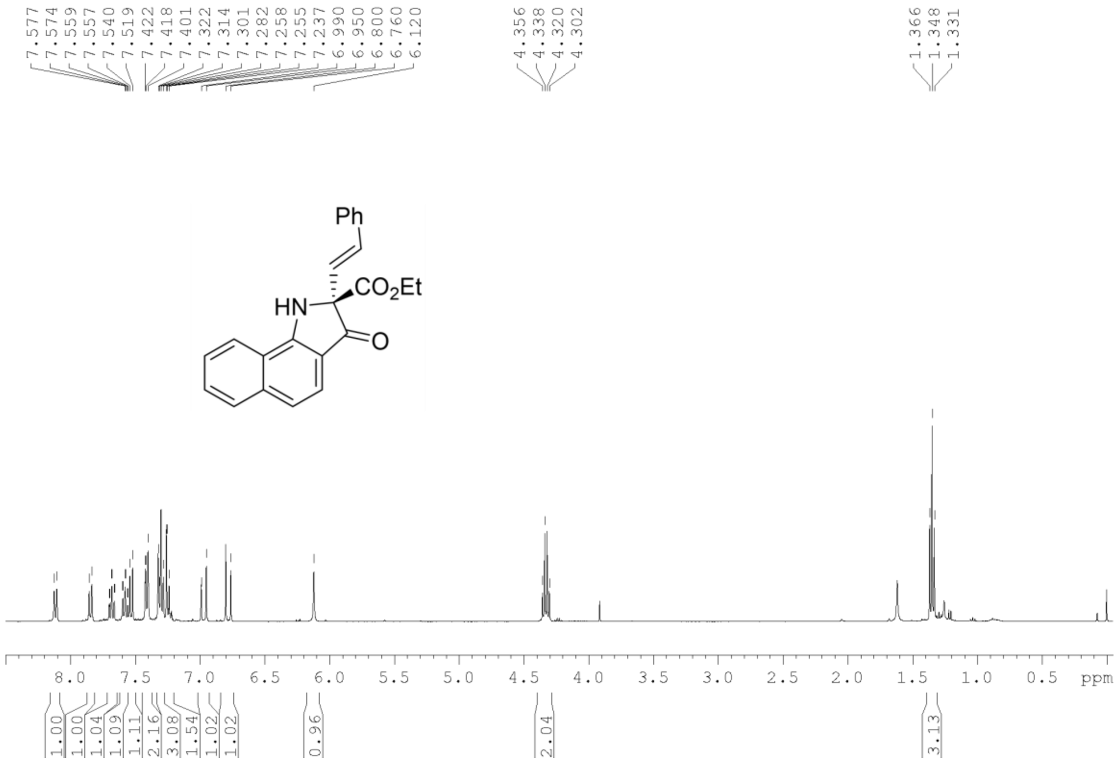

^{1}H NMR (400 MHz, $CDCl_3$) of **4ar**

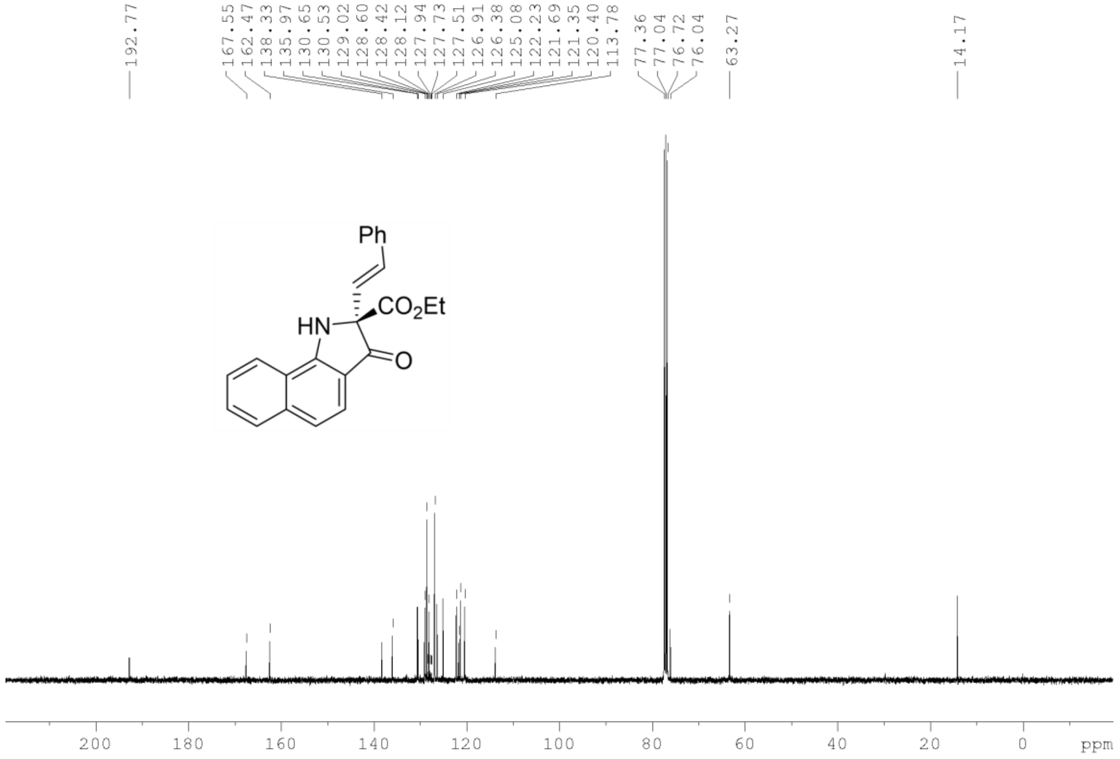

^{13}C NMR (100 MHz, $CDCl_3$) of **4ar**

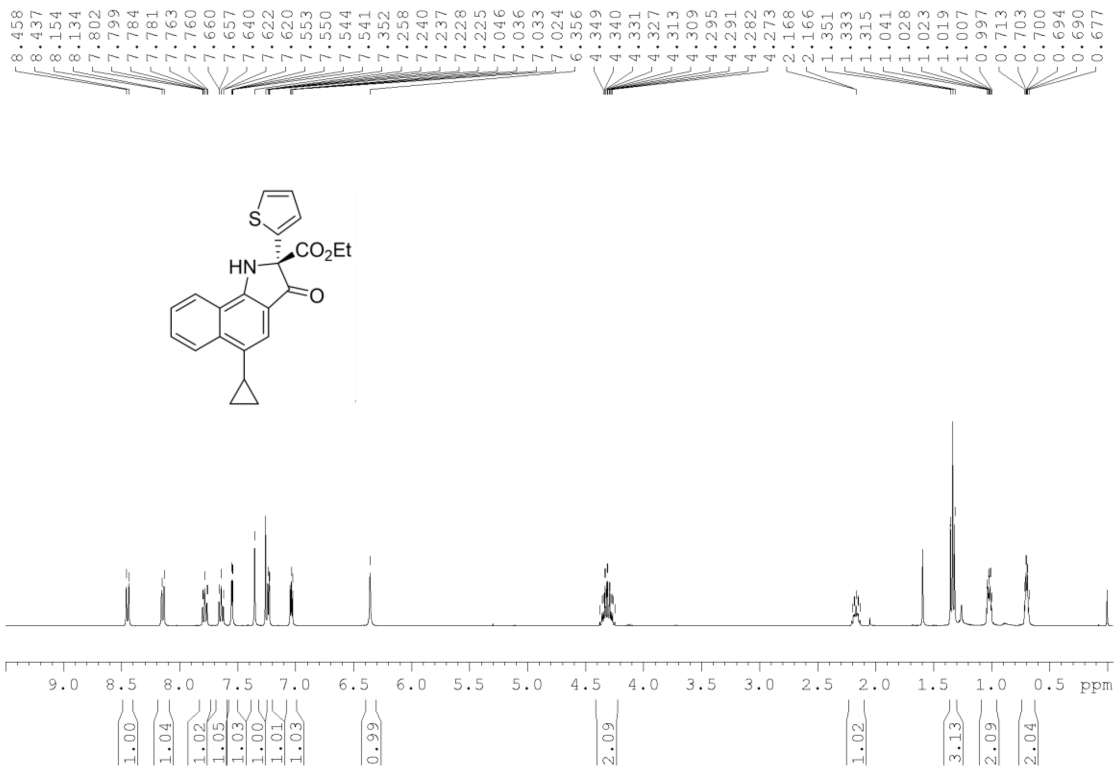

^{1}H NMR (400 MHz, $CDCl_3$) of **4bq**

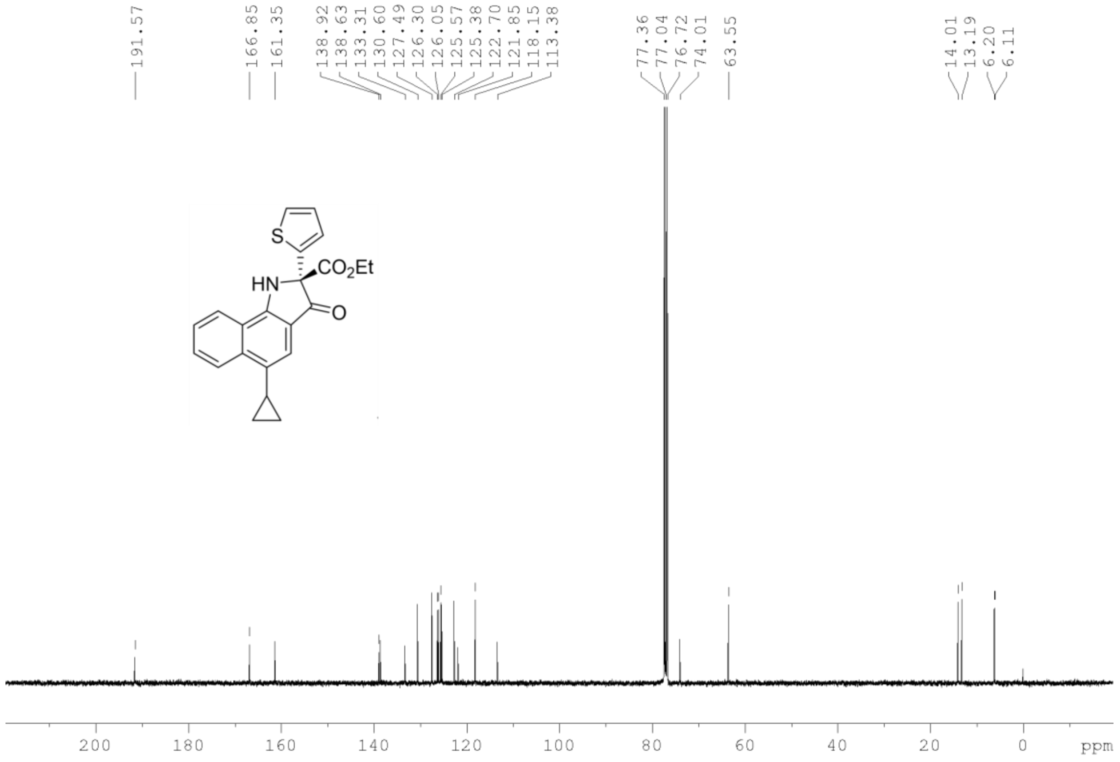

^{13}C NMR (100 MHz, $CDCl_3$) of **4bq**

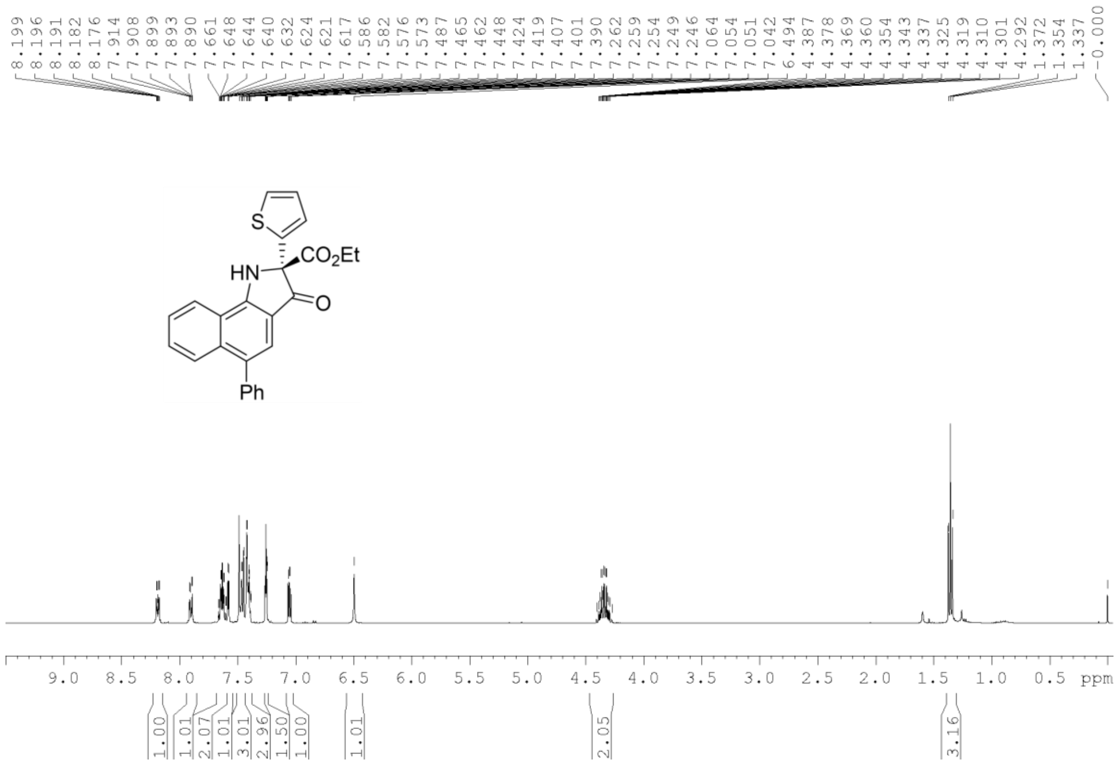

^{1}H NMR (400 MHz, $CDCl_3$) of **4cq**

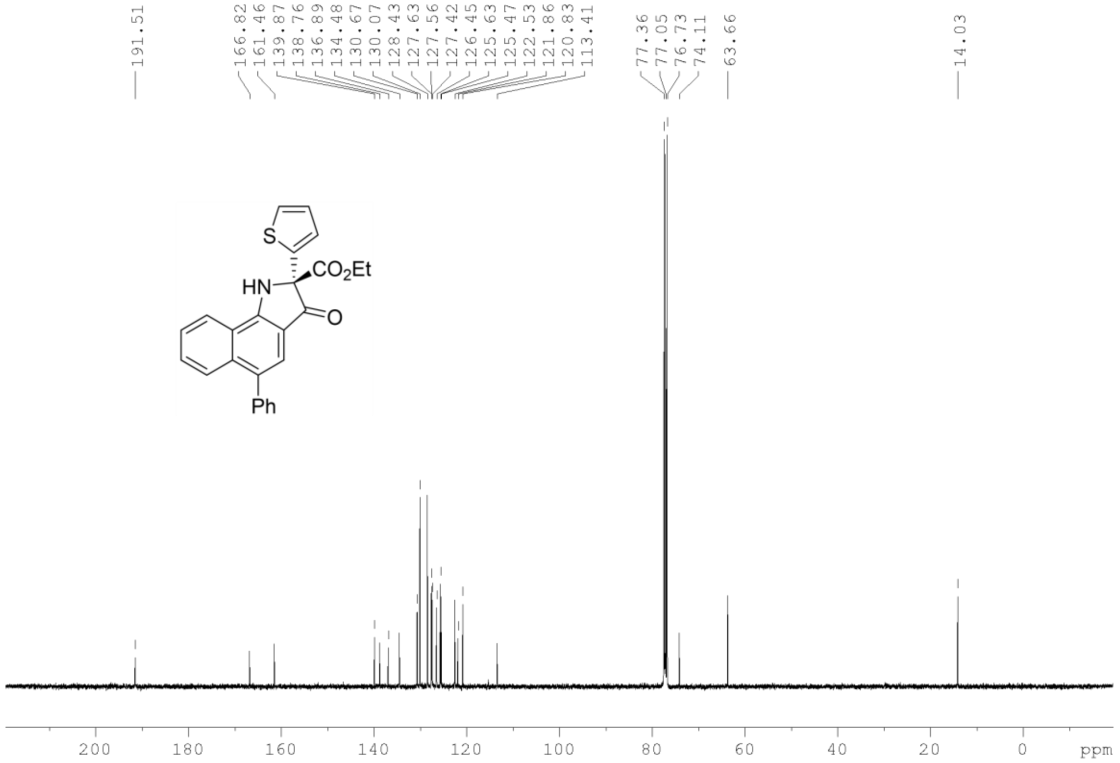

^{13}C NMR (100 MHz, $CDCl_3$) of **4cq**

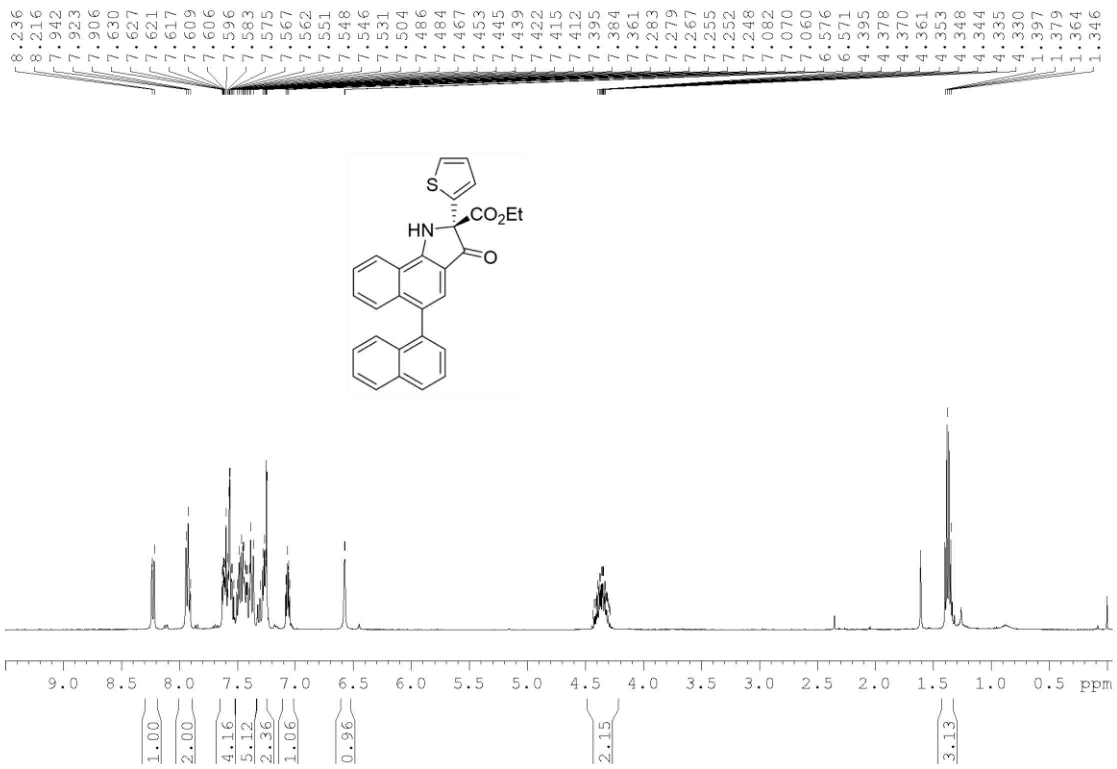

^{1}H NMR (400 MHz, $CDCl_3$) of **4dq**

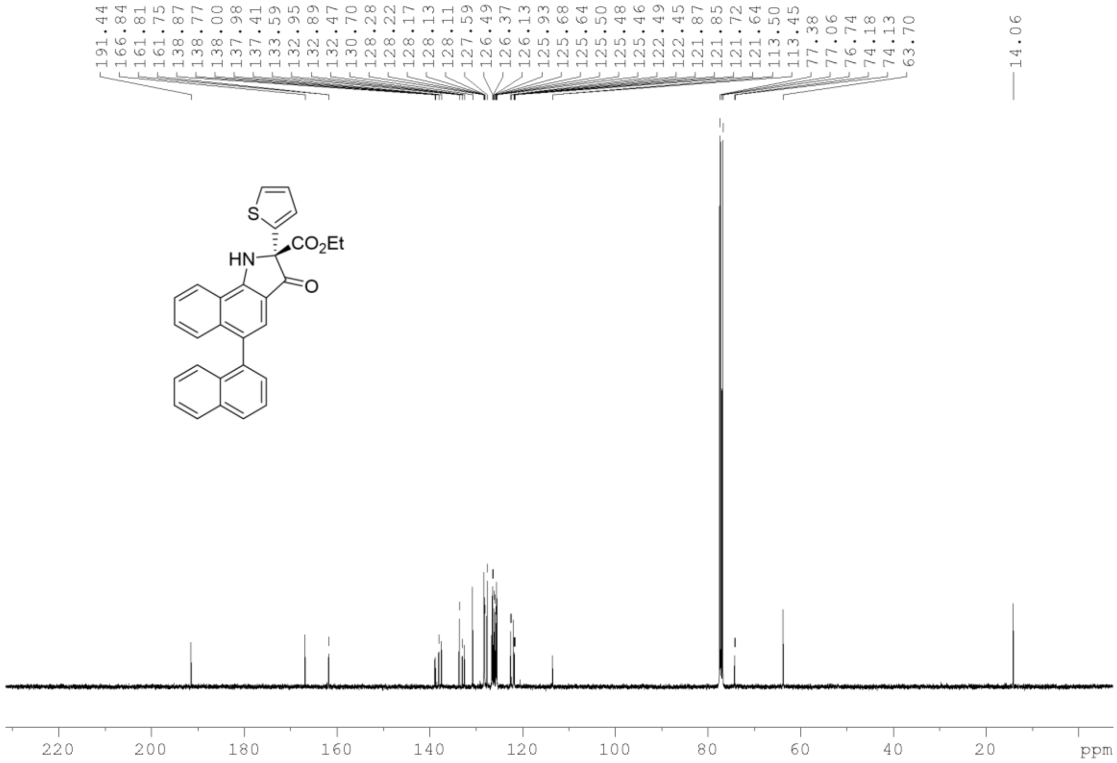

^{13}C NMR (100 MHz, $CDCl_3$) of **4dq**

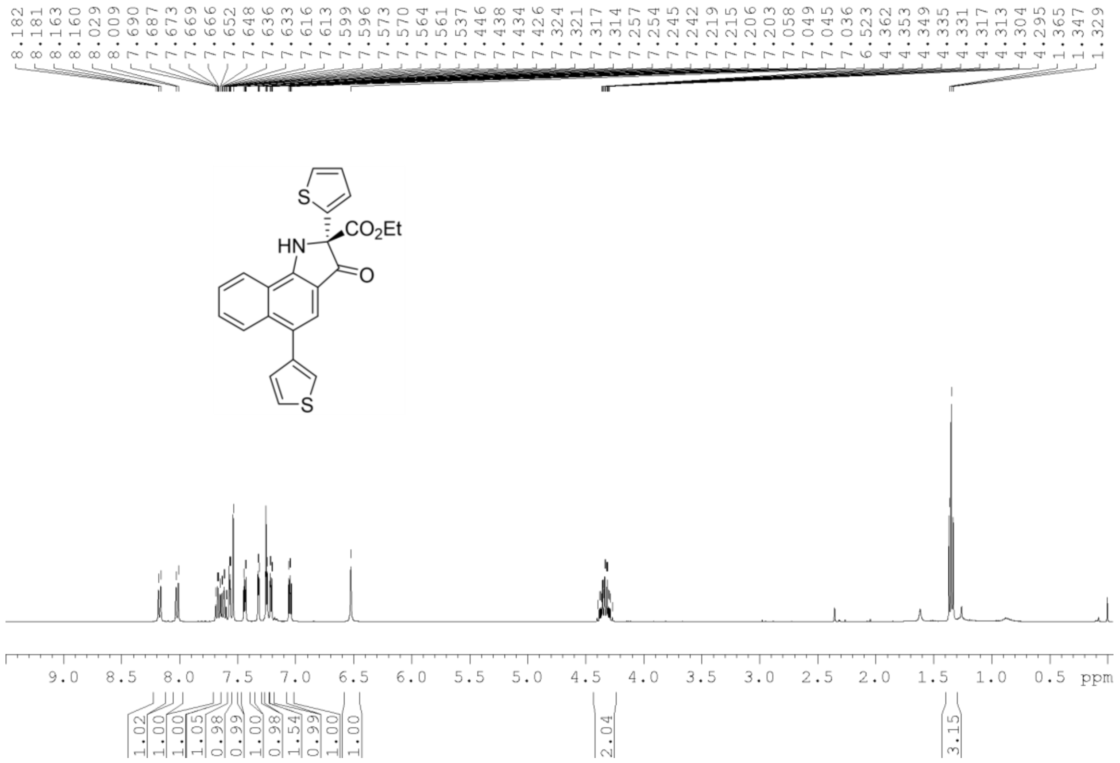

^{1}H NMR (400 MHz, $CDCl_3$) of **4eq**

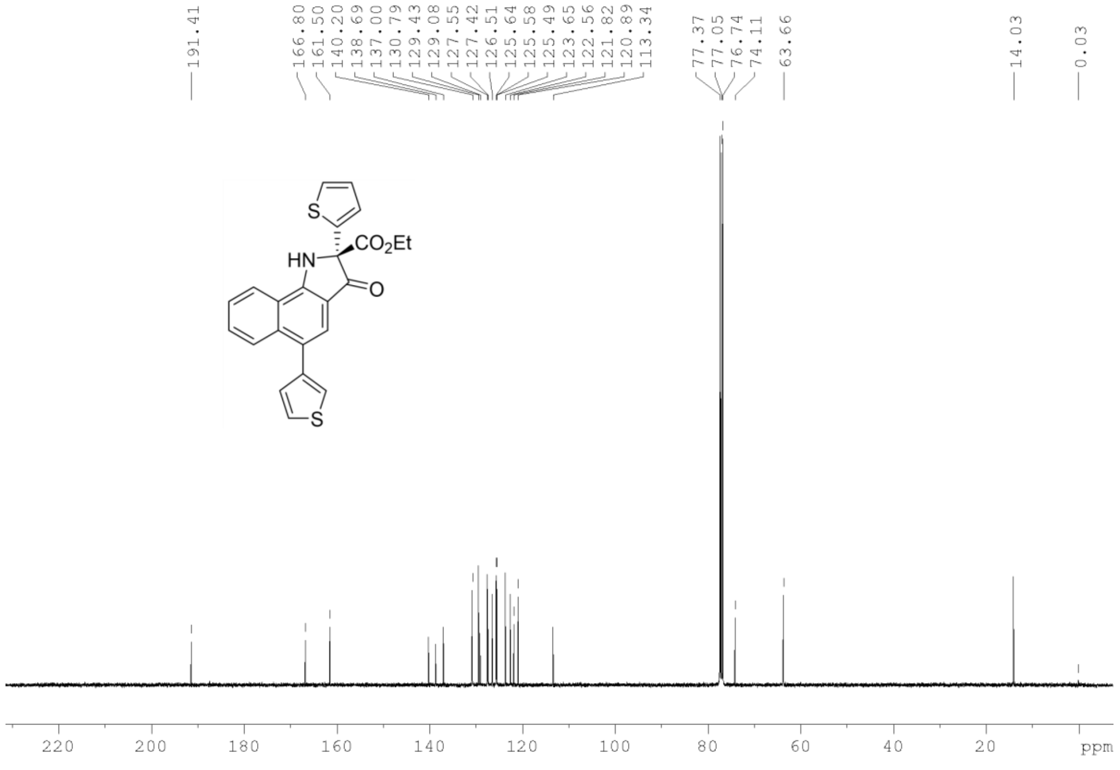

^{13}C NMR (100 MHz, $CDCl_3$) of **4eq**

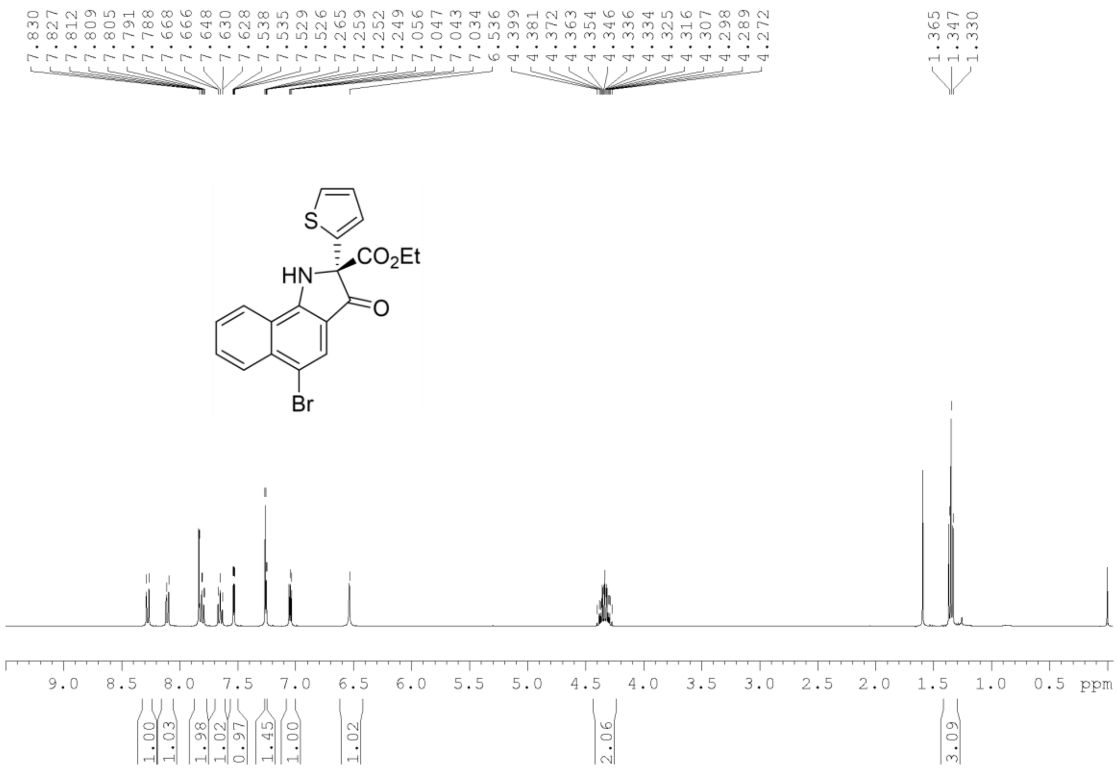

^{1}H NMR (400 MHz, $CDCl_3$) of **4fq**

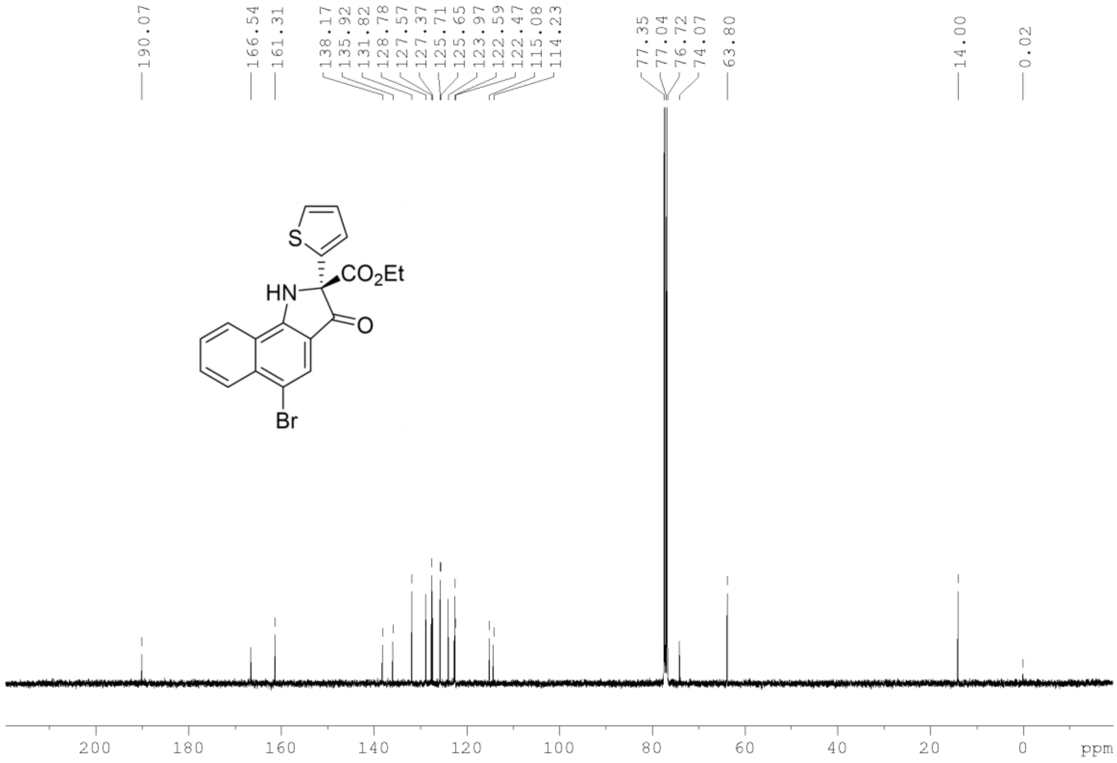

^{13}C NMR (100 MHz, $CDCl_3$) of **4fq**

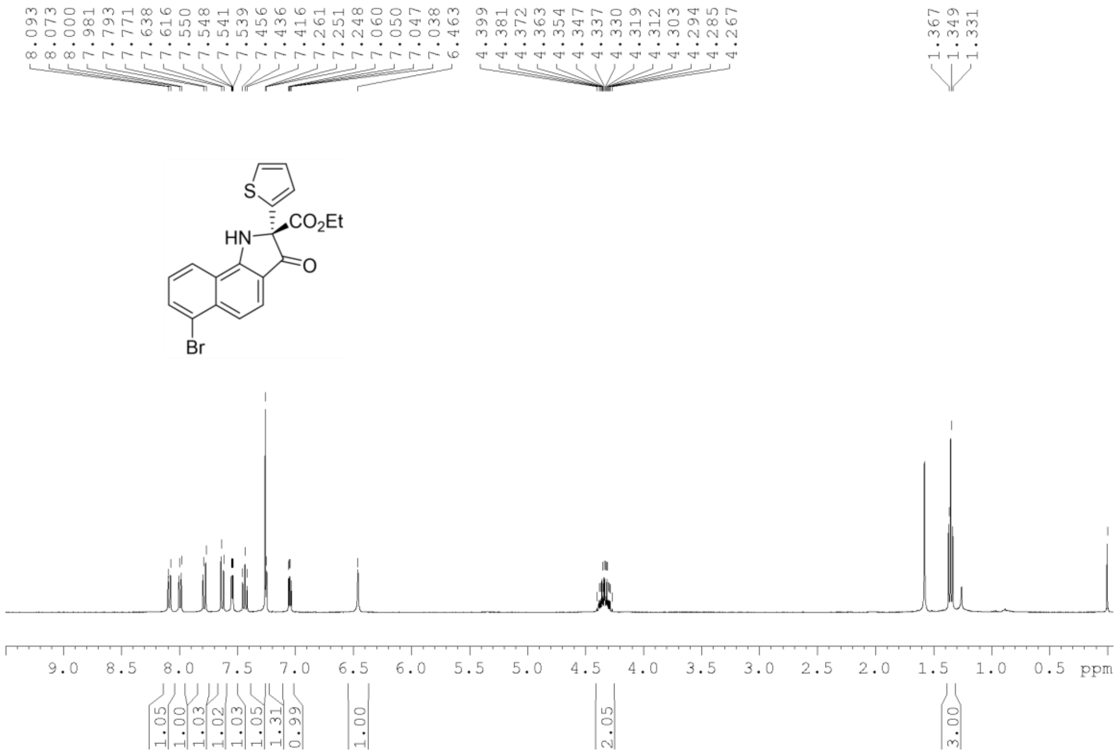

^{1}H NMR (400 MHz, CDCl3) of **4gq**

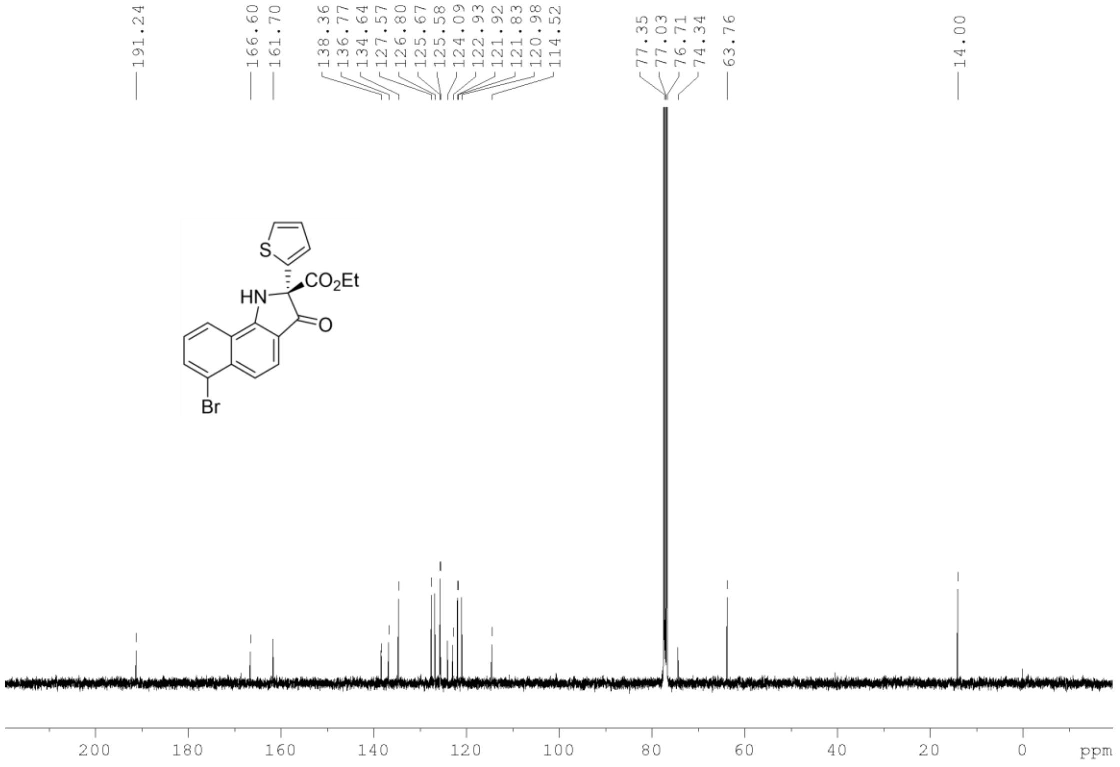

^{13}C NMR (100 MHz, CDCl3) of **4gq**

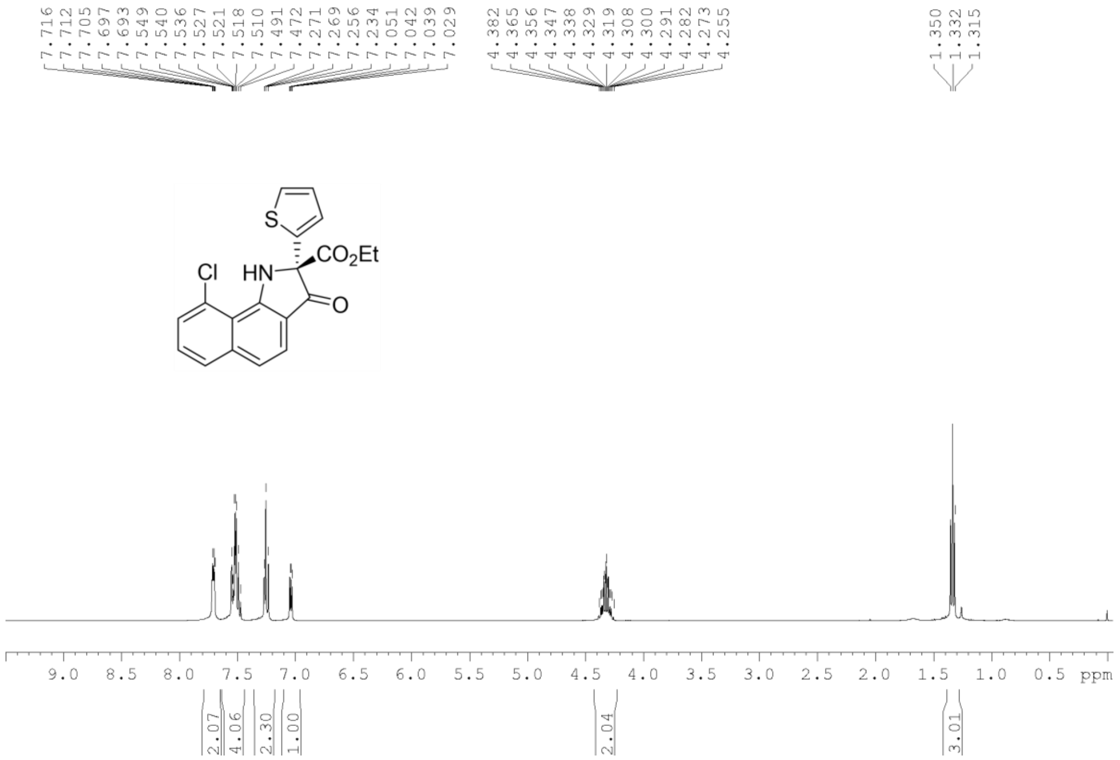

^{1}H NMR (400 MHz, $CDCl_3$) of **4hq**

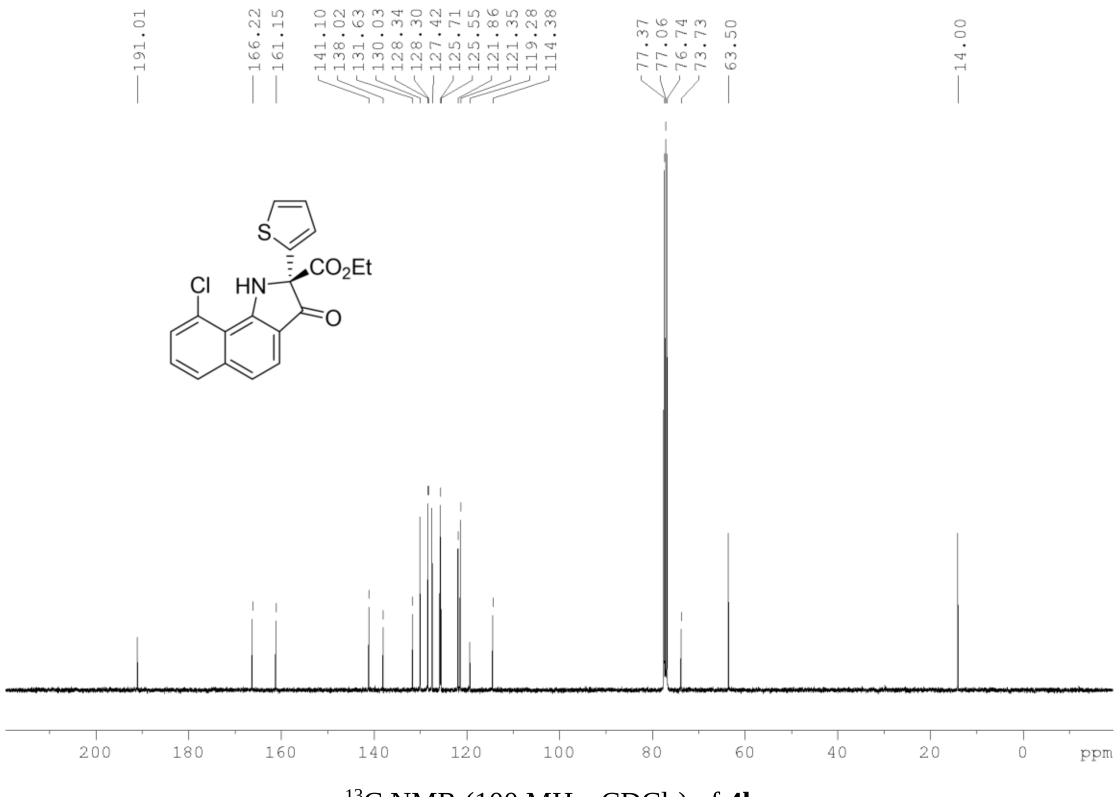

^{13}C NMR (100 MHz, $CDCl_3$) of **4hq**

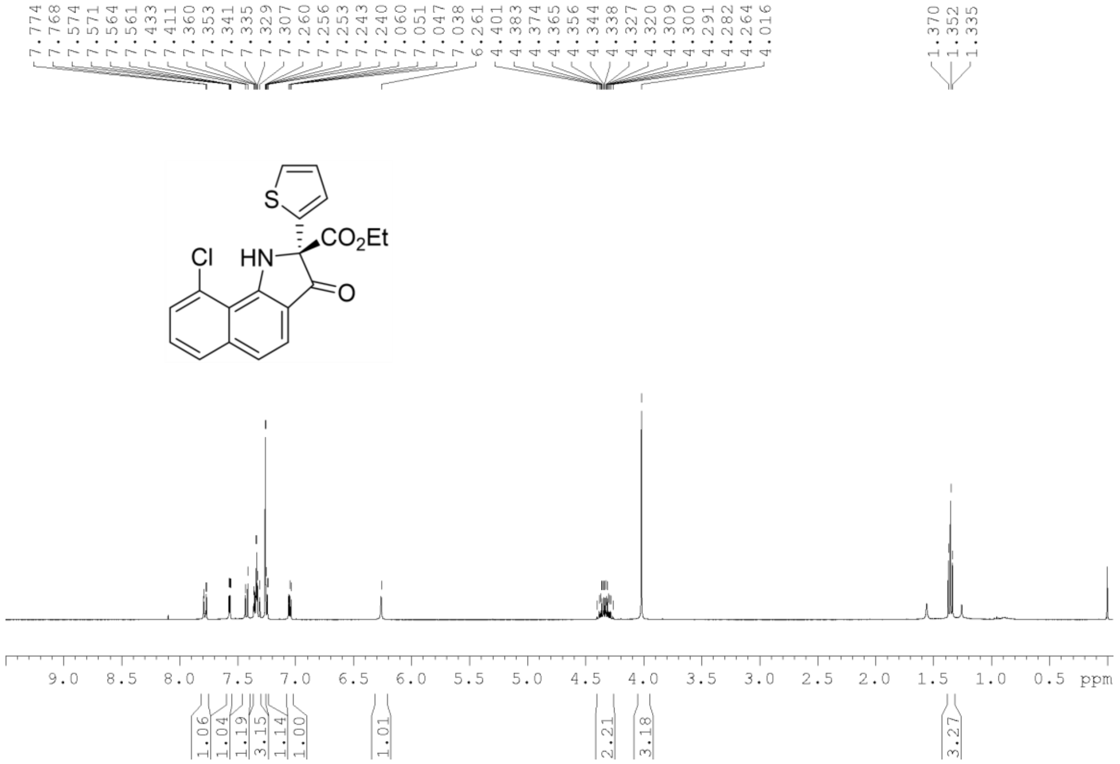

^{1}H NMR (400 MHz, $CDCl_3$) of **4iq**

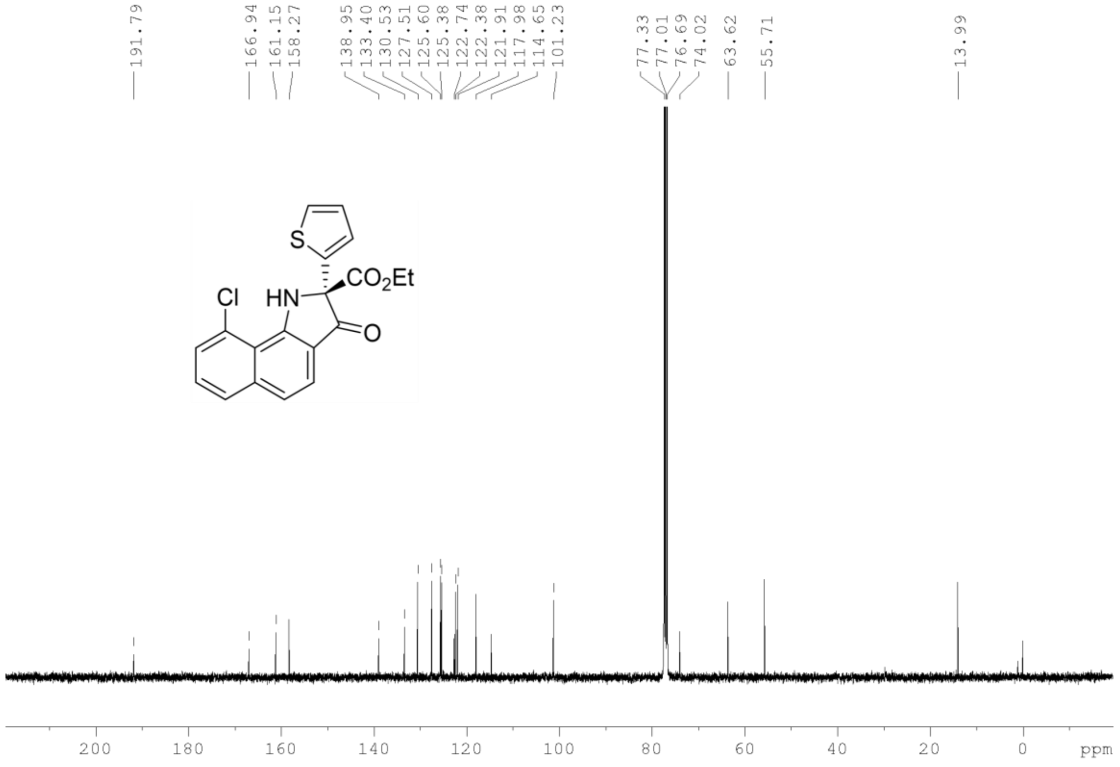

^{13}C NMR (100 MHz, $CDCl_3$) of **4iq**

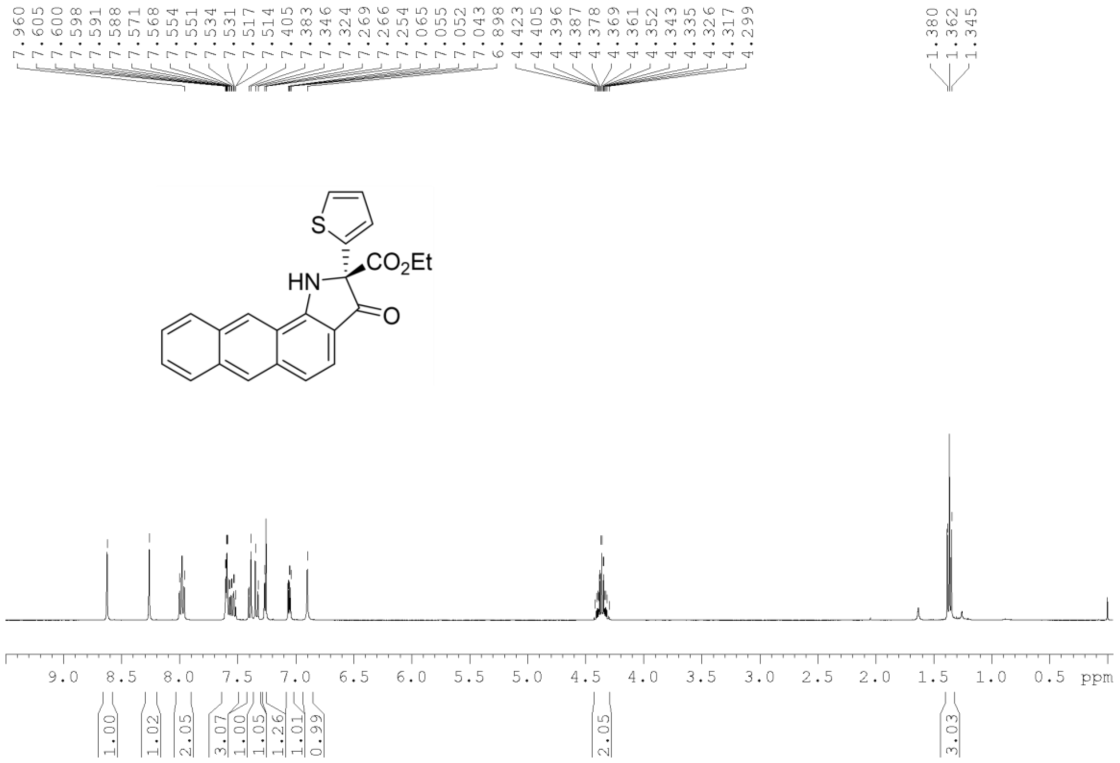

^{1}H NMR (400 MHz, CDCl3) of **4jq**

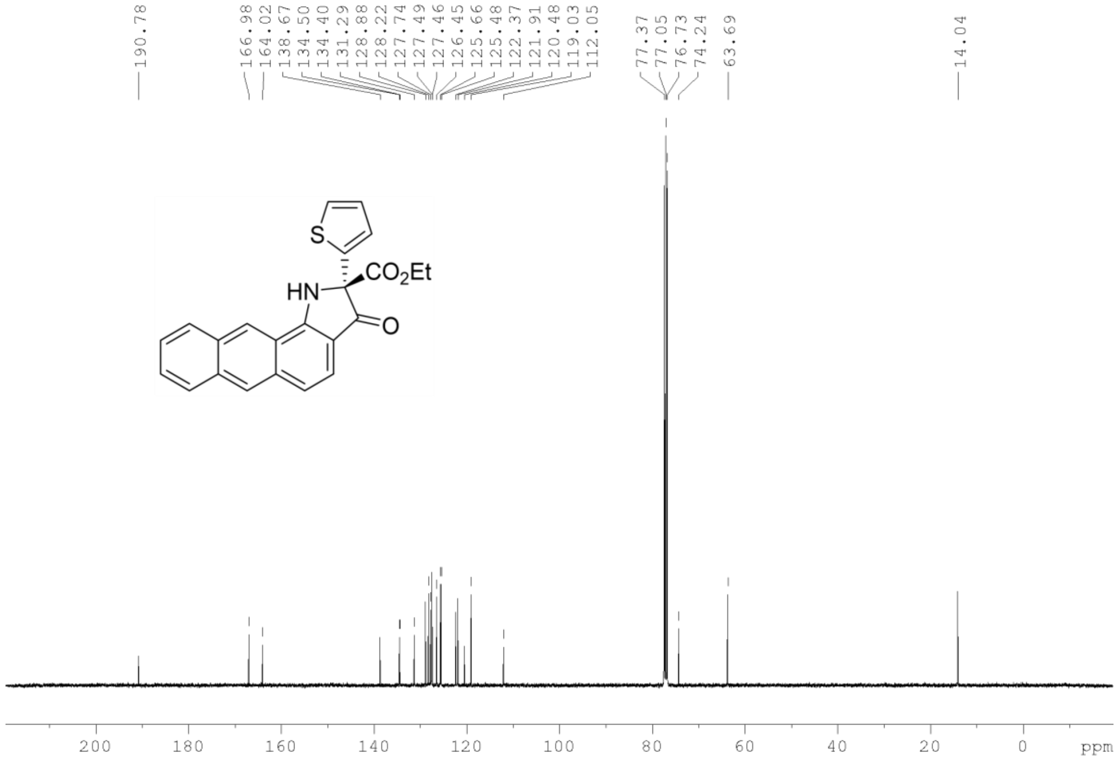

^{13}C NMR (100 MHz, CDCl3) of **4jq**

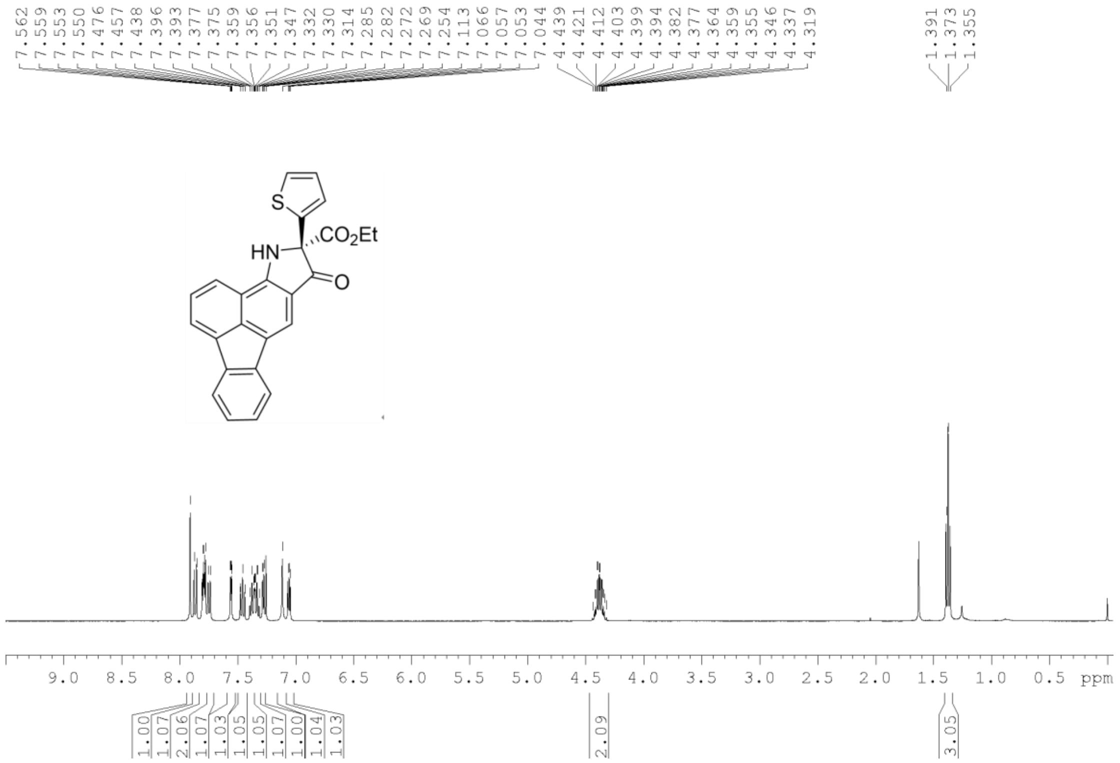

^{1}H NMR (400 MHz, $CDCl_3$) of **4kq**

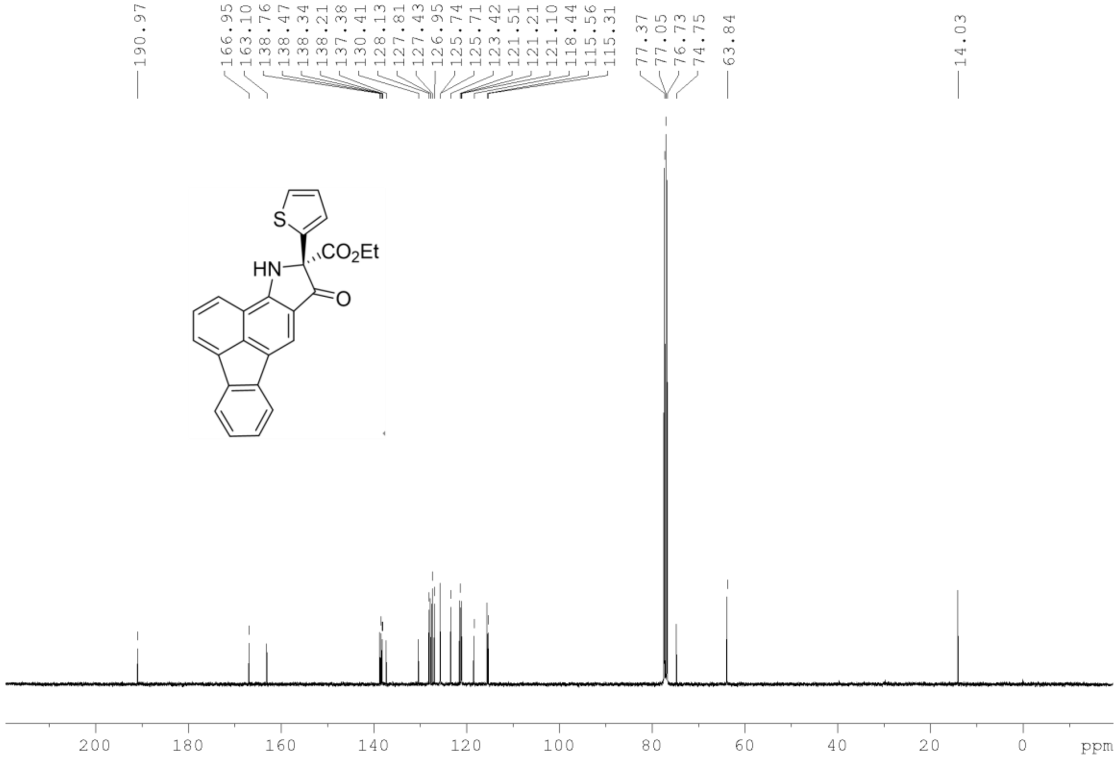

^{13}C NMR (100 MHz, $CDCl_3$) of **4kq**

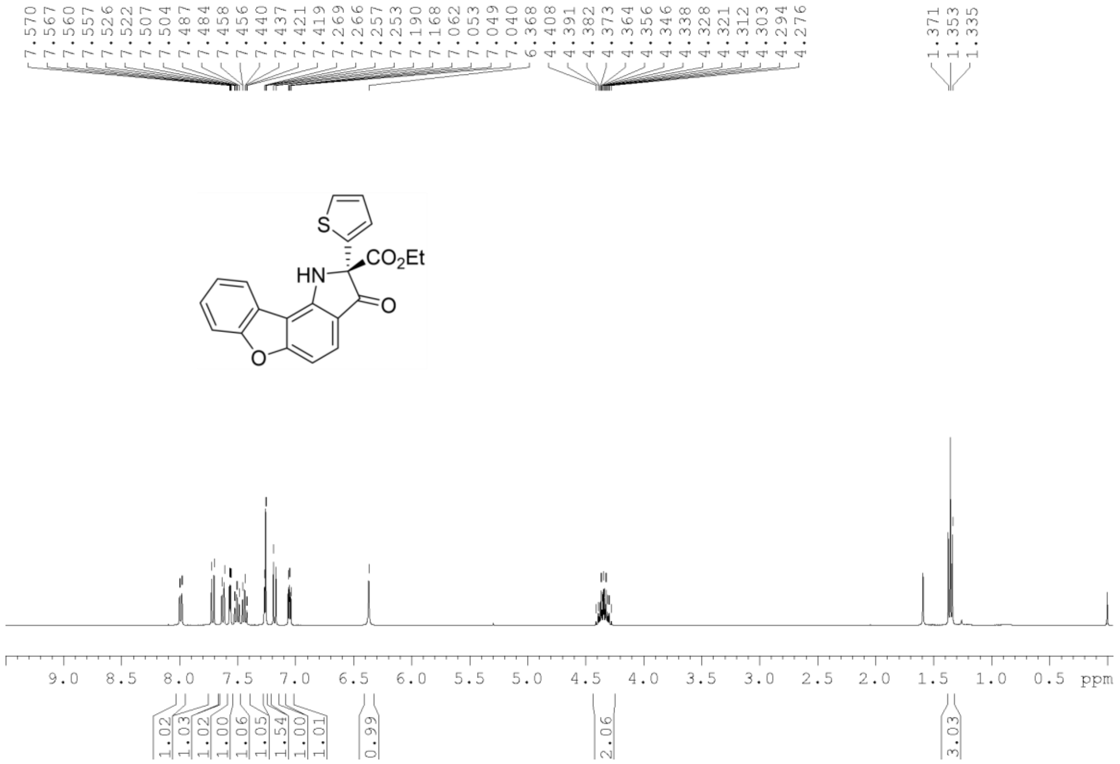

^{1}H NMR (400 MHz, $CDCl_3$) of **4lq**

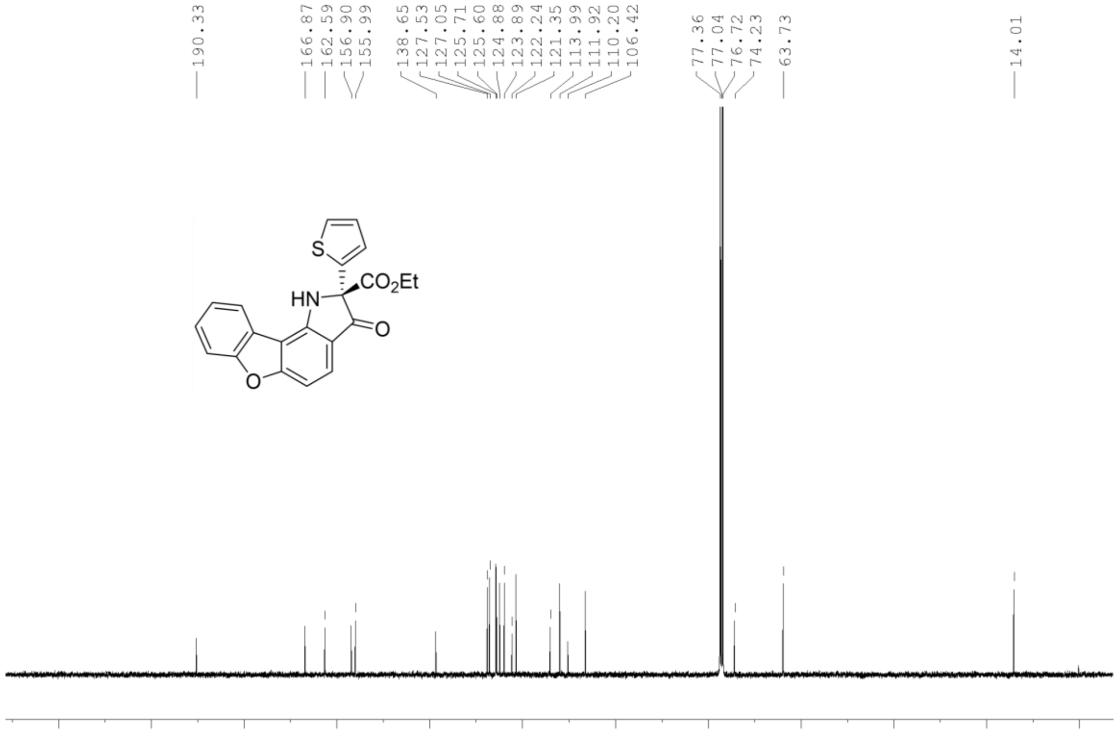

^{13}C NMR (100 MHz, $CDCl_3$) of **4lq**

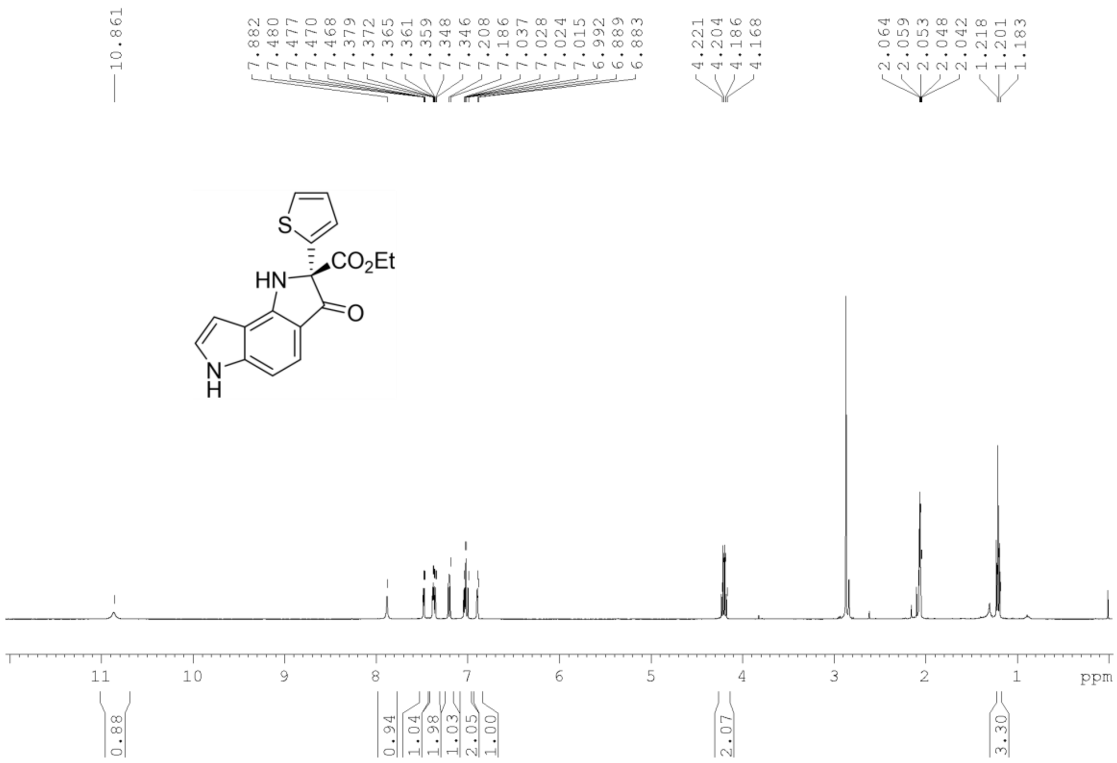

^{1}H NMR (400 MHz, acetone-d_6) of **4mq**

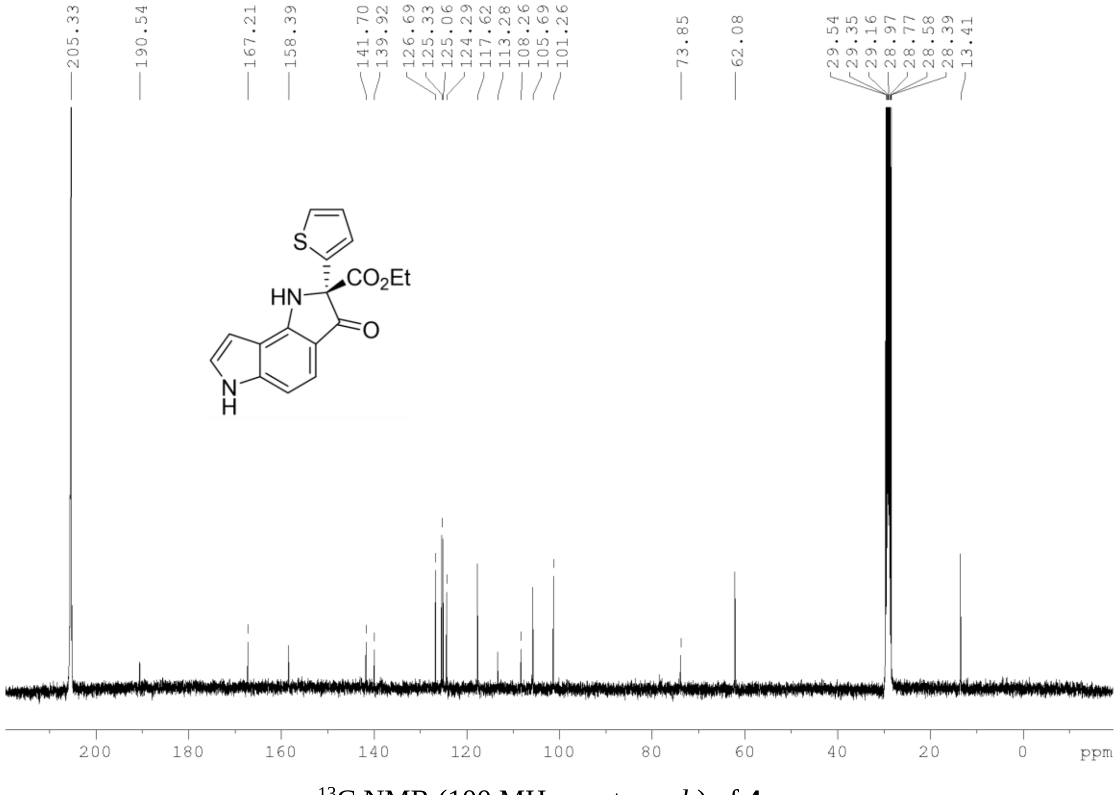

^{13}C NMR (100 MHz, acetone-d_6) of **4mq**

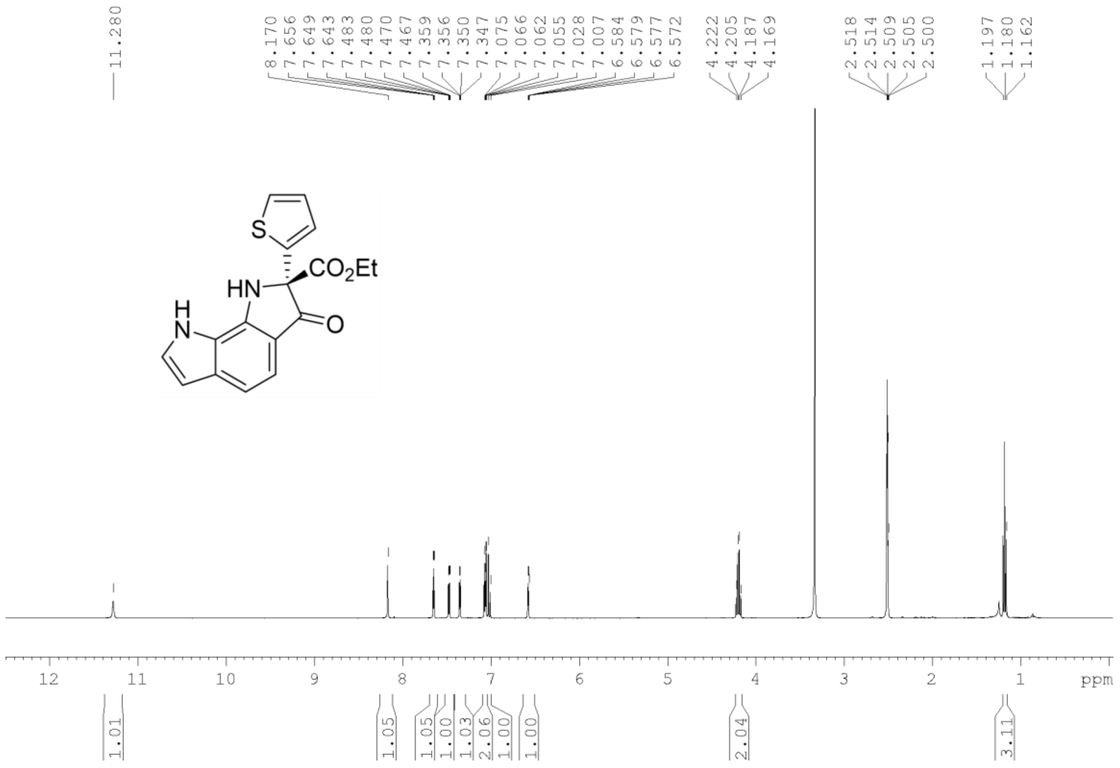

^{1}H NMR (400 MHz, DMSO-d_6) of **4nq**

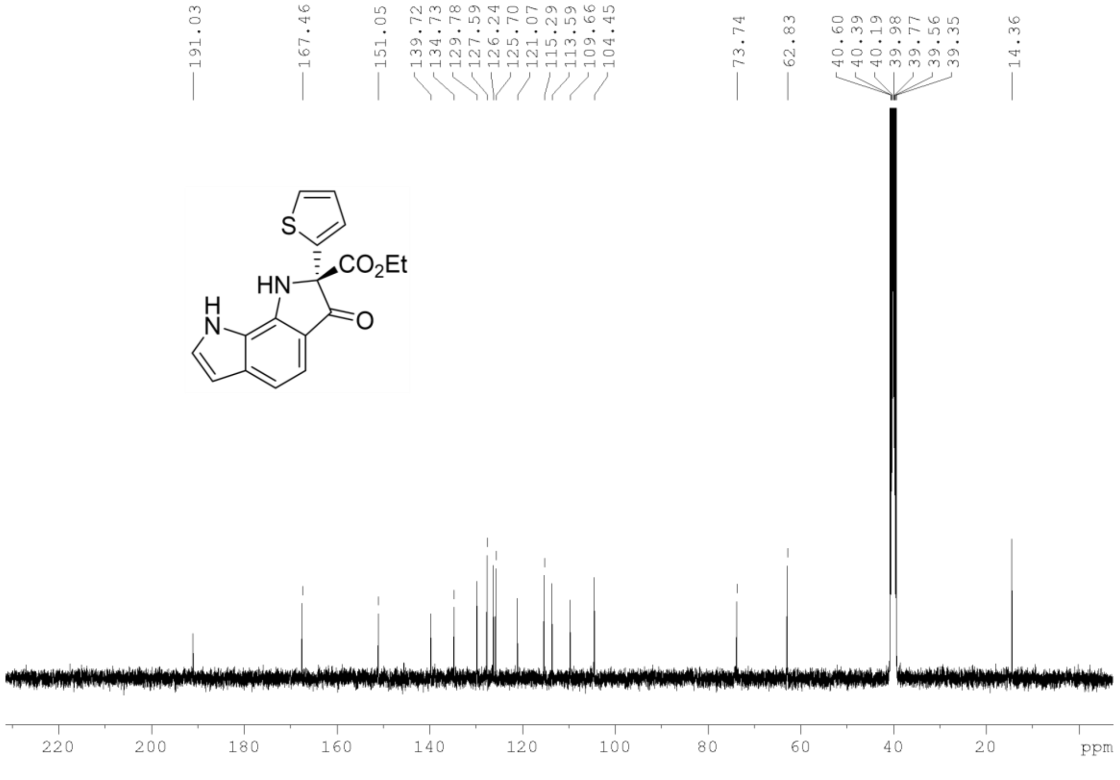

^{13}C NMR (100 MHz, DMSO-d_6) of **4nq**

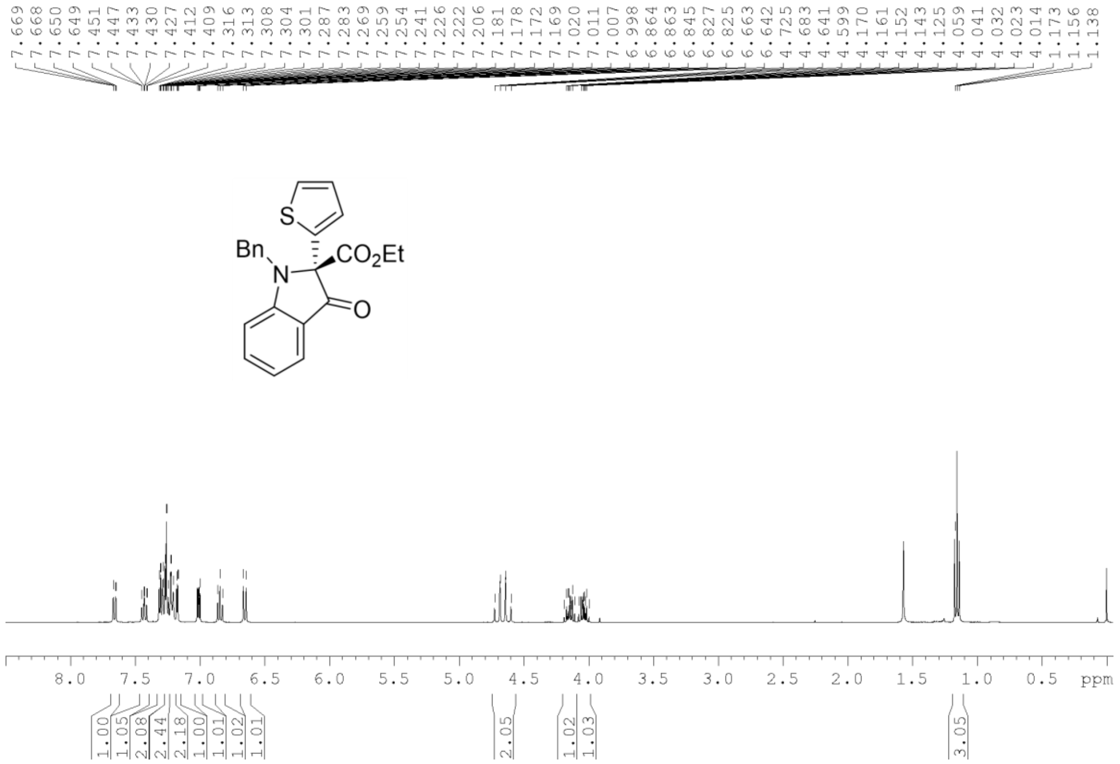

^{1}H NMR (400 MHz, CDCl3) of **4pq**

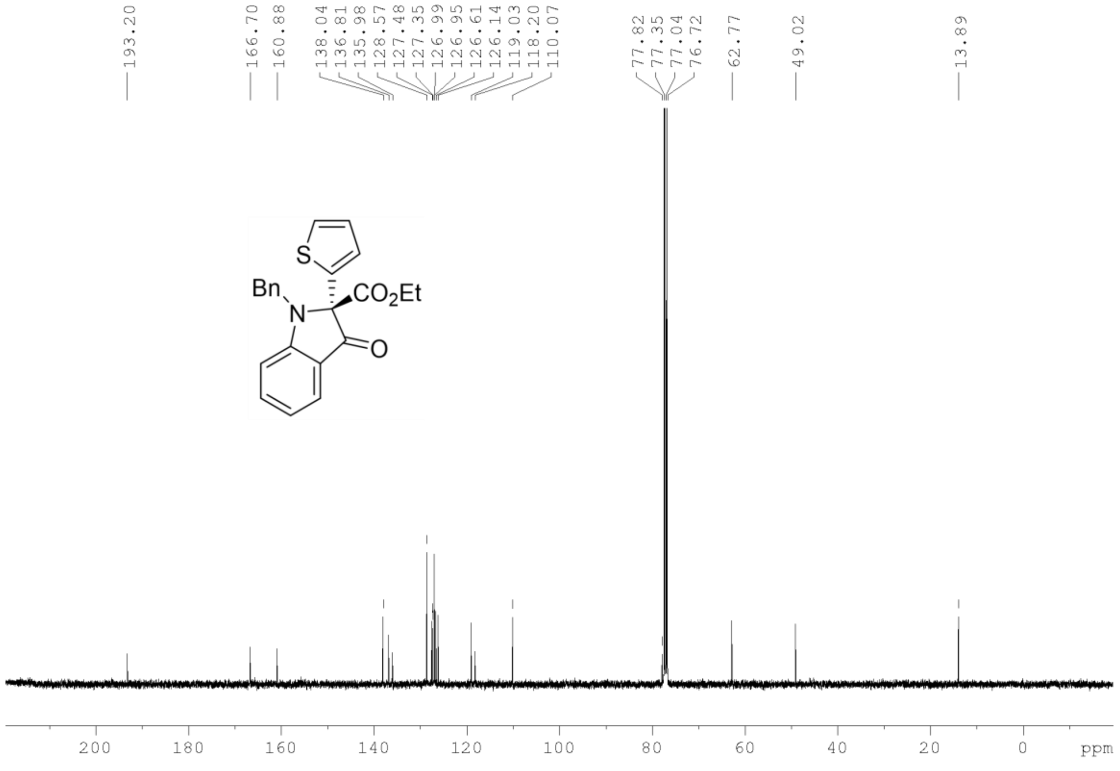

^{13}C NMR (100 MHz, CDCl3) of **4pq**

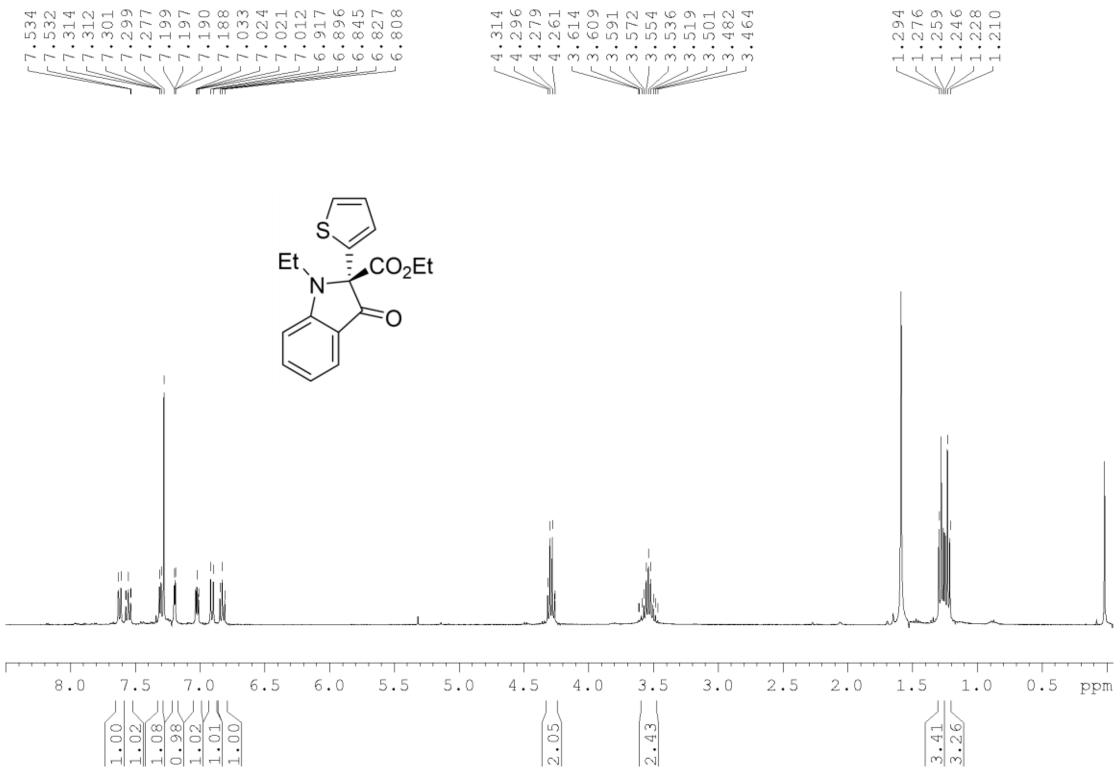

^{1}H NMR (400 MHz, CDCl3) of **4rq**

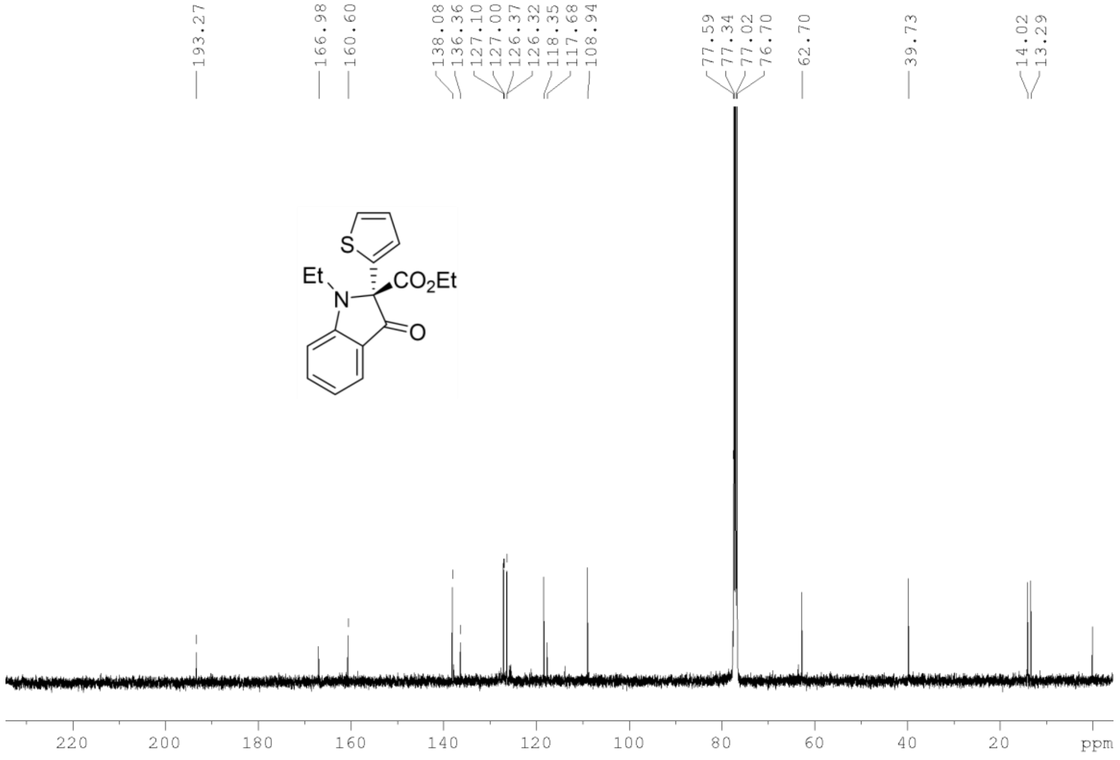

^{13}C NMR (100 MHz, CDCl3) of **4rq**

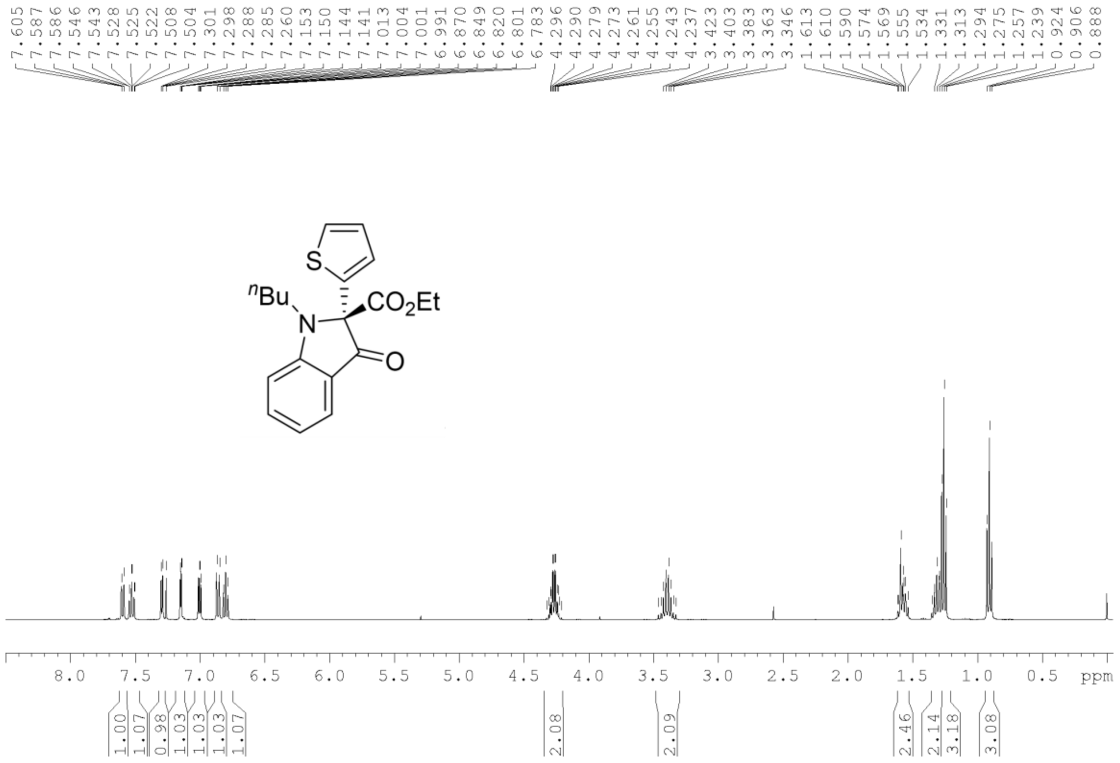

^{1}H NMR (400 MHz, $CDCl_3$) of **4sq**

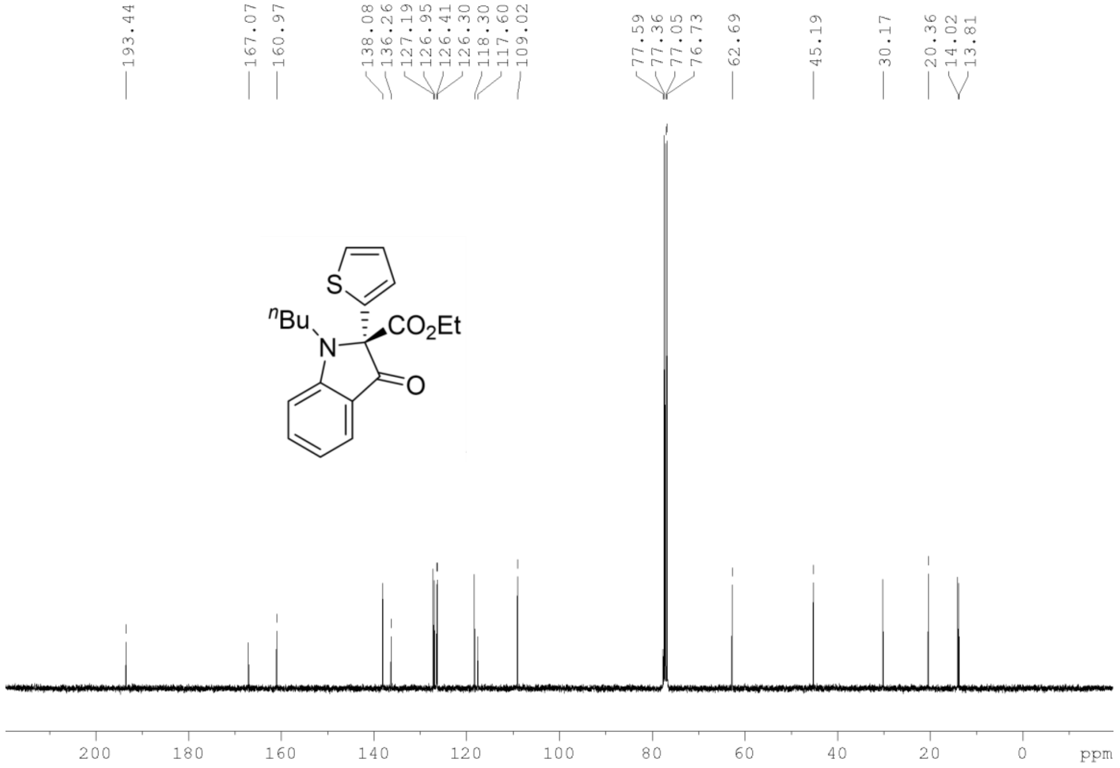

^{13}C NMR (100 MHz, $CDCl_3$) of **4sq**

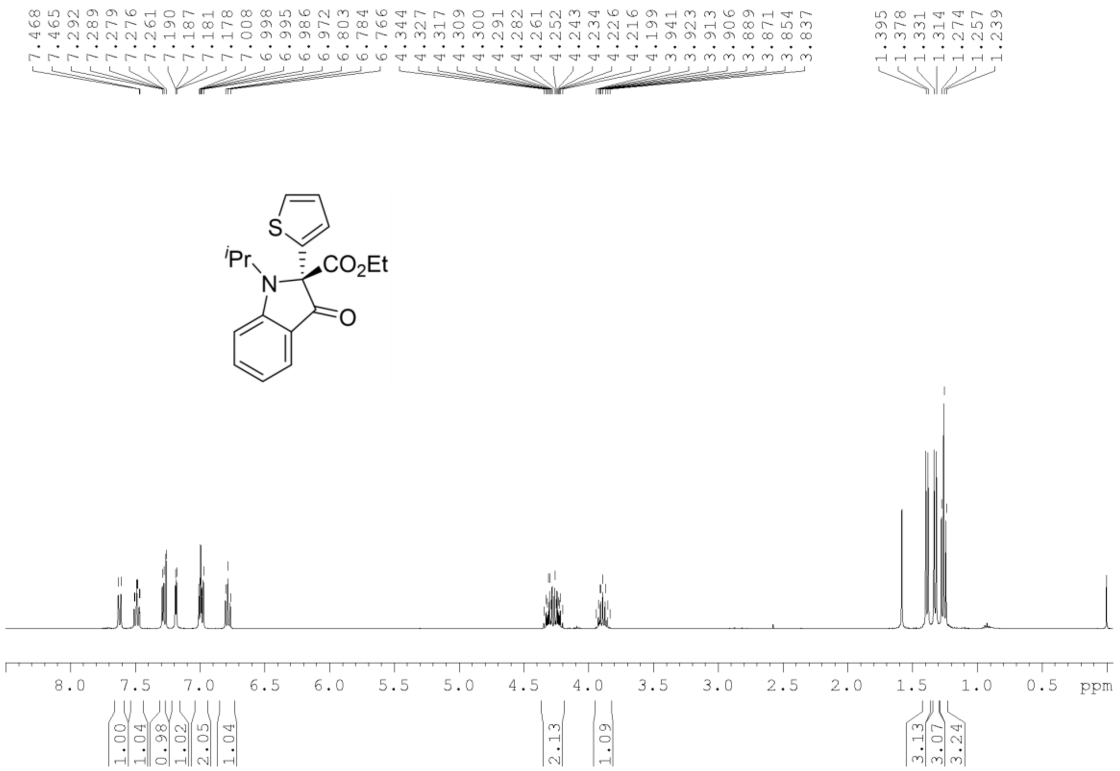

^{1}H NMR (400 MHz, $CDCl_3$) of **4tq**

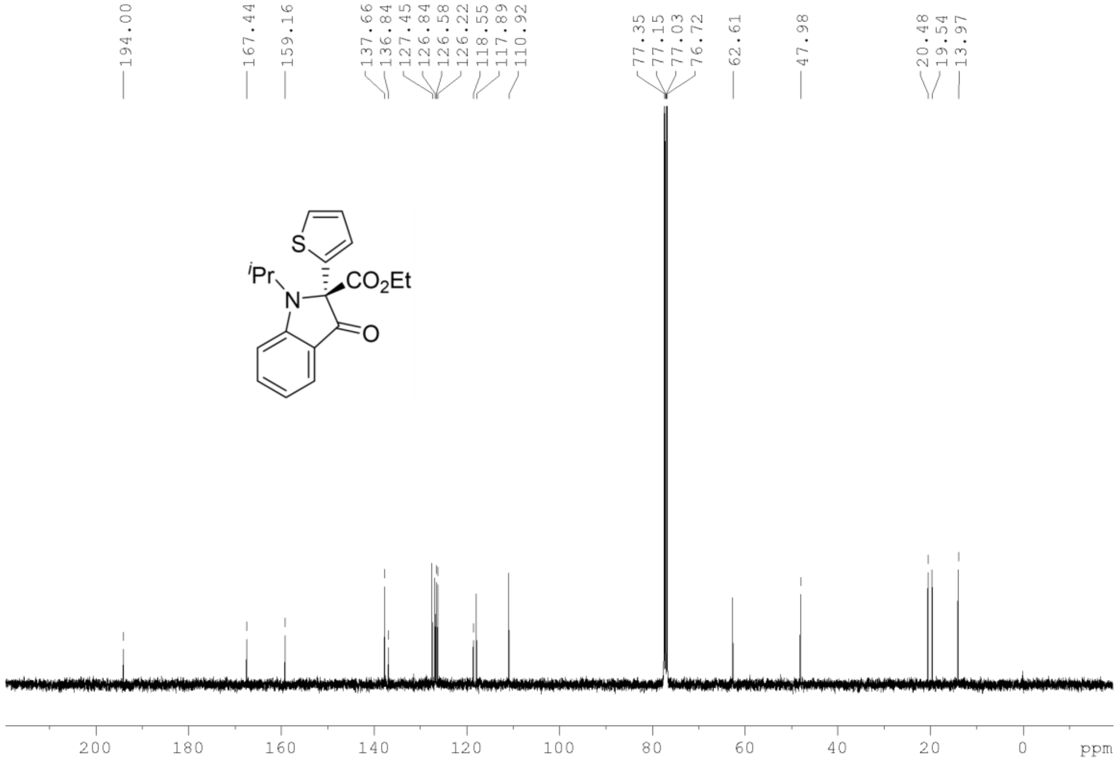

^{13}C NMR (100 MHz, $CDCl_3$) of **4tq**

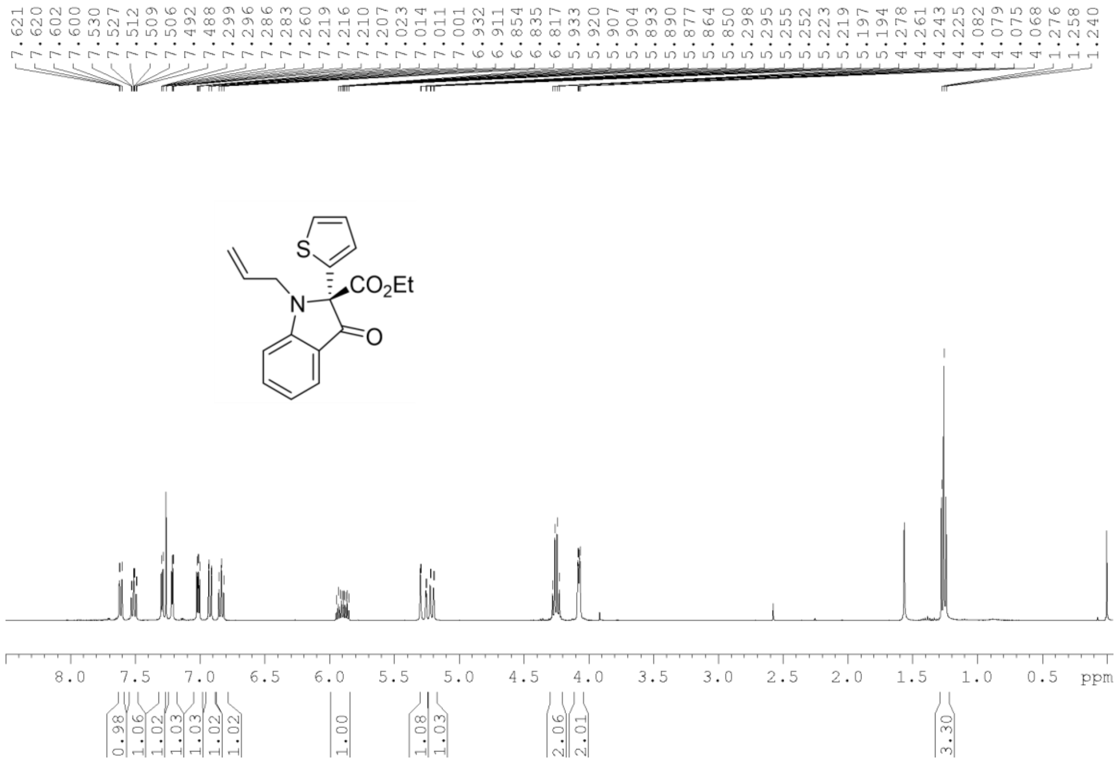

^{1}H NMR (400 MHz, $CDCl_3$) of **4uq**

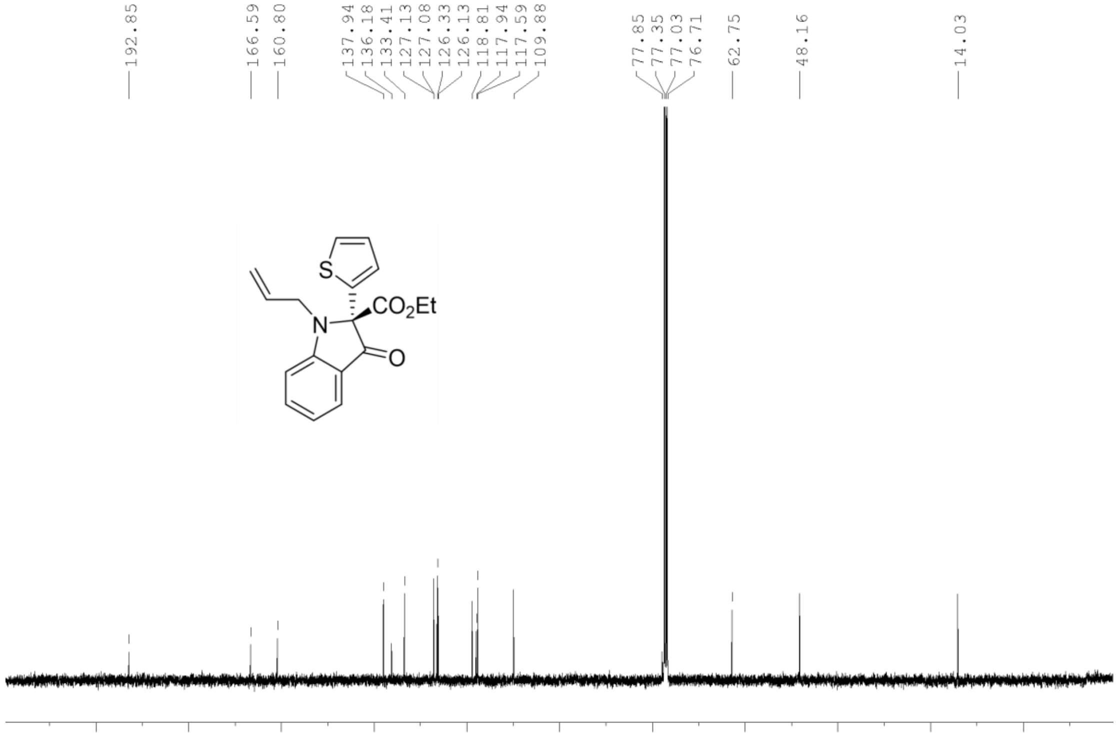

^{13}C NMR (100 MHz, $CDCl_3$) of **4uq**

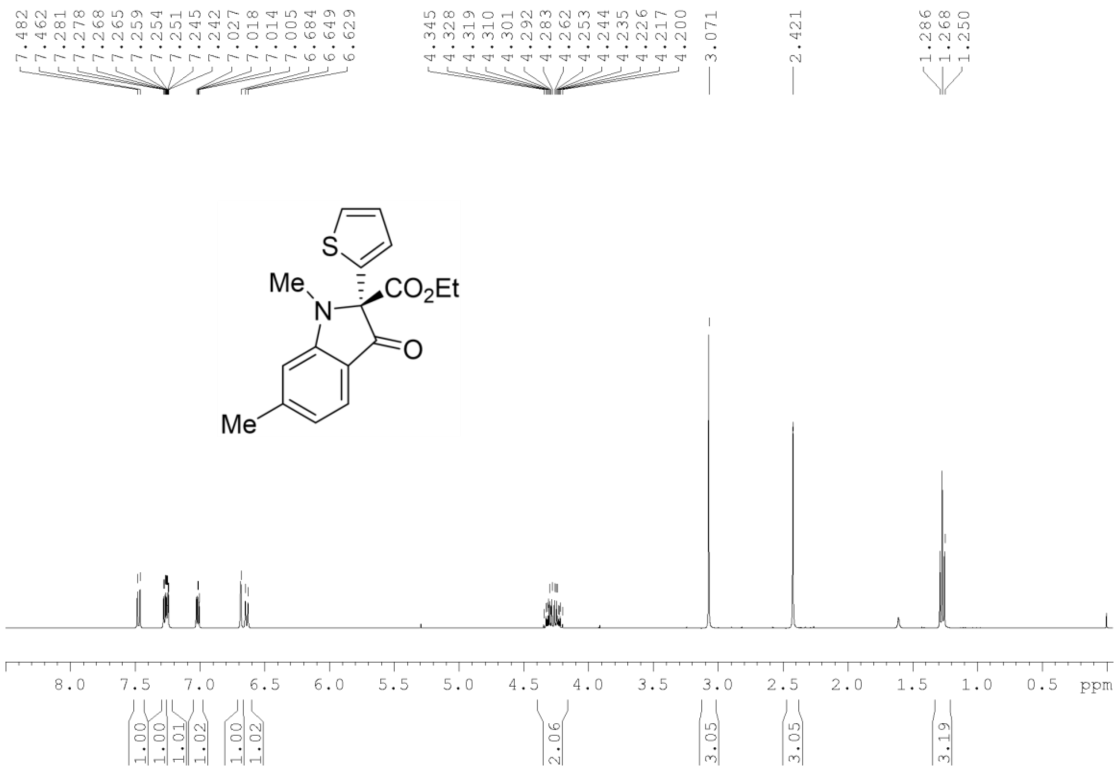

^{1}H NMR (400 MHz, $CDCl_3$) of **4vq**

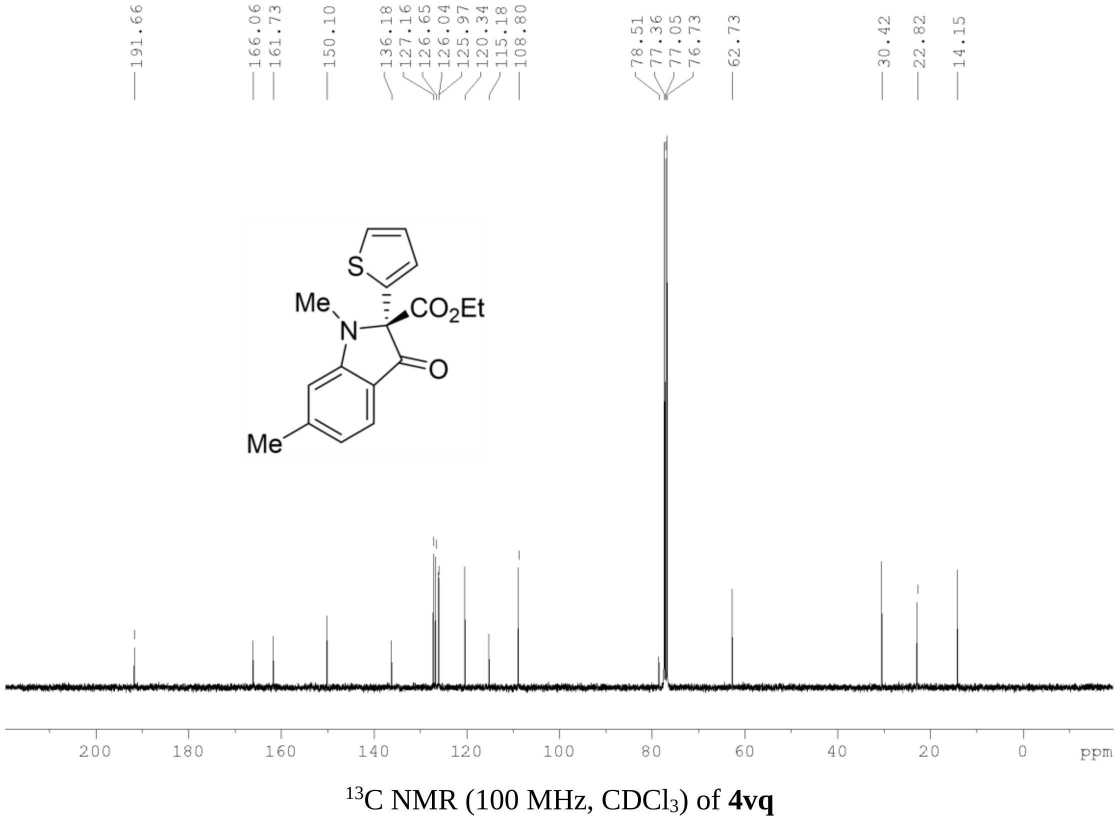

^{13}C NMR (100 MHz, $CDCl_3$) of **4vq**

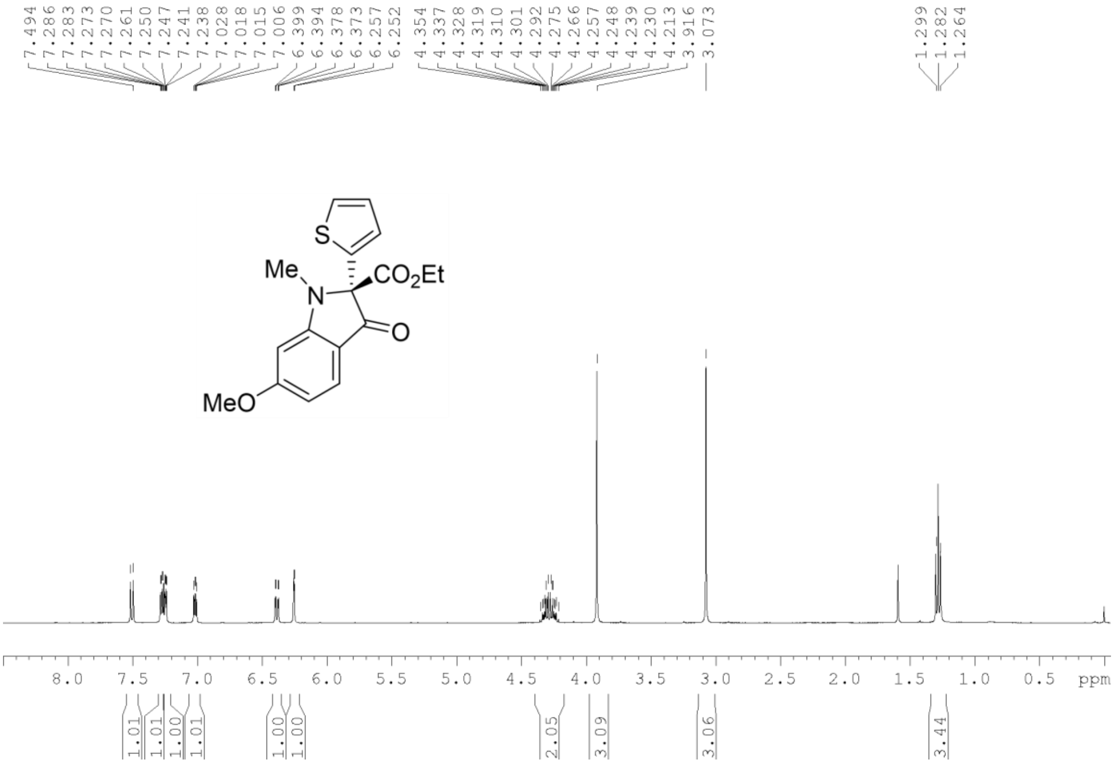

^{1}H NMR (400 MHz, $CDCl_3$) of **4wq**

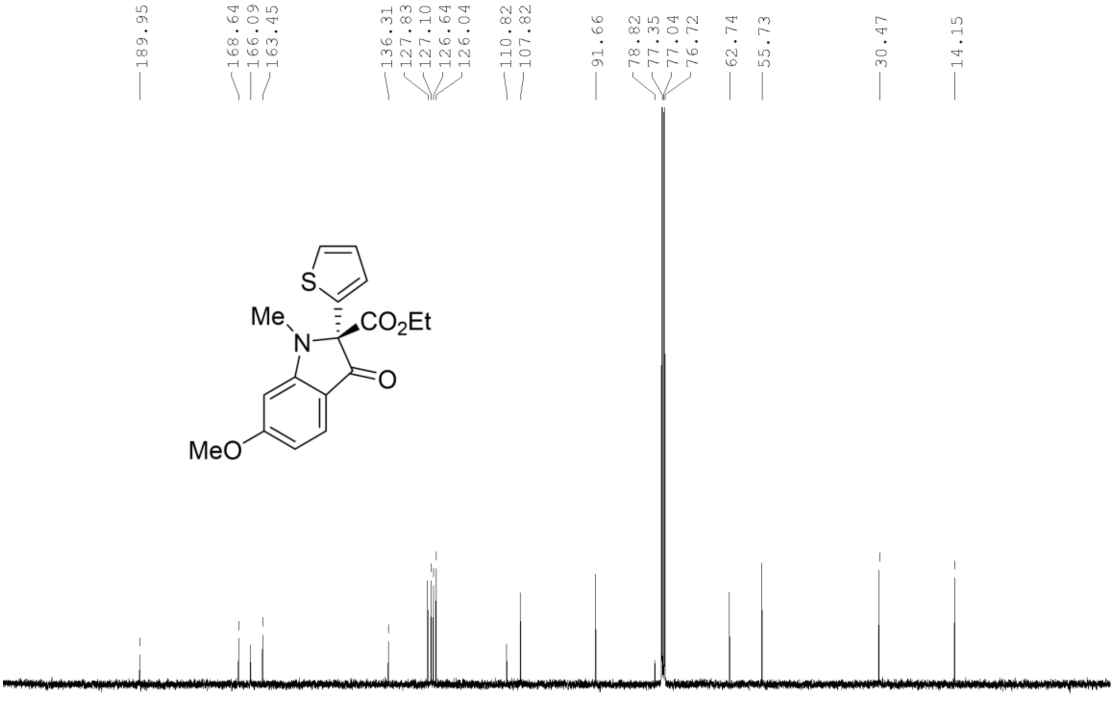

^{13}C NMR (100 MHz, $CDCl_3$) of **4wq**

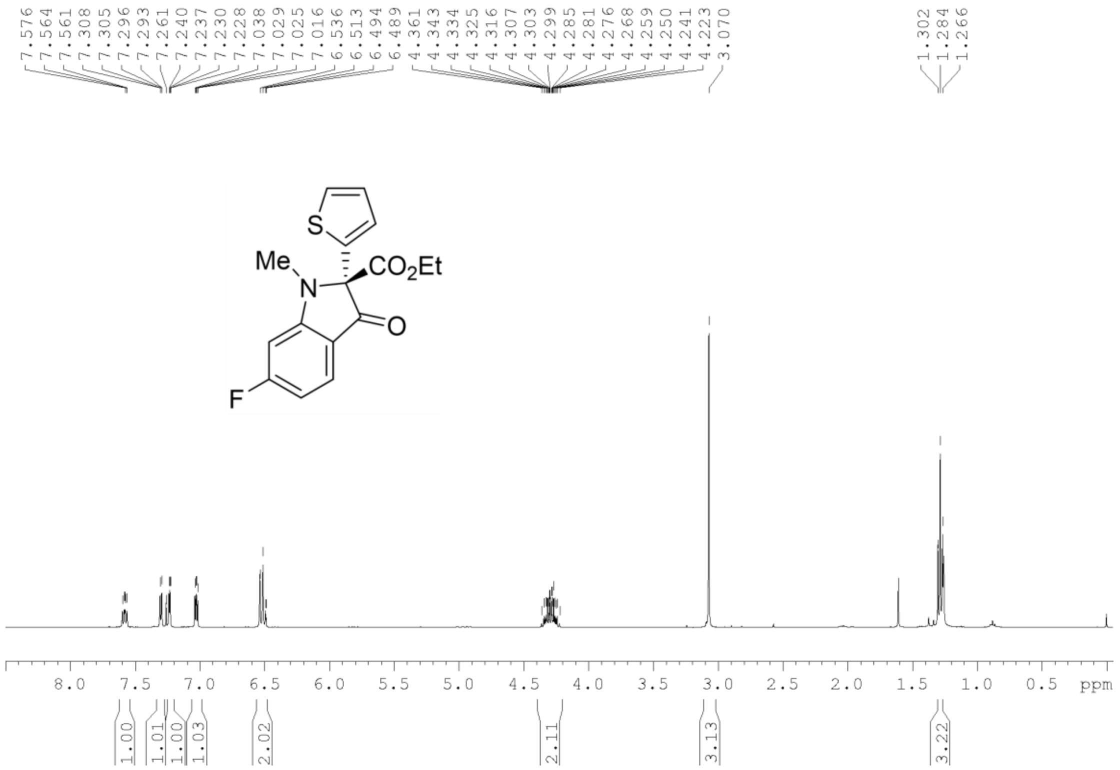

^{1}H NMR (400 MHz, $CDCl_3$) of **4xq**

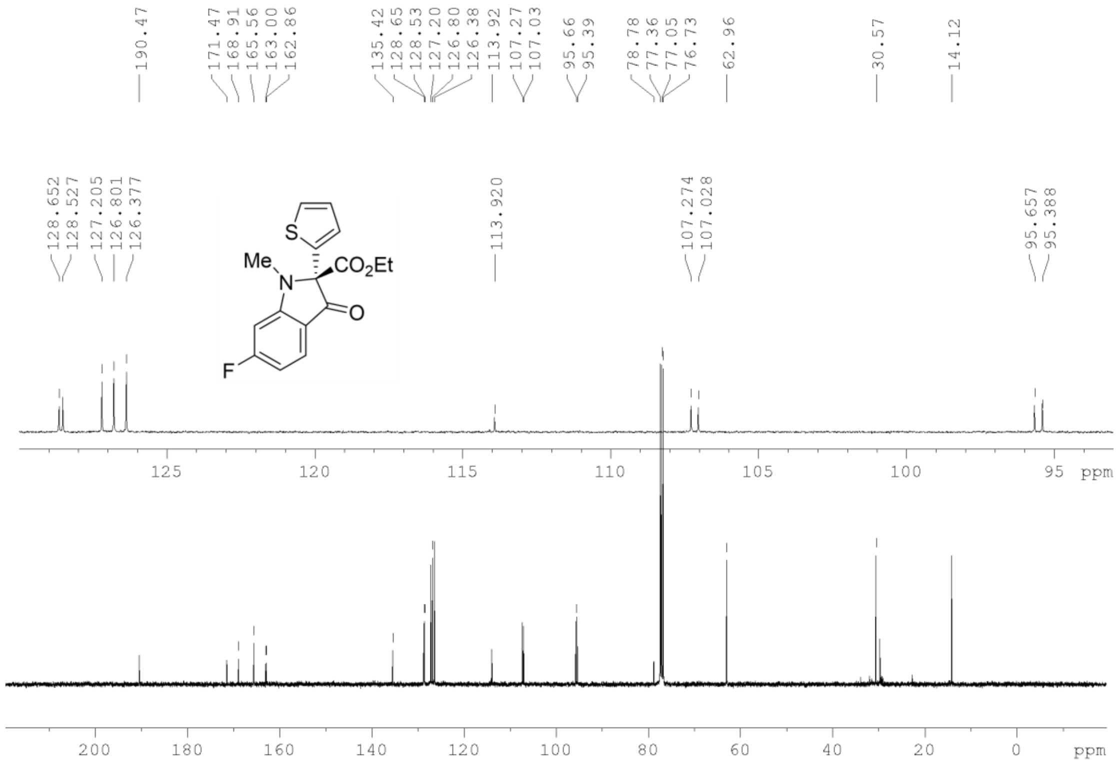

^{13}C NMR (100 MHz, $CDCl_3$) of **4xq**

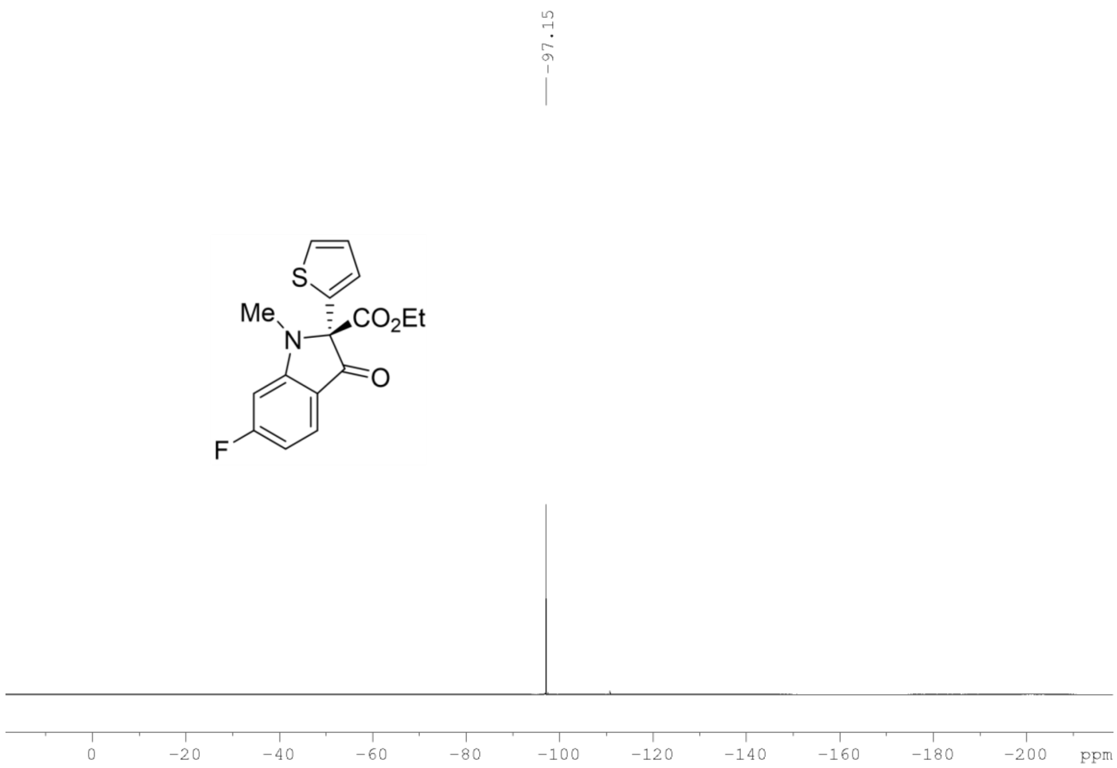

^{19}F NMR (376 MHz, $CDCl_3$) of **4xq**

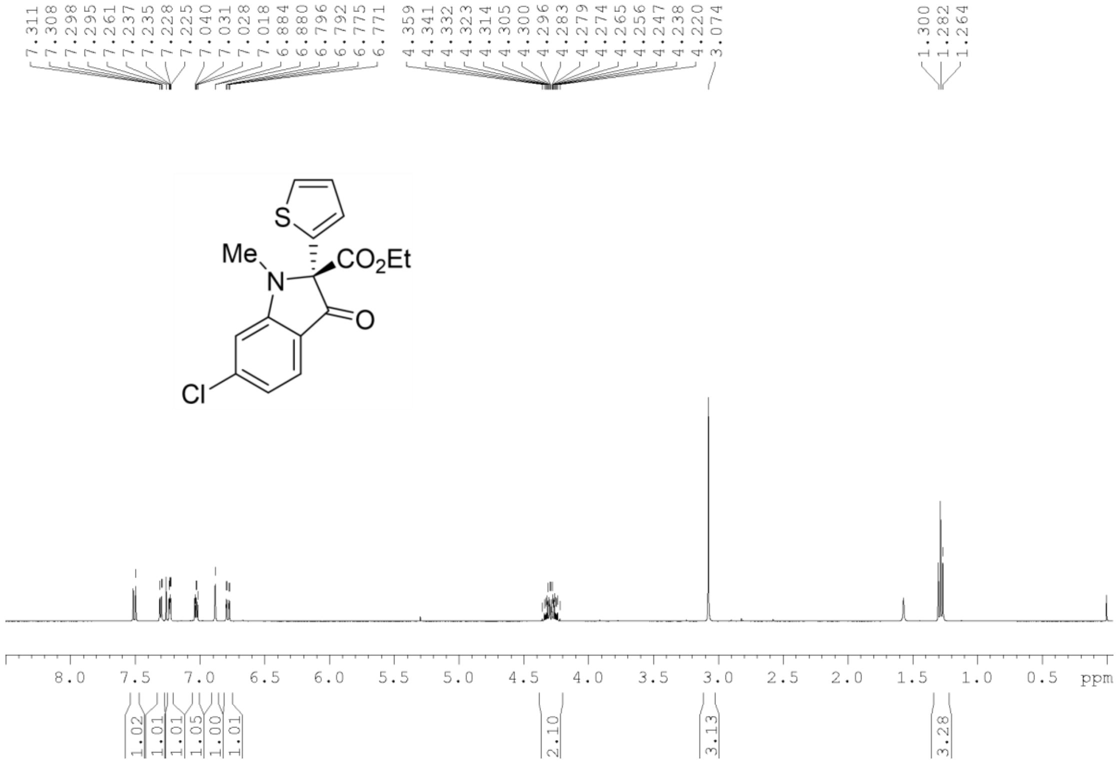

^{1}H NMR (400 MHz, $CDCl_3$) of **4yq**

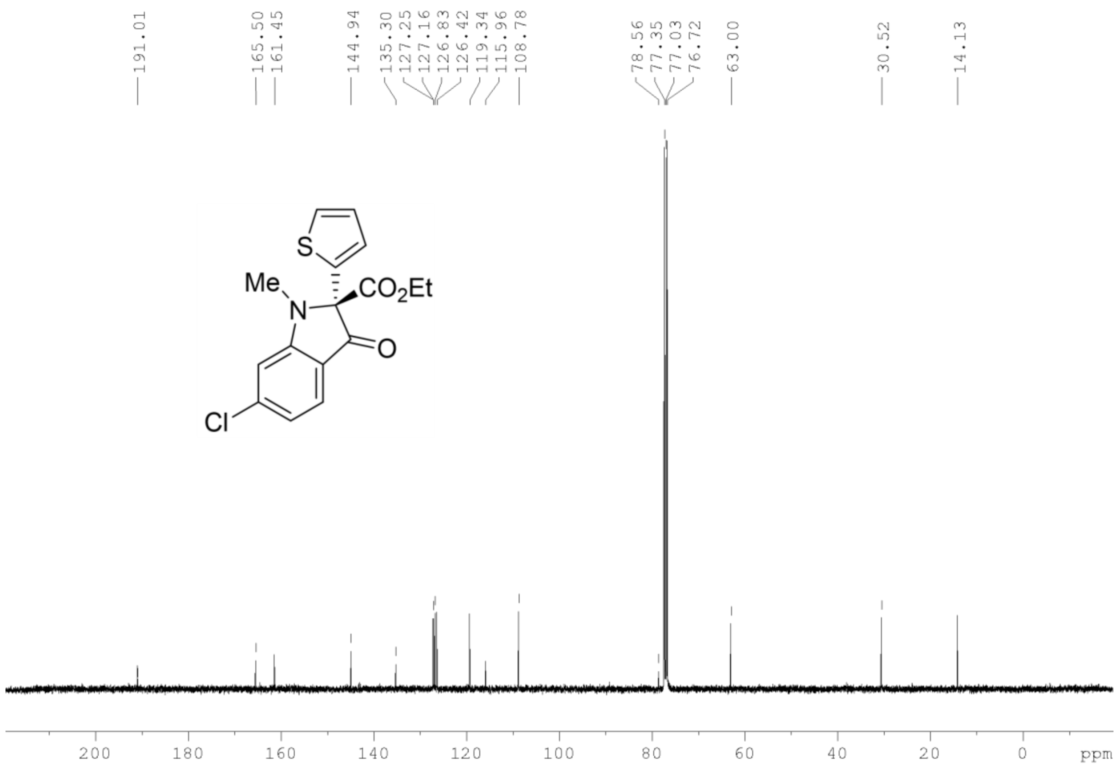

^{13}C NMR (100 MHz, $CDCl_3$) of **4yq**

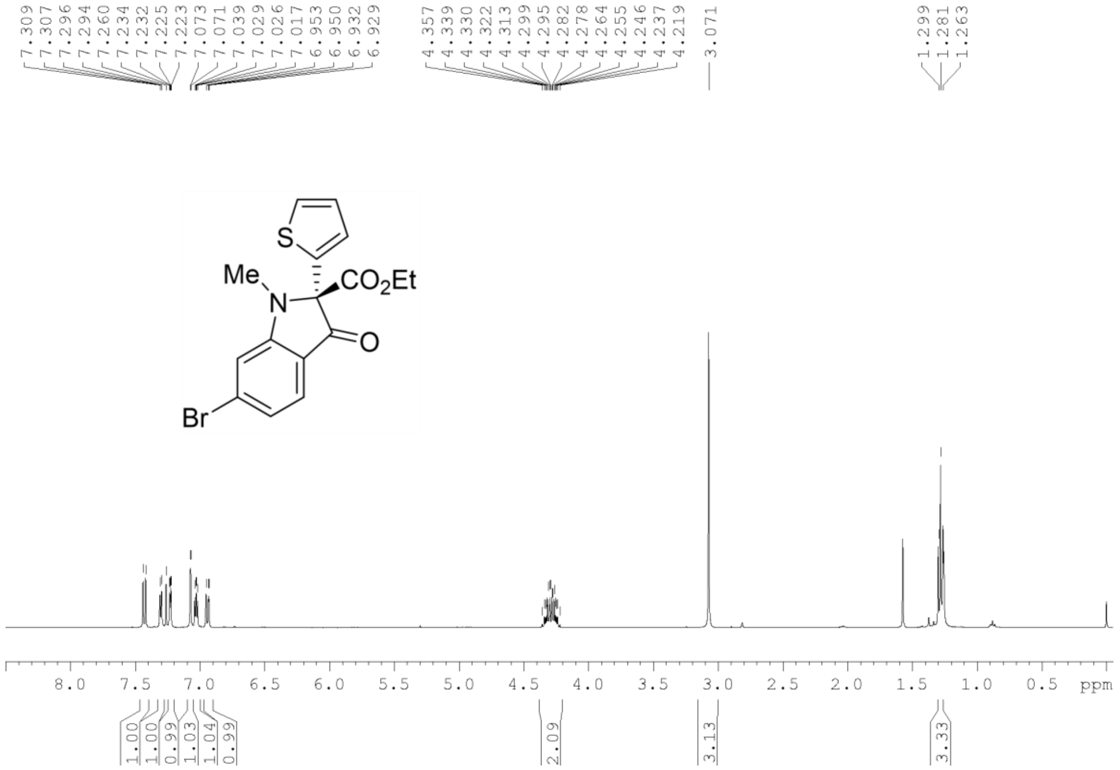

^{1}H NMR (400 MHz, $CDCl_3$) of **4zq**

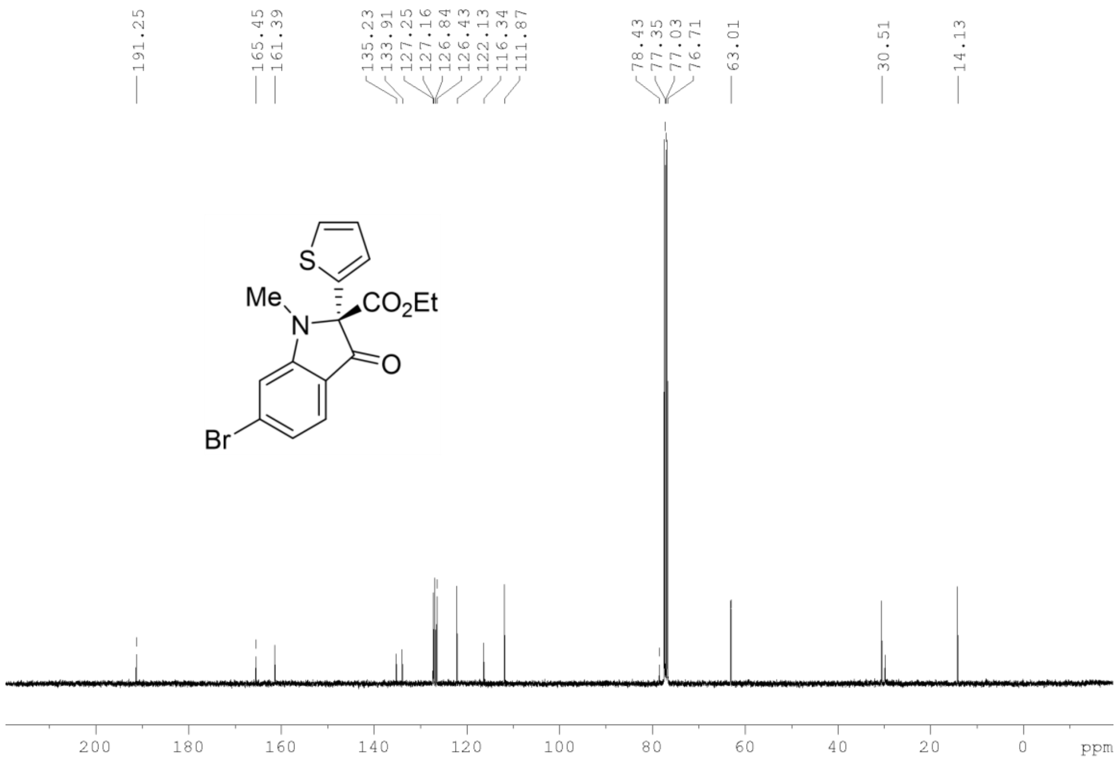

^{13}C NMR (100 MHz, $CDCl_3$) of **4zq**

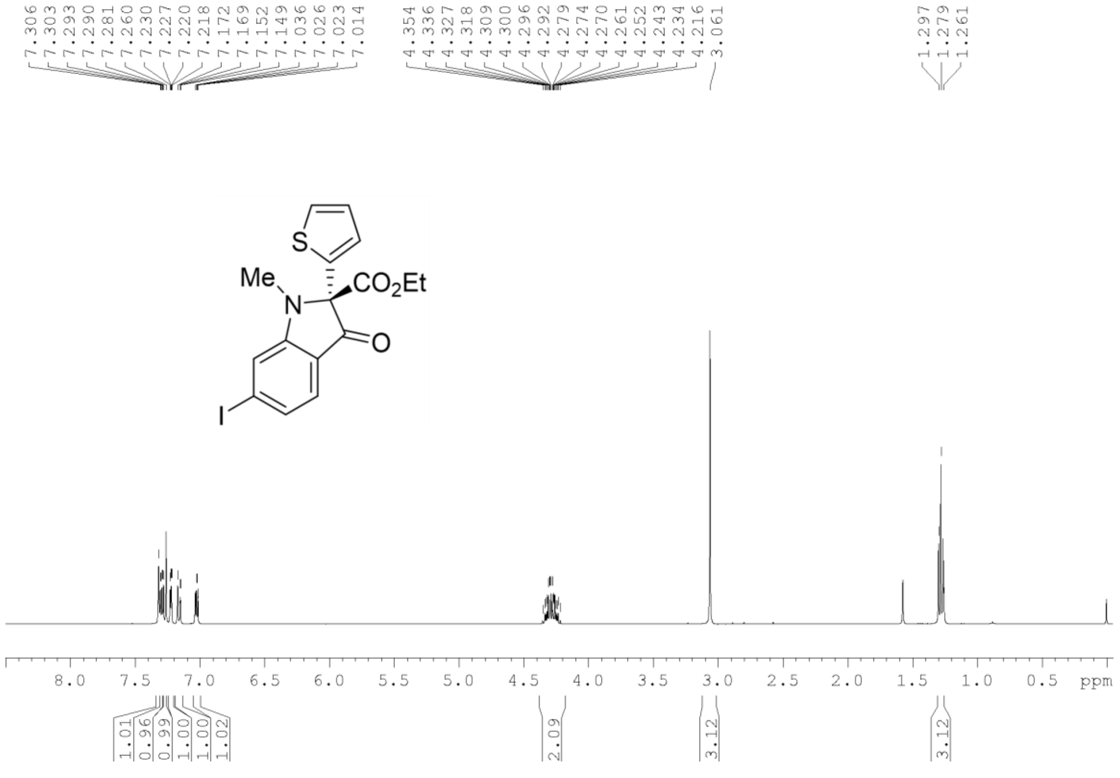

^{1}H NMR (400 MHz, $CDCl_3$) of **4Aq**

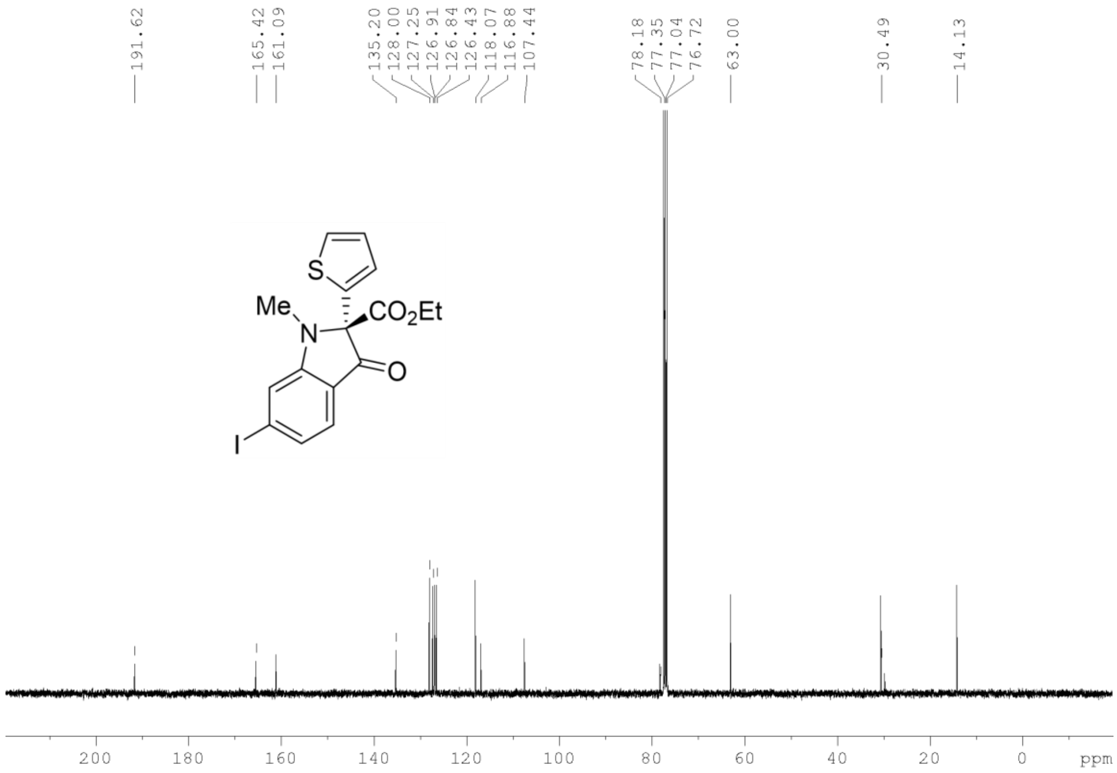

^{13}C NMR (100 MHz, $CDCl_3$) of **4Aq**

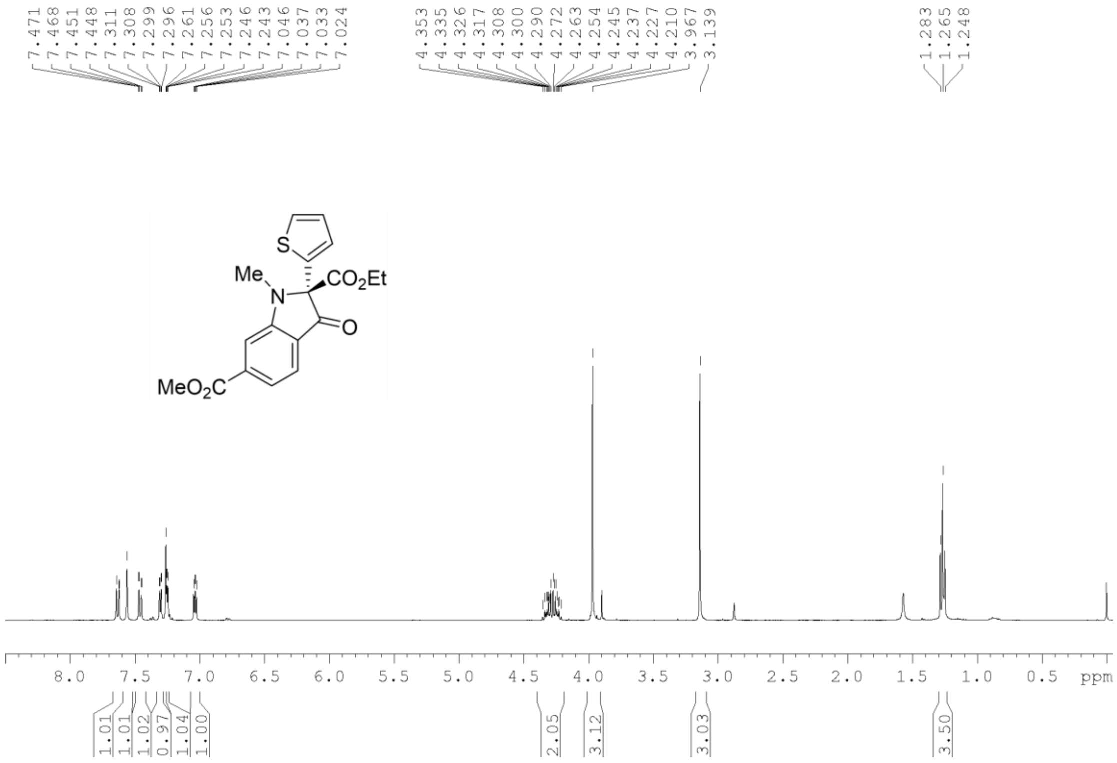

^{1}H NMR (400 MHz, $CDCl_3$) of **4Bq**

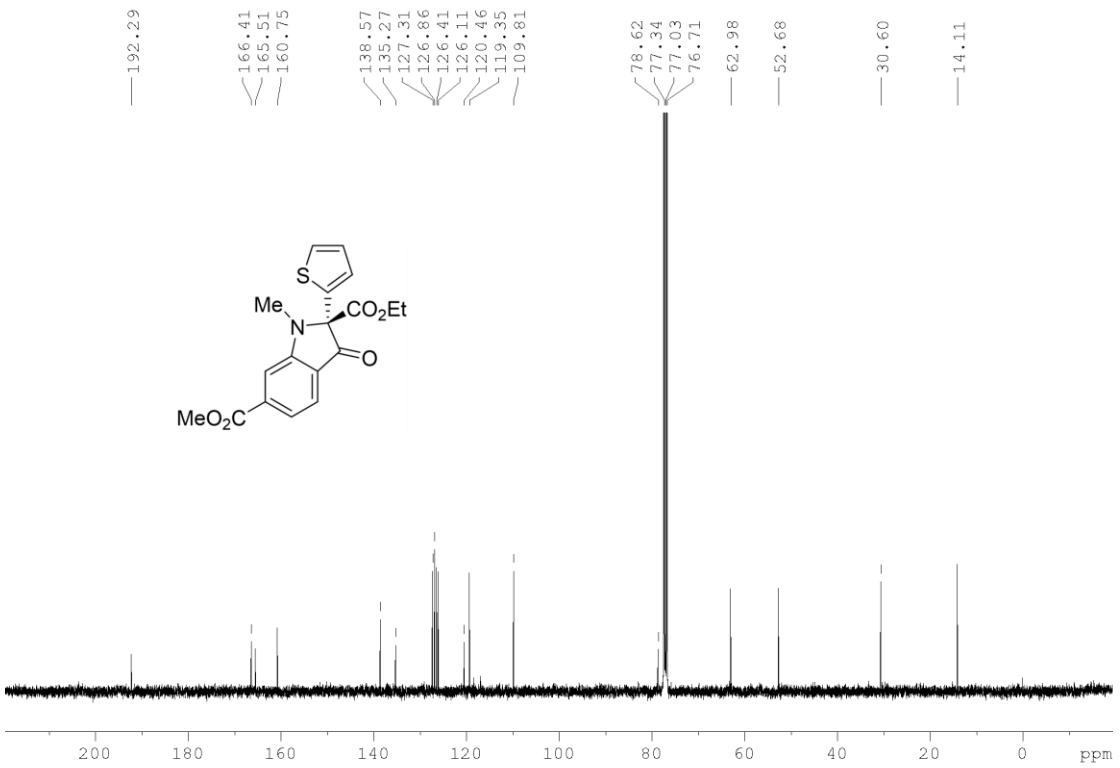

^{13}C NMR (100 MHz, $CDCl_3$) of **4Bq**

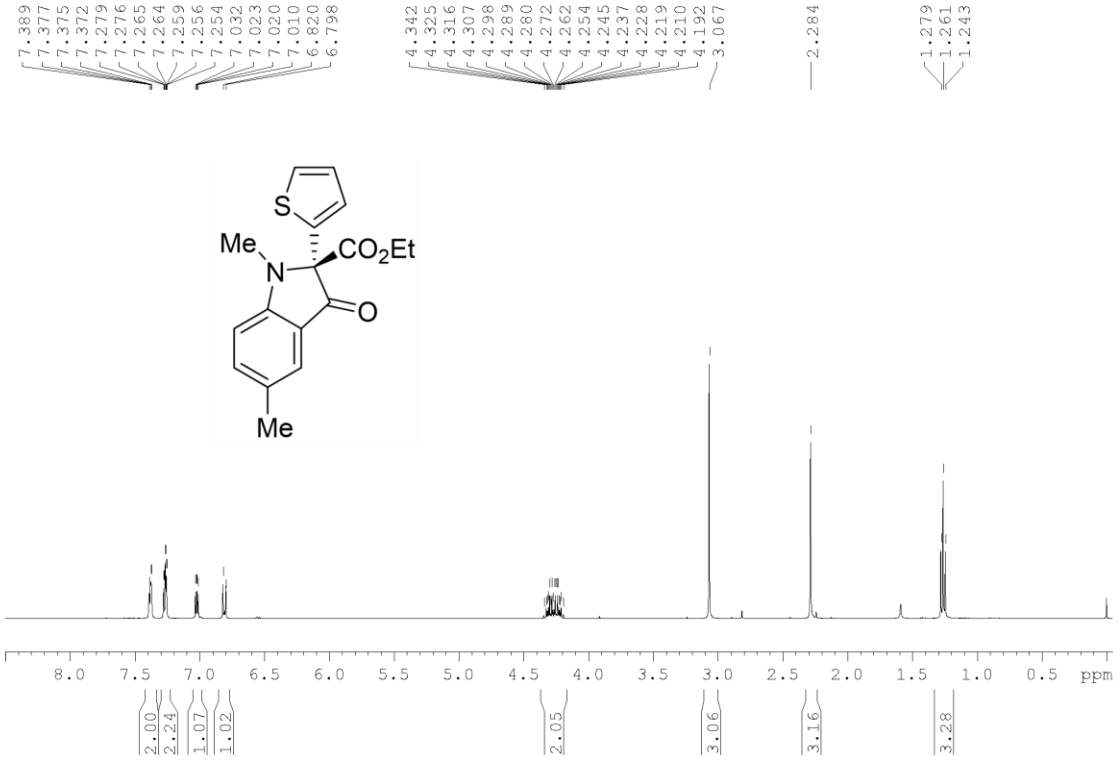

^{1}H NMR (400 MHz, $CDCl_3$) of **4Cq**

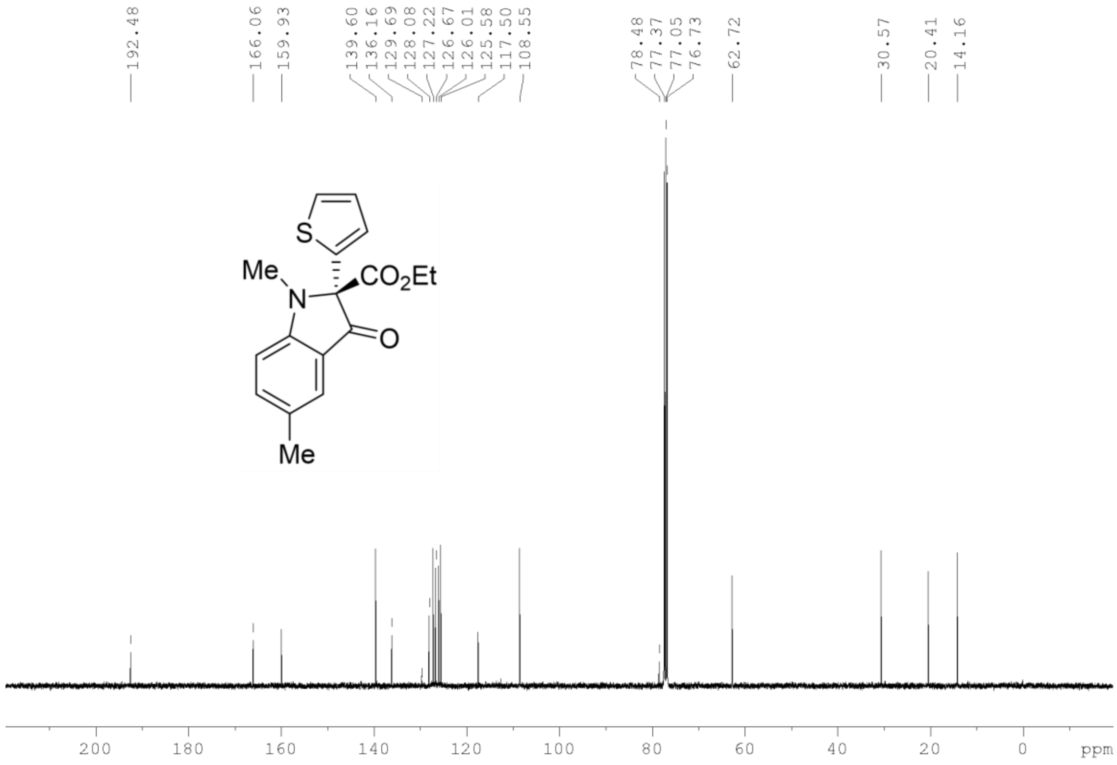

^{13}C NMR (100 MHz, CDCl$_3$) of **4Cq**

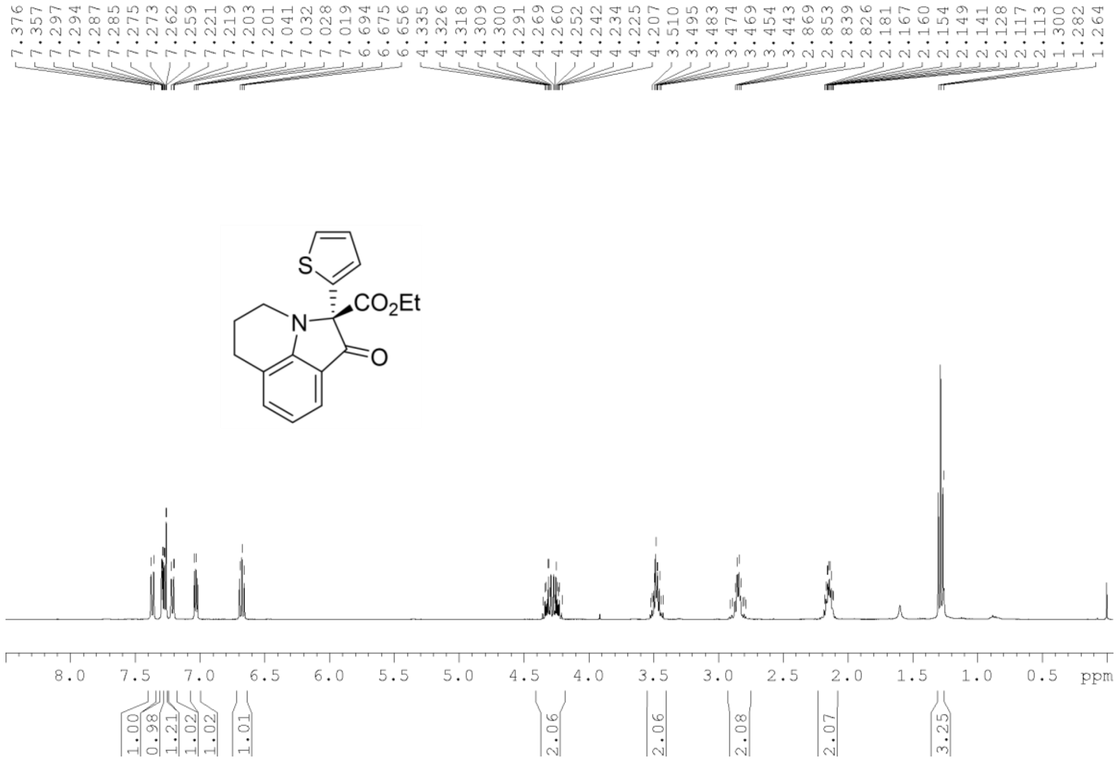

^{1}H NMR (400 MHz, CDCl$_3$) of **4Fq**

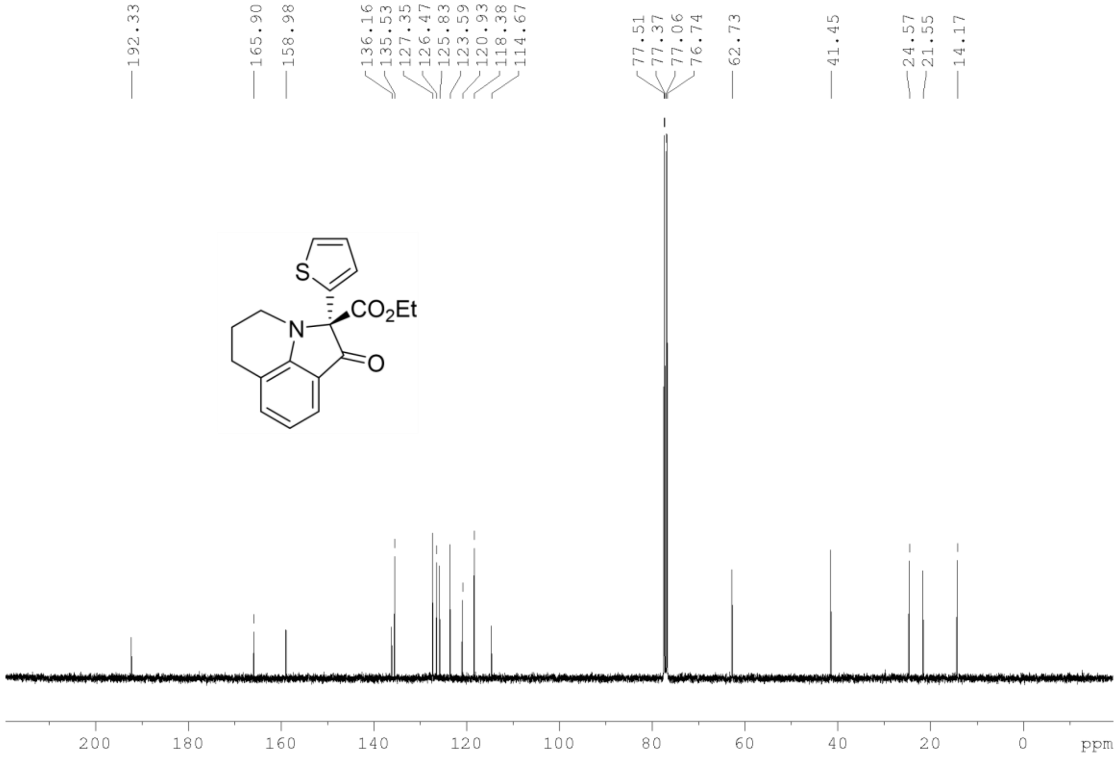

^{13}C NMR (100 MHz, $CDCl_3$) of **4Fq**

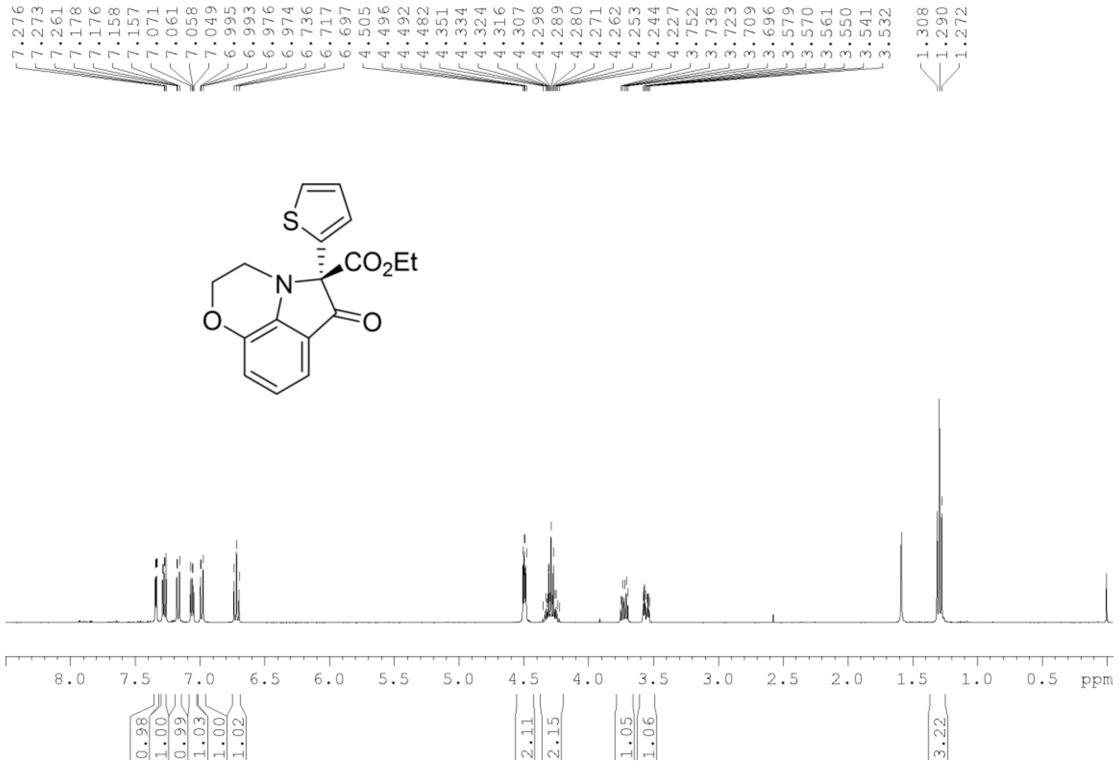

^{1}H NMR (400 MHz, $CDCl_3$) of **4Gq**

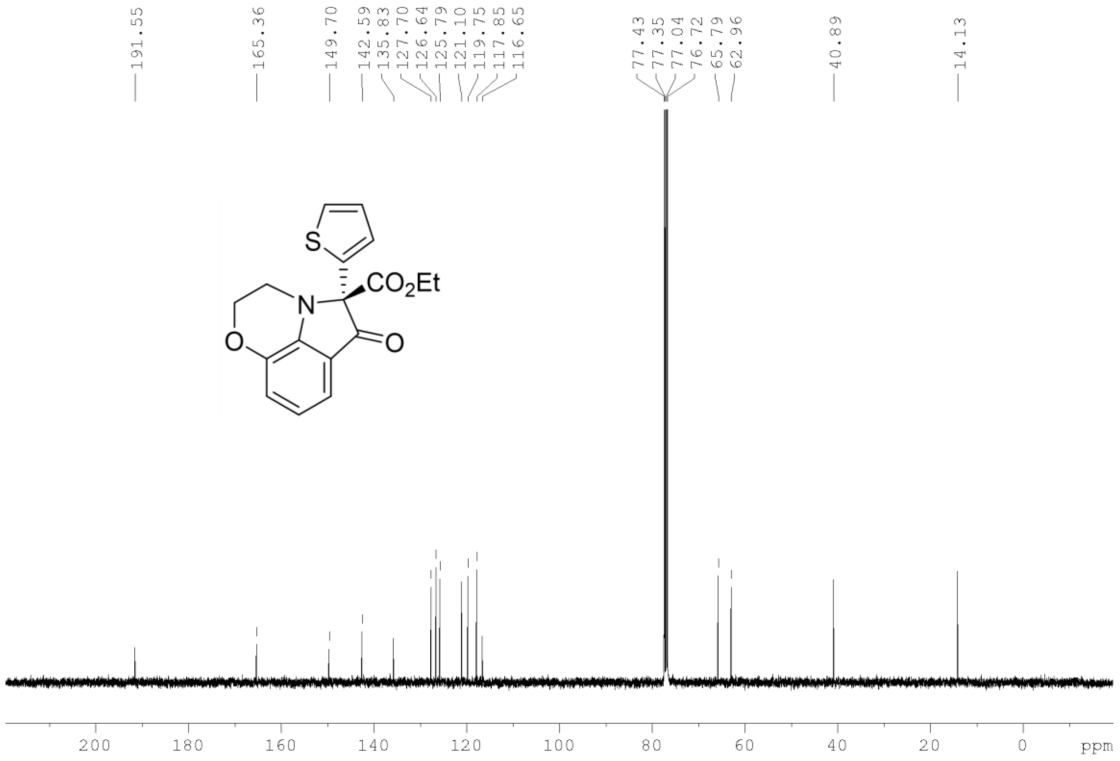

^{13}C NMR (100 MHz, $CDCl_3$) of **4Gq**

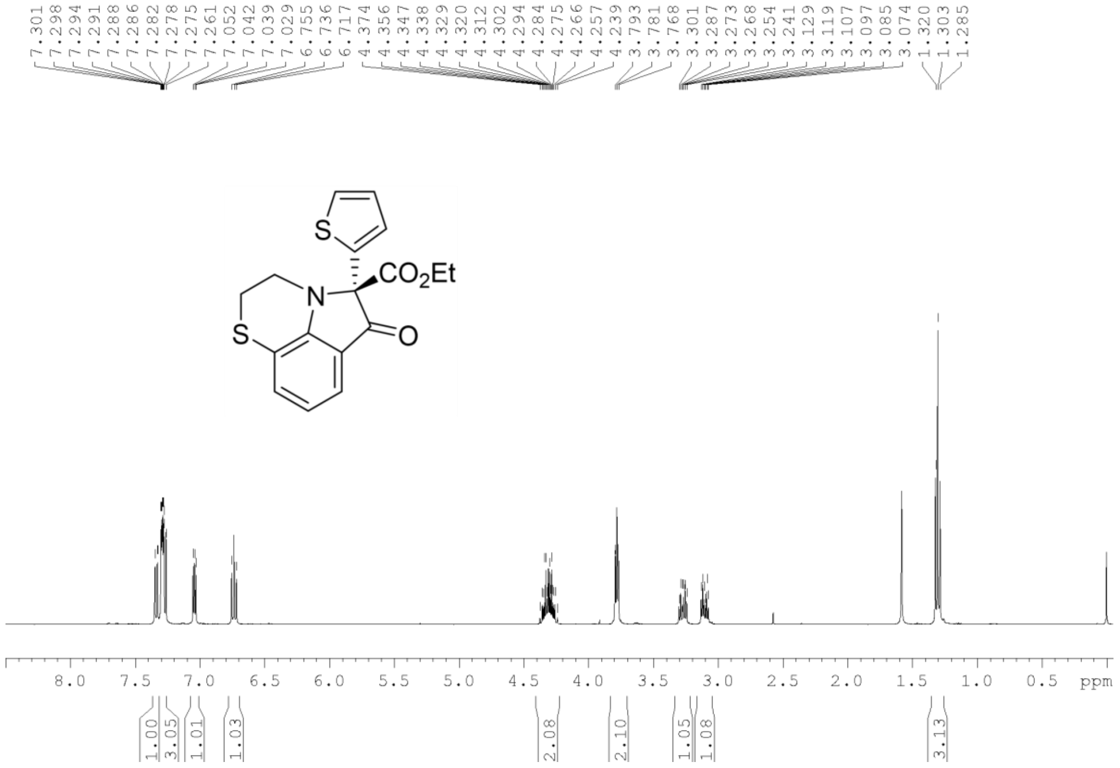

^{1}H NMR (400 MHz, $CDCl_3$) of **4Hq**

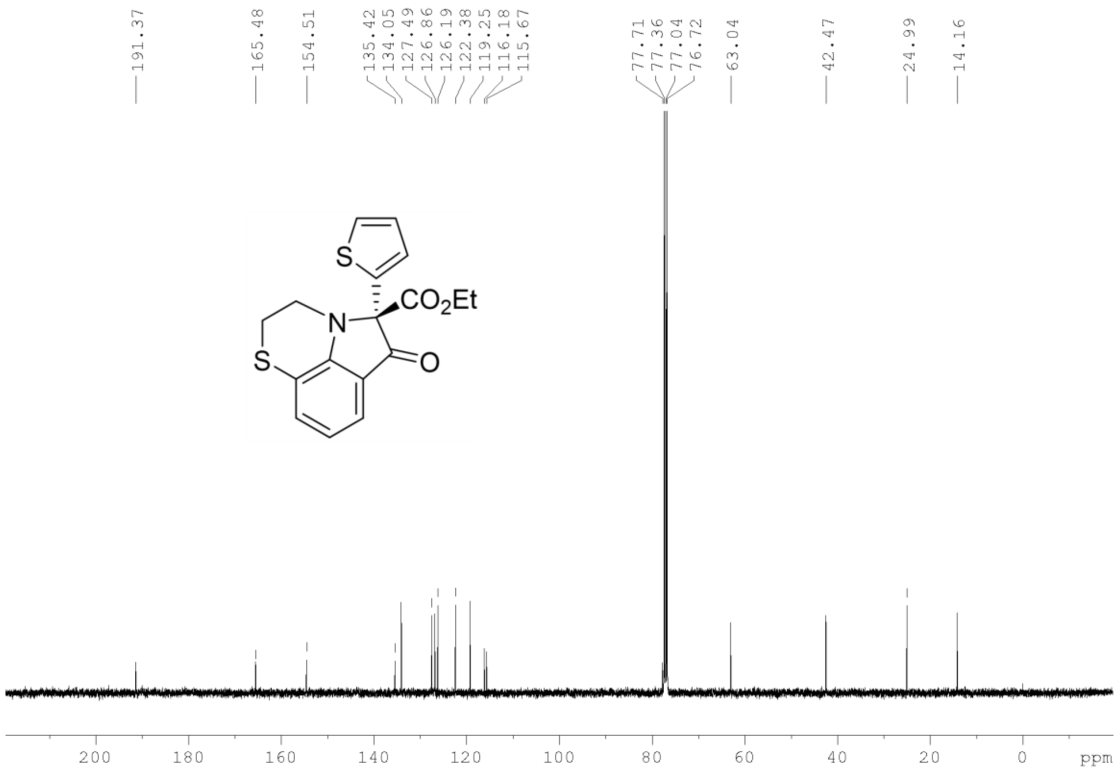

^{13}C NMR (100 MHz, CDCl$_3$) of **4Hq**

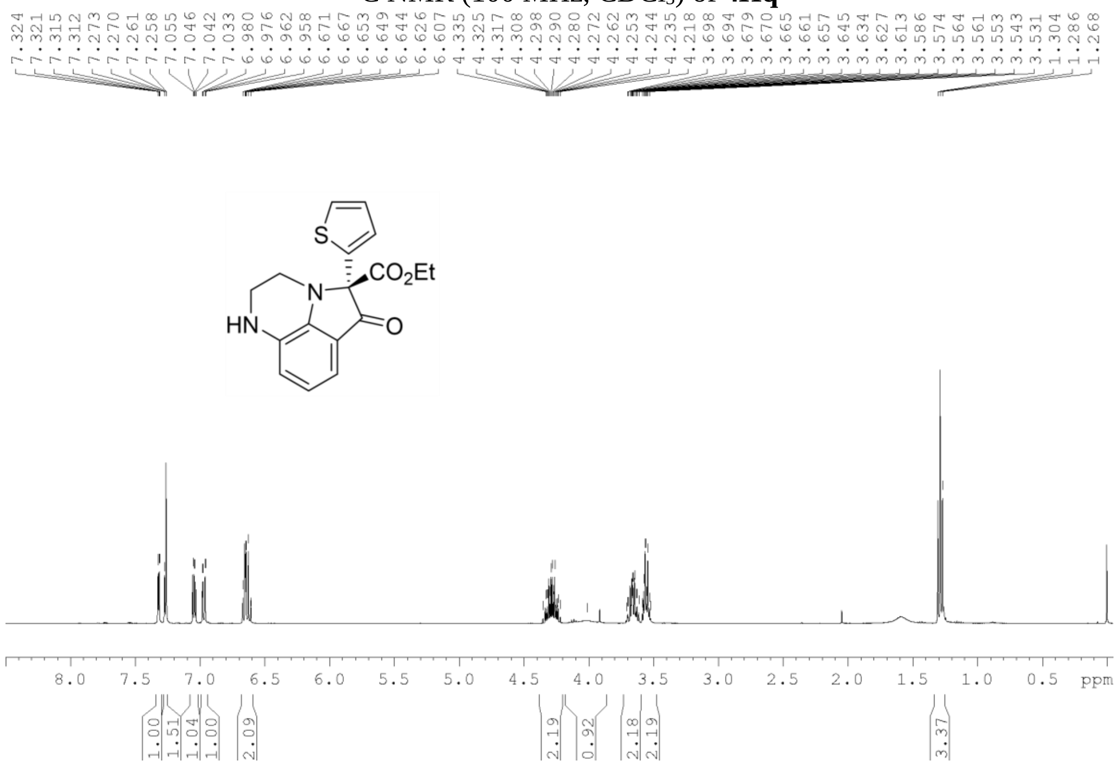

^{1}H NMR (400 MHz, CDCl$_3$) of **4Iq**

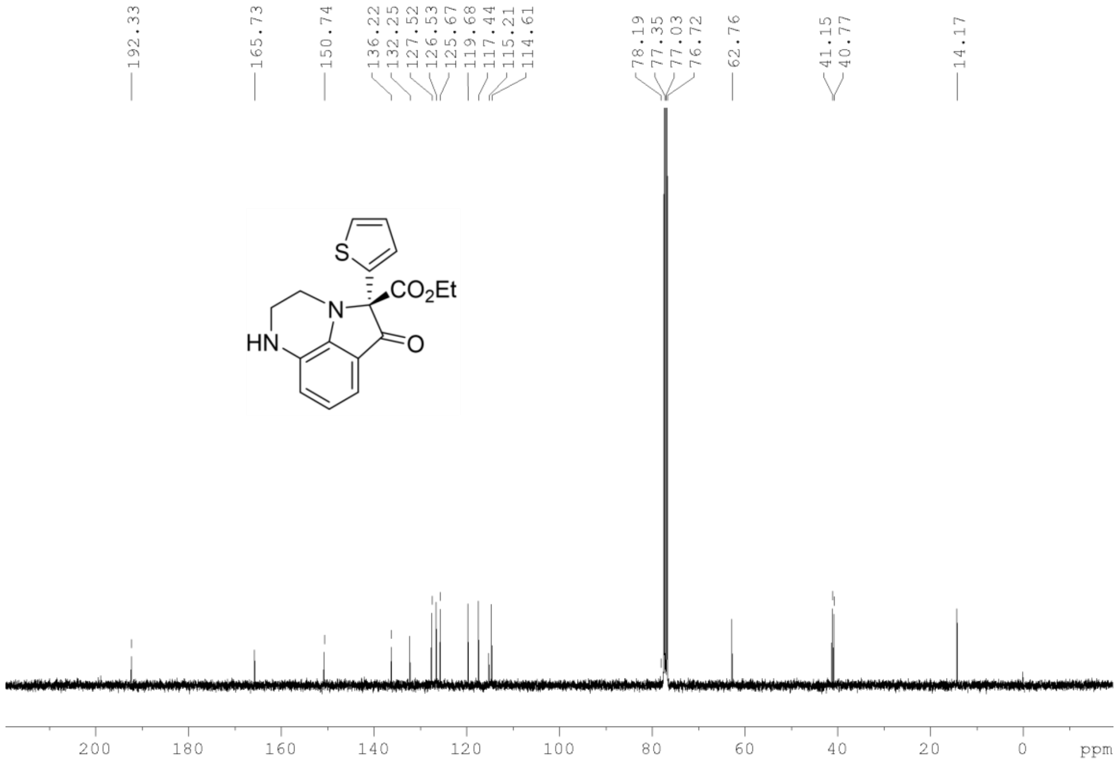

^{13}C NMR (100 MHz, CDCl$_3$) of **4Iq**

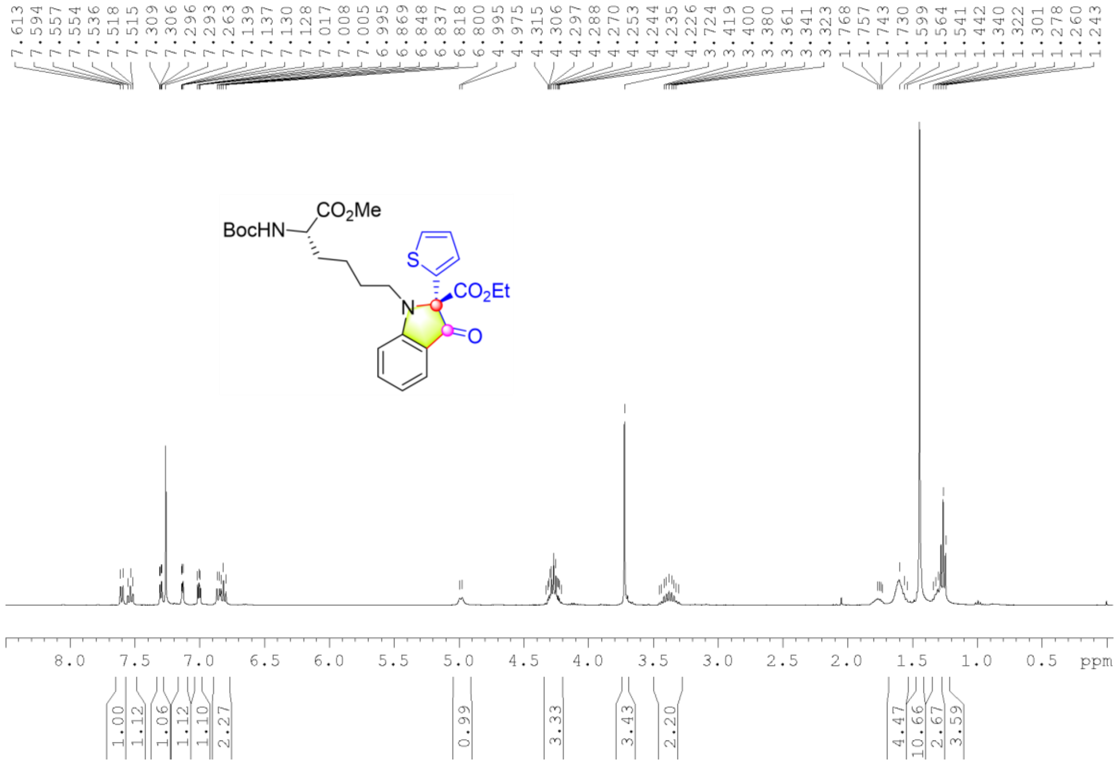

^{1}H NMR (400 MHz, CDCl$_3$) of **4Jq**

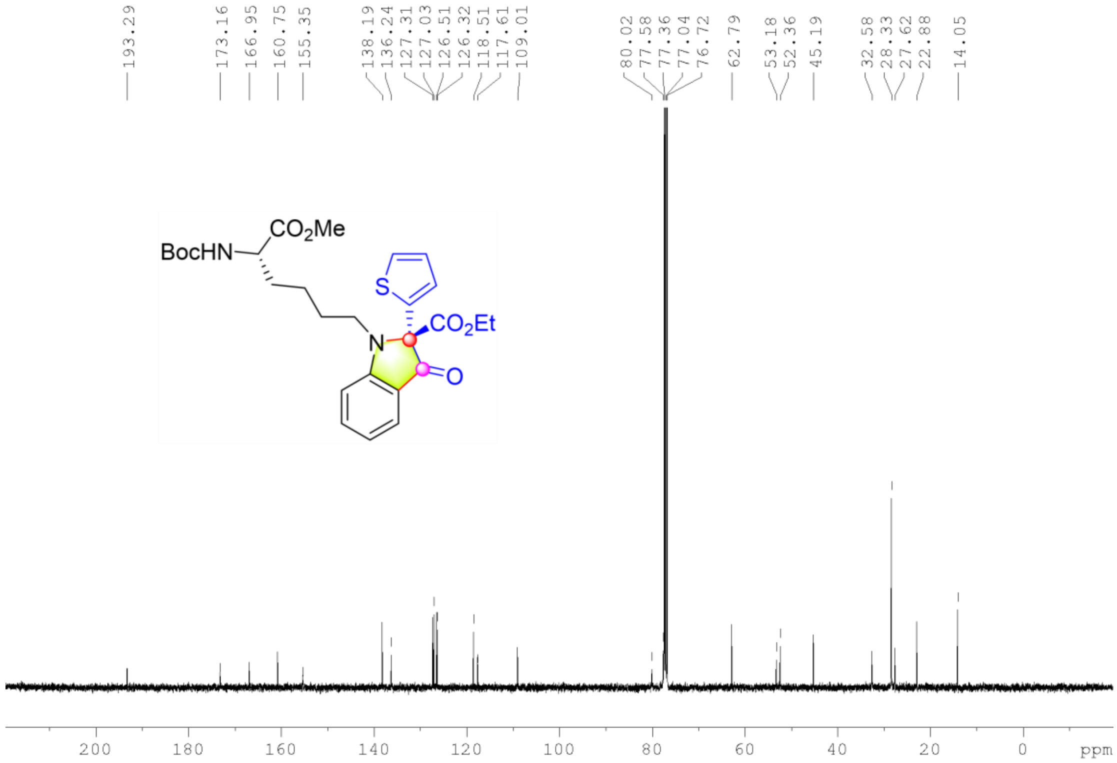

^{13}C NMR (100 MHz, $CDCl_3$) of **4Jq**

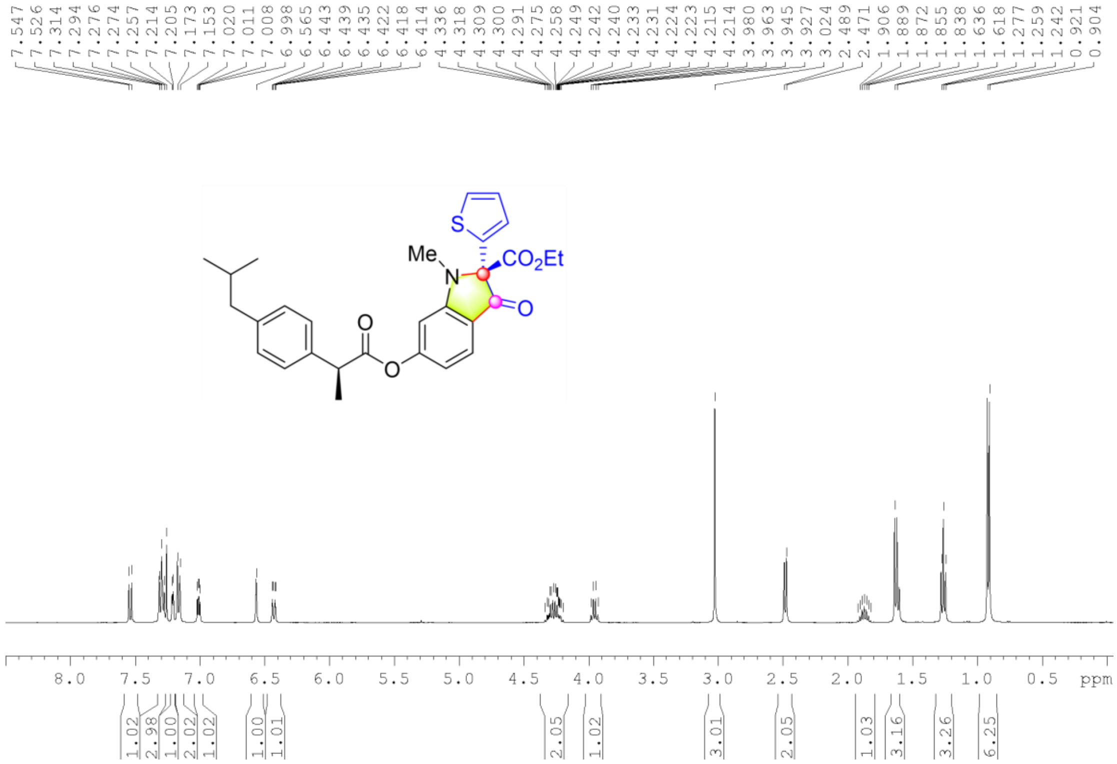

^{1}H NMR (400 MHz, $CDCl_3$) of **4Kq**

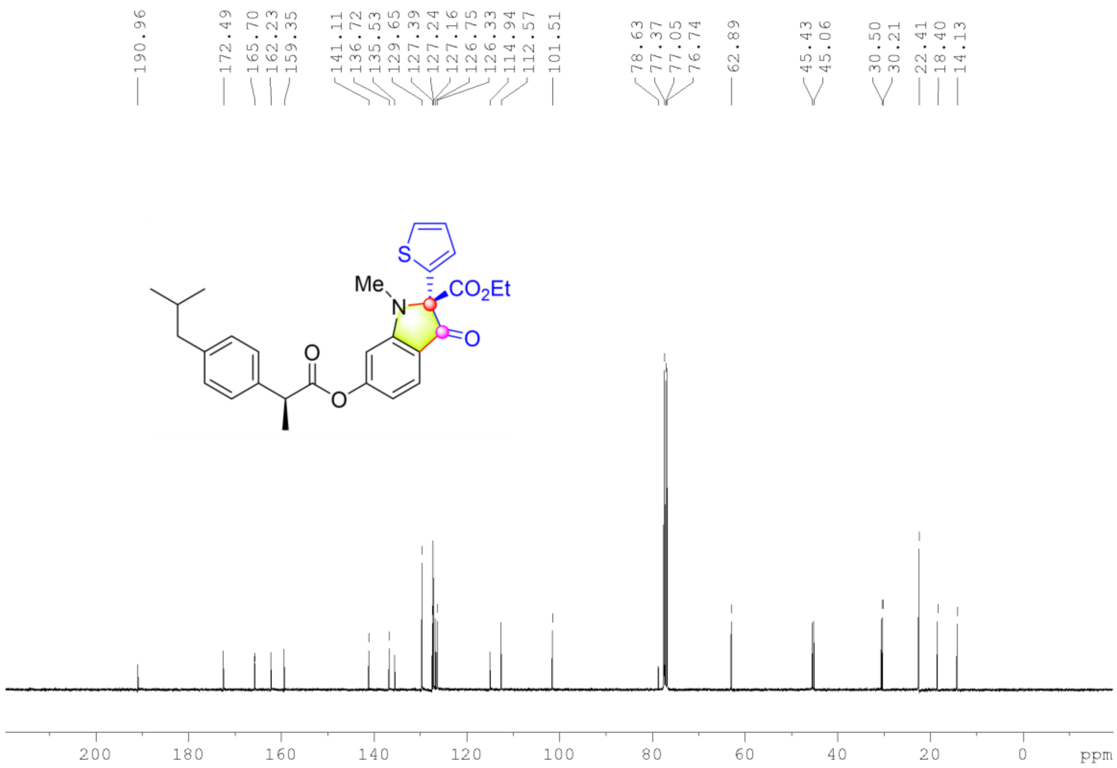

^{13}C NMR (100 MHz, $CDCl_3$) of **4Kq**

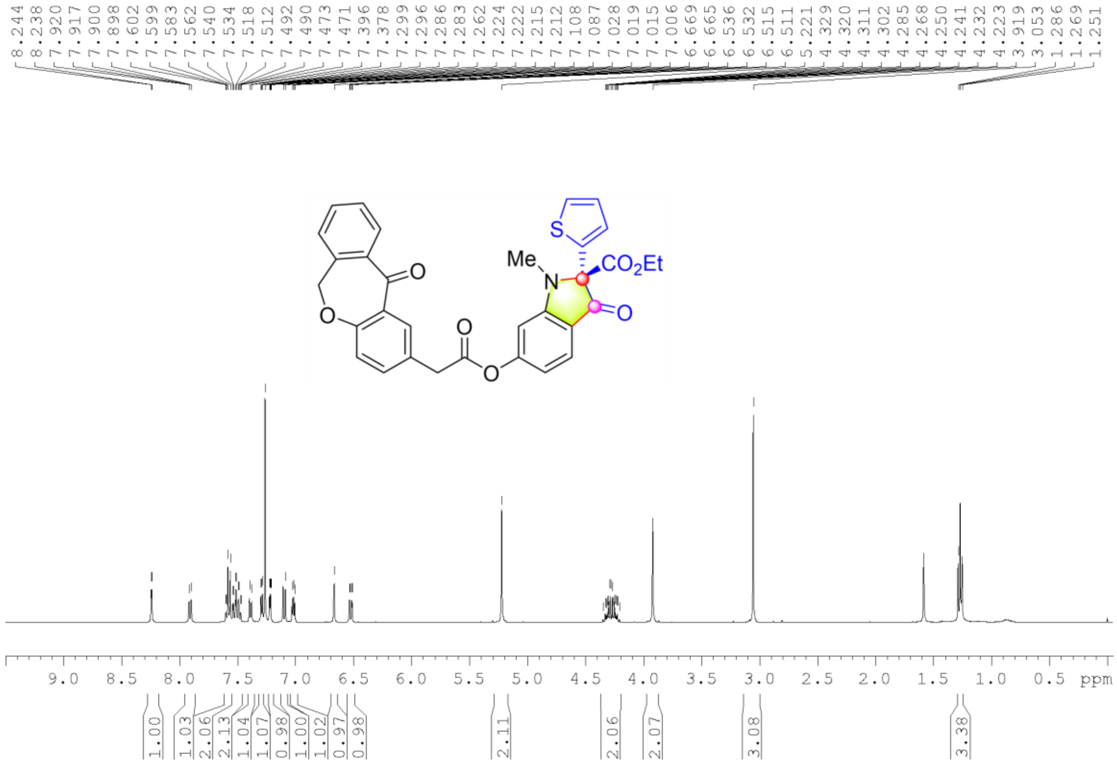

^{1}H NMR (400 MHz, $CDCl_3$) of **4Lq**

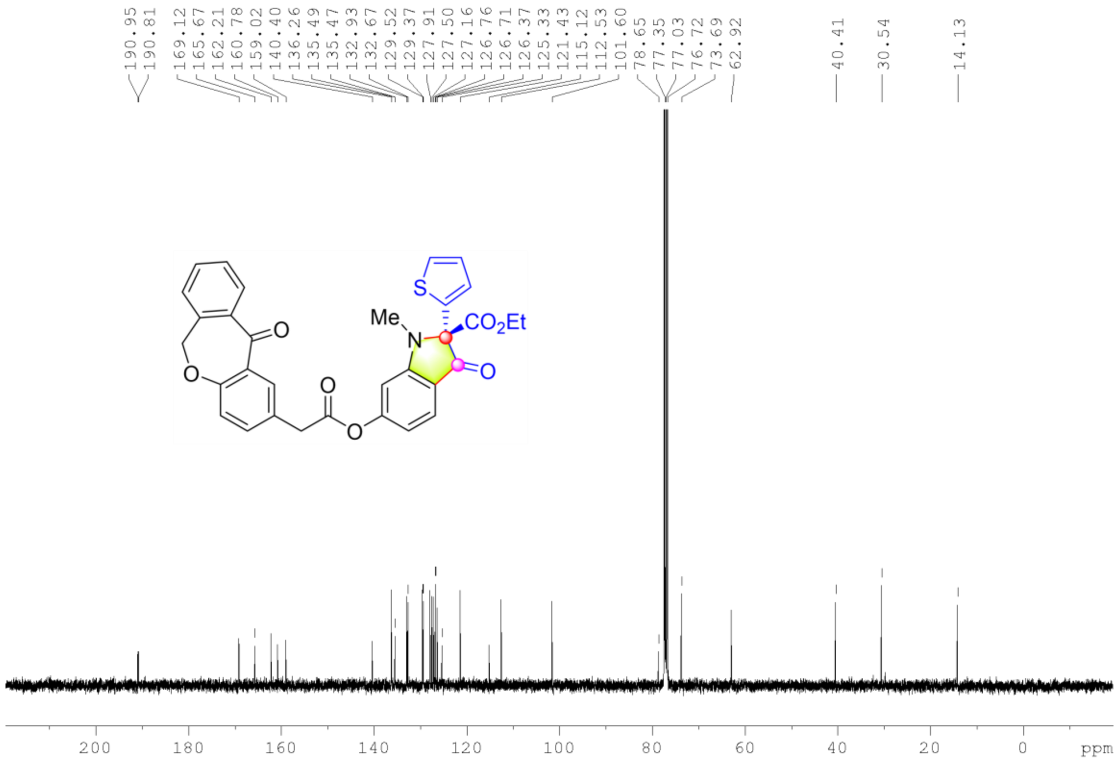

^{13}C NMR (100 MHz, $CDCl_3$) of **4Lq**

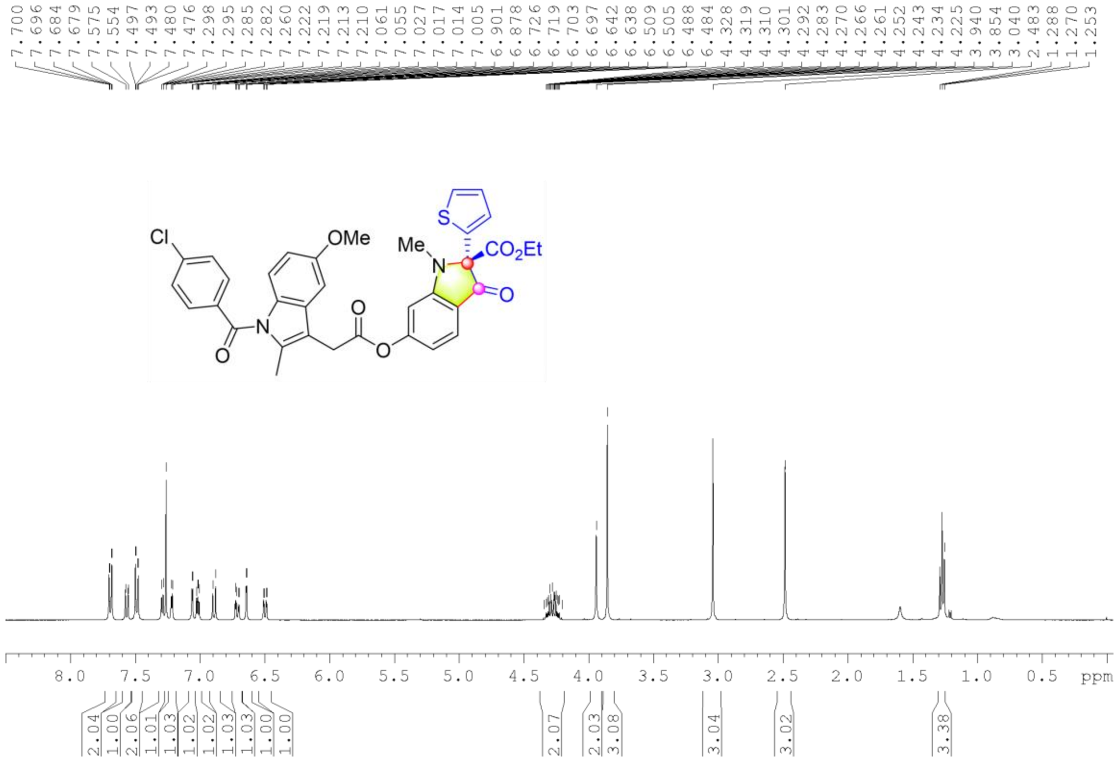

^{1}H NMR (400 MHz, $CDCl_3$) of **4Mq**

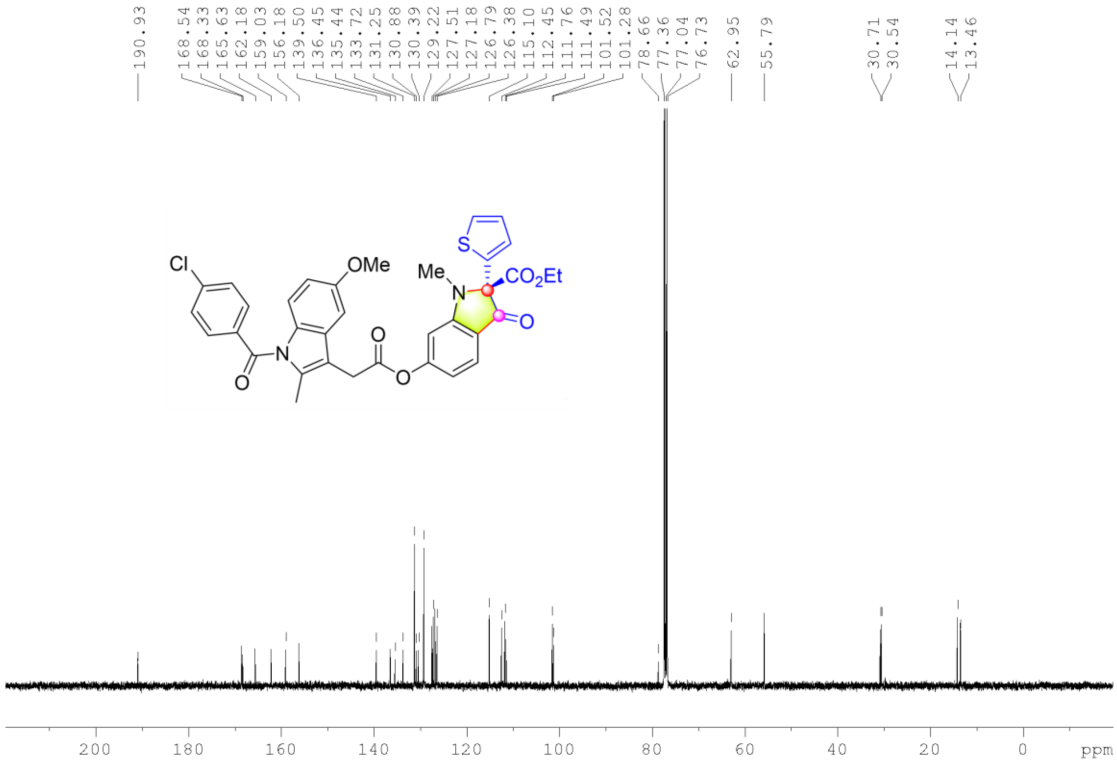

^{13}C NMR (100 MHz, CDCl$_3$) of **4Mq**

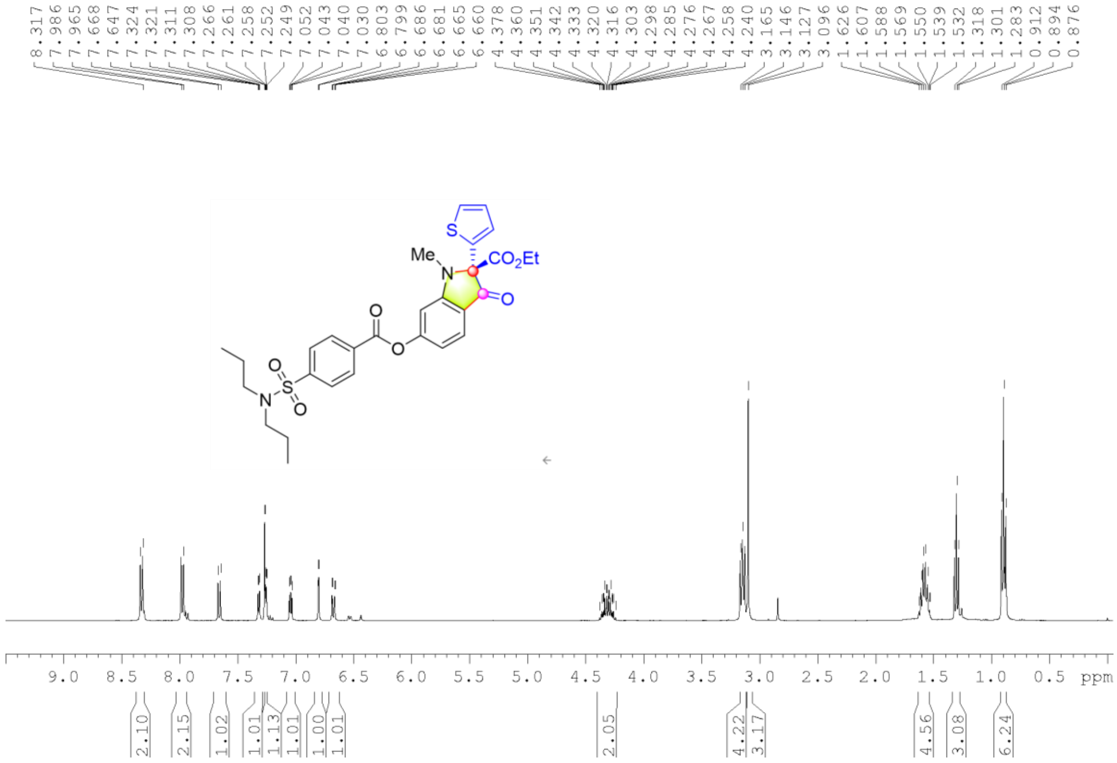

^{1}H NMR (400 MHz, CDCl$_3$) of **4Nq**

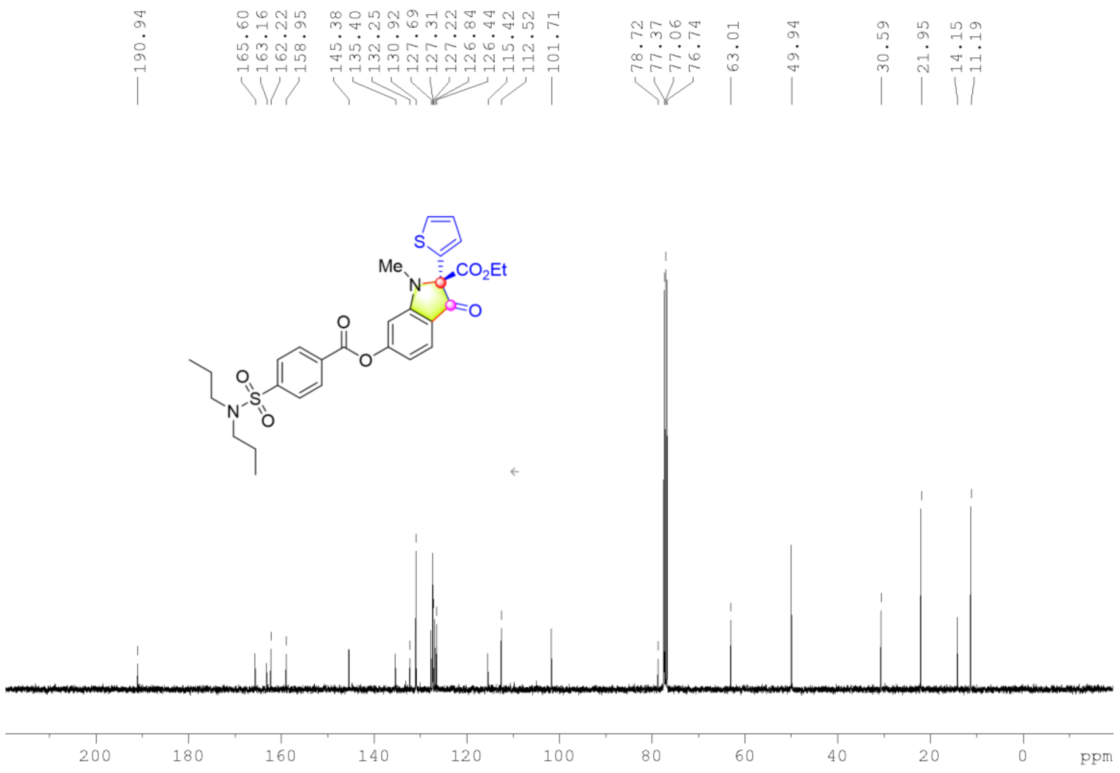

^{13}C NMR (100 MHz, $CDCl_3$) of **4Nq**

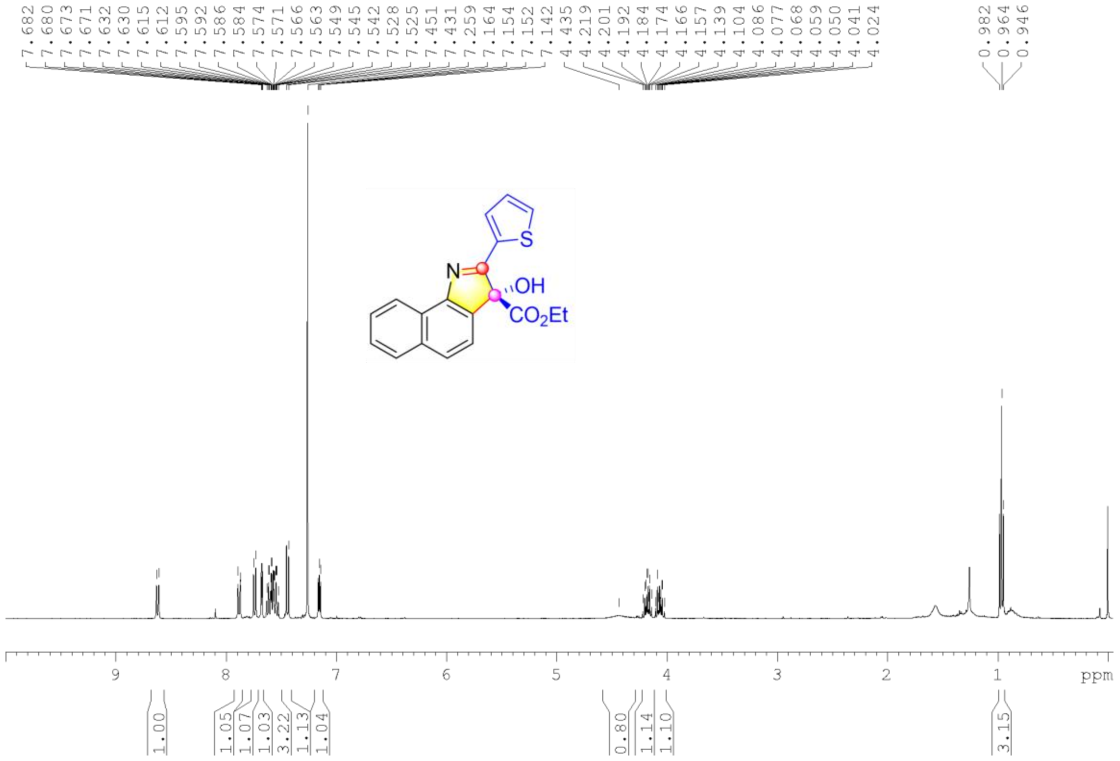

^{1}H NMR (400 MHz, $CDCl_3$) of **3aq**

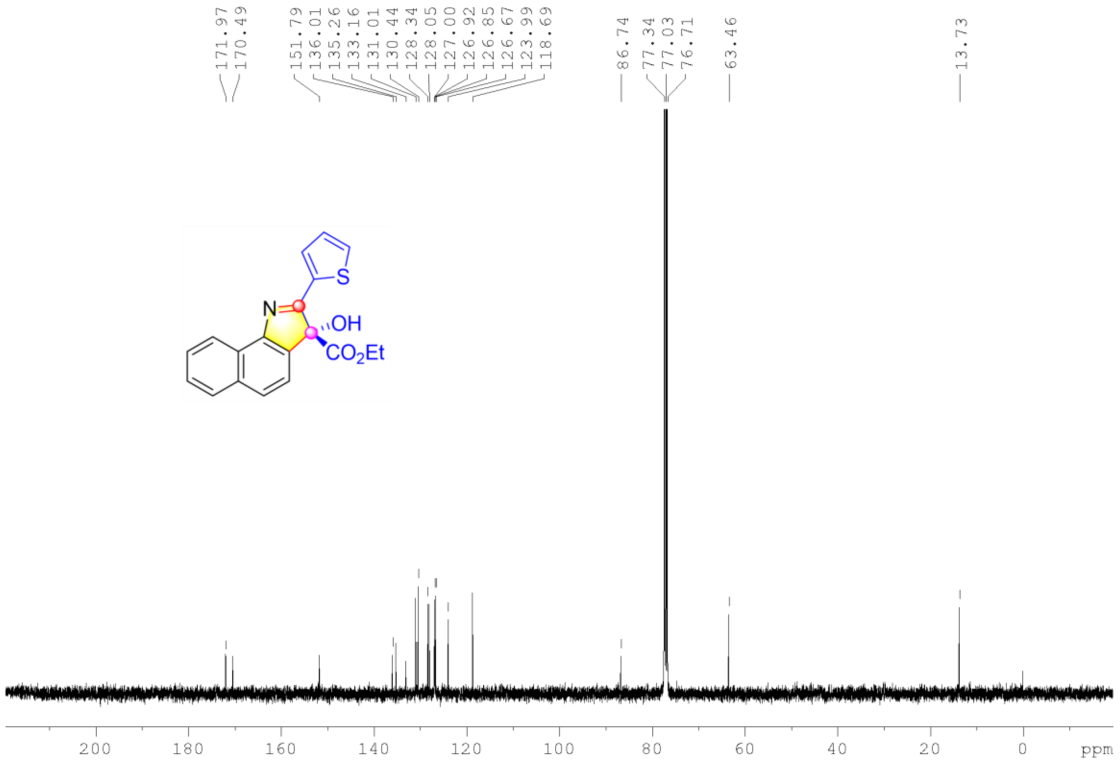

^{13}C NMR (100 MHz, $CDCl_3$) of **3aq**

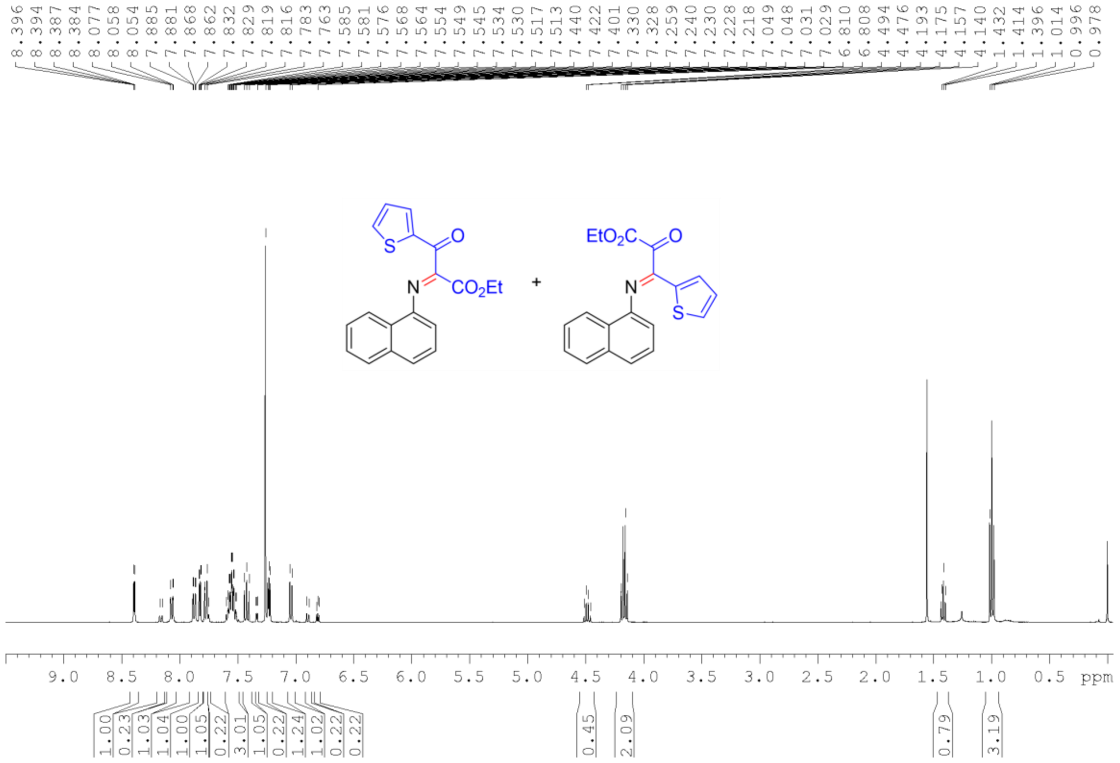

^{1}H NMR (400 MHz, $CDCl_3$) of **7**

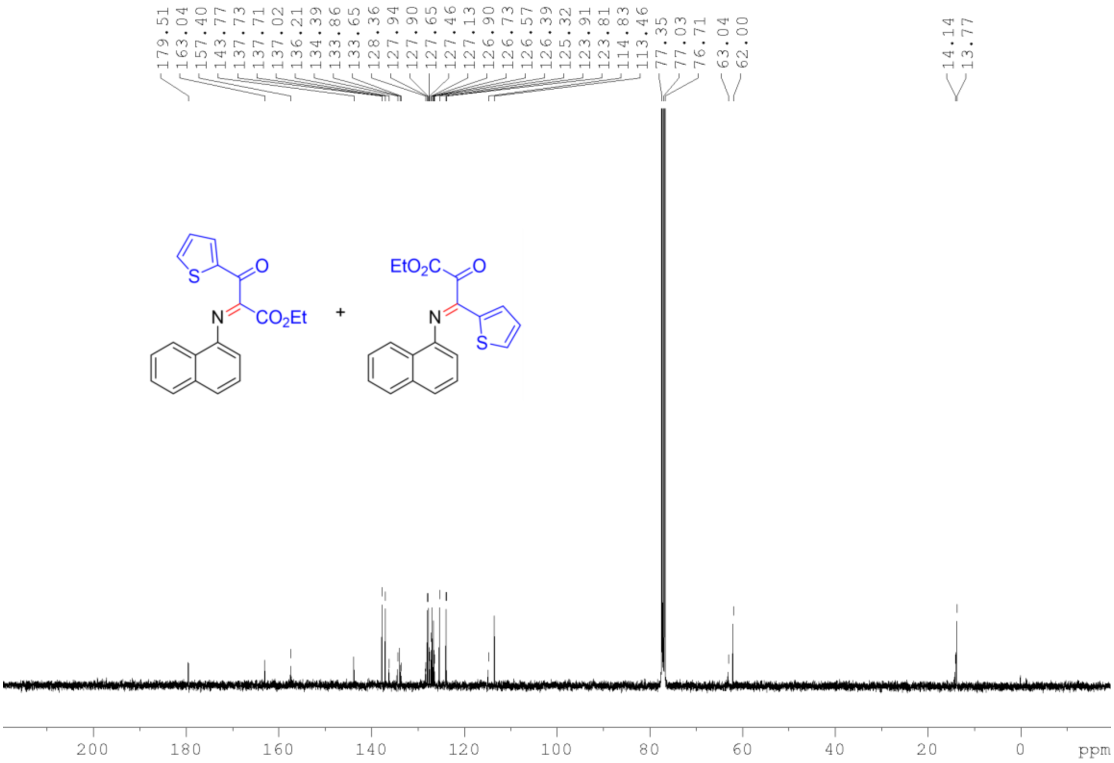

^{13}C NMR (100 MHz, CDCl$_3$) of **7**

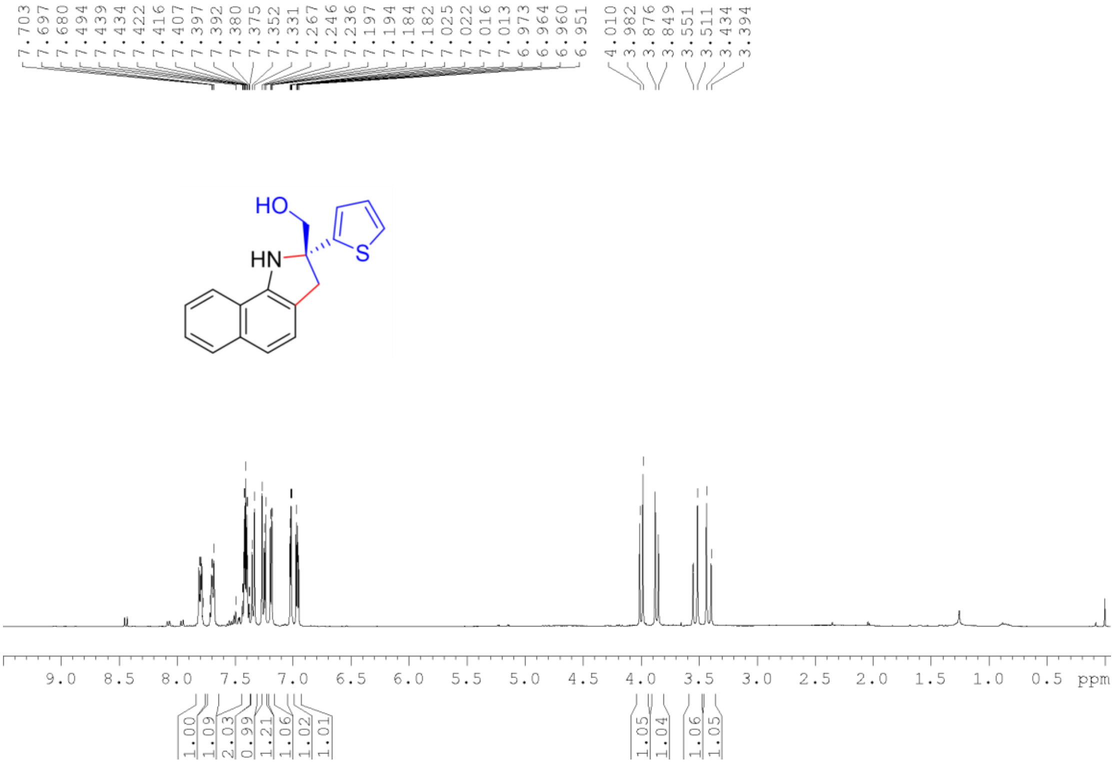

^{1}H NMR (400 MHz, CDCl$_3$) of **8**

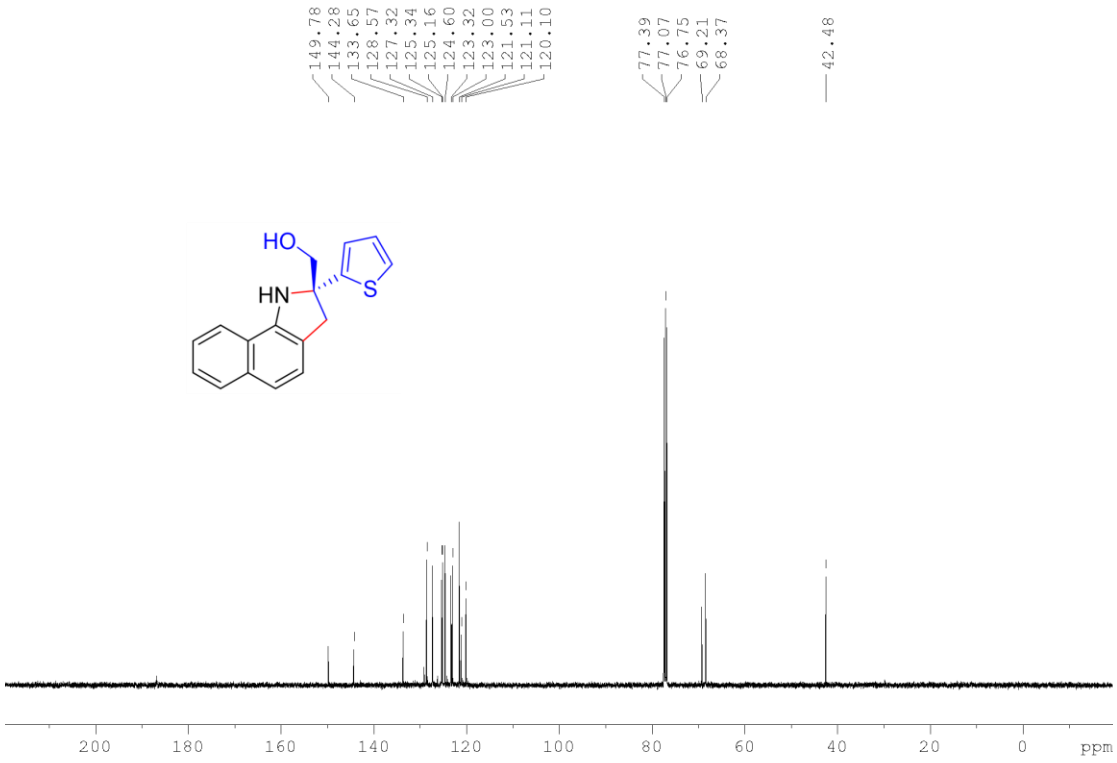

^{13}C NMR (100 MHz, $CDCl_3$) of **8**

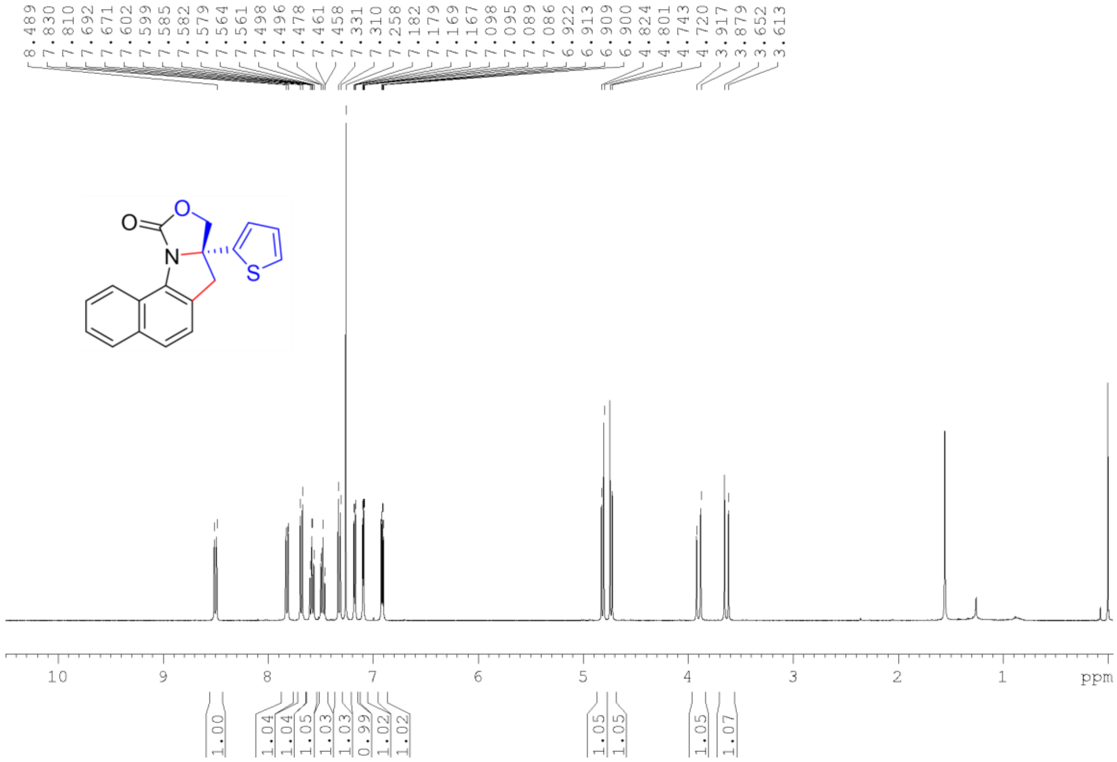

^{1}H NMR (400 MHz, $CDCl_3$) of **9**

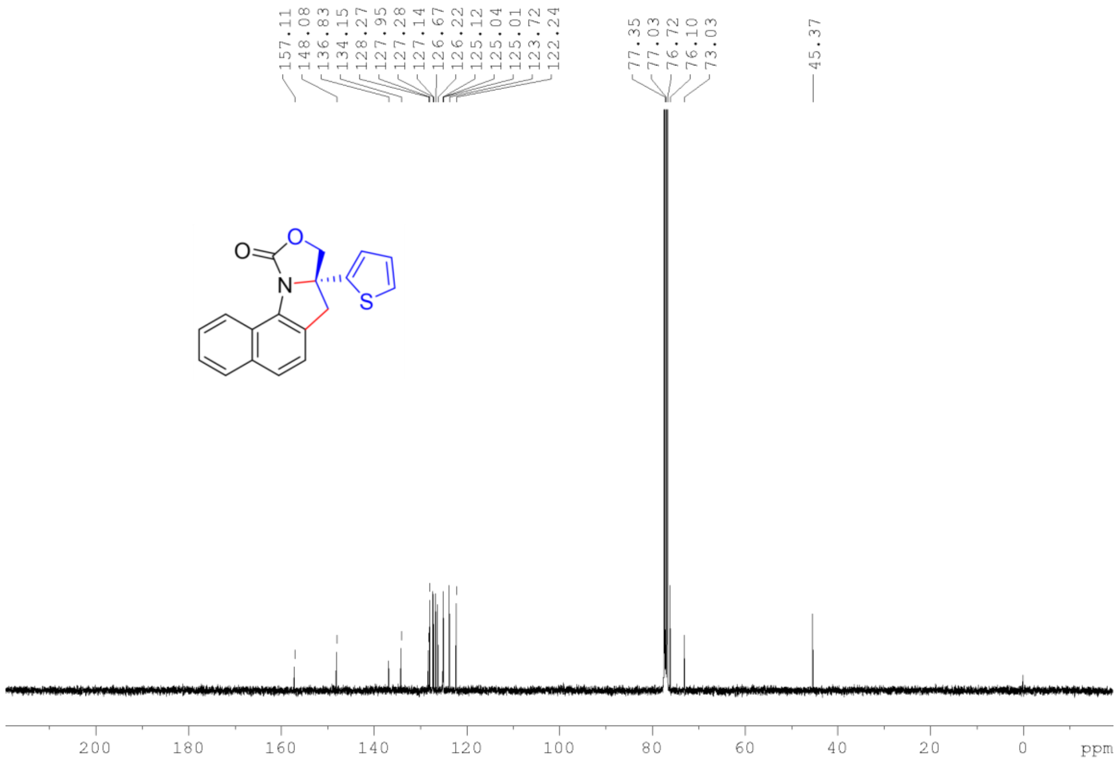

^{13}C NMR (100 MHz, $CDCl_3$) of **9**

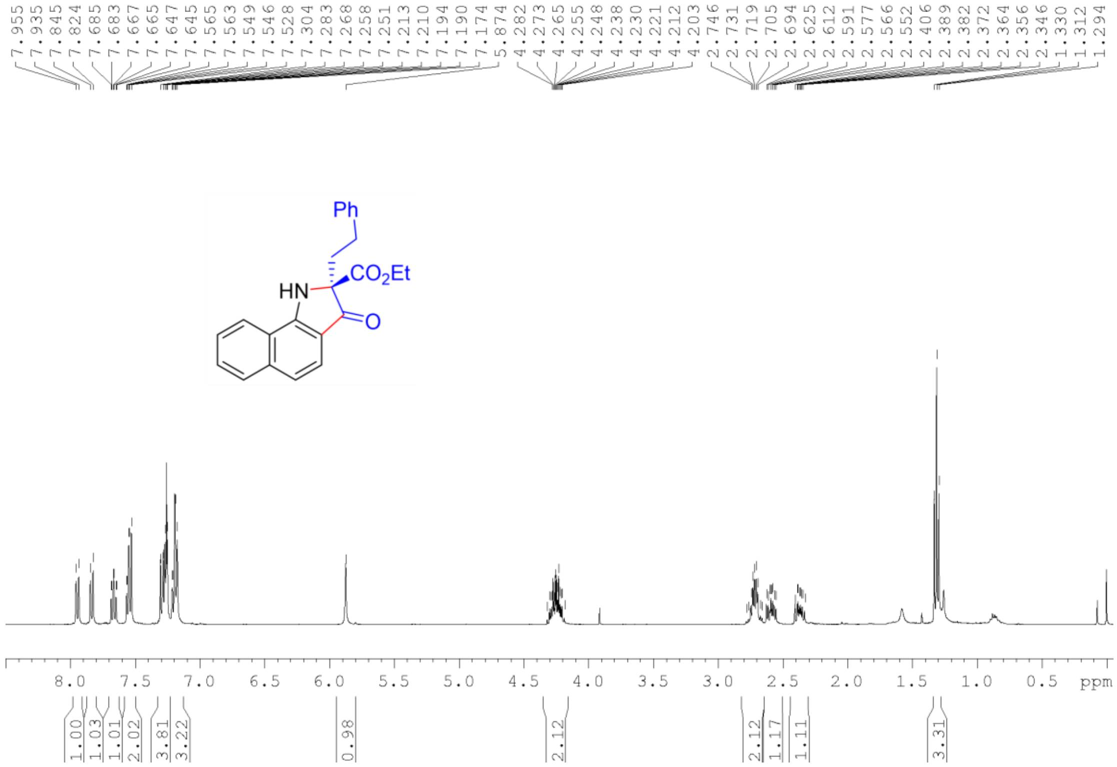

^{1}H NMR (400 MHz, $CDCl_3$) of **10**

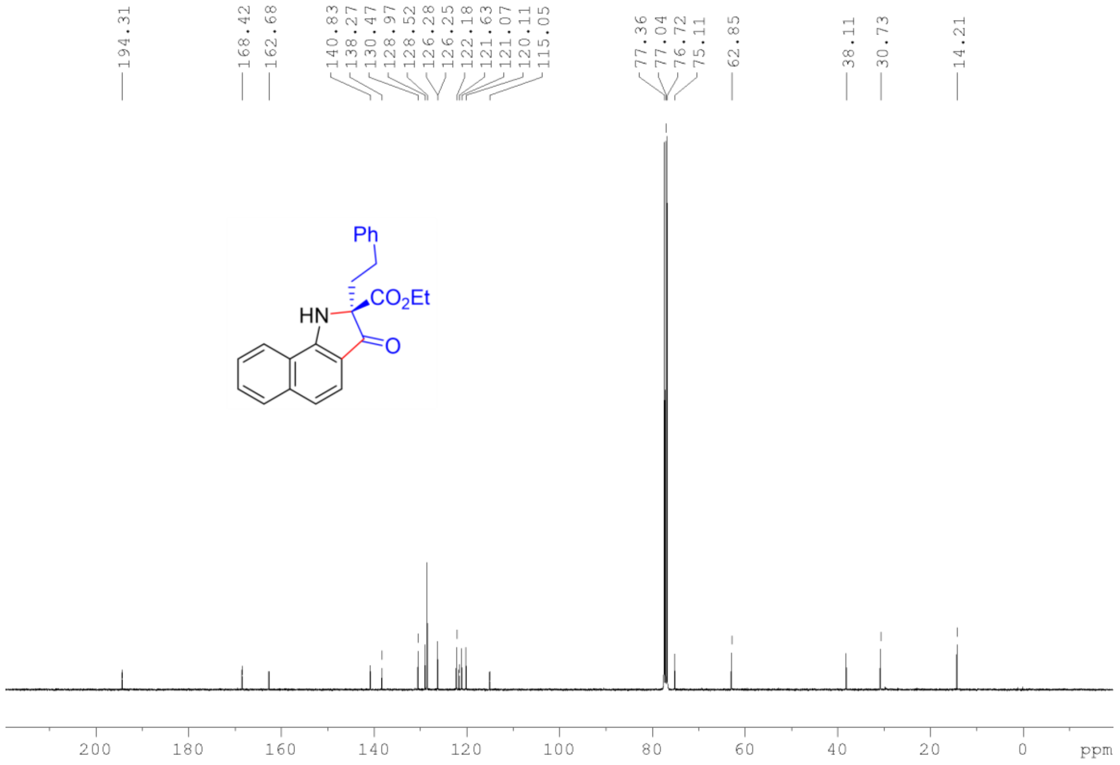

^{13}C NMR (100 MHz, $CDCl_3$) of **10**

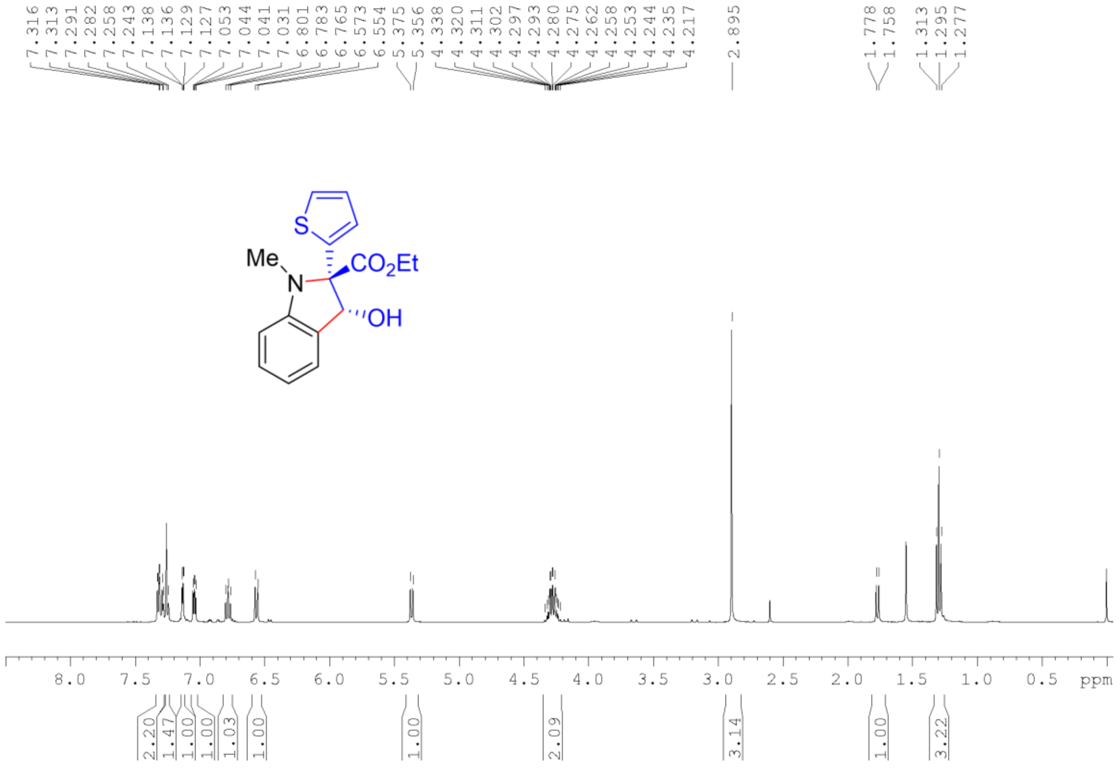

^{1}H NMR (400 MHz, $CDCl_3$) of **11**

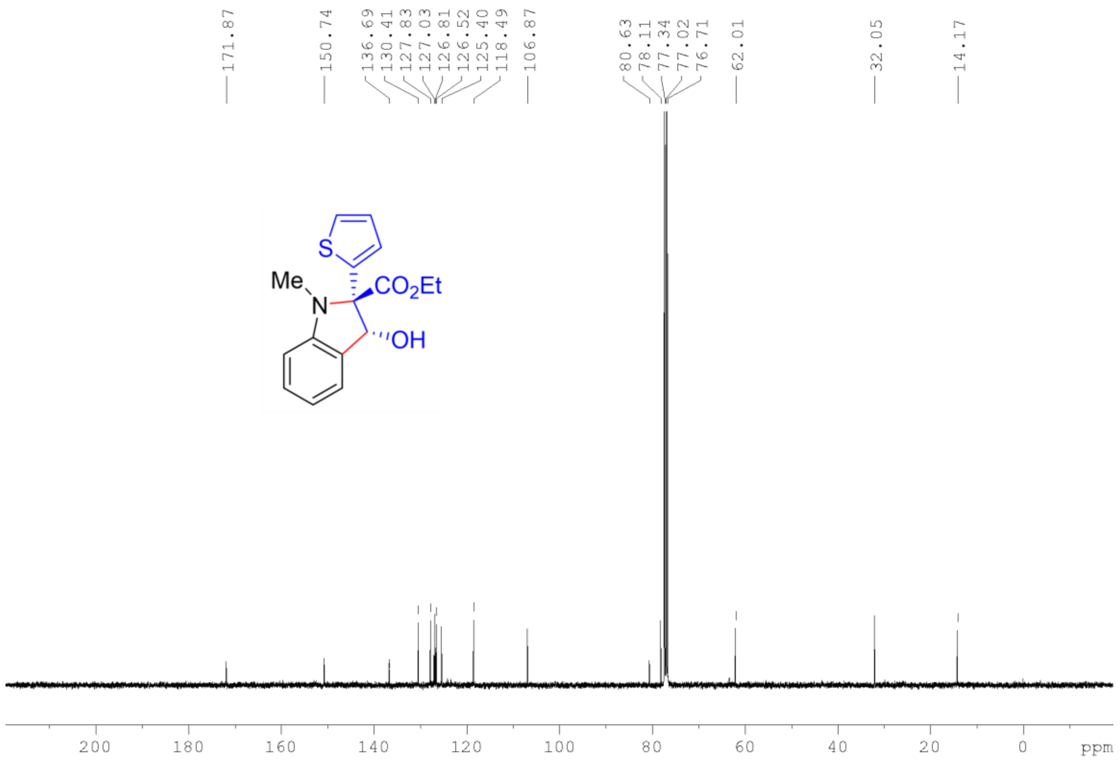

^{13}C NMR (100 MHz, $CDCl_3$) of **11**

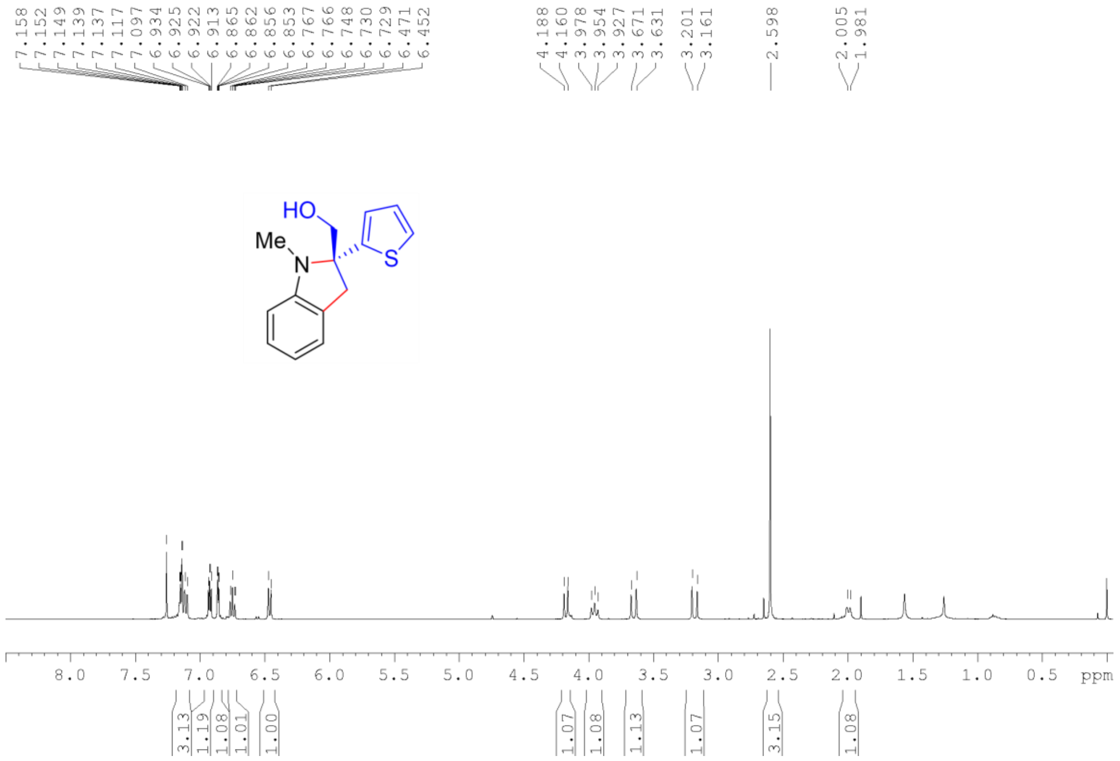

^{1}H NMR (400 MHz, $CDCl_3$) of **12**

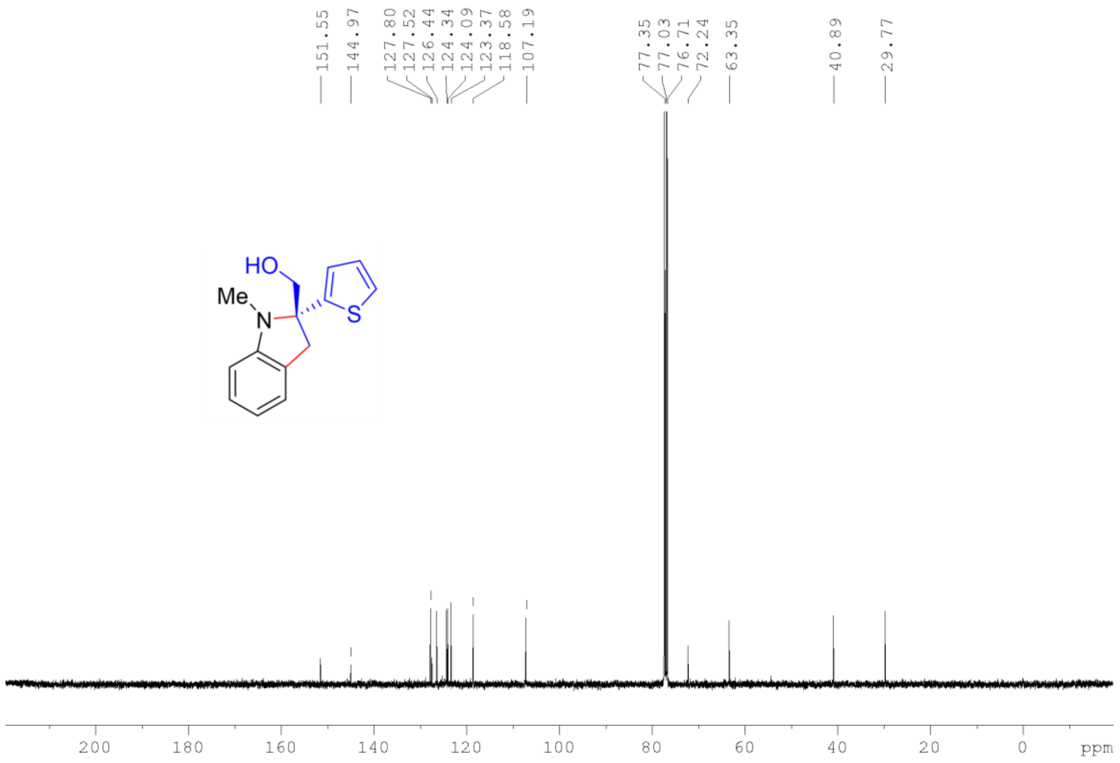

^{13}C NMR (100 MHz, $CDCl_3$) of **12**

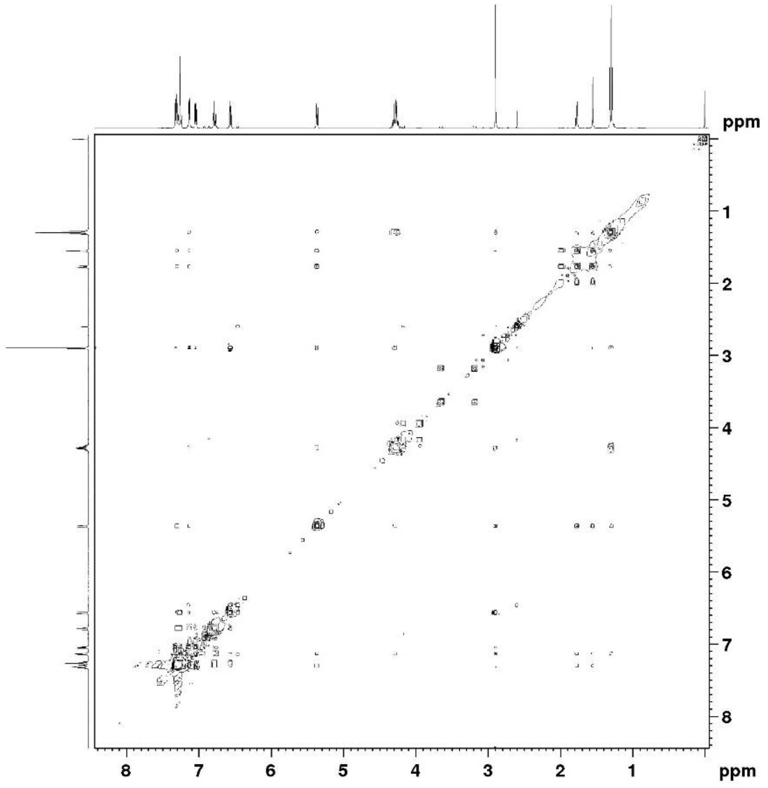

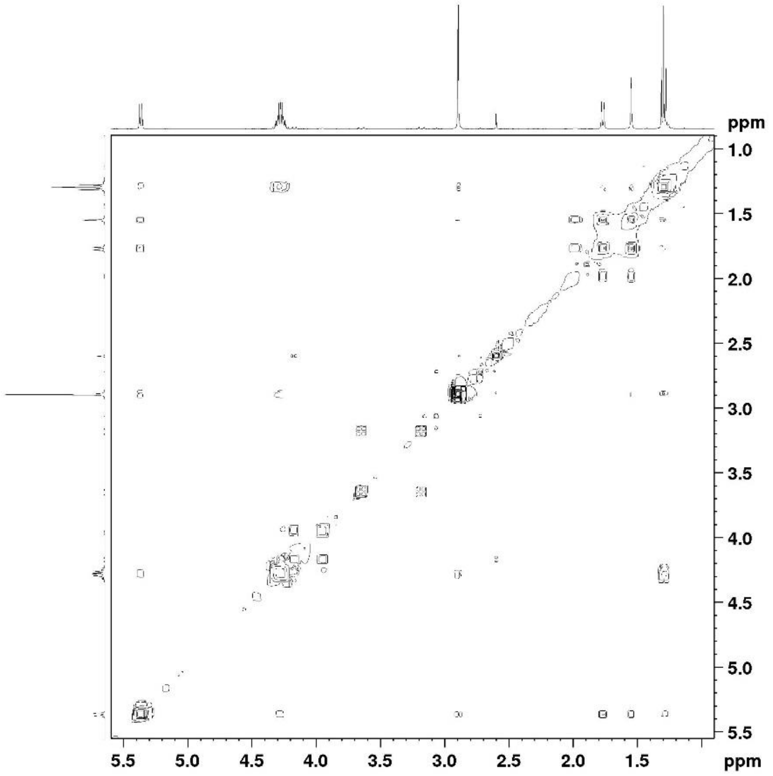

noesy NMR (400 MHz, $CDCl_3$) of **12**

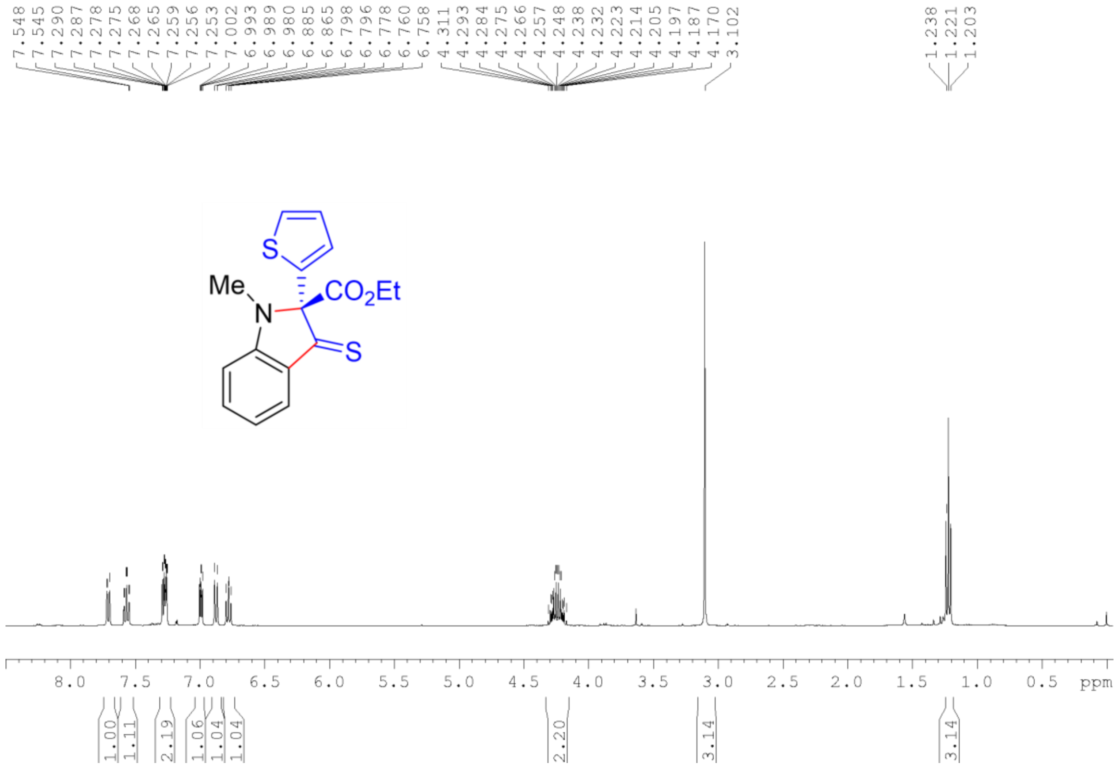

^{1}H NMR (400 MHz, $CDCl_3$) of **13**

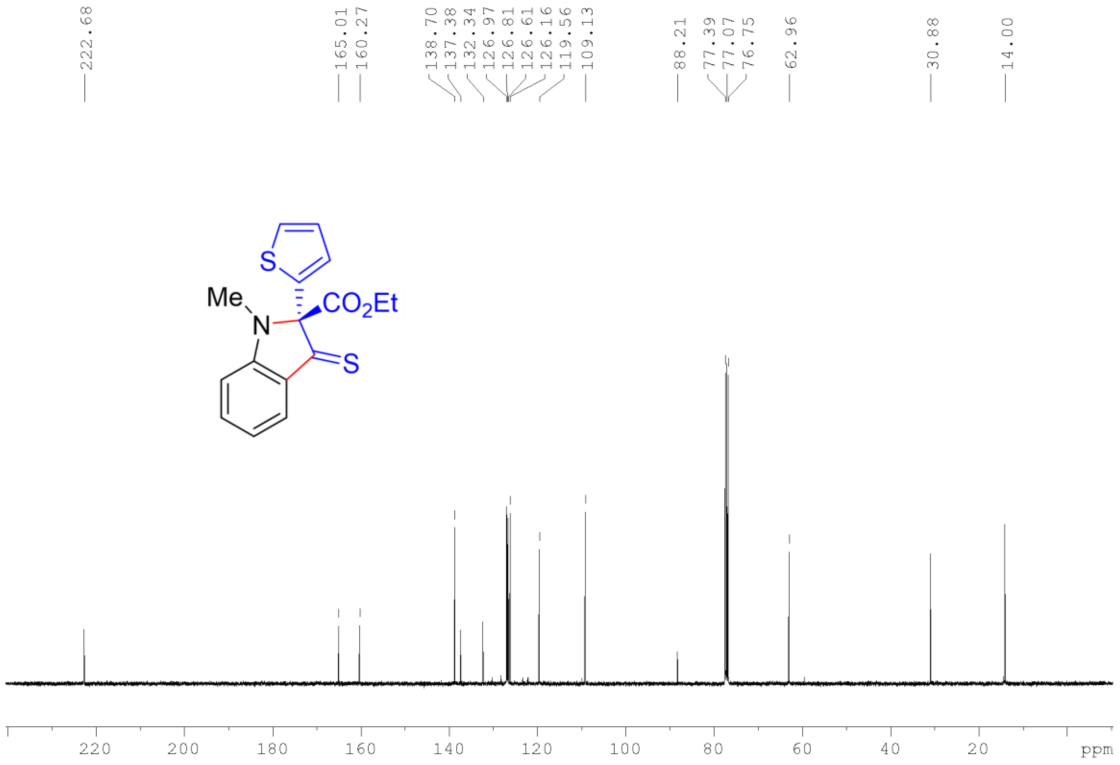

^{13}C NMR (100 MHz, $CDCl_3$) of **13**

Copies of HPLC Spectra

HPLC spectra of **4aa**

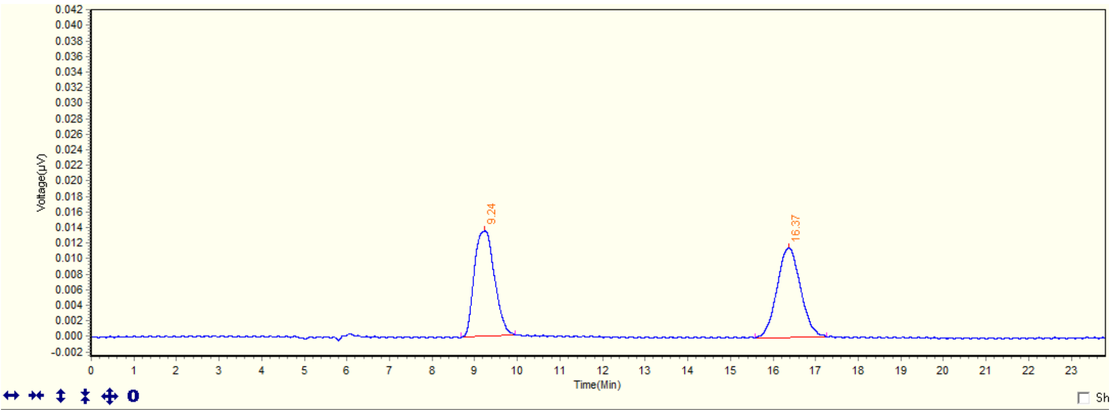

Integration Result | Calculation Result | TimeTable

No.	Retention Time	Peak Area	Peak Height	Peak Area(%)	Peak Width	Peak Type
1	9.24	222741	6781	50.21%	1.265	BB
2	16.37	220901	5750	49.79%	1.667	BB
Total		443,642	12,531	100.00%		

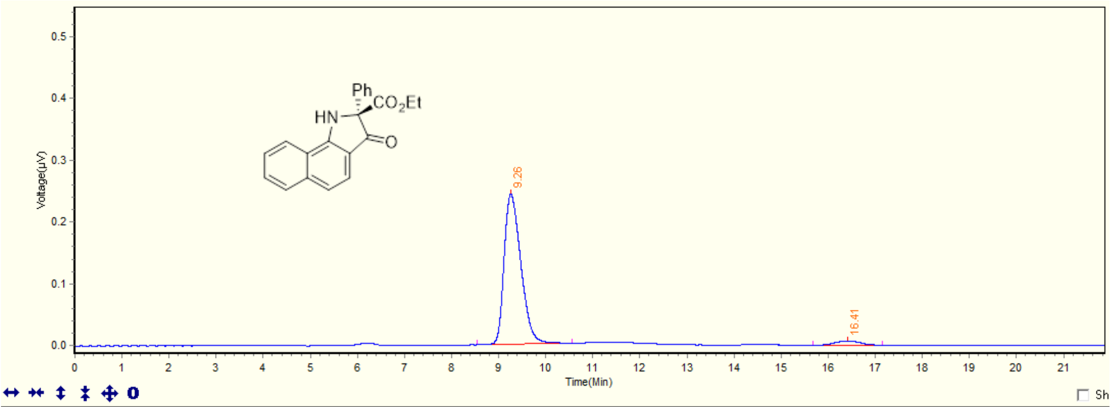

Integration Result | Calculation Result | TimeTable

No.	Retention Time	Peak Area	Peak Height	Peak Area(%)	Peak Width	Peak Type
1	9.26	3013727	121445	95.79%	2.009	BB
2	16.41	132328	3519	4.21%	1.483	BB
Total		3,146,055	124,964	100.00%		

HPLC spectra of **4ab**

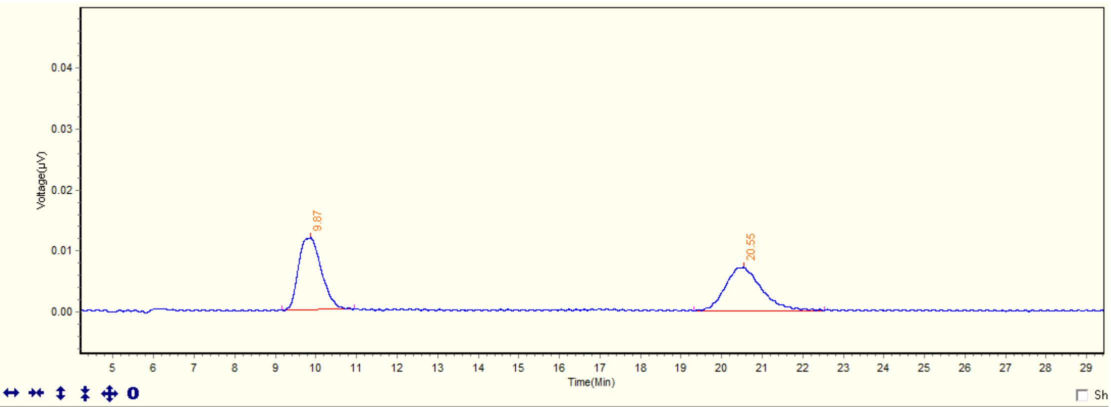

Integration Result | Calculation Result | TimeTable

No.	Retention Time	Peak Area	Peak Height	Peak Area(%)	Peak Width	Peak Type
1	9.87	229385	5877	50.92%	1.785	BB
2	20.55	221054	3550	49.08%	3.217	BB
Total		450,439	9,427	100.00%		

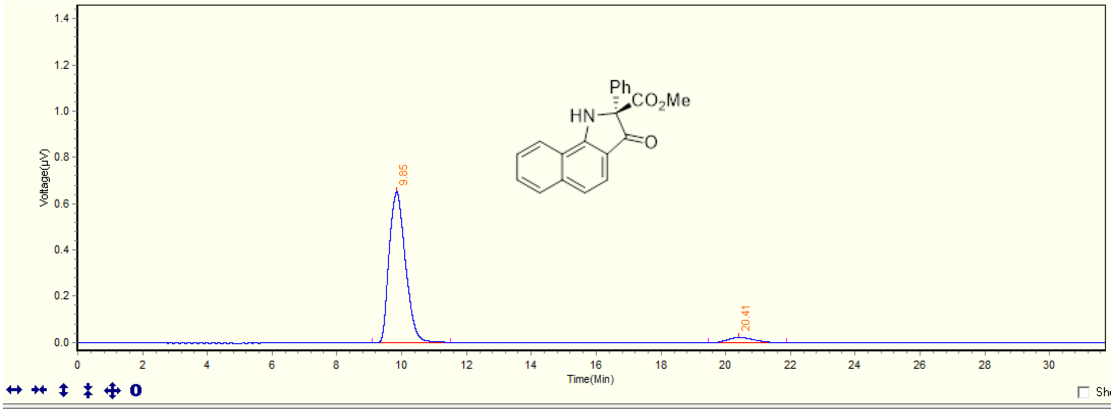

Integration Result | Calculation Result | TimeTable

No.	Retention Time	Peak Area	Peak Height	Peak Area(%)	Peak Width	Peak Type
1	9.85	11400218	326764	94.27%	2.415	BB
2	20.41	692999	11888	5.73%	2.415	BB
Total		12,093,217	338,652	100.00%		

HPLC spectra of **4ac**

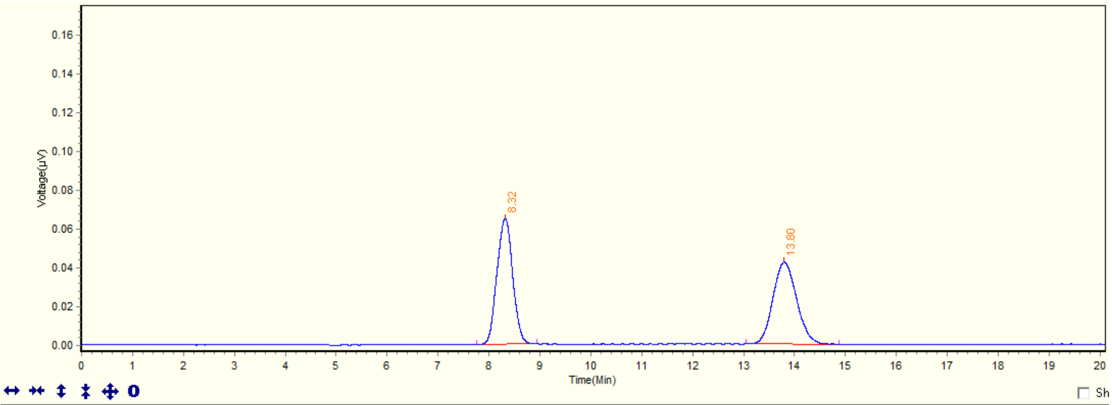

Integration Result | Calculation Result | TimeTable

No.	Retention Time	Peak Area	Peak Height	Peak Area(%)	Peak Width	Peak Type
1	8.32	689852	32193	50.39%	1.183	BB
2	13.80	679177	21093	49.61%	1.824	BB
Total		1,369,029	53,286	100.00%		

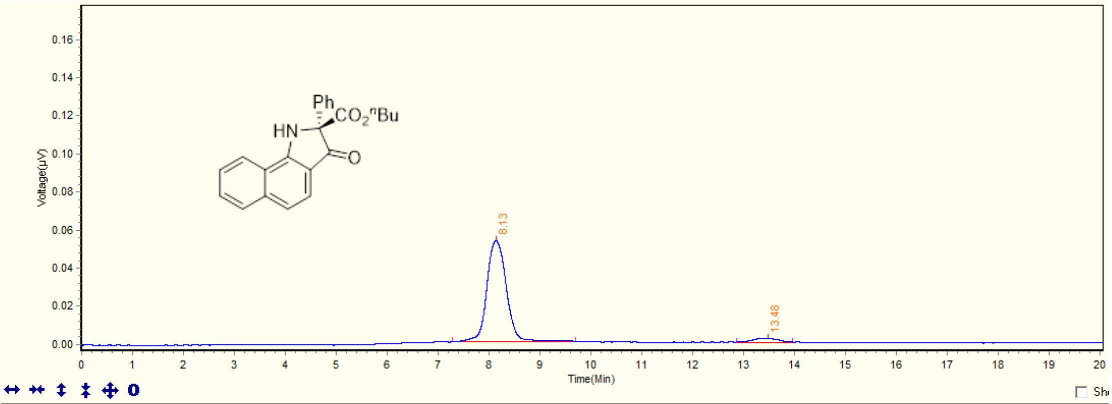

Integration Result | Calculation Result | TimeTable

No.	Retention Time	Peak Area	Peak Height	Peak Area(%)	Peak Width	Peak Type
1	8.13	705212	26633	95.31%	2.424	BB
2	13.48	34737	1069	4.69%	1.104	BB
Total		739,949	27,702	100.00%		

HPLC spectra of **4ad**

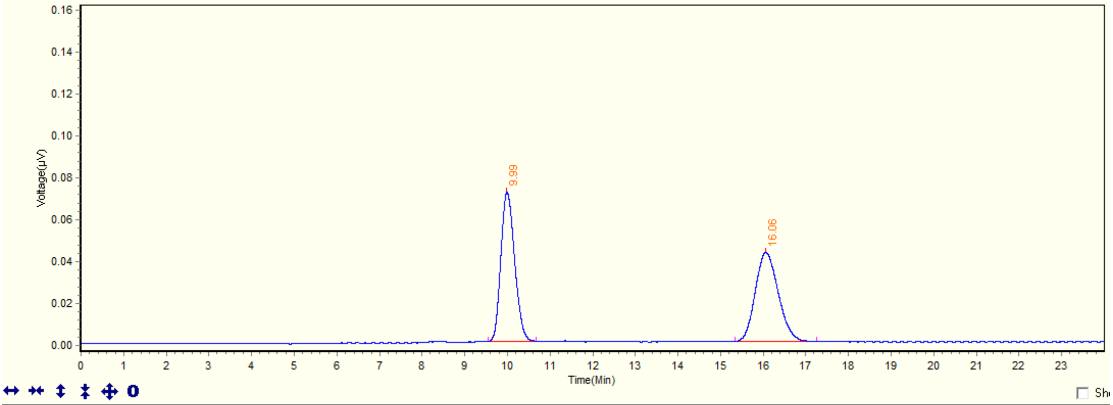

No.	Retention Time	Peak Area	Peak Height	Peak Area(%)	Peak Width	Peak Type
1	9.99	800600	35623	50.23%	1.103	BB
2	16.06	793285	21234	49.77%	1.914	BB
Total		1,593,885	56,857	100.00%		

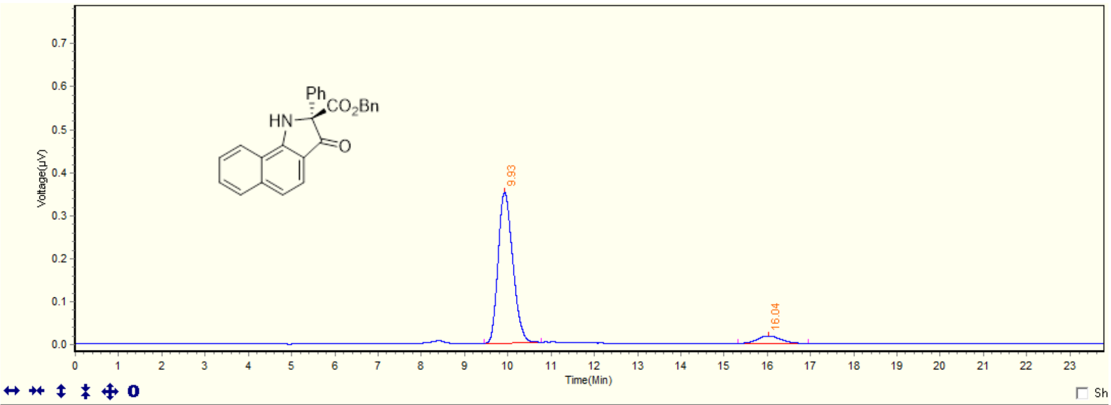

No.	Retention Time	Peak Area	Peak Height	Peak Area(%)	Peak Width	Peak Type
1	9.93	4112306	174964	92.81%	1.327	BB
2	16.04	318800	8562	7.19%	1.619	BB
Total		4,431,106	183,526	100.00%		

HPLC spectra of **4ae**

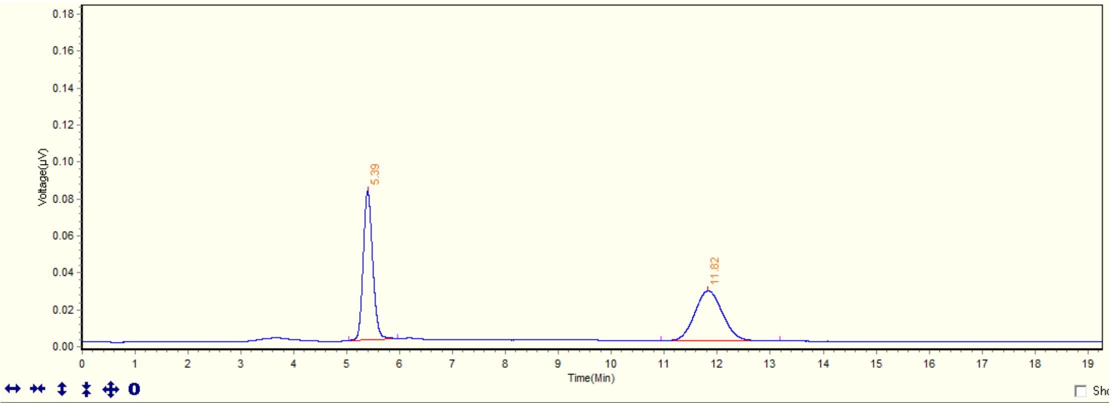

No.	Retention Time	Peak Area	Peak Height	Peak Area(%)	Peak Width	Peak Type
1	5.39	496531	40167	50.23%	0.922	BB
2	11.82	491944	13526	49.77%	2.26	BB
Total		988,475	53,693	100.00%		

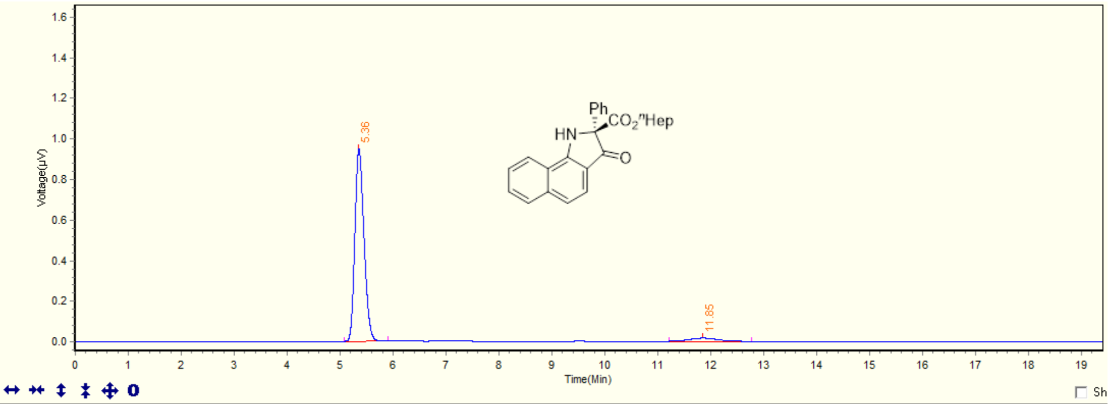

Integration Result | Calculation Result | TimeTable

No.	Retention Time	Peak Area	Peak Height	Peak Area(%)	Peak Width	Peak Type
1	5.36	5645468	472412	95.30%	0.817	BB
2	11.85	278399	7803	4.70%	1.558	BB
Total		5,923,867	480,215	100.00%		

HPLC spectra of **4af**

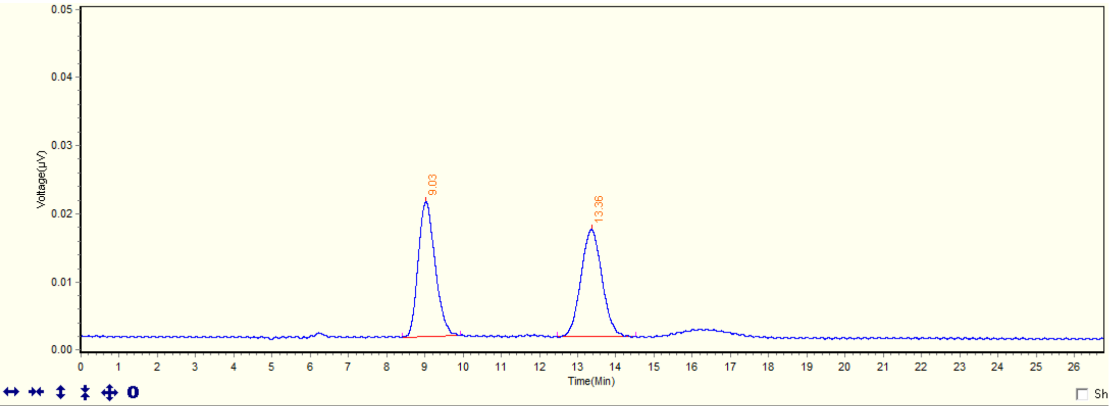

Integration Result | Calculation Result | TimeTable

No.	Retention Time	Peak Area	Peak Height	Peak Area(%)	Peak Width	Peak Type
1	9.03	302739	9851	50.17%	1.531	BB
2	13.36	300658	7871	49.83%	2.068	BB
Total		603,397	17,722	100.00%		

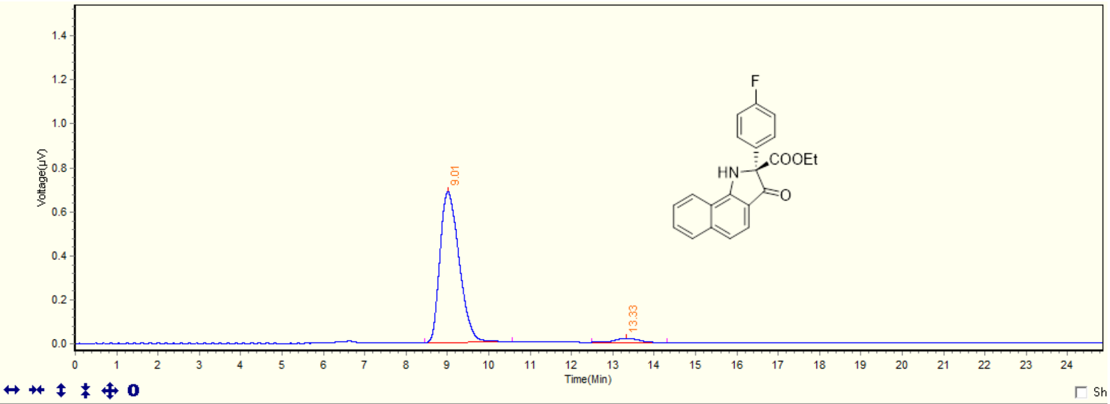

Integration Result | Calculation Result | TimeTable

No.	Retention Time	Peak Area	Peak Height	Peak Area(%)	Peak Width	Peak Type
1	9.01	11085964	342929	96.76%	2.137	BB
2	13.33	370911	9677	3.24%	1.83	BB
Total		11,456,875	352,606	100.00%		

HPLC spectra of **4ag**

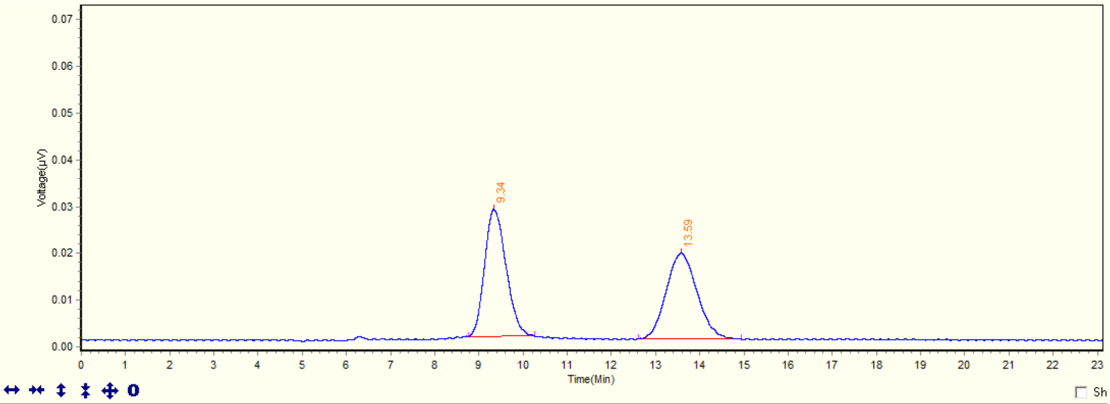

Integration Result | Calculation Result | TimeTable

No.	Retention Time	Peak Area	Peak Height	Peak Area(%)	Peak Width	Peak Type
1	9.34	457134	13557	50.73%	1.489	BB
2	13.59	444041	9164	49.27%	2.338	BB
Total		901,175	22,721	100.00%		

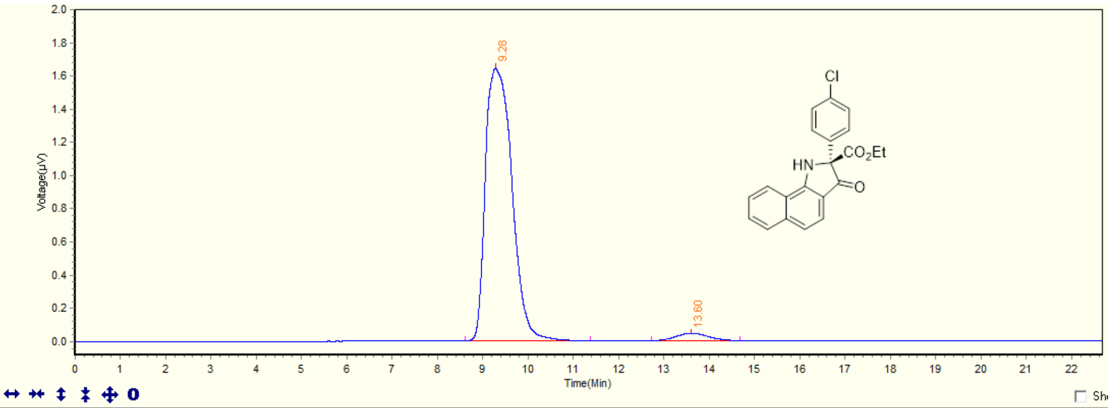

Integration Result | Calculation Result | TimeTable

No.	Retention Time	Peak Area	Peak Height	Peak Area(%)	Peak Width	Peak Type
1	9.28	33320552	821051	96.81%	2.753	BB
2	13.60	1097215	22303	3.19%	1.971	BB
Total		34,417,767	843,354	100.00%		

HPLC spectra of **4ah**

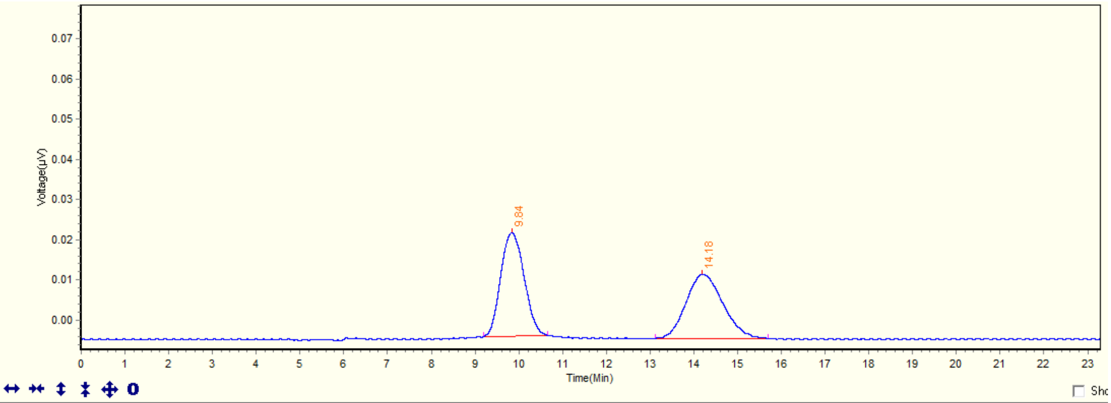

Integration Result | Calculation Result | TimeTable

No.	Retention Time	Peak Area	Peak Height	Peak Area(%)	Peak Width	Peak Type
1	9.84	477597	12873	50.44%	1.462	BB
2	14.18	469217	7960	49.56%	2.58	BB
Total		946,814	20,833	100.00%		

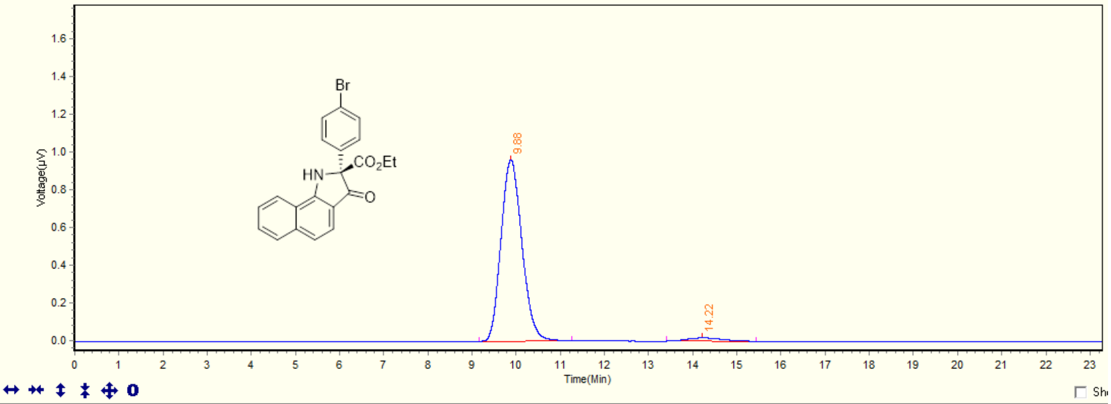

Integration Result | Calculation Result | TimeTable

No.	Retention Time	Peak Area	Peak Height	Peak Area(%)	Peak Width	Peak Type
1	9.88	15835017	480773	97.10%	2.091	BB
2	14.22	472951	8601	2.90%	2.044	BB
Total		16,307,968	489,374	100.00%		

HPLC spectra of **4ai**

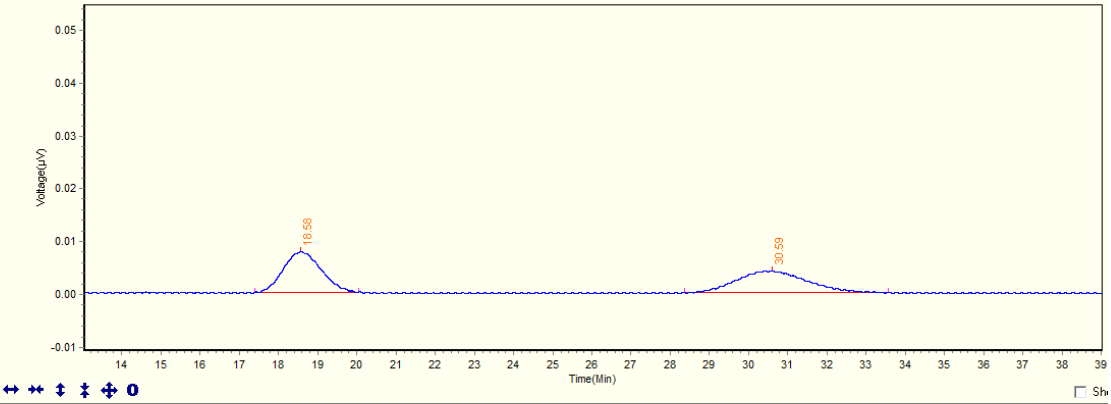

Integration Result | Calculation Result | TimeTable

No.	Retention Time	Peak Area	Peak Height	Peak Area(%)	Peak Width	Peak Type
1	18.58	256552	3879	50.54%	2.651	BB
2	30.59	251064	2064	49.46%	5.198	BB
Total		507,616	5,943	100.00%		

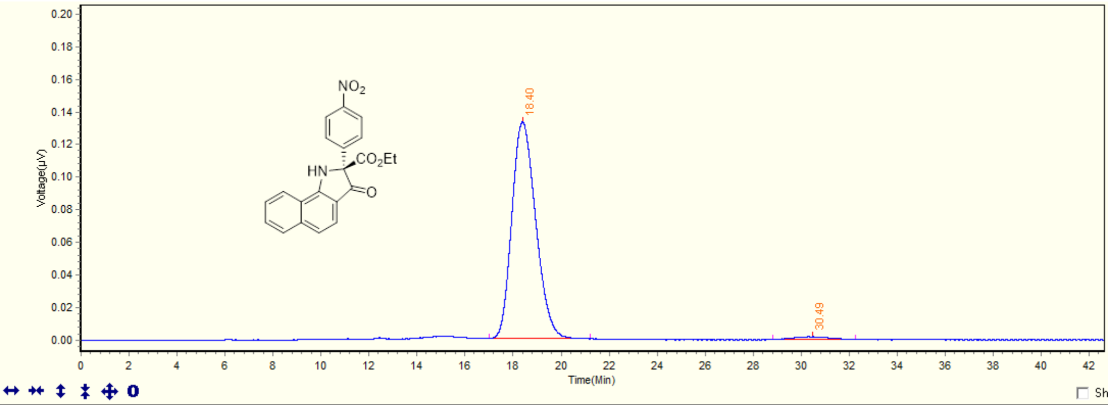

Integration Result | Calculation Result | TimeTable

No.	Retention Time	Peak Area	Peak Height	Peak Area(%)	Peak Width	Peak Type
1	18.40	4595931	66392	98.06%	4.217	BB
2	30.49	90898	876	1.94%	3.453	BB
Total		4,686,829	67,268	100.00%		

HPLC spectra of **4aj**

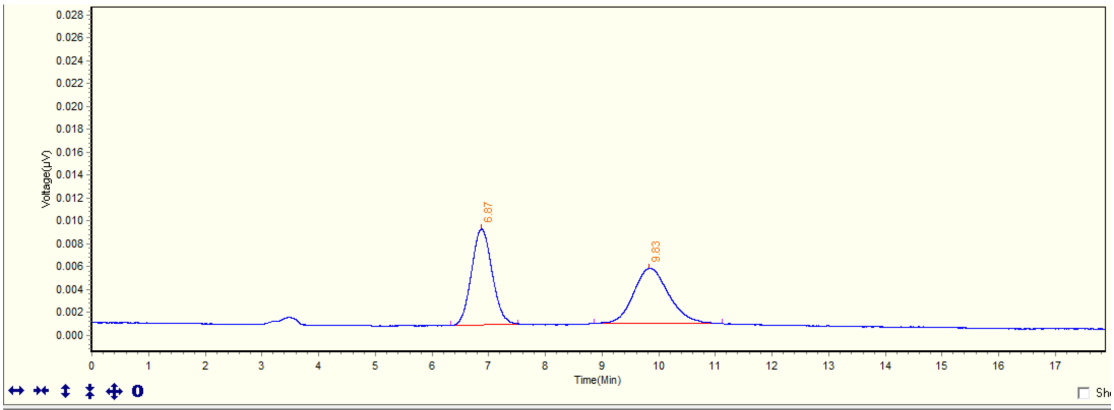

Integration Result | Calculation Result | TimeTable

No.	Retention Time	Peak Area	Peak Height	Peak Area(%)	Peak Width	Peak Type
1	6.87	105516	4193	50.36%	1.183	BB
2	9.83	104021	2421	49.64%	2.262	BB
Total		209,537	6,614	100.00%		

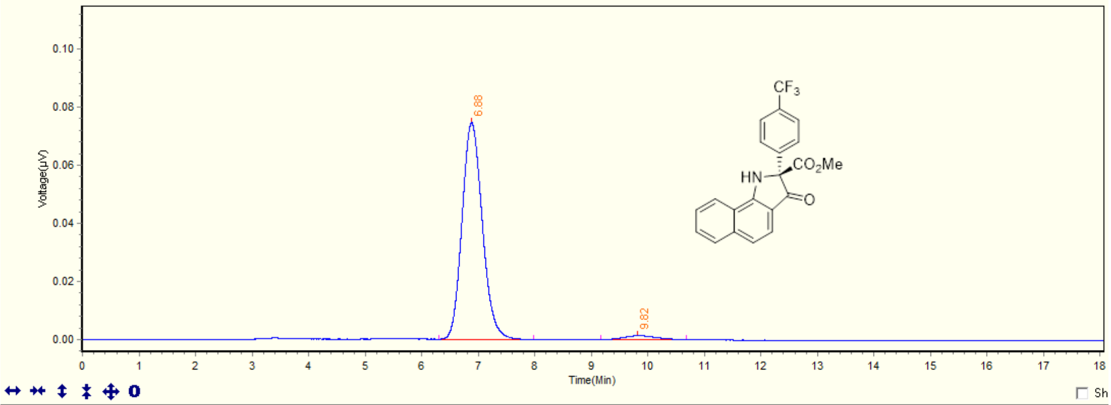

Integration Result | Calculation Result | TimeTable

No.	Retention Time	Peak Area	Peak Height	Peak Area(%)	Peak Width	Peak Type
1	6.88	929843	37355	97.31%	1.664	BB
2	9.82	25677	652	2.69%	1.513	BB
Total		955,520	38,007	100.00%		

HPLC spectra of **4ak**

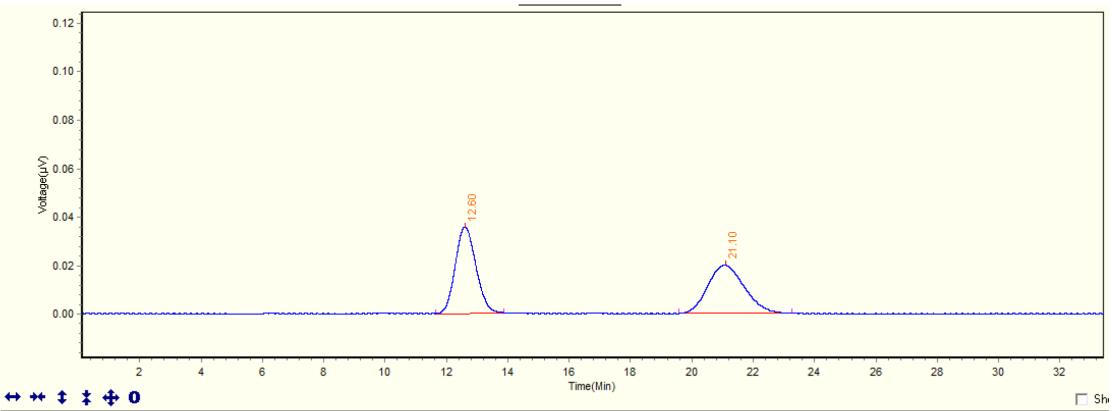

Integration Result | Calculation Result | TimeTable

No.	Retention Time	Peak Area	Peak Height	Peak Area(%)	Peak Width	Peak Type
1	12.60	830432	17779	50.22%	2.223	BB
2	21.10	823086	9903	49.78%	3.683	BB
Total		1,653,518	27,682	100.00%		

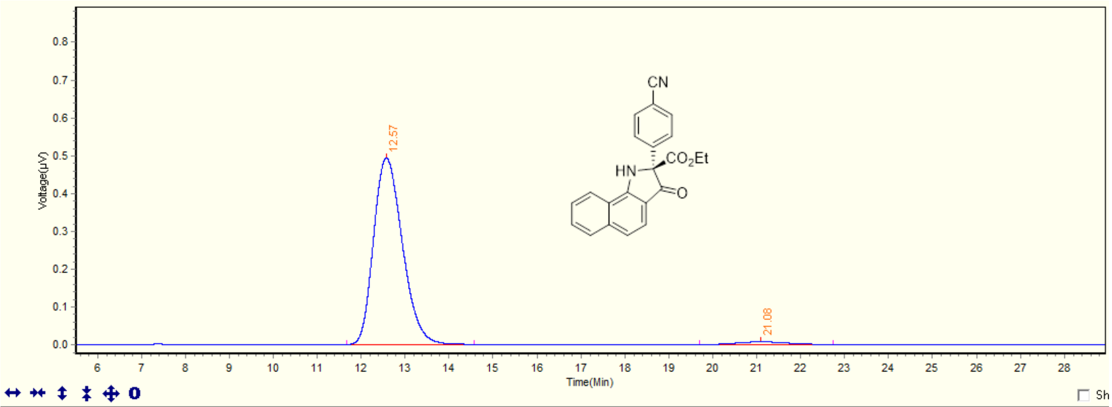

Integration Result | Calculation Result | TimeTable

No.	Retention Time	Peak Area	Peak Height	Peak Area(%)	Peak Width	Peak Type
1	12.57	11216661	246463	97.58%	2.905	BB
2	21.08	278350	3559	2.42%	3.053	BB
Total		11,495,011	250,022	100.00%		

HPLC spectra of 4**al**

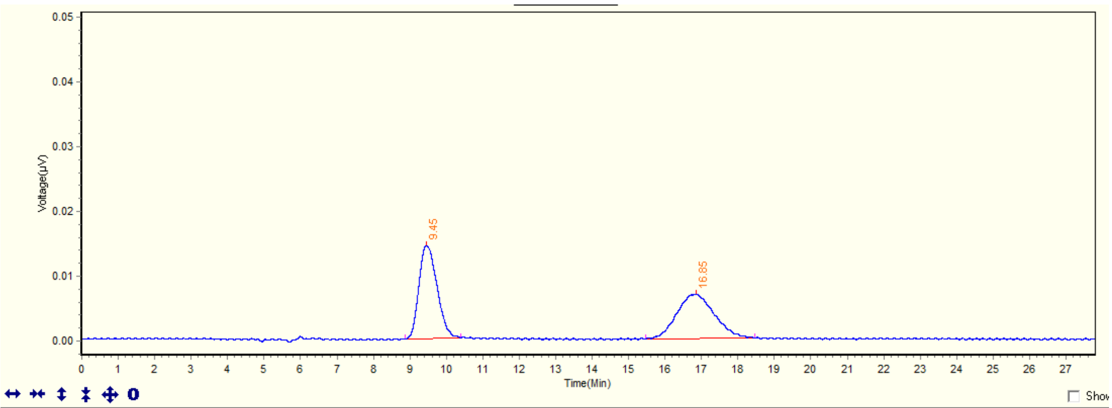

Integration Result | Calculation Result | TimeTable

No.	Retention Time	Peak Area	Peak Height	Peak Area(%)	Peak Width	Peak Type
1	9.45	248438	7185	50.00%	1.512	BB
2	16.85	248431	3461	50.00%	3.006	BB
Total		496,869	10,646	100.00%		

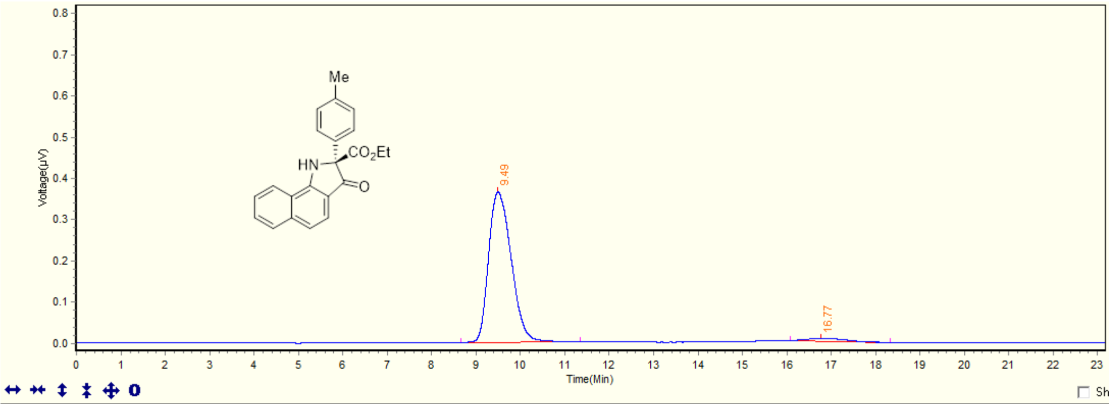

Integration Result | Calculation Result | TimeTable

No.	Retention Time	Peak Area	Peak Height	Peak Area(%)	Peak Width	Peak Type
1	9.49	6445157	182501	97.10%	2.69	BB
2	16.77	192659	3354	2.90%	2.253	BB
Total		6,637,816	185,855	100.00%		

HPLC spectra of **4am**

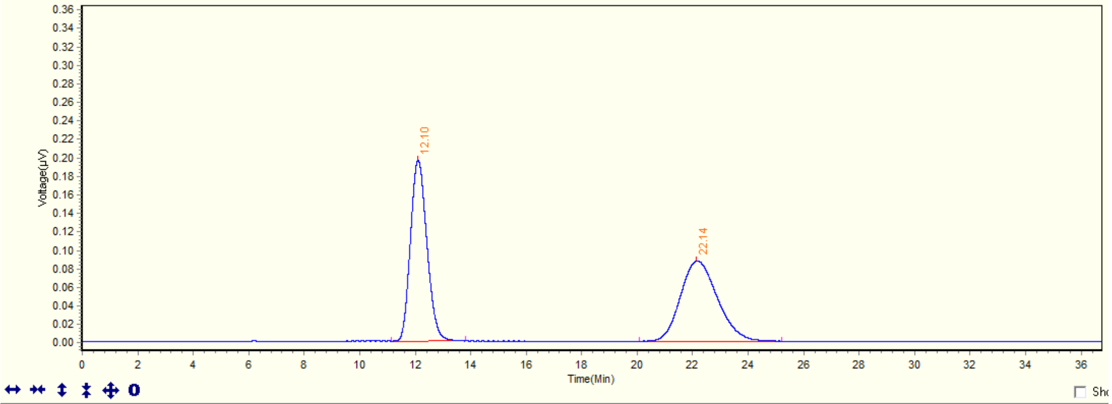

No.	Retention Time	Peak Area	Peak Height	Peak Area(%)	Peak Width	Peak Type
1	12.10	4032059	97805	50.23%	2.682	BB
2	22.14	3995905	43326	49.77%	5.1	BB
Total		8,027,964	141,131	100.00%		

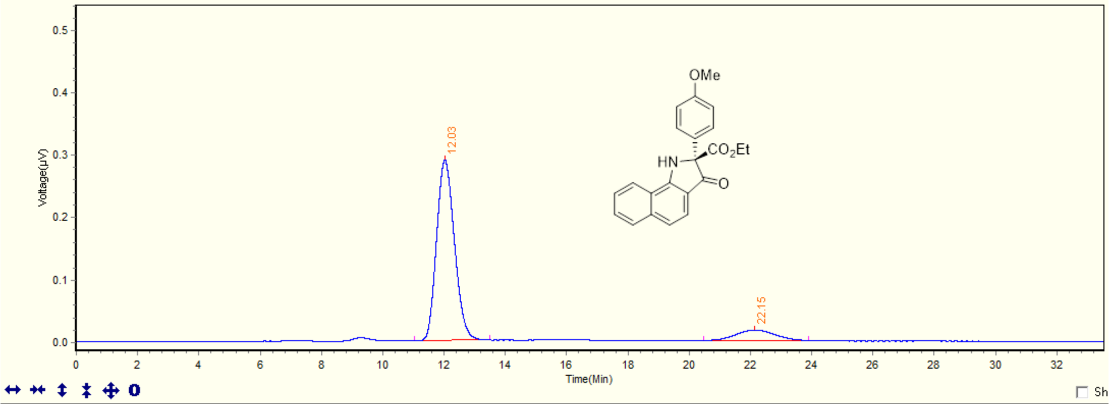

No.	Retention Time	Peak Area	Peak Height	Peak Area(%)	Peak Width	Peak Type
1	12.03	5828371	144541	88.87%	2.474	BB
2	22.15	729857	8407	11.13%	3.443	BB
Total		6,558,228	152,948	100.00%		

HPLC spectra of **4an**

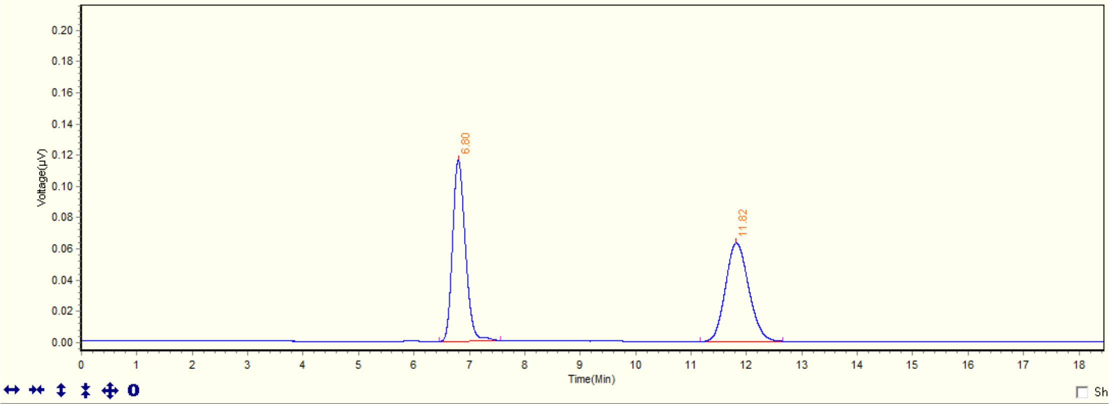

No.	Retention Time	Peak Area	Peak Height	Peak Area(%)	Peak Width	Peak Type
1	6.80	928226	58023	50.35%	1.103	BB
2	11.82	915392	31515	49.65%	1.49	BB
Total		1,843,618	89,538	100.00%		

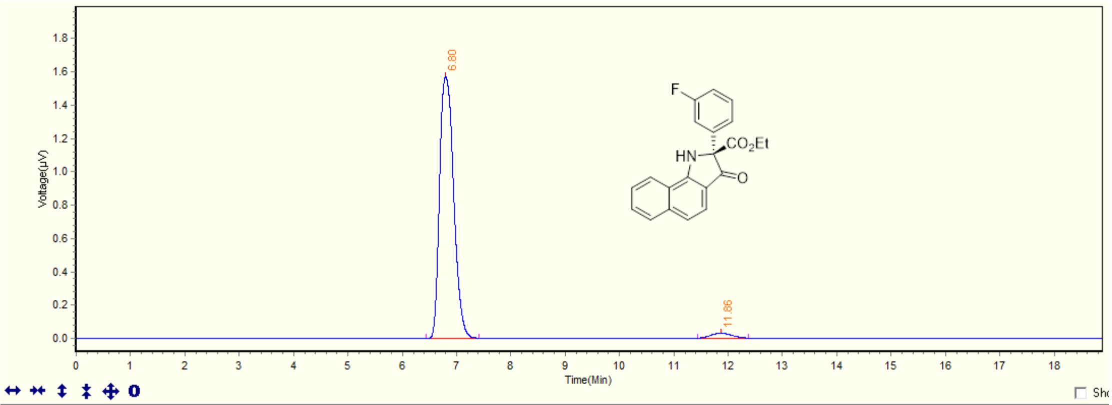

Integration Result | Calculation Result | TimeTable

No.	Retention Time	Peak Area	Peak Height	Peak Area(%)	Peak Width	Peak Type
1	6.80	14127516	782887	97.27%	0.973	BB
2	11.86	396413	14909	2.73%	0.936	BB
Total		14,523,929	797,796	100.00%		

HPLC spectra of **4ao**

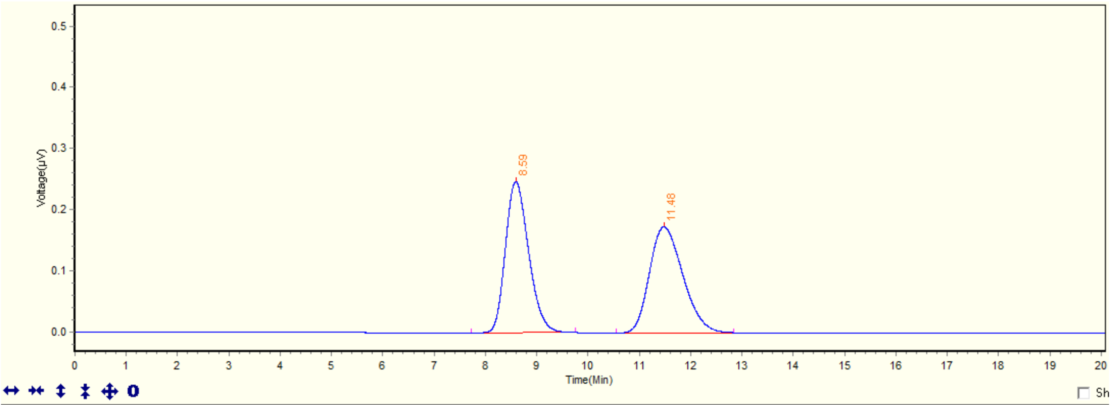

Integration Result | Calculation Result | TimeTable

No.	Retention Time	Peak Area	Peak Height	Peak Area(%)	Peak Width	Peak Type
1	8.59	3877884	123431	49.79%	2.03	BB
2	11.48	3910798	86704	50.21%	2.288	BB
Total		7,788,682	210,135	100.00%		

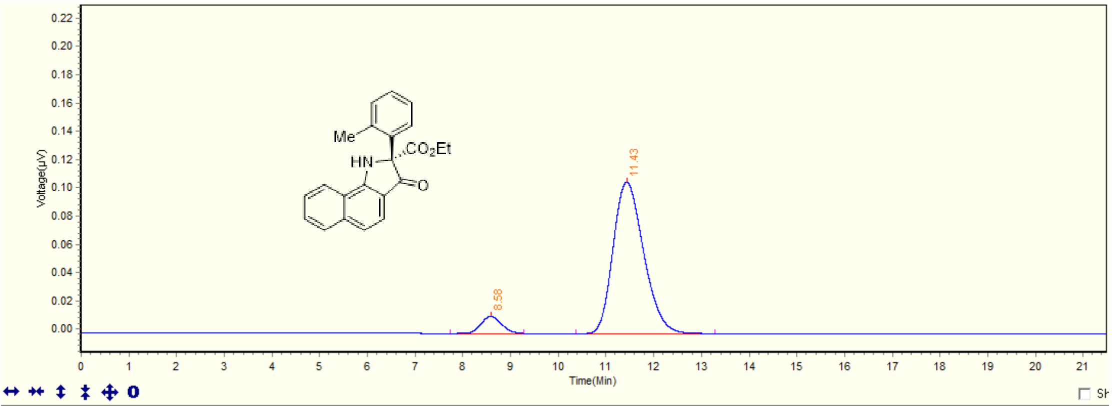

Integration Result | Calculation Result | TimeTable

No.	Retention Time	Peak Area	Peak Height	Peak Area(%)	Peak Width	Peak Type
1	8.58	180204	6058	7.03%	1.543	BB
2	11.43	2382698	53629	92.97%	2.913	BB
Total		2,562,902	59,687	100.00%		

HPLC spectra of **4ap**

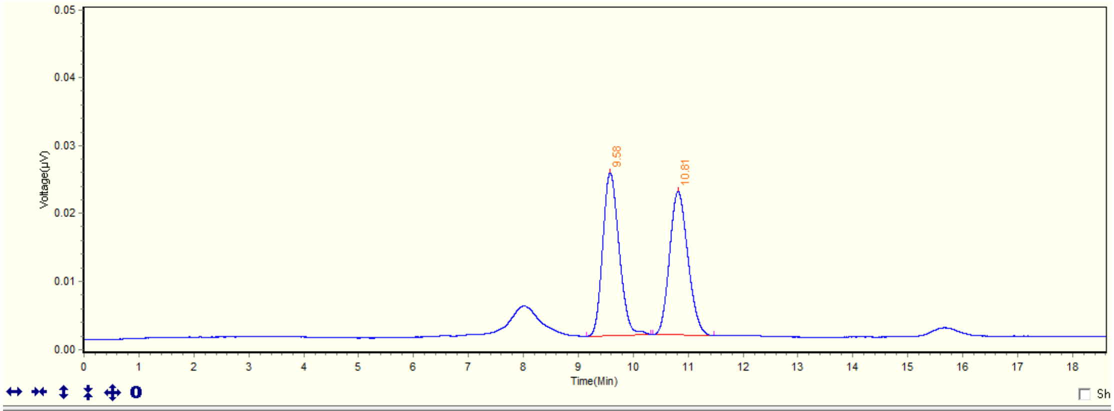

No.	Retention Time	Peak Area	Peak Height	Peak Area(%)	Peak Width	Peak Type
1	9.58	249015	11989	50.39%	1.172	BB
2	10.81	245193	10561	49.61%	1.13	BB
Total		494,208	22,550	100.00%		

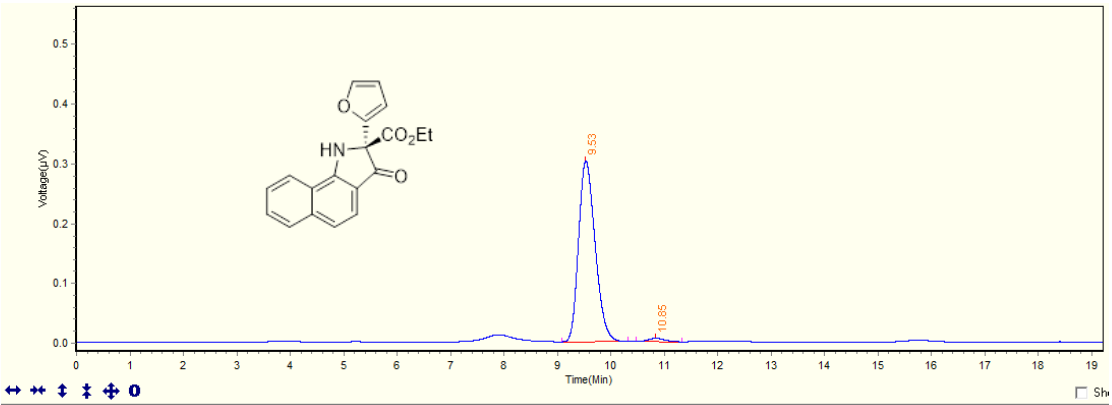

No.	Retention Time	Peak Area	Peak Height	Peak Area(%)	Peak Width	Peak Type
1	9.53	3245238	151056	98.15%	1.223	BB
2	10.85	61197	2802	1.85%	0.843	BB
Total		3,306,435	153,858	100.00%		

HPLC spectra of **4aq**

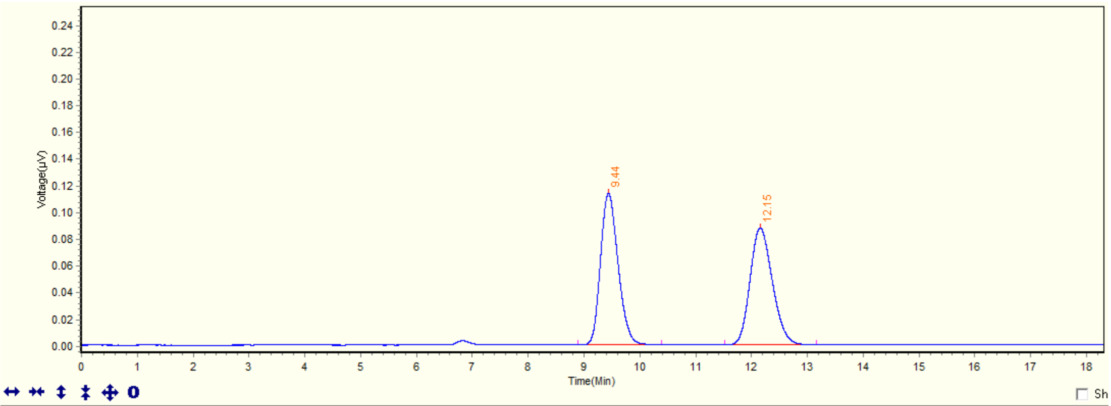

No.	Retention Time	Peak Area	Peak Height	Peak Area(%)	Peak Width	Peak Type
1	9.44	1230147	56571	50.39%	1.489	BB
2	12.15	1211176	43704	49.61%	1.643	BB
Total		2,441,323	100,275	100.00%		

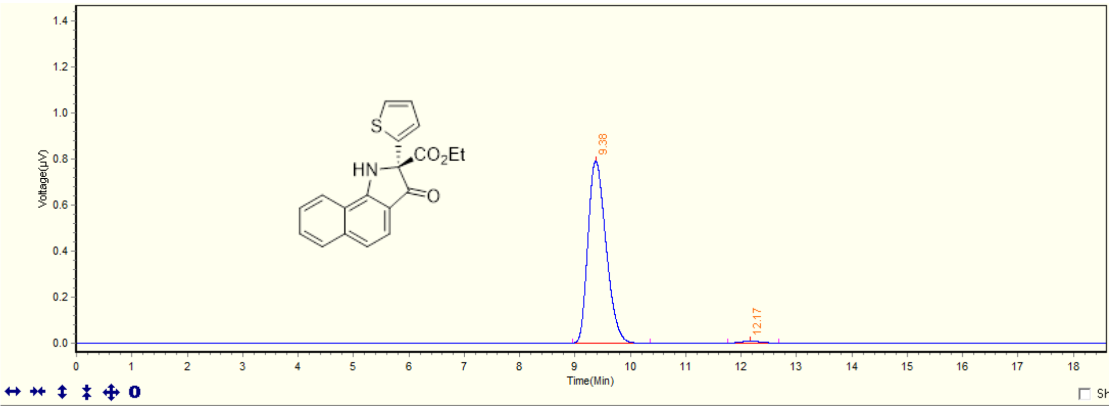

Integration Result | Calculation Result | TimeTable

No.	Retention Time	Peak Area	Peak Height	Peak Area(%)	Peak Width	Peak Type
1	9.38	8945504	395846	98.73%	1.394	BB
2	12.17	115230	4510	1.27%	0.923	BB
Total		9,060,734	400,356	100.00%		

HPLC spectra of **4ar**

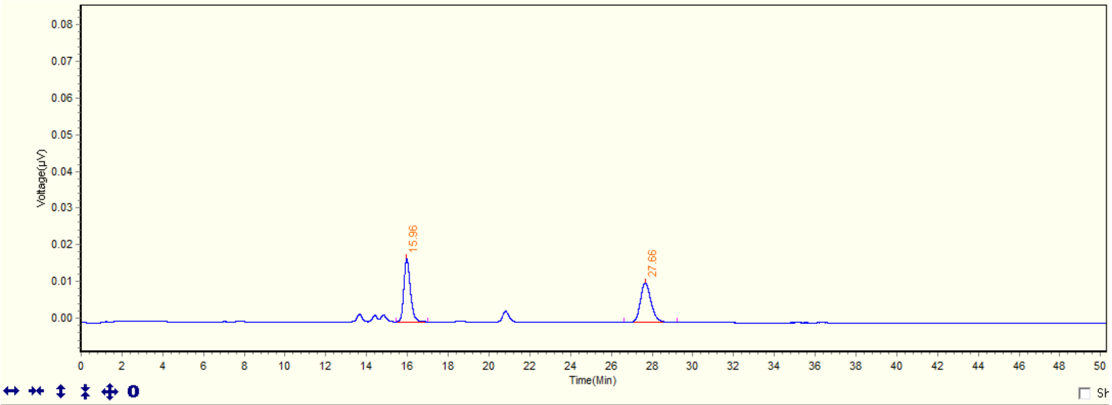

Integration Result | Calculation Result | TimeTable

No.	Retention Time	Peak Area	Peak Height	Peak Area(%)	Peak Width	Peak Type
1	15.96	189629	8580	49.24%	1.582	BB
2	27.66	195477	5365	50.76%	2.628	BB
Total		385,106	13,945	100.00%		

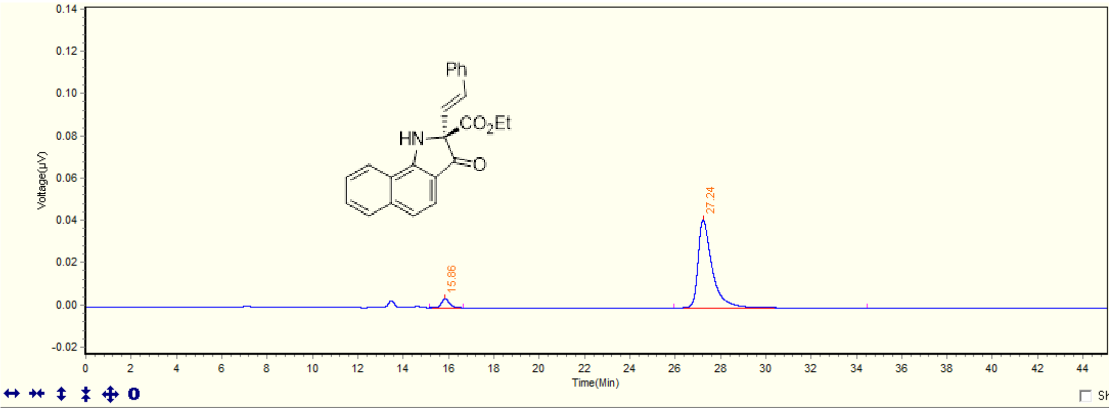

Integration Result | Calculation Result | TimeTable

No.	Retention Time	Peak Area	Peak Height	Peak Area(%)	Peak Width	Peak Type
1	15.86	50429	2049	5.25%	1.478	BB
2	27.24	910600	20770	94.75%	8.493	BB
Total		961,029	22,819	100.00%		

HPLC spectra of **4bq**

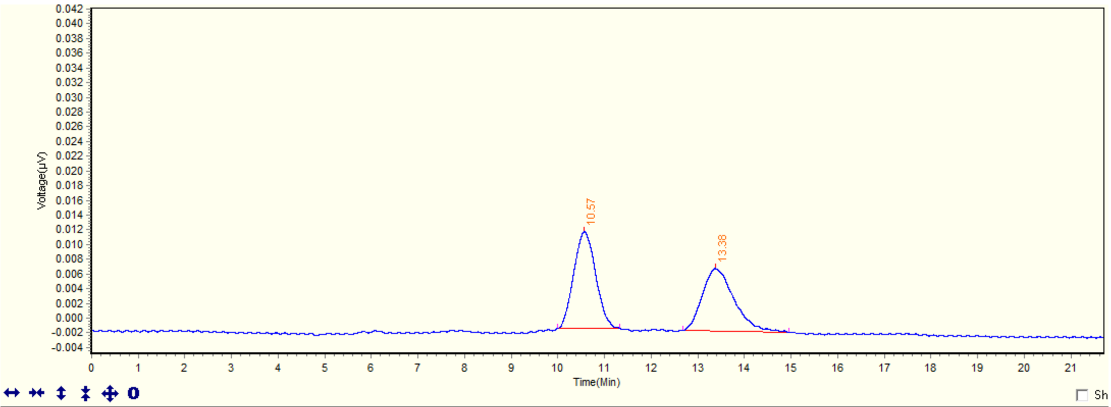

Integration Result | Calculation Result | TimeTable

No.	Retention Time	Peak Area	Peak Height	Peak Area(%)	Peak Width	Peak Type
1	10.57	214475	6583	51.28%	1.336	BB
2	13.38	203807	4264	48.72%	2.277	BB
Total		418,282	10,847	100.00%		

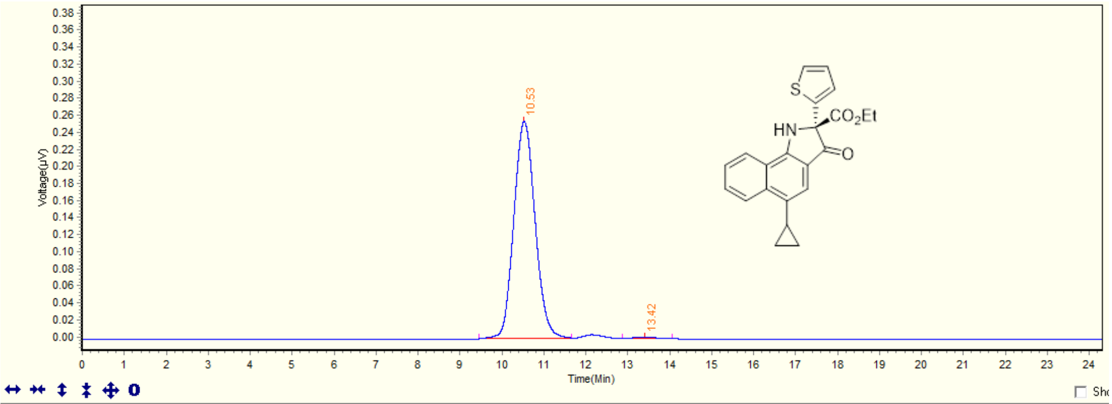

Integration Result | Calculation Result | TimeTable

No.	Retention Time	Peak Area	Peak Height	Peak Area(%)	Peak Width	Peak Type
1	10.53	4472519	127041	99.30%	2.204	BB
2	13.42	31628	861	0.70%	1.178	BB
Total		4,504,147	127,902	100.00%		

HPLC spectra of **4cq**

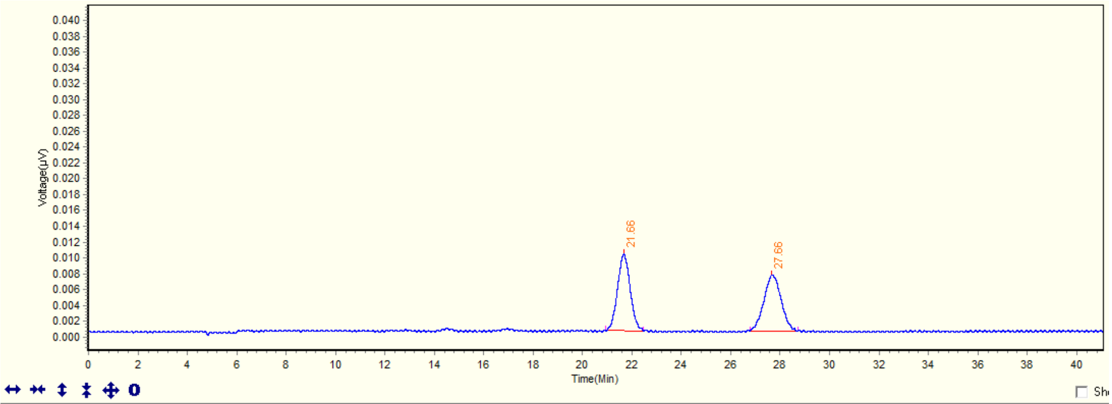

Integration Result | Calculation Result | TimeTable

No.	Retention Time	Peak Area	Peak Height	Peak Area(%)	Peak Width	Peak Type
1	21.66	173008	4843	50.34%	1.533	BB
2	27.66	170672	3541	49.66%	1.924	BB
Total		343,680	8,384	100.00%		

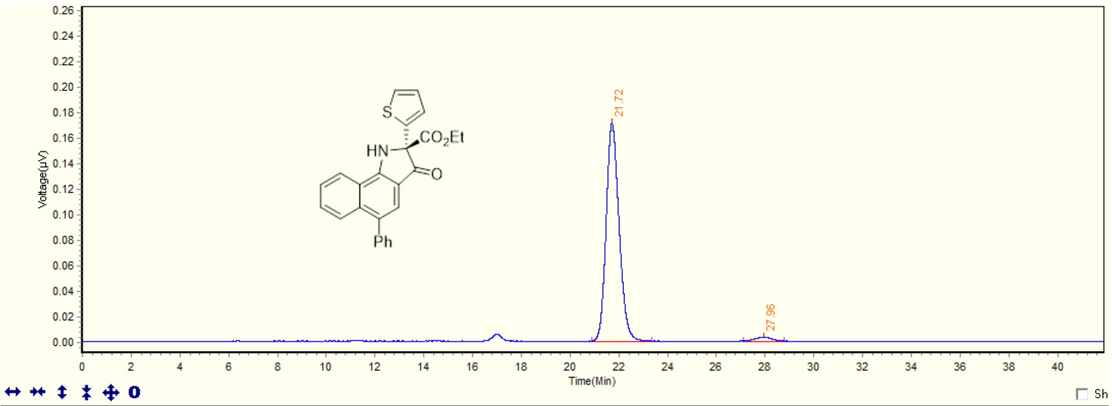

Integration Result | Calculation Result | TimeTable

No.	Retention Time	Peak Area	Peak Height	Peak Area(%)	Peak Width	Peak Type
1	21.72	3150190	85404	97.77%	2.471	BB
2	27.96	72010	1561	2.23%	1.669	BB
Total		3,222,200	86,965	100.00%		

HPLC spectra of **4dq**

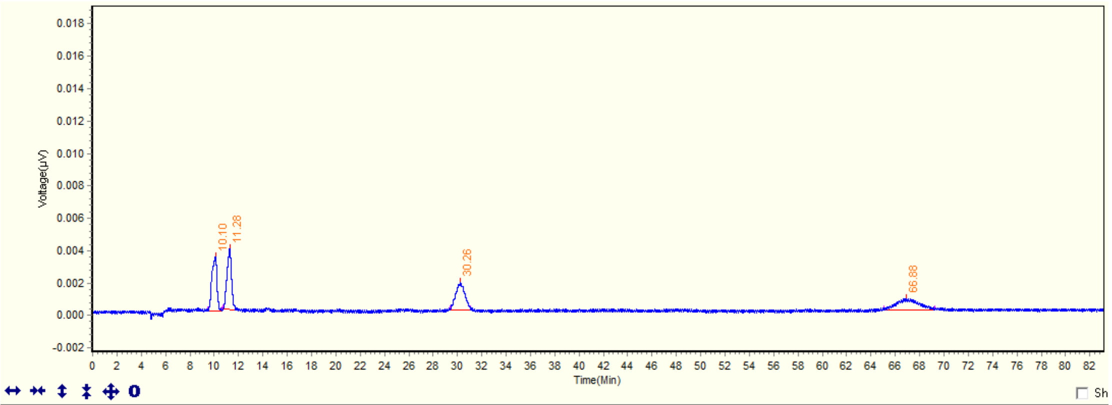

Integration Result | Calculation Result | TimeTable

No.	Retention Time	Peak Area	Peak Height	Peak Area(%)	Peak Width	Peak Type
1	10.10	48543	1667	26.27%	1.107	BB
2	11.28	49068	1884	26.56%	1.083	BB
3	30.26	43164	830	23.36%	1.697	BB
4	66.88	44003	350	23.81%	4.139	BB
Total		184,778	4,731	100.00%		

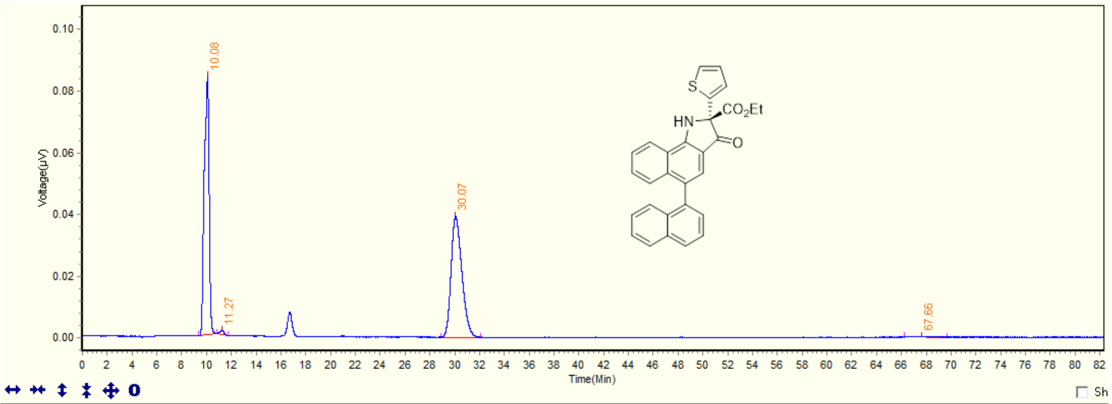

Integration Result | Calculation Result | TimeTable

No.	Retention Time	Peak Area	Peak Height	Peak Area(%)	Peak Width	Peak Type
1	10.08	1133714	41890	48.24%	1.433	BB
2	11.27	15662	700	0.67%	0.91	BB
3	30.07	1186578	19595	50.49%	3.187	BB
4	67.66	14297	154	0.61%	3.448	BB
Total		2,350,251	62,339	100.00%		

HPLC spectra of **4eq**

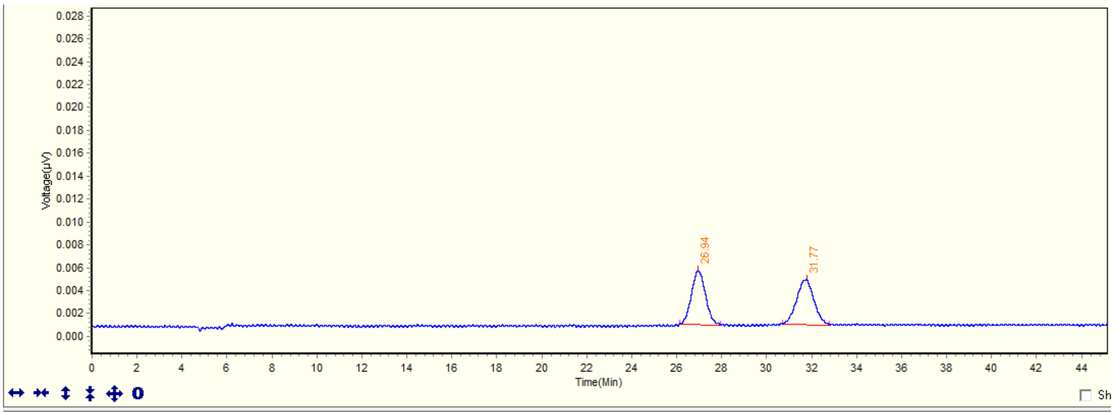

No.	Retention Time	Peak Area	Peak Height	Peak Area(%)	Peak Width	Peak Type
1	26.94	103613	2366	49.81%	1.839	BB
2	31.77	104390	1955	50.19%	2.092	BB
Total		208,003	4,321	100.00%		

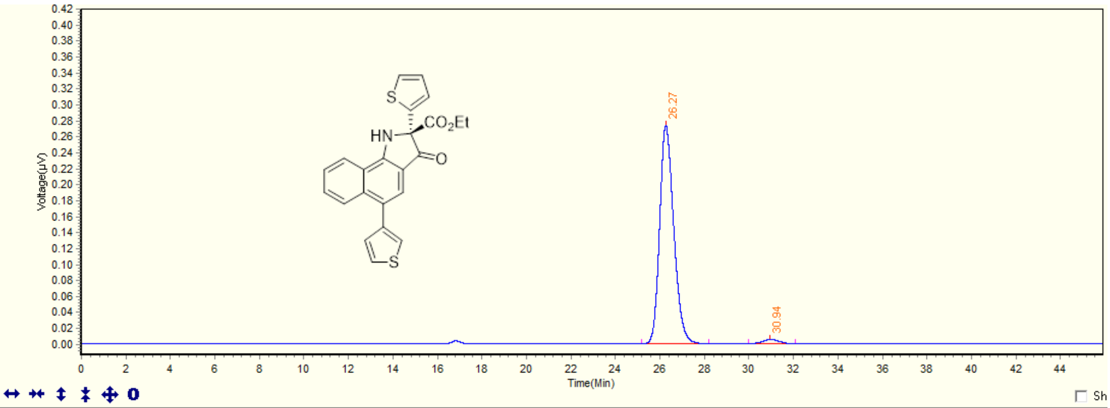

No.	Retention Time	Peak Area	Peak Height	Peak Area(%)	Peak Width	Peak Type
1	26.27	6016864	136668	97.77%	3.028	BB
2	30.94	137195	2674	2.23%	2.096	BB
Total		6,154,059	139,342	100.00%		

HPLC spectra of **4fq**

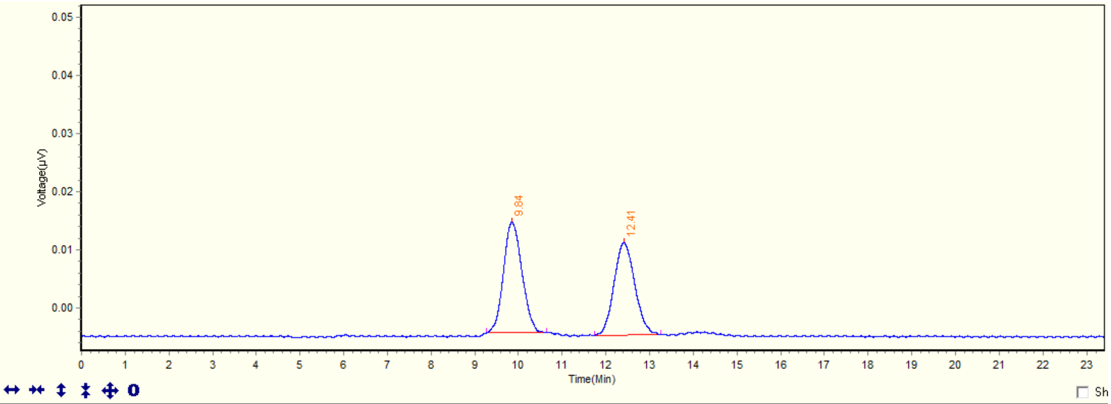

No.	Retention Time	Peak Area	Peak Height	Peak Area(%)	Peak Width	Peak Type
1	9.84	279991	9521	51.35%	1.372	BB
2	12.41	265240	7982	48.65%	1.518	BB
Total		545,231	17,503	100.00%		

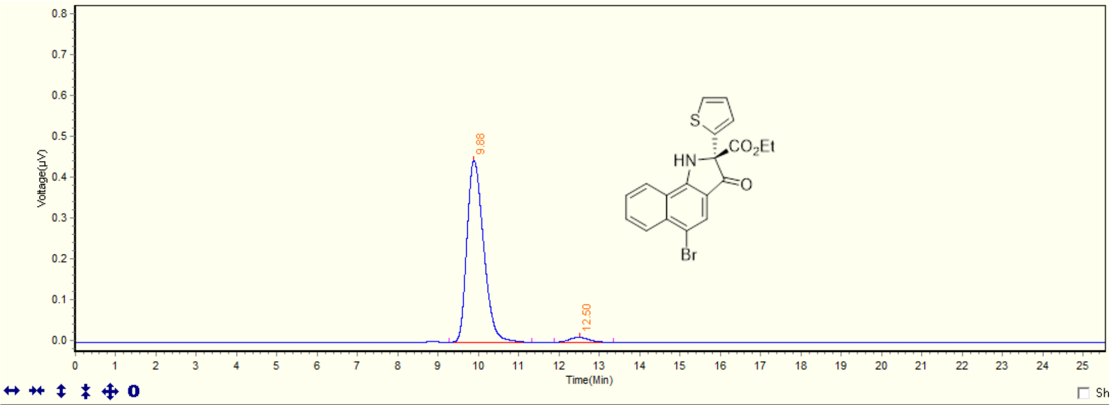

Integration Result | Calculation Result | TimeTable

No.	Retention Time	Peak Area	Peak Height	Peak Area(%)	Peak Width	Peak Type
1	9.88	6403288	222511	96.96%	2.067	BB
2	12.50	200862	6082	3.04%	1.476	BB
Total		6,604,150	228,593	100.00%		

HPLC spectra of **4gq**

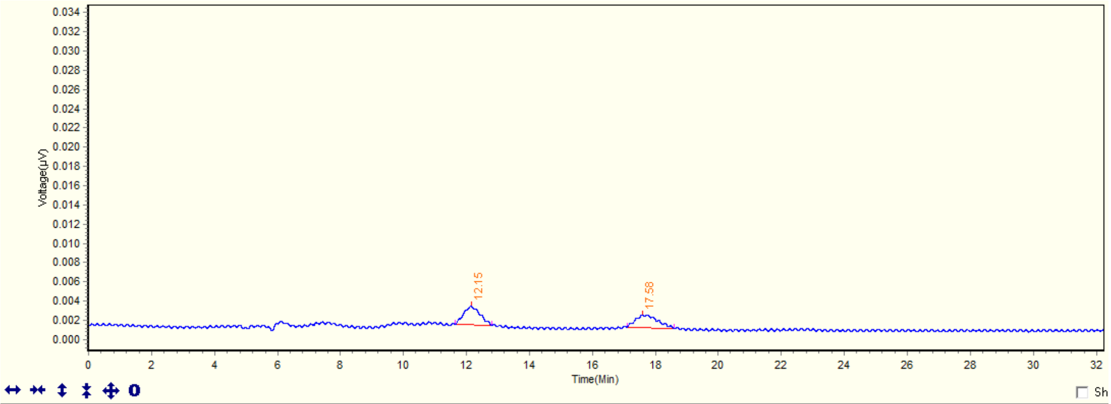

Integration Result | Calculation Result | TimeTable

No.	Retention Time	Peak Area	Peak Height	Peak Area(%)	Peak Width	Peak Type
1	12.15	34138	968	50.96%	1.163	BB
2	17.58	32856	673	49.04%	1.509	BB
Total		66,994	1,641	100.00%		

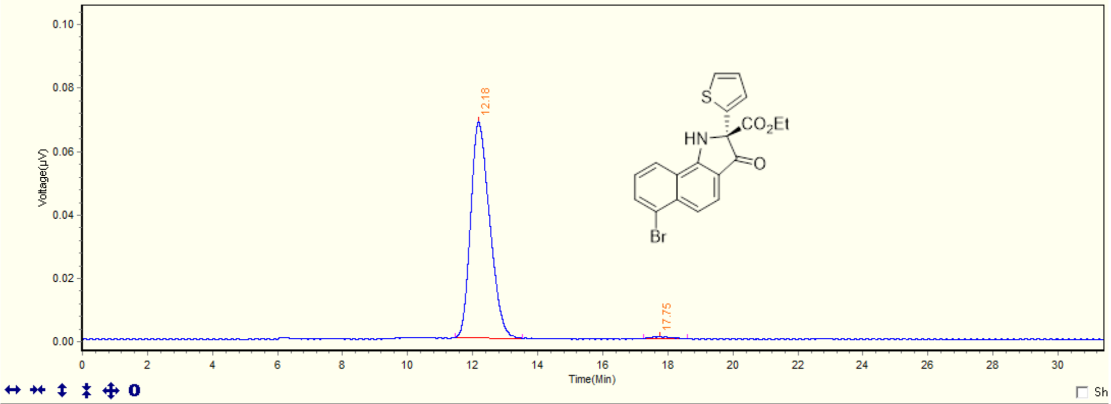

Integration Result | Calculation Result | TimeTable

No.	Retention Time	Peak Area	Peak Height	Peak Area(%)	Peak Width	Peak Type
1	12.18	1386970	34097	98.93%	2.091	BB
2	17.75	14969	358	1.07%	1.338	BB
Total		1,401,939	34,455	100.00%		

HPLC spectra of **4hq**

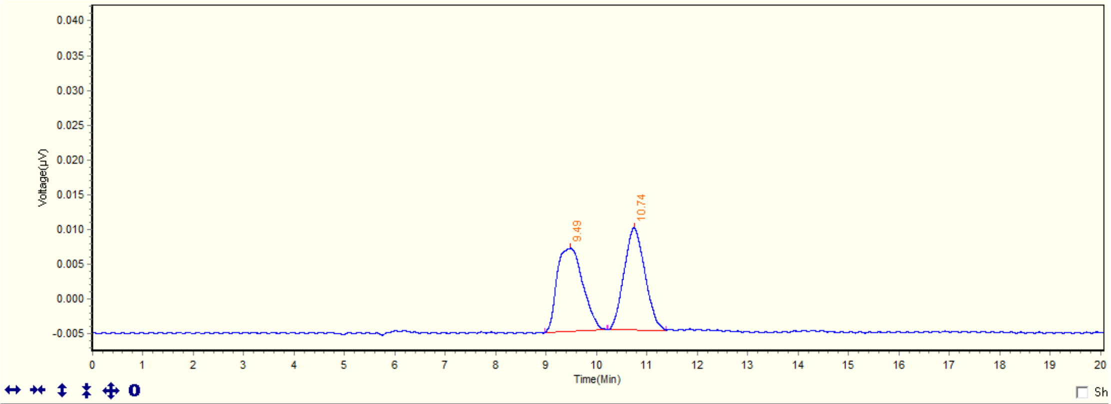

No.	Retention Time	Peak Area	Peak Height	Peak Area(%)	Peak Width	Peak Type
1	9.49	214566	5984	50.34%	1.242	BB
2	10.74	211626	7342	49.66%	1.143	BB
Total		426,192	13,326	100.00%		

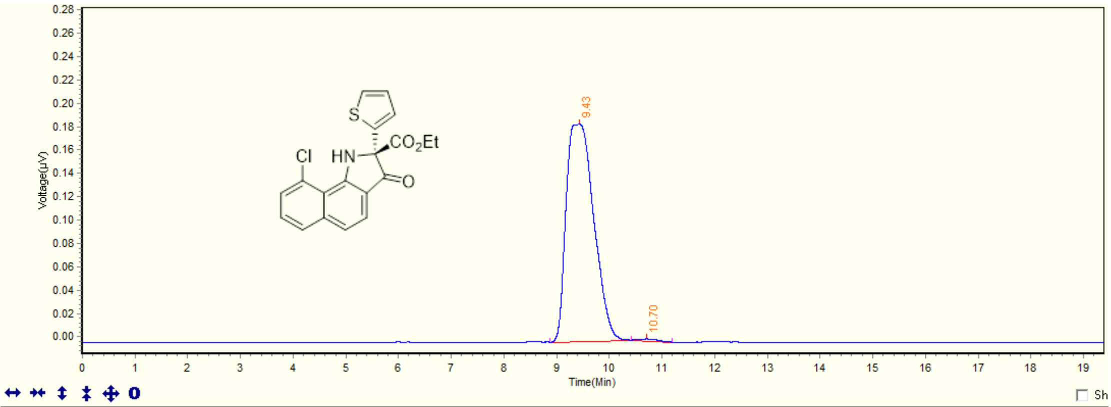

No.	Retention Time	Peak Area	Peak Height	Peak Area(%)	Peak Width	Peak Type
1	9.43	3390165	92917	99.44%	1.53	BB
2	10.70	19189	814	0.56%	0.765	BB
Total		3,409,354	93,731	100.00%		

HPLC spectra of **4iq**

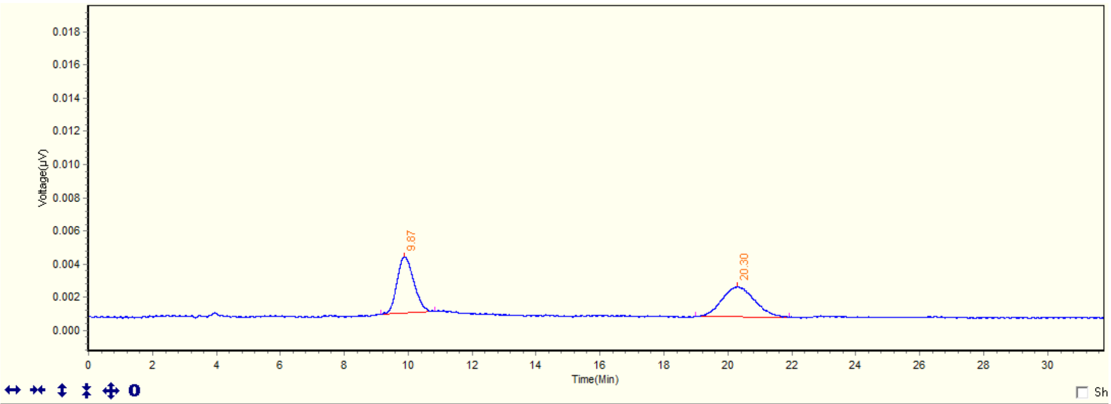

No.	Retention Time	Peak Area	Peak Height	Peak Area(%)	Peak Width	Peak Type
1	9.87	61906	1700	49.67%	1.693	BB
2	20.30	62718	904	50.33%	2.945	BB
Total		124,624	2,604	100.00%		

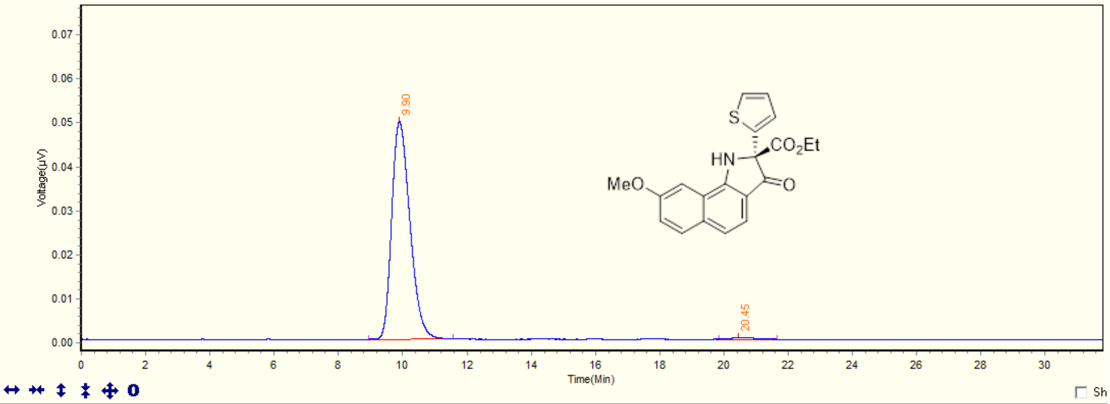

Integration Result | Calculation Result | TimeTable

No.	Retention Time	Peak Area	Peak Height	Peak Area(%)	Peak Width	Peak Type
1	9.90	973315	24692	98.98%	2.63	BB
2	20.45	10015	166	1.02%	1.814	BB
Total		983,330	24,858	100.00%		

HPLC spectra of **4jq**

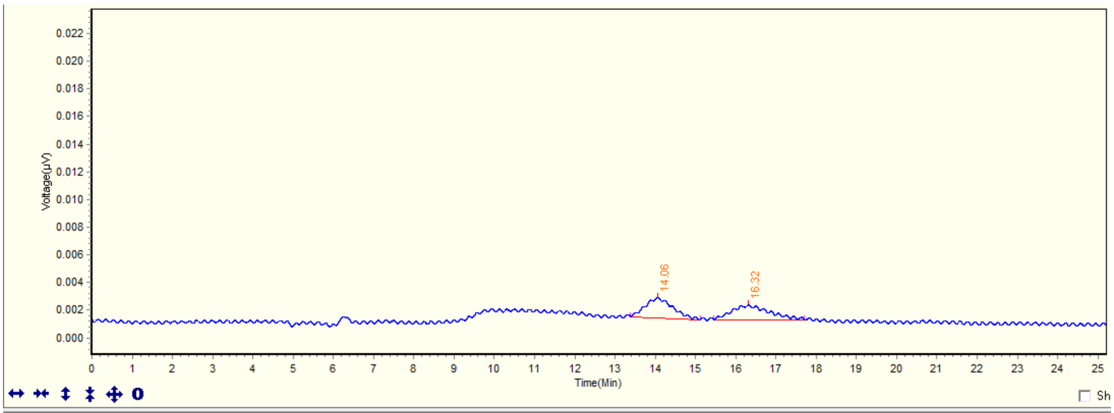

Integration Result | Calculation Result | TimeTable

No.	Retention Time	Peak Area	Peak Height	Peak Area(%)	Peak Width	Peak Type
1	14.06	34273	742	49.42%	1.73	BB
2	16.32	35073	556	50.58%	2.252	BB
Total		69,346	1,298	100.00%		

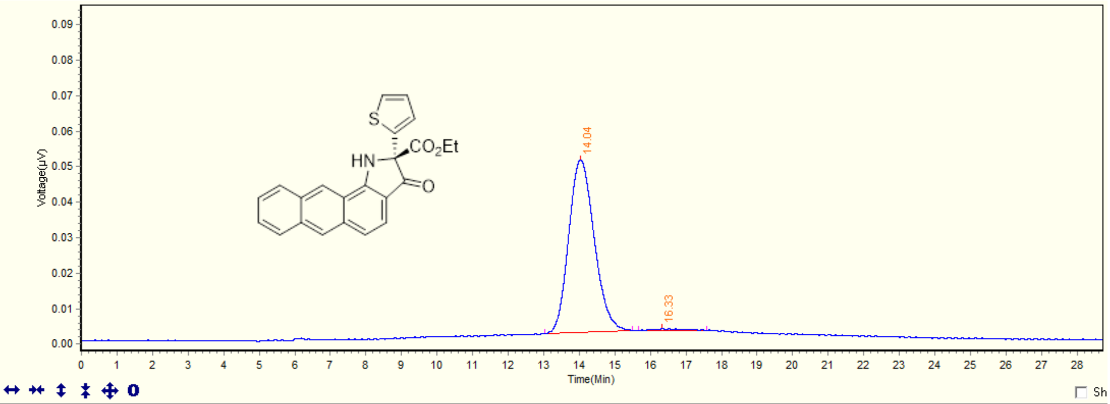

Integration Result | Calculation Result | TimeTable

No.	Retention Time	Peak Area	Peak Height	Peak Area(%)	Peak Width	Peak Type
1	14.04	1217279	24321	98.70%	2.477	BB
2	16.33	16035	259	1.30%	1.899	BB
Total		1,233,314	24,580	100.00%		

HPLC spectra of **4kq**

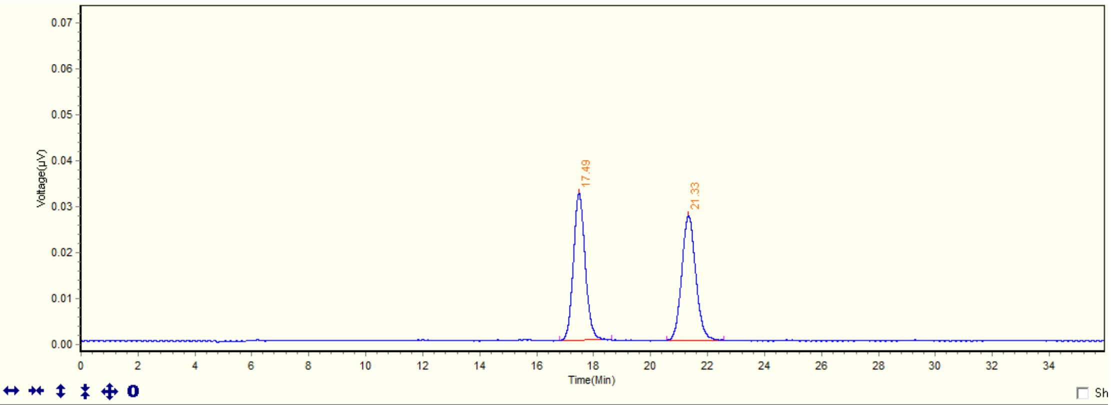

Integration Result | Calculation Result | TimeTable

No.	Retention Time	Peak Area	Peak Height	Peak Area(%)	Peak Width	Peak Type
1	17.49	462939	16038	49.80%	1.826	BB
2	21.33	466690	13575	50.20%	2.014	BB
Total		929,629	29,613	100.00%		

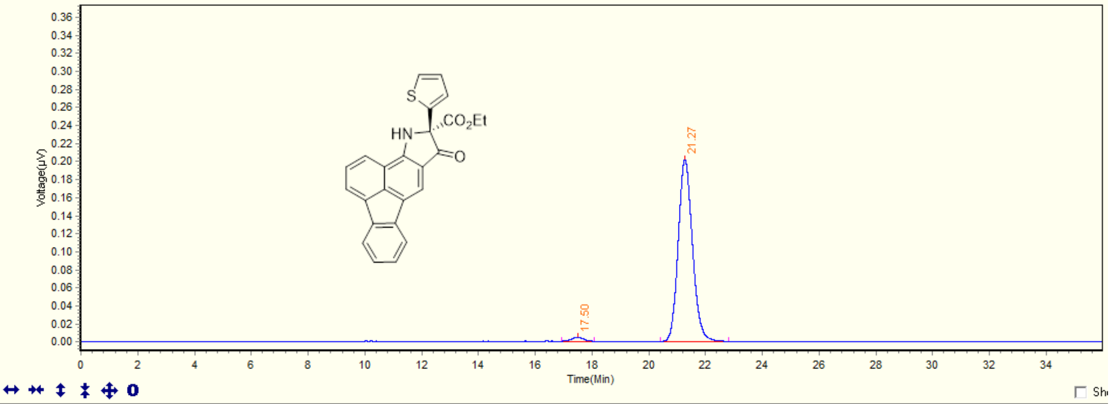

Integration Result | Calculation Result | TimeTable

No.	Retention Time	Peak Area	Peak Height	Peak Area(%)	Peak Width	Peak Type
1	17.50	63934	2229	1.79%	1.136	BB
2	21.27	3505676	100564	98.21%	2.424	BB
Total		3,569,610	102,793	100.00%		

HPLC spectra of **4lq**

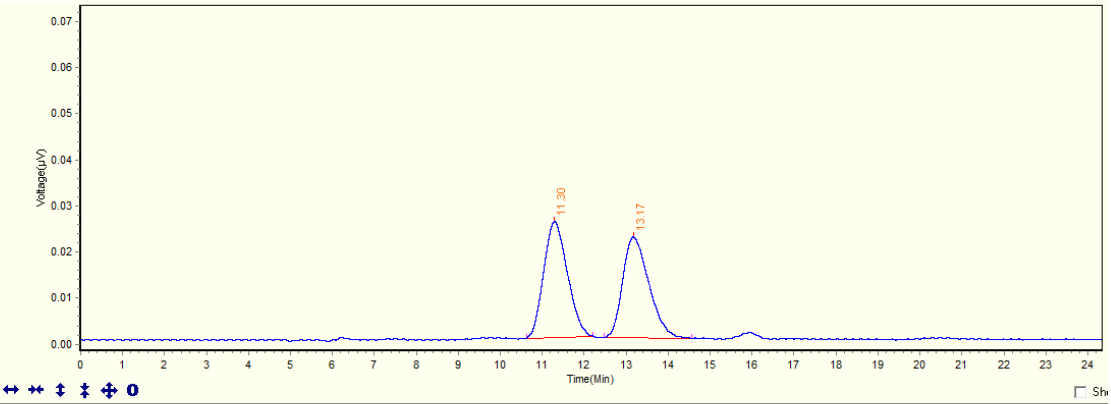

Integration Result | Calculation Result | TimeTable

No.	Retention Time	Peak Area	Peak Height	Peak Area(%)	Peak Width	Peak Type
1	11.30	492653	12556	50.78%	1.598	BB
2	13.17	477536	10974	49.22%	2.081	BB
Total		970,189	23,530	100.00%		

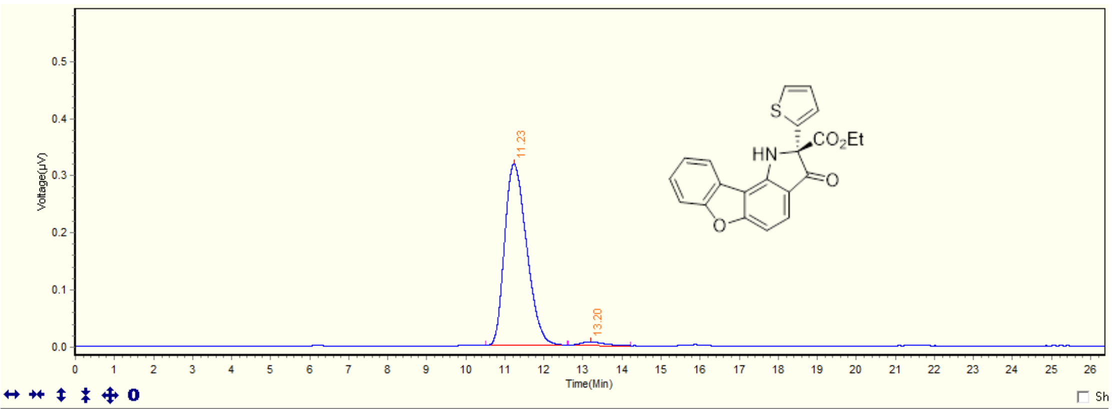

Integration Result | Calculation Result | TimeTable

No.	Retention Time	Peak Area	Peak Height	Peak Area(%)	Peak Width	Peak Type
1	11.23	6253717	159230	98.10%	2.075	BB
2	13.20	120891	2996	1.90%	1.605	BB
Total		6,374,608	162,226	100.00%		

HPLC spectra of **4mq**

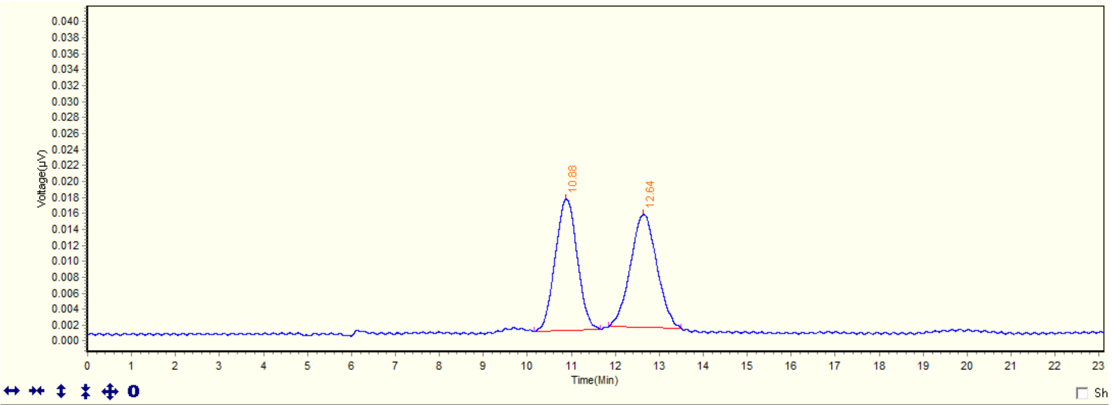

Integration Result | Calculation Result | TimeTable

No.	Retention Time	Peak Area	Peak Height	Peak Area(%)	Peak Width	Peak Type
1	10.88	287001	8258	49.31%	1.496	BB
2	12.64	295075	7061	50.69%	1.644	BB
Total		582,076	15,319	100.00%		

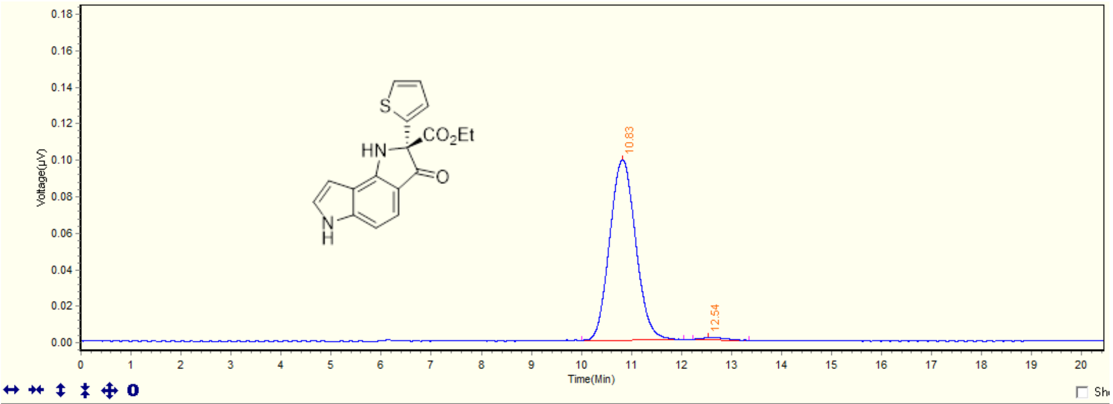

Integration Result | Calculation Result | TimeTable

No.	Retention Time	Peak Area	Peak Height	Peak Area(%)	Peak Width	Peak Type
1	10.83	1780520	49354	98.72%	2.031	BB
2	12.54	22996	609	1.28%	1.137	BB
Total		1,803,516	49,963	100.00%		

HPLC spectra of **4nq**

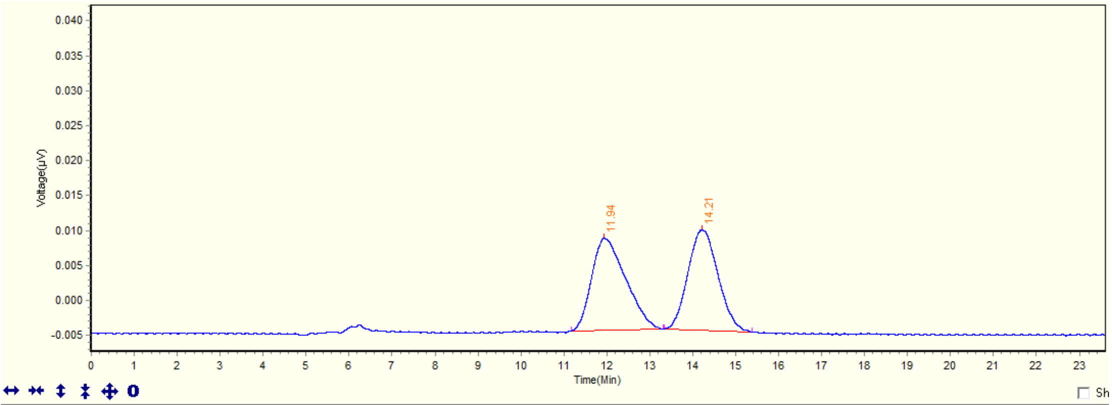

No.	Retention Time	Peak Area	Peak Height	Peak Area(%)	Peak Width	Peak Type
1	11.94	364114	6596	50.82%	2.146	BB
2	14.21	352432	7201	49.18%	2.043	BB
Total		716,546	13,797	100.00%		

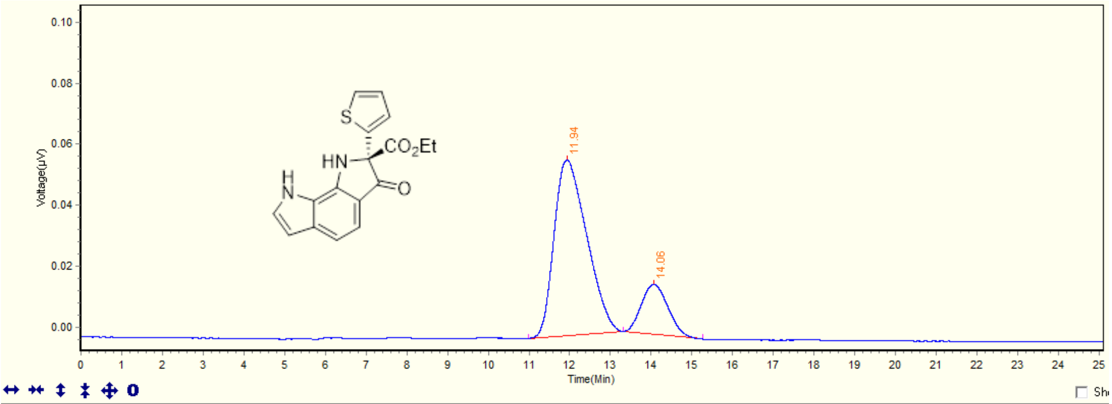

No.	Retention Time	Peak Area	Peak Height	Peak Area(%)	Peak Width	Peak Type
1	11.94	1597205	28765	81.07%	2.313	BB
2	14.06	372975	8174	18.93%	1.948	BB
Total		1,970,180	36,939	100.00%		

HPLC spectra of **4pq**

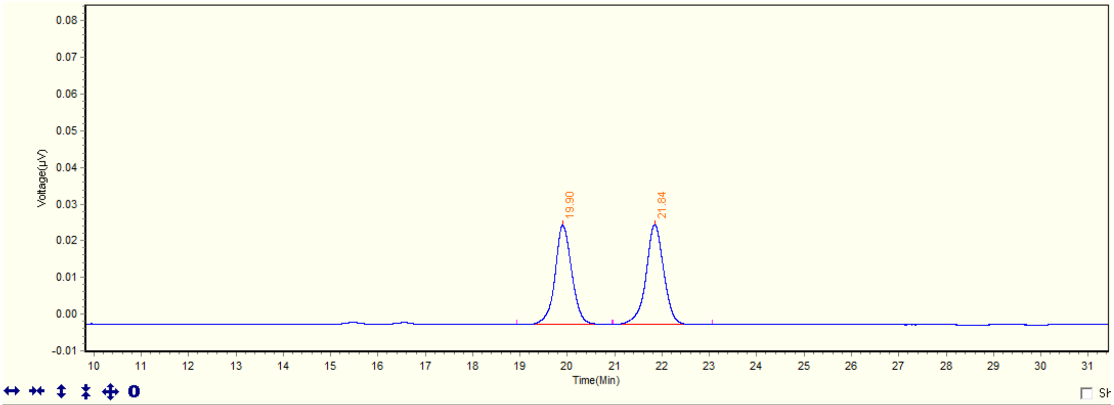

No.	Retention Time	Peak Area	Peak Height	Peak Area(%)	Peak Width	Peak Type
1	19.90	330361	13476	48.41%	2.012	BB
2	21.84	352125	13571	51.59%	2.103	BB
Total		682,486	27,047	100.00%		

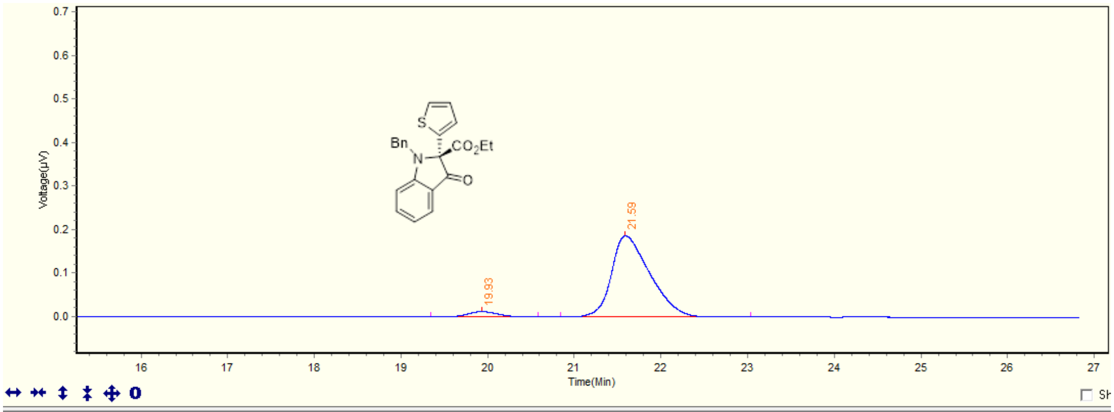

No.	Retention Time	Peak Area	Peak Height	Peak Area(%)	Peak Width	Peak Type
1	19.93	149682	6159	4.94%	1.241	BB
2	21.59	2878240	93263	95.06%	2.194	BB
Total		3,027,922	99,422	100.00%		

HPLC spectra of **4qq**

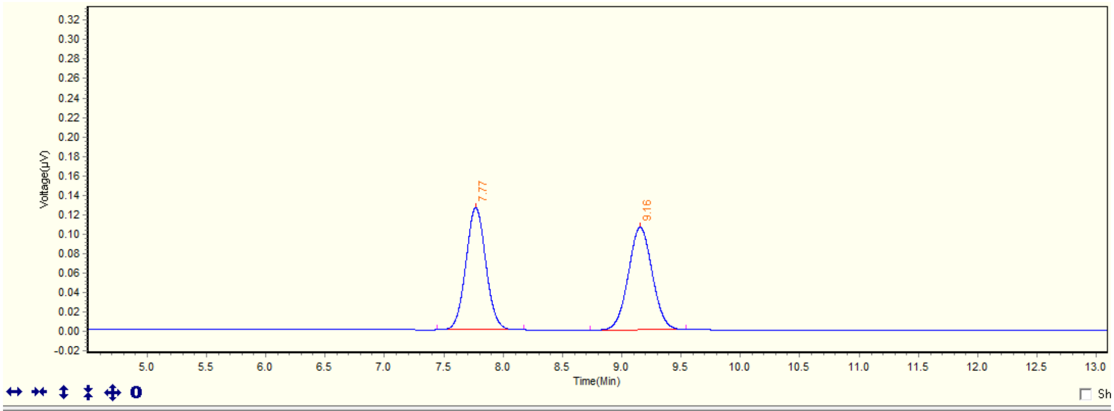

No.	Retention Time	Peak Area	Peak Height	Peak Area(%)	Peak Width	Peak Type
1	7.77	734905	62828	49.99%	0.731	BB
2	9.16	735323	52521	50.01%	0.802	BB
Total		1,470,228	115,349	100.00%		

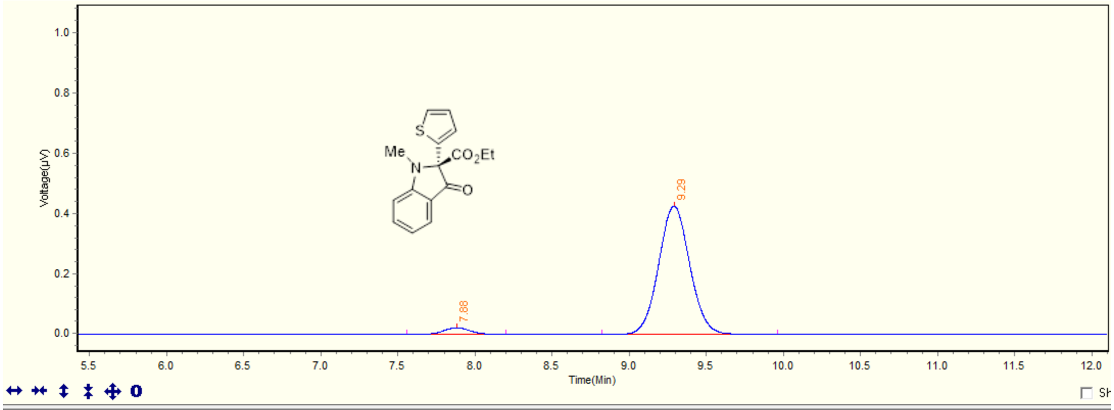

No.	Retention Time	Peak Area	Peak Height	Peak Area(%)	Peak Width	Peak Type
1	7.88	125332	10899	4.03%	0.637	BB
2	9.29	2983684	212722	95.97%	1.139	BB
Total		3,109,016	223,621	100.00%		

HPLC spectra of **4rq**

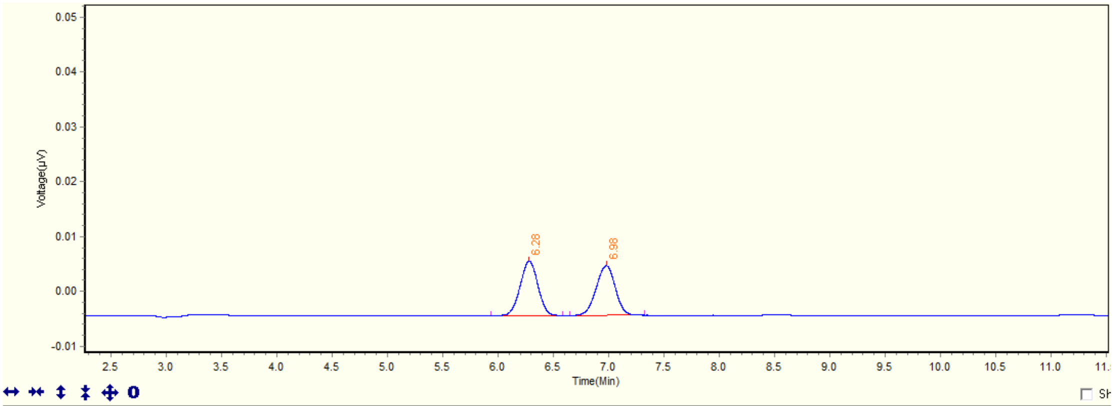

Integration Result | Calculation Result | TimeTable

No.	Retention Time	Peak Area	Peak Height	Peak Area(%)	Peak Width	Peak Type
1	6.28	54779	4952	50.29%	0.648	BB
2	6.98	54141	4518	49.71%	0.677	BB
Total		108,920	9,470	100.00%		

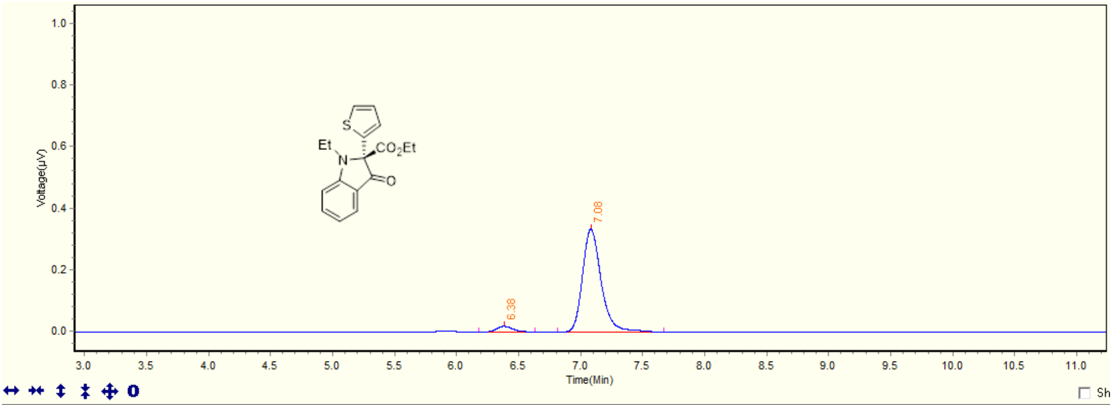

Integration Result | Calculation Result | TimeTable

No.	Retention Time	Peak Area	Peak Height	Peak Area(%)	Peak Width	Peak Type
1	6.38	80036	8981	4.38%	0.456	BB
2	7.08	1746099	167113	95.62%	0.864	BB
Total		1,826,135	176,094	100.00%		

HPLC spectra of **4sq**

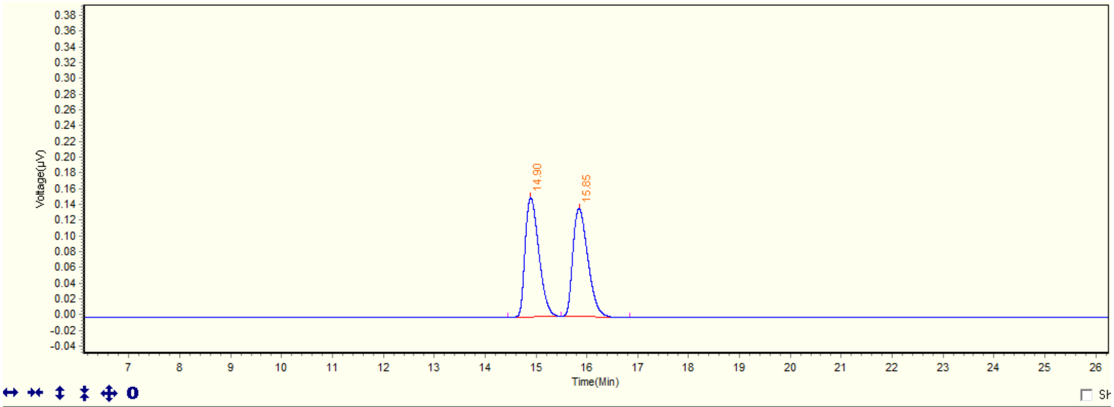

Integration Result | Calculation Result | TimeTable

No.	Retention Time	Peak Area	Peak Height	Peak Area(%)	Peak Width	Peak Type
1	14.90	1419072	75773	50.20%	1.048	BB
2	15.85	1407673	68583	49.80%	1.35	BB
Total		2,826,745	144,356	100.00%		

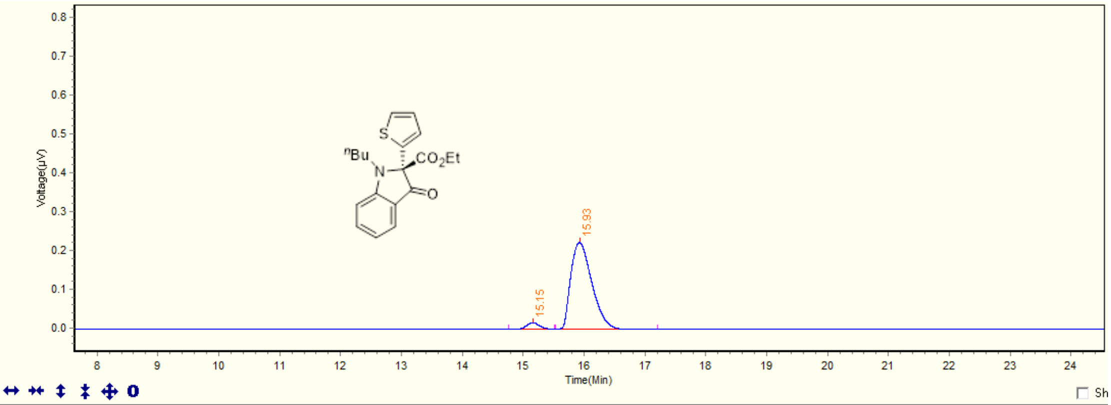

No.	Retention Time	Peak Area	Peak Height	Peak Area(%)	Peak Width	Peak Type
1	15.15	121845	8386	4.36%	0.762	BB
2	15.93	2669715	111361	95.64%	1.671	BB
Total		2,791,560	119,747	100.00%		

HPLC spectra of **4tq**

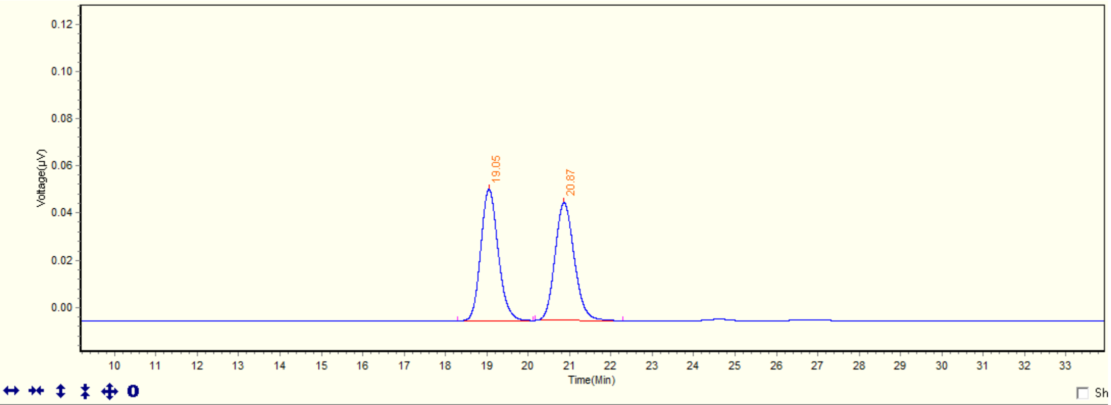

No.	Retention Time	Peak Area	Peak Height	Peak Area(%)	Peak Width	Peak Type
1	19.05	811837	27867	50.05%	1.813	BB
2	20.87	810285	25023	49.95%	2.107	BB
Total		1,622,122	52,890	100.00%		

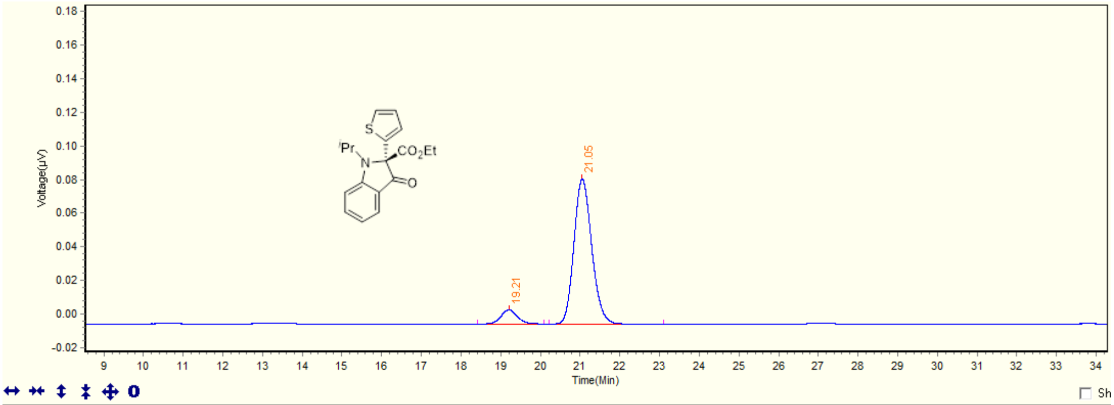

No.	Retention Time	Peak Area	Peak Height	Peak Area(%)	Peak Width	Peak Type
1	19.21	116874	4118	8.01%	1.653	BB
2	21.05	1342163	43027	91.99%	2.892	BB
Total		1,459,037	47,145	100.00%		

HPLC spectra of **4uq**

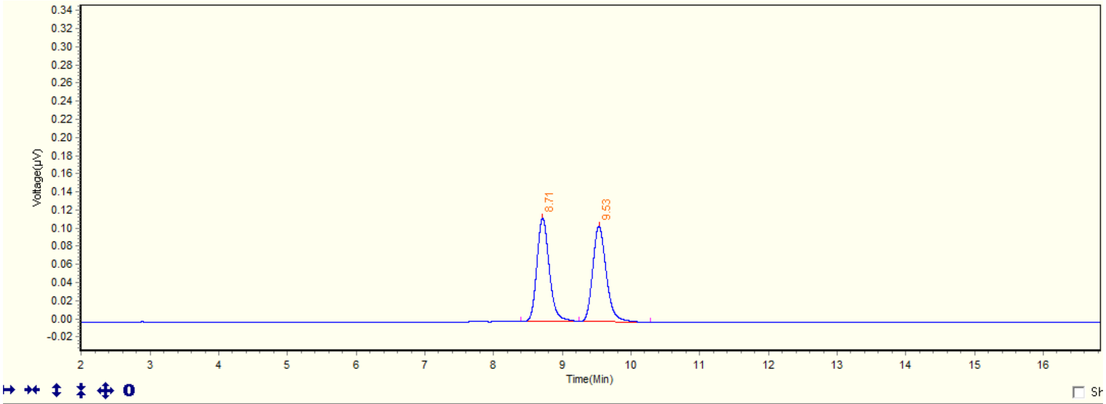

Integration Result	Calculation Result	TimeTable				
No.	Retention Time	Peak Area	Peak Height	Peak Area(%)	Peak Width	Peak Type
1	8.71	714226	56976	49.86%	0.847	BB
2	9.53	718129	52651	50.14%	1.038	BB
Total		1,432,355	109,627	100.00%		

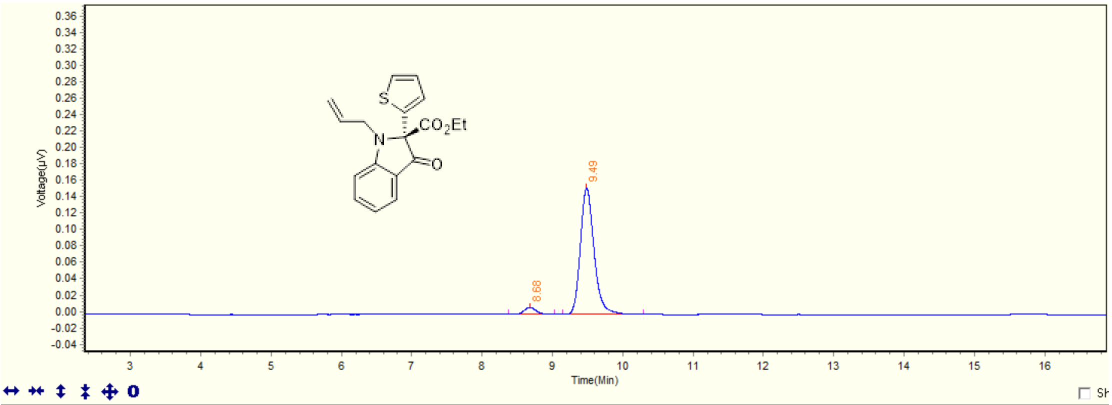

Integration Result	Calculation Result	TimeTable				
No.	Retention Time	Peak Area	Peak Height	Peak Area(%)	Peak Width	Peak Type
1	8.68	50938	4255	4.65%	0.656	BB
2	9.49	1043355	77023	95.35%	1.151	BB
Total		1,094,293	81,278	100.00%		

HPLC spectra of **4vq**

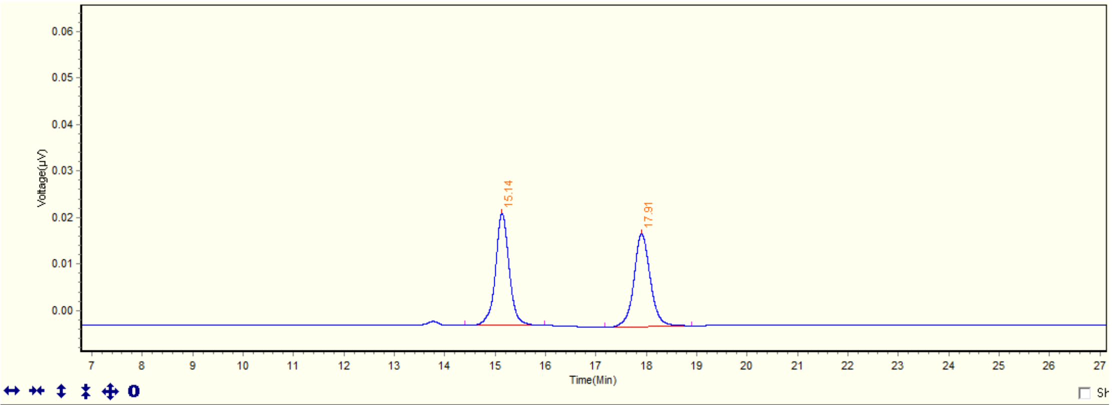

Integration Result	Calculation Result	TimeTable				
No.	Retention Time	Peak Area	Peak Height	Peak Area(%)	Peak Width	Peak Type
1	15.14	227240	12064	50.35%	1.581	BB
2	17.91	224038	9952	49.65%	1.731	BB
Total		451,278	22,016	100.00%		

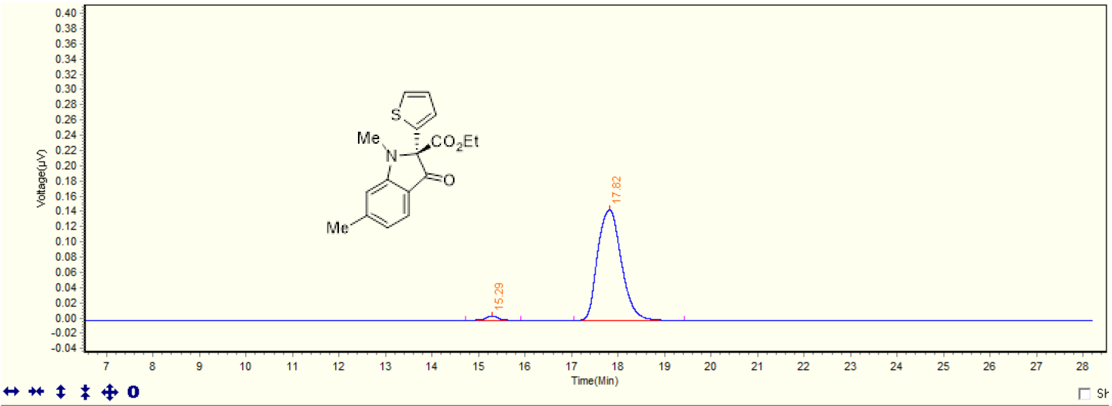

Integration Result | Calculation Result | TimeTable

No.	Retention Time	Peak Area	Peak Height	Peak Area(%)	Peak Width	Peak Type
1	15.29	48917	2769	1.89%	1.179	BB
2	17.82	2543693	72408	98.11%	2.359	BB
Total		2,592,610	75,177	100.00%		

HPLC spectra of **4wq**

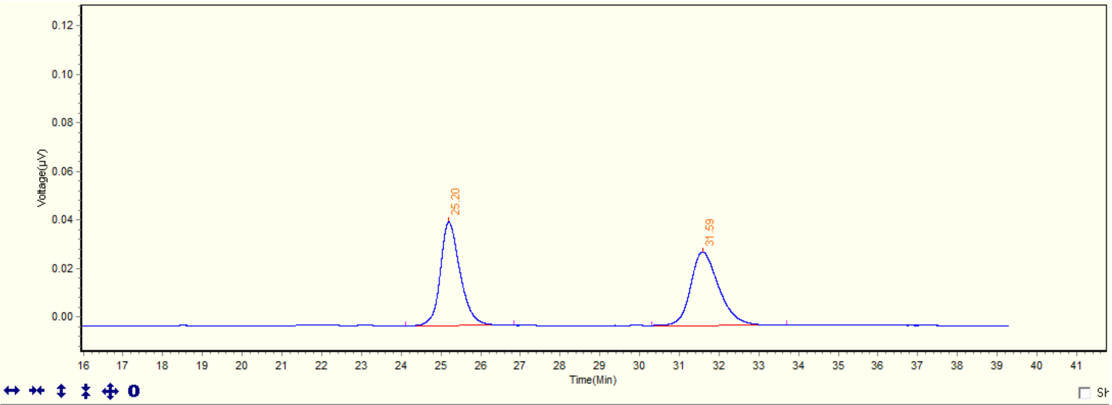

Integration Result | Calculation Result | TimeTable

No.	Retention Time	Peak Area	Peak Height	Peak Area(%)	Peak Width	Peak Type
1	25.20	734368	21281	49.66%	2.712	BB
2	31.59	744389	15146	50.34%	3.404	BB
Total		1,478,757	36,427	100.00%		

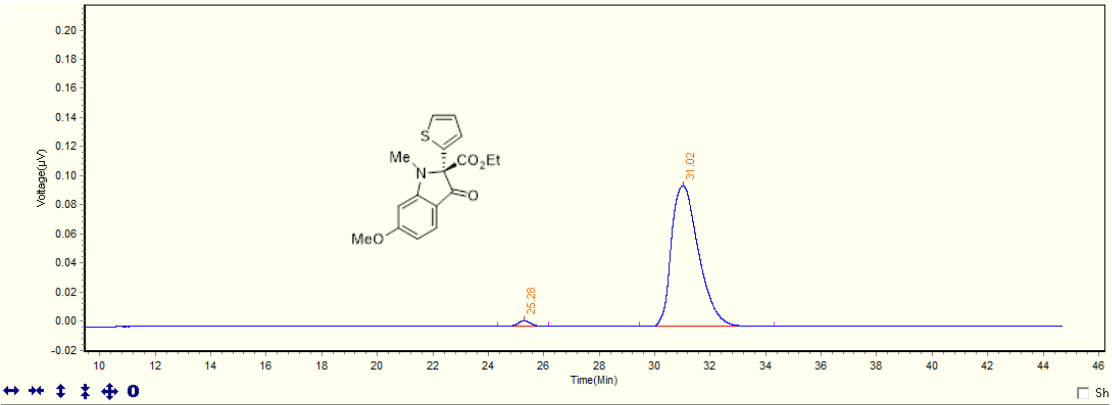

Integration Result | Calculation Result | TimeTable

No.	Retention Time	Peak Area	Peak Height	Peak Area(%)	Peak Width	Peak Type
1	25.28	61213	1962	1.82%	1.845	BB
2	31.02	3299068	48308	98.18%	4.842	BB
Total		3,360,281	50,270	100.00%		

HPLC spectra of **4xq**

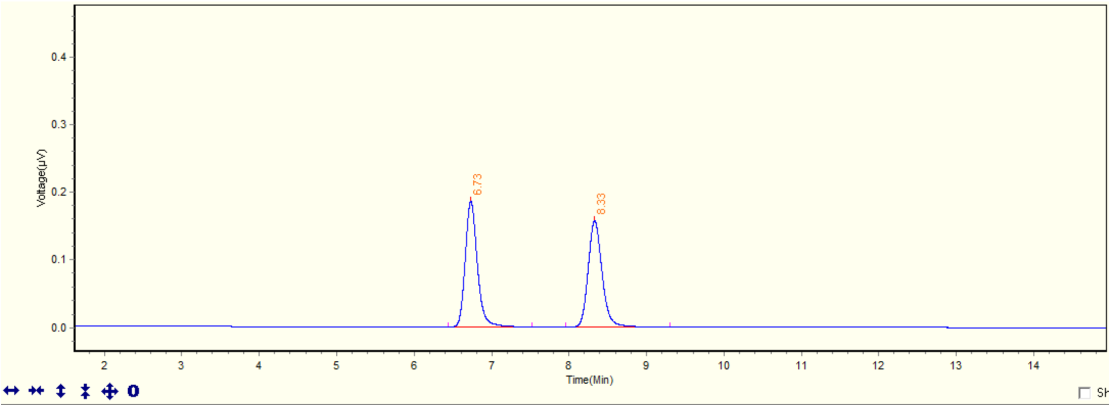

No.	Retention Time	Peak Area	Peak Height	Peak Area(%)	Peak Width	Peak Type
1	6.73	1005557	92832	50.60%	1.082	BB
2	8.33	981602	78427	49.40%	1.348	BB
Total		1,987,159	171,259	100.00%		

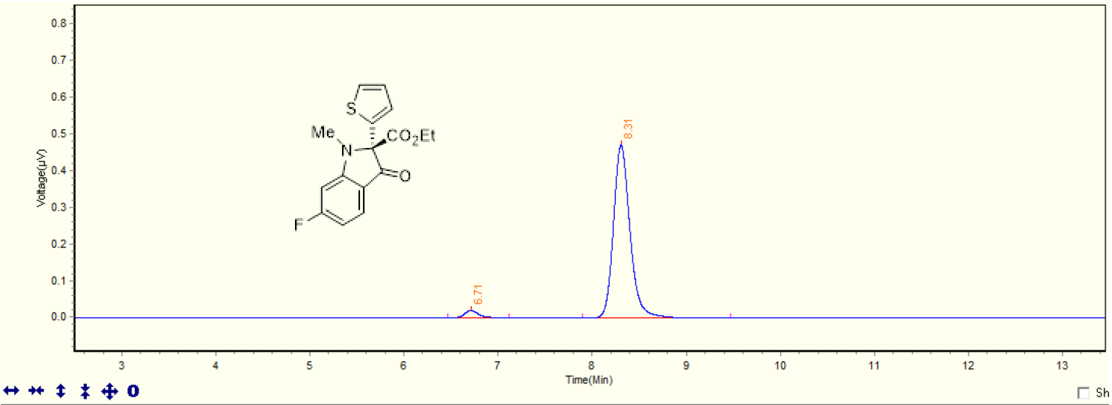

No.	Retention Time	Peak Area	Peak Height	Peak Area(%)	Peak Width	Peak Type
1	6.71	98659	9946	3.35%	0.648	BB
2	8.31	2850623	234791	96.65%	1.578	BB
Total		2,949,282	244,737	100.00%		

HPLC spectra of **4yq**

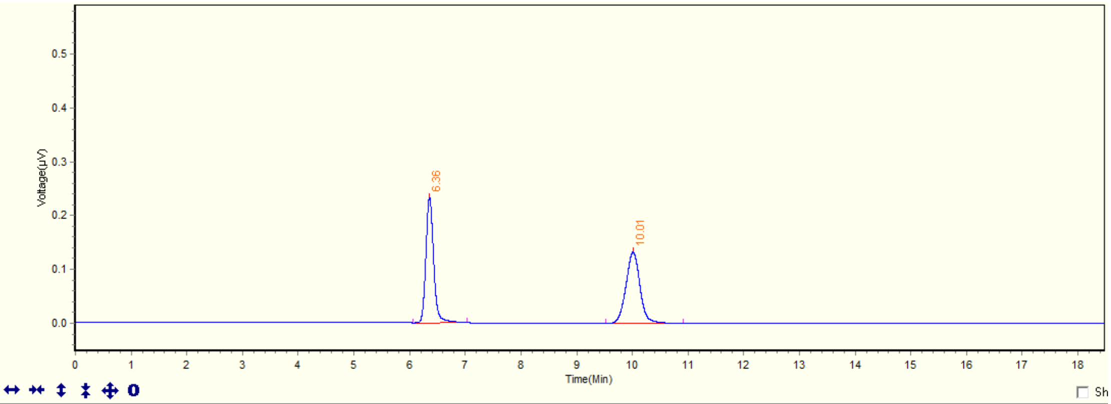

No.	Retention Time	Peak Area	Peak Height	Peak Area(%)	Peak Width	Peak Type
1	6.36	1127943	116347	49.75%	0.969	BB
2	10.01	1139378	65922	50.25%	1.378	BB
Total		2,267,321	182,269	100.00%		

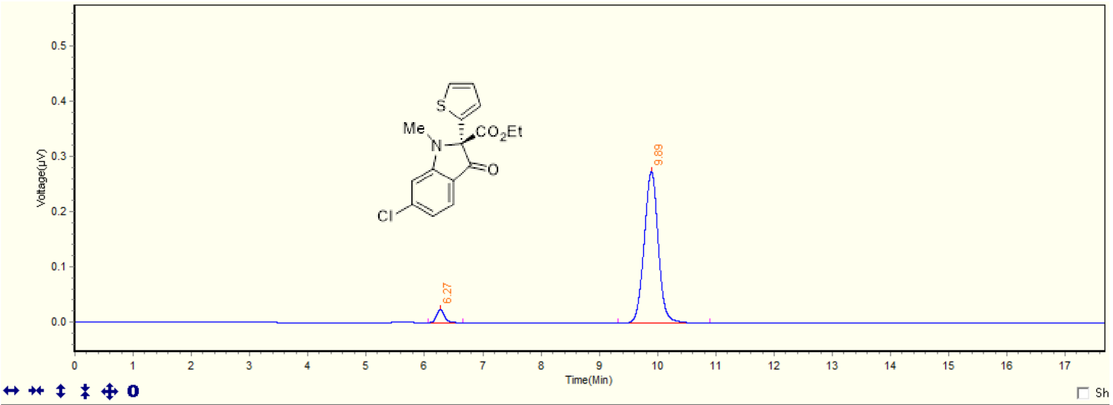

Integration Result | Calculation Result | TimeTable

No.	Retention Time	Peak Area	Peak Height	Peak Area(%)	Peak Width	Peak Type
1	6.27	111317	11700	4.63%	0.598	BB
2	9.89	2290583	137210	95.37%	1.578	BB
Total		2,401,900	148,910	100.00%		

HPLC spectra of **4zq**

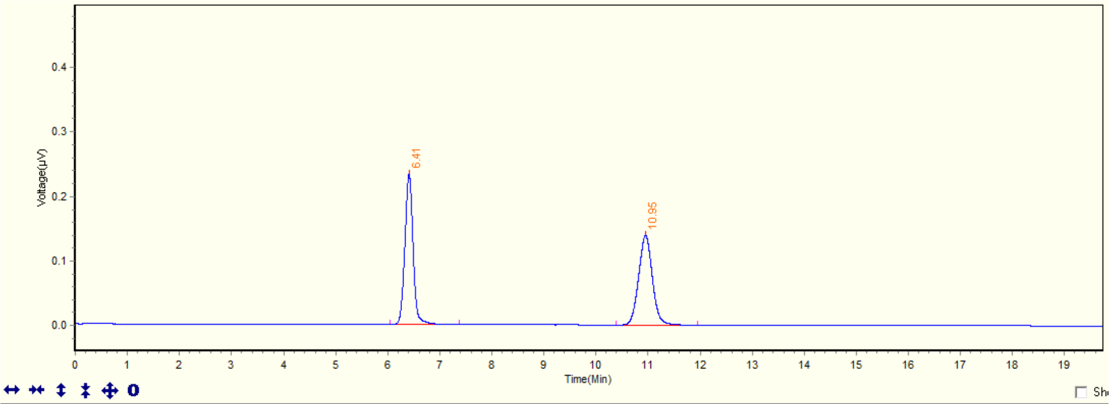

Integration Result | Calculation Result | TimeTable

No.	Retention Time	Peak Area	Peak Height	Peak Area(%)	Peak Width	Peak Type
1	6.41	1280053	116523	50.13%	1.328	BB
2	10.95	1273410	69460	49.87%	1.564	BB
Total		2,553,463	185,983	100.00%		

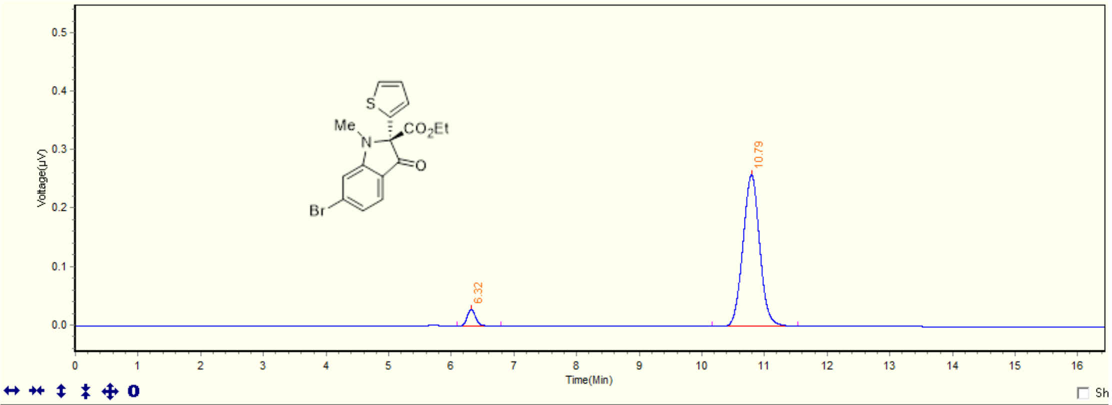

Integration Result | Calculation Result | TimeTable

No.	Retention Time	Peak Area	Peak Height	Peak Area(%)	Peak Width	Peak Type
1	6.32	138855	14456	5.46%	0.704	BB
2	10.79	2403053	129574	94.54%	1.368	BB
Total		2,541,908	144,030	100.00%		

HPLC spectra of **4Aq**

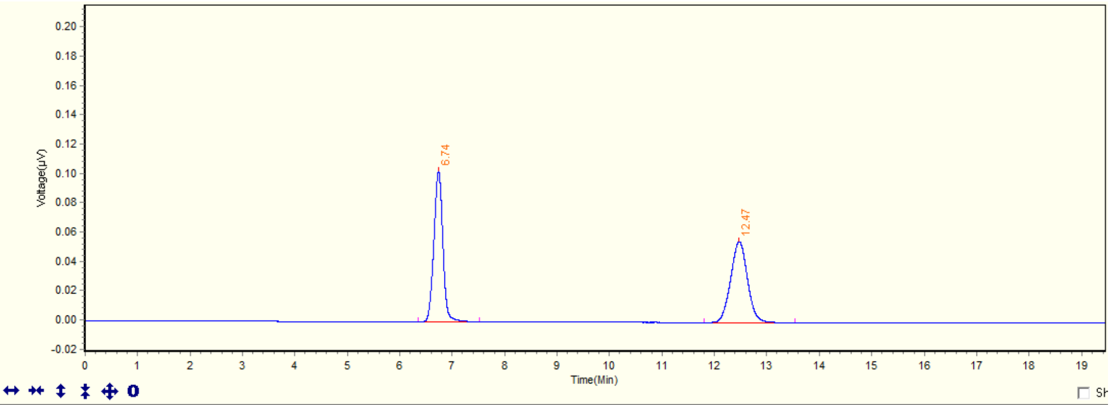

Integration Result | Calculation Result | TimeTable

No.	Retention Time	Peak Area	Peak Height	Peak Area(%)	Peak Width	Peak Type
1	6.74	611771	51410	49.87%	1.185	BB
2	12.47	615076	27642	50.13%	1.728	BB
Total		1,226,847	79,052	100.00%		

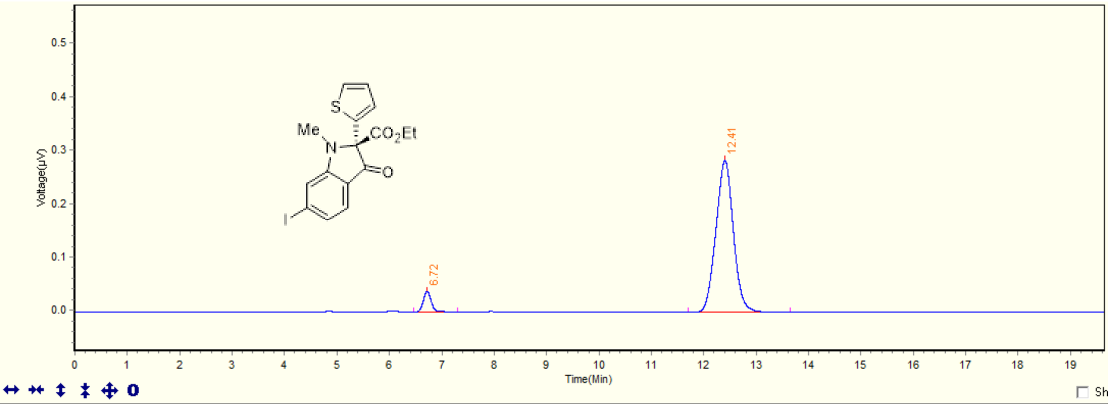

Integration Result | Calculation Result | TimeTable

No.	Retention Time	Peak Area	Peak Height	Peak Area(%)	Peak Width	Peak Type
1	6.72	196865	18899	5.69%	0.834	BB
2	12.41	3260358	141642	94.31%	1.949	BB
Total		3,457,223	160,541	100.00%		

HPLC spectra of **4Bq**

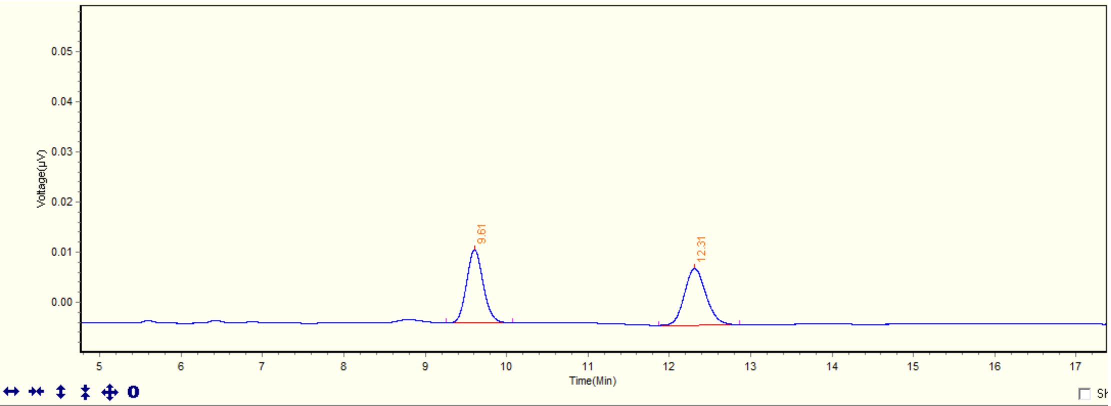

Integration Result | Calculation Result | TimeTable

No.	Retention Time	Peak Area	Peak Height	Peak Area(%)	Peak Width	Peak Type
1	9.61	103138	7286	49.89%	0.804	BB
2	12.31	103604	5638	50.11%	0.993	BB
Total		206,742	12,924	100.00%		

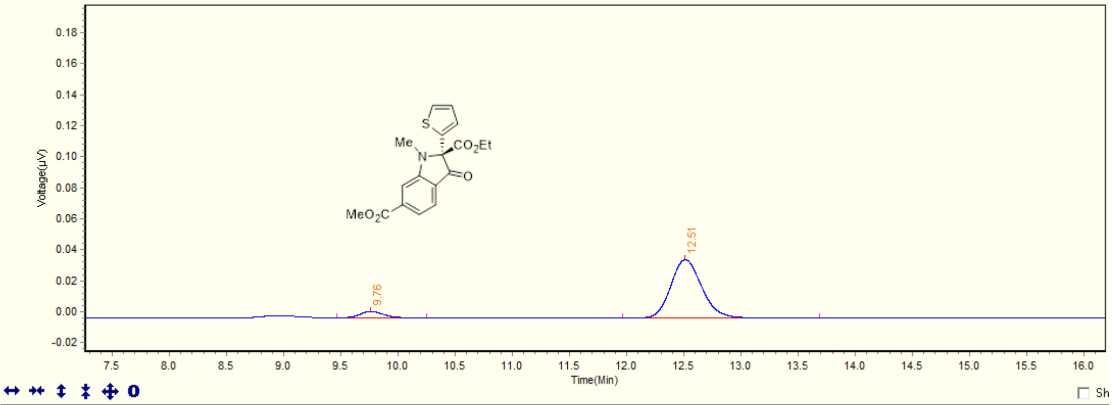

Integration Result | Calculation Result | TimeTable

No.	Retention Time	Peak Area	Peak Height	Peak Area(%)	Peak Width	Peak Type
1	9.76	29431	2056	7.70%	0.783	BB
2	12.51	352559	18807	92.30%	1.731	BB
Total		381,990	20,863	100.00%		

HPLC spectra of **4Cq**

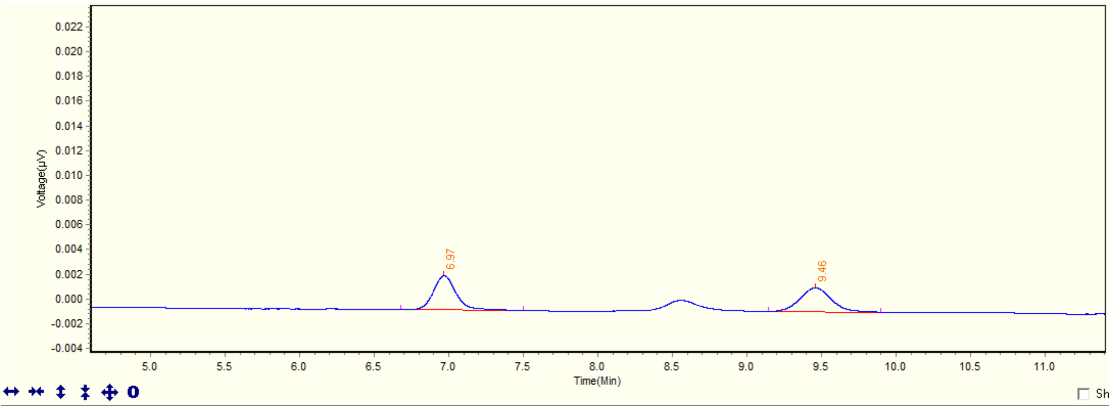

Integration Result | Calculation Result | TimeTable

No.	Retention Time	Peak Area	Peak Height	Peak Area(%)	Peak Width	Peak Type
1	6.97	14542	1372	51.04%	0.822	BB
2	9.46	13948	963	48.96%	0.756	BB
Total		28,490	2,335	100.00%		

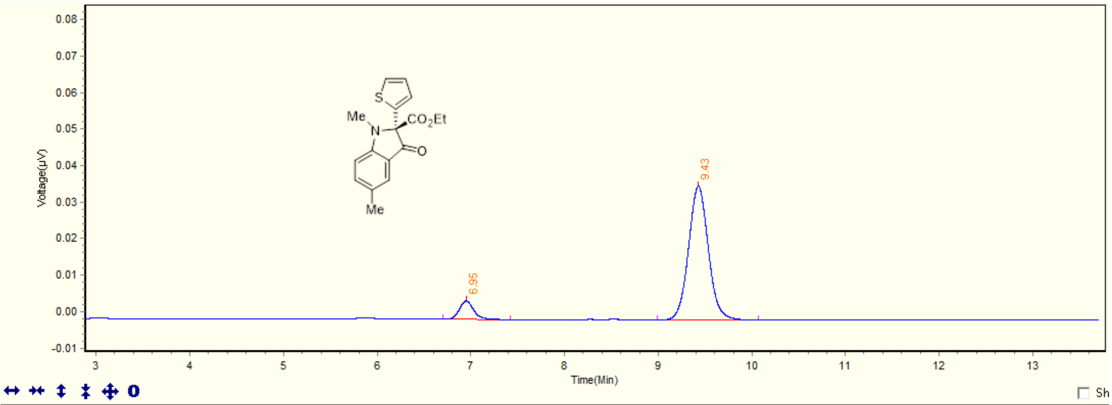

Integration Result | Calculation Result | TimeTable

No.	Retention Time	Peak Area	Peak Height	Peak Area(%)	Peak Width	Peak Type
1	6.95	27076	2529	9.18%	0.716	BB
2	9.43	267840	18331	90.82%	1.081	BB
Total		294,916	20,860	100.00%		

HPLC spectra of **4Fq**

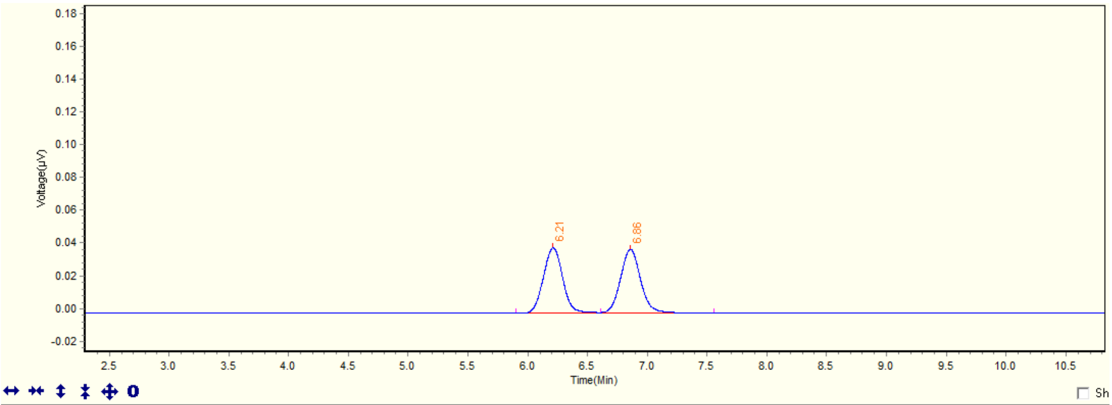

Integration Result | Calculation Result | TimeTable

No.	Retention Time	Peak Area	Peak Height	Peak Area(%)	Peak Width	Peak Type
1	6.86	222916	19387	50.31%	0.943	BB
2	6.21	220180	19913	49.69%	0.712	BB
Total		443,096	39,300	100.00%		

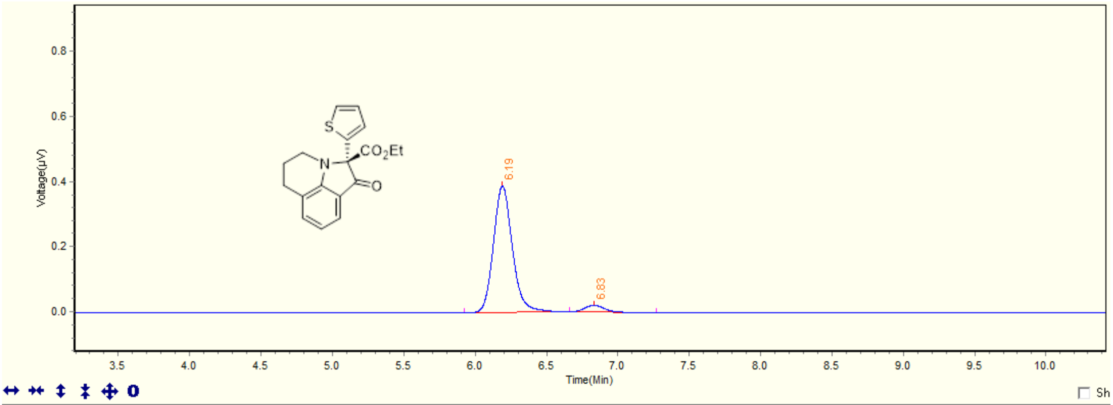

Integration Result | Calculation Result | TimeTable

No.	Retention Time	Peak Area	Peak Height	Peak Area(%)	Peak Width	Peak Type
1	6.19	1791562	194000	94.65%	0.738	BB
2	6.83	101184	10551	5.35%	0.607	BB
Total		1,892,746	204,551	100.00%		

HPLC spectra of **4Gq**

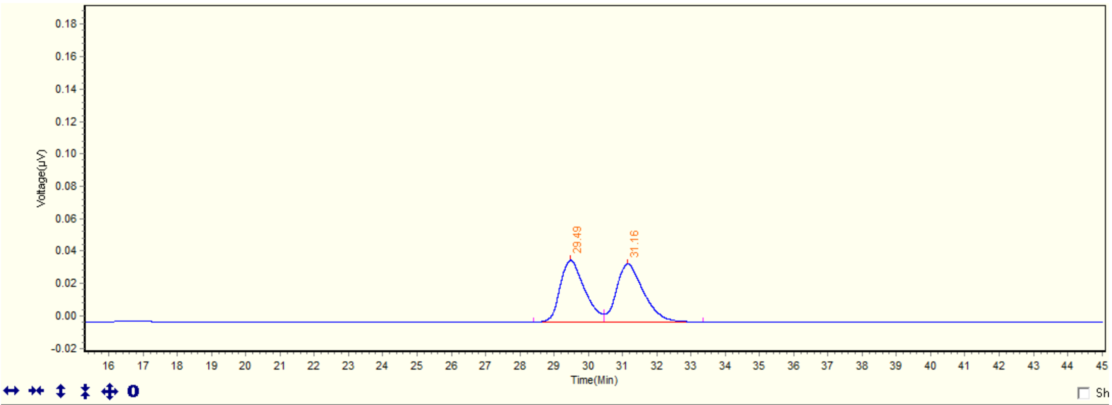

Integration Result | Calculation Result | TimeTable

No.	Retention Time	Peak Area	Peak Height	Peak Area(%)	Peak Width	Peak Type
1	29.49	941607	19065	48.88%	2.05	BV
2	31.16	984944	17993	51.12%	2.902	VB
Total		1,926,551	37,058	100.00%		

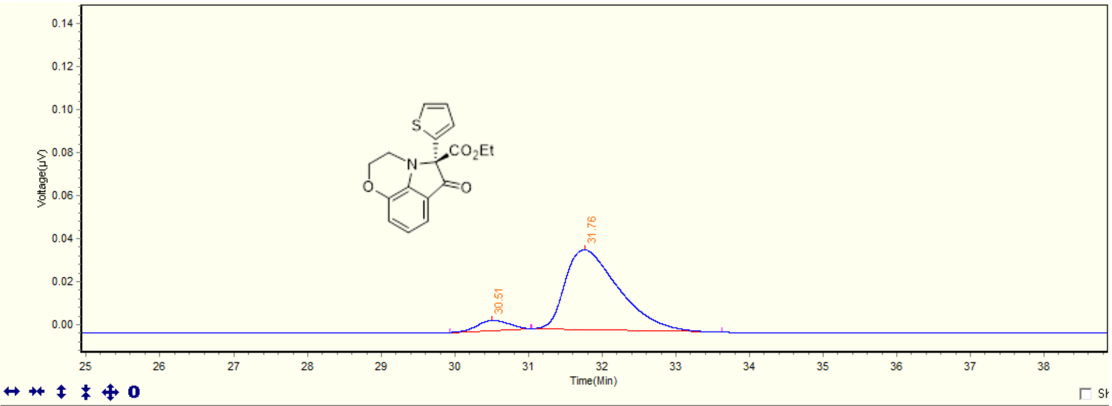

Integration Result | Calculation Result | TimeTable

No.	Retention Time	Peak Area	Peak Height	Peak Area(%)	Peak Width	Peak Type
1	30.51	68082	2337	6.77%	1.101	BB
2	31.76	937729	18581	93.23%	2.588	BB
Total		1,005,811	20,918	100.00%		

HPLC spectra of **4Hq**

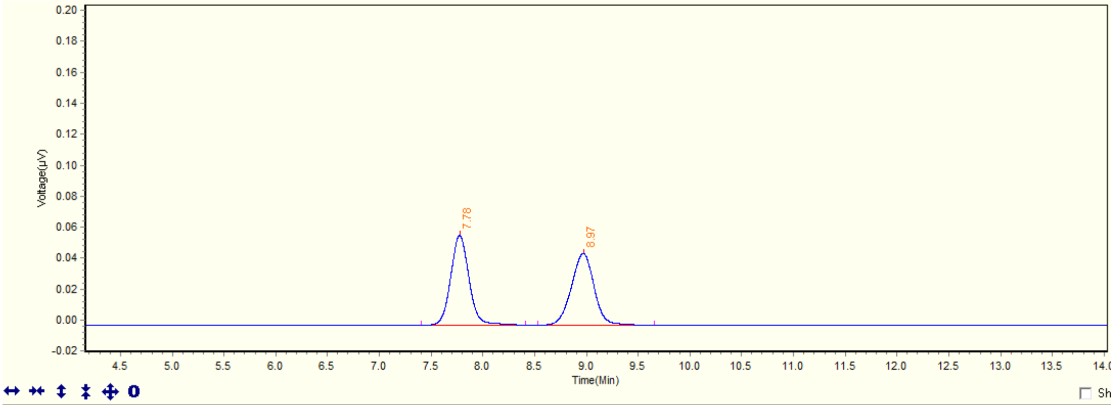

Integration Result | Calculation Result | TimeTable

No.	Retention Time	Peak Area	Peak Height	Peak Area(%)	Peak Width	Peak Type
1	7.78	351519	28863	50.15%	1.01	BB
2	8.97	349372	23023	49.85%	1.131	BB
Total		700,891	51,886	100.00%		

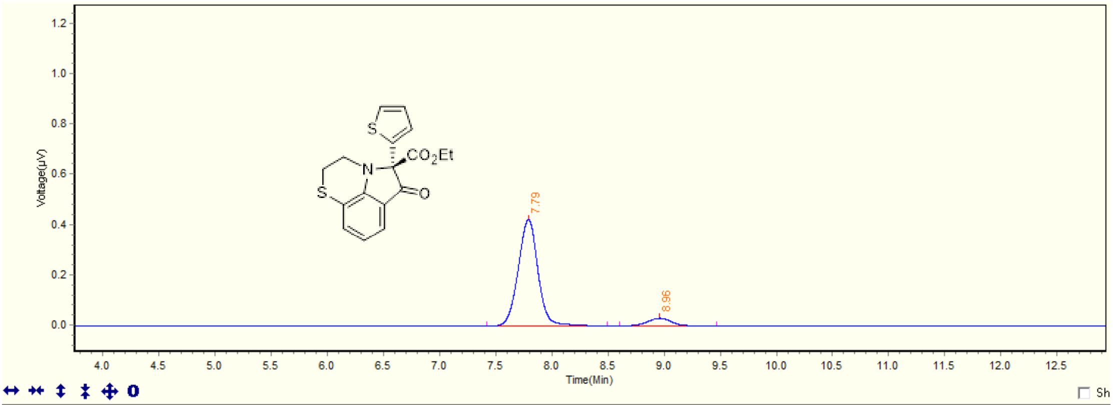

Integration Result | Calculation Result | TimeTable

No.	Retention Time	Peak Area	Peak Height	Peak Area(%)	Peak Width	Peak Type
1	7.79	2570911	210578	92.09%	1.075	BB
2	8.96	220719	14981	7.91%	0.859	BB
Total		2,791,630	225,559	100.00%		

HPLC spectra of **4Iq**

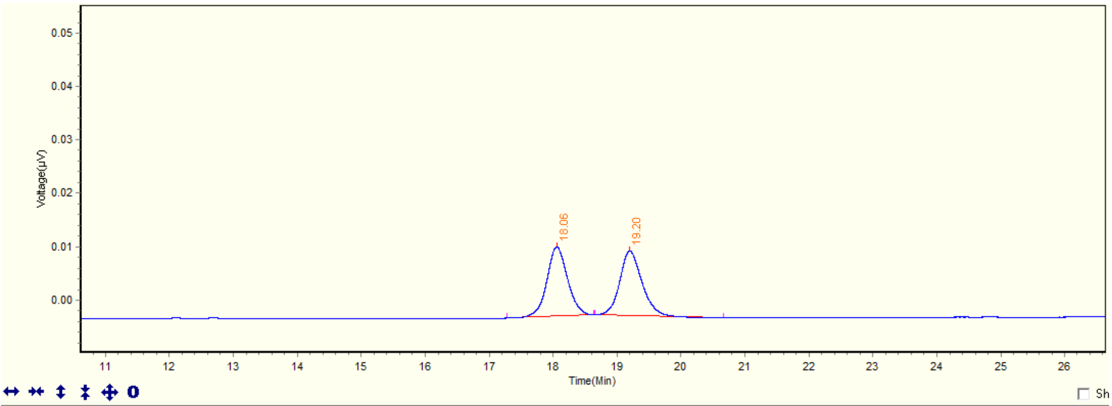

No.	Retention Time	Peak Area	Peak Height	Peak Area(%)	Peak Width	Peak Type
1	18.06	145807	6488	49.87%	1.366	BB
2	19.20	146570	6061	50.13%	2.02	BB
Total		292,377	12,549	100.00%		

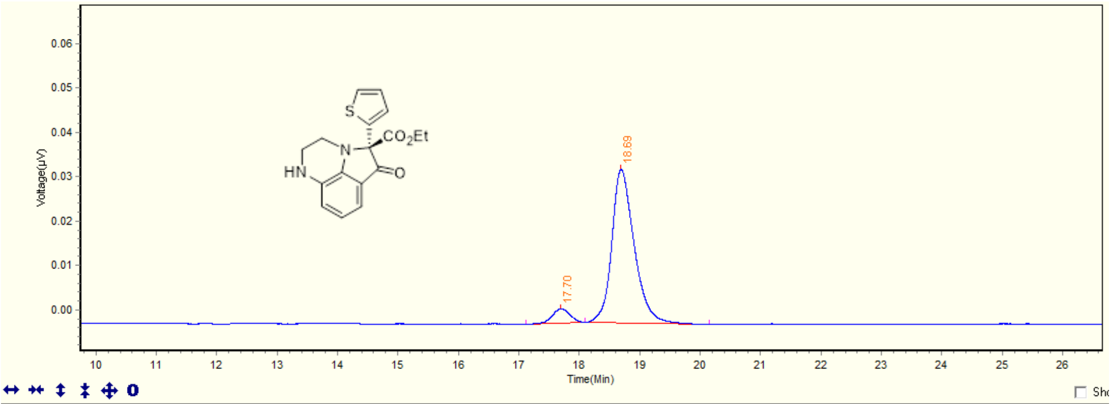

No.	Retention Time	Peak Area	Peak Height	Peak Area(%)	Peak Width	Peak Type
1	18.69	446331	17318	93.17%	2.053	BB
2	17.70	32728	1614	6.83%	0.972	BB
Total		479,059	18,932	100.00%		

HPLC spectra of **4Jq**

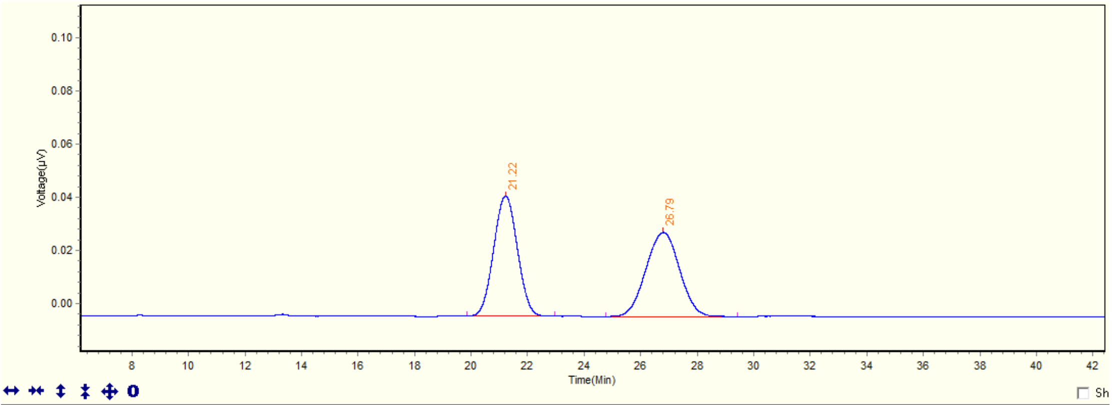

No.	Retention Time	Peak Area	Peak Height	Peak Area(%)	Peak Width	Peak Type
1	21.22	1318849	22635	50.29%	3.093	BB
2	26.79	1303749	15809	49.71%	4.667	BB
Total		2,622,598	38,444	100.00%		

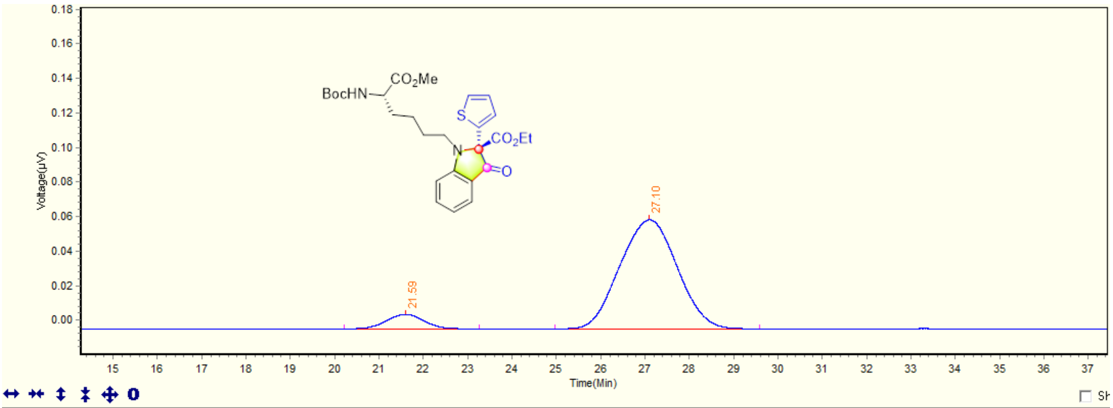

No.	Retention Time	Peak Area	Peak Height	Peak Area(%)	Peak Width	Peak Type
1	21.59	242051	4168	7.89%	3.051	BB
2	27.10	2826868	31475	92.11%	4.632	BB
Total		3,068,919	35,643	100.00%		

HPLC spectra of **4Kq**

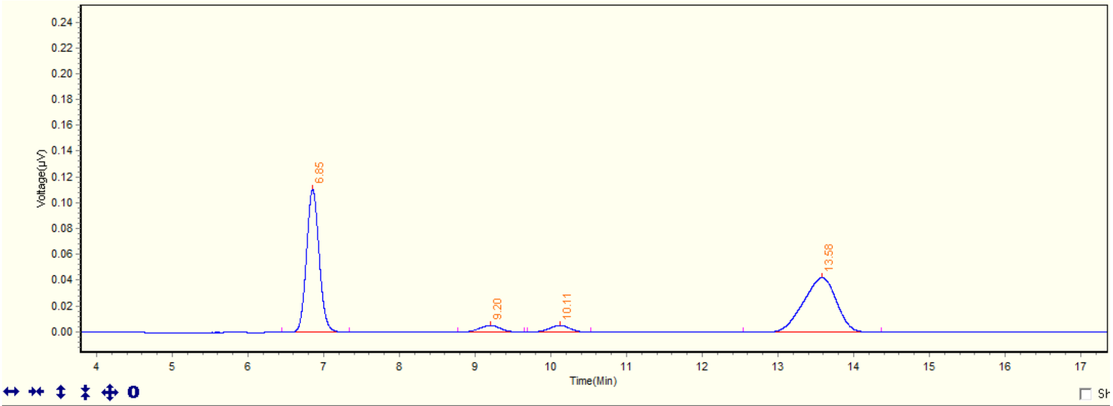

No.	Retention Time	Peak Area	Peak Height	Peak Area(%)	Peak Width	Peak Type
1	6.85	644840	55456	46.48%	0.898	BB
2	9.20	51071	2640	3.68%	0.874	BB
3	10.11	48211	2616	3.47%	0.84	BB
4	13.58	643271	21169	46.37%	1.818	BB
Total		1,387,393	81,881	100.00%		

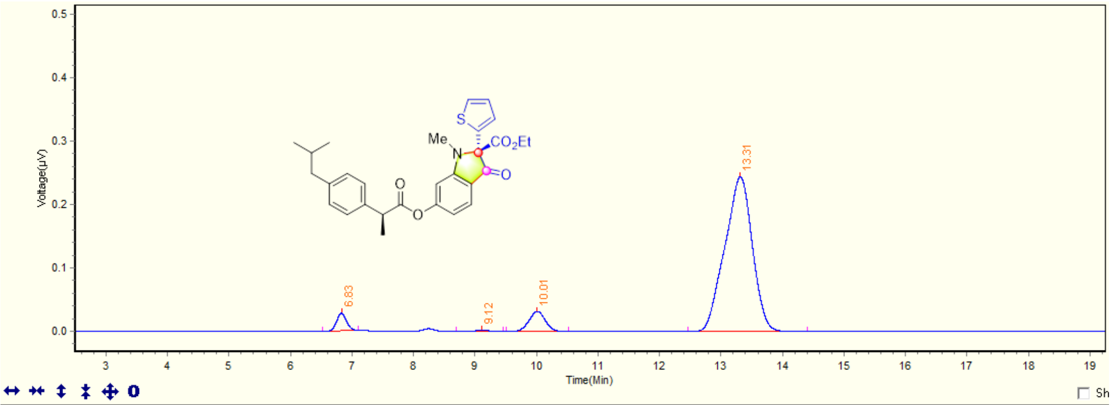

No.	Retention Time	Peak Area	Peak Height	Peak Area(%)	Peak Width	Peak Type
1	6.83	154009	13968	3.62%	0.57	BB
2	9.12	13370	722	0.31%	0.754	BB
3	10.01	288338	15694	6.78%	1.011	BB
4	13.31	3796215	121958	89.28%	1.944	BB
Total		4,251,932	152,342	100.00%		

HPLC spectra of **4Lq**

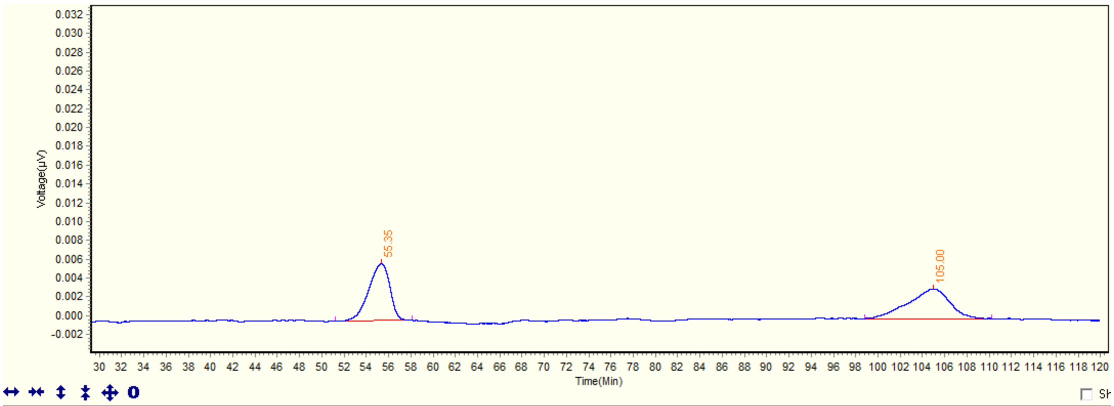

Integration Result | Calculation Result | TimeTable

No.	Retention Time	Peak Area	Peak Height	Peak Area(%)	Peak Width	Peak Type
1	55.35	404183	3044	49.79%	6.853	BB
2	105.00	407576	1586	50.21%	11.394	BB
Total		811,759	4,630	100.00%		

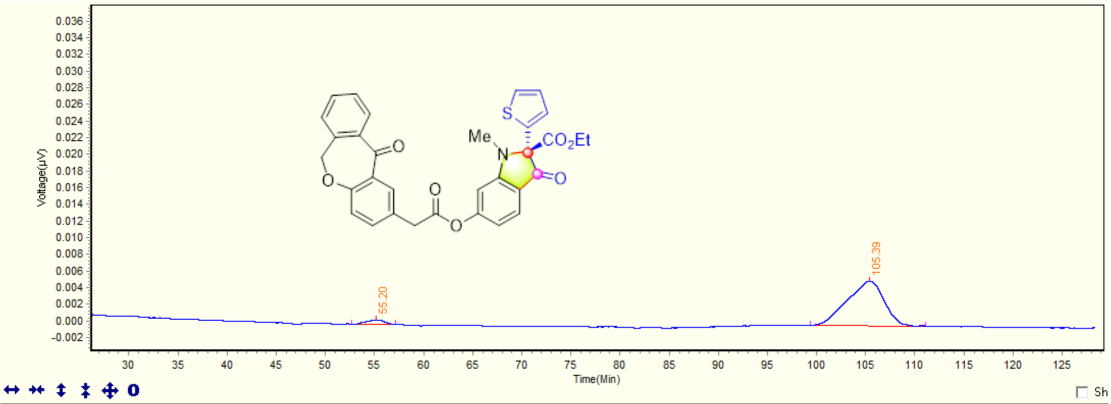

Integration Result | Calculation Result | TimeTable

No.	Retention Time	Peak Area	Peak Height	Peak Area(%)	Peak Width	Peak Type
1	55.20	36557	276	4.97%	4.408	BB
2	105.39	698479	2677	95.03%	11.775	BB
Total		735,036	2,953	100.00%		

HPLC spectra of **4Mq**

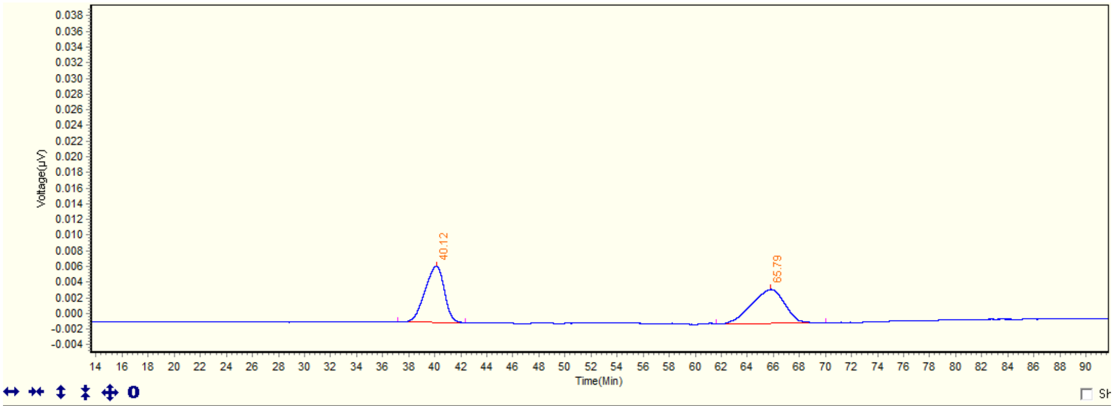

Integration Result | Calculation Result | TimeTable

No.	Retention Time	Peak Area	Peak Height	Peak Area(%)	Peak Width	Peak Type
1	40.12	368242	3580	49.35%	5.213	BB
2	65.79	377930	2156	50.65%	8.382	BB
Total		746,172	5,736	100.00%		

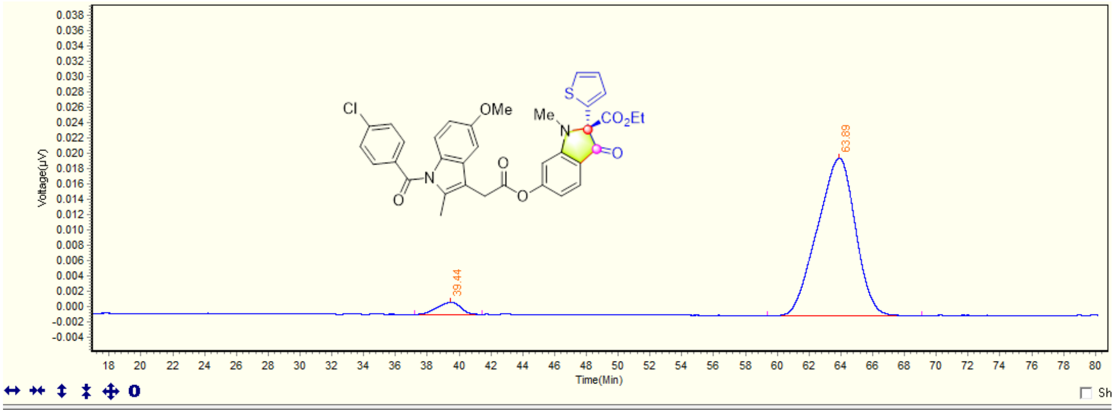

No.	Retention Time	Peak Area	Peak Height	Peak Area(%)	Peak Width	Peak Type
1	39.44	79857	789	4.46%	4.21	BB
2	63.89	1708907	10301	95.54%	9.702	BB
Total		1,788,764	11,090	100.00%		

HPLC spectra of **4Nq**

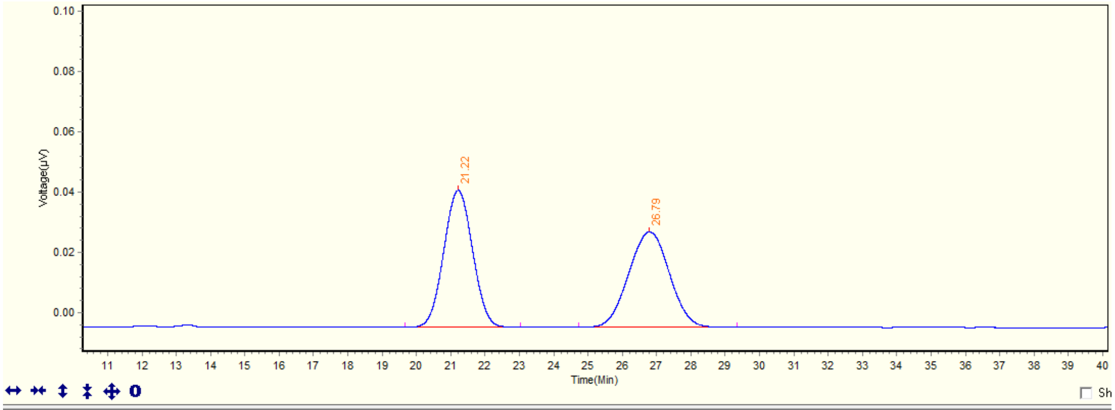

No.	Retention Time	Peak Area	Peak Height	Peak Area(%)	Peak Width	Peak Type
1	21.22	1320549	22644	50.32%	3.363	BB
2	26.79	1303648	15808	49.68%	4.591	BB
Total		2,624,197	38,452	100.00%		

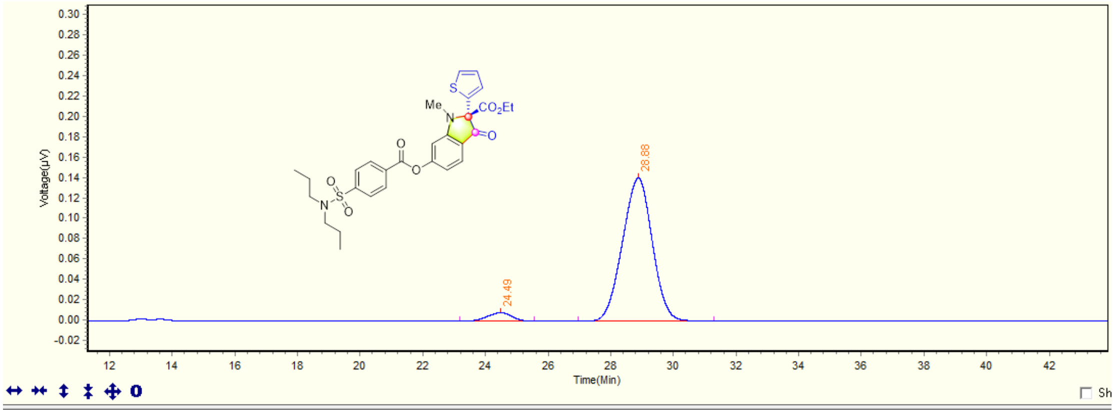

No.	Retention Time	Peak Area	Peak Height	Peak Area(%)	Peak Width	Peak Type
1	24.49	220029	4091	4.54%	2.38	BB
2	28.88	4627035	70155	95.46%	4.333	BB
Total		4,847,064	74,246	100.00%		

HPLC spectra of **3aq**

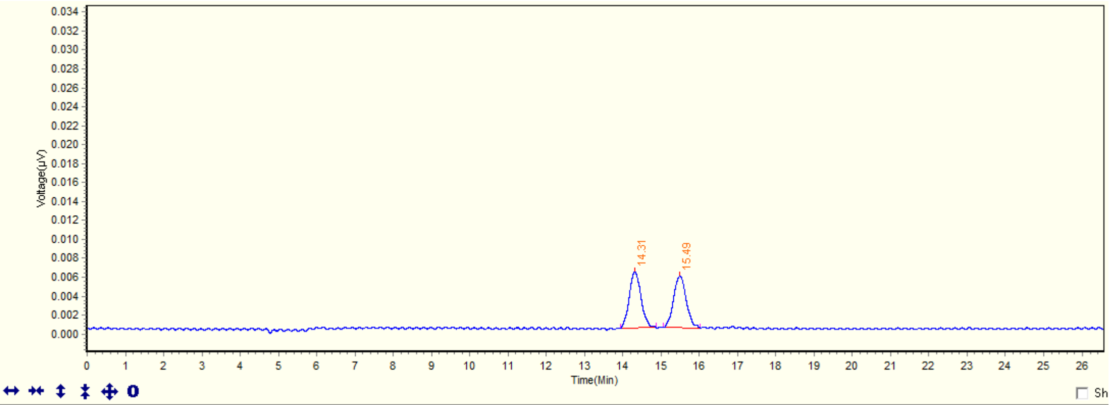

Integration Result | Calculation Result | TimeTable

No.	Retention Time	Peak Area	Peak Height	Peak Area(%)	Peak Width	Peak Type
1	14.31	62781	2940	50.00%	0.908	BB
2	15.49	62784	2723	50.00%	0.954	BB
Total		125,565	5,663	100.00%		

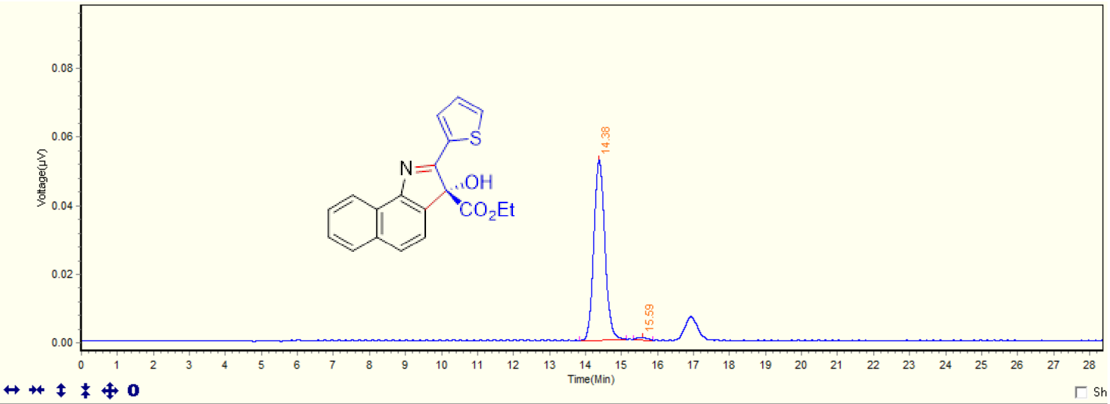

Integration Result | Calculation Result | TimeTable

No.	Retention Time	Peak Area	Peak Height	Peak Area(%)	Peak Width	Peak Type
1	14.38	560038	26279	98.83%	1.314	BB
2	15.59	6629	369	1.17%	0.549	BB
Total		566,667	26,648	100.00%		

HPLC spectra of **8**

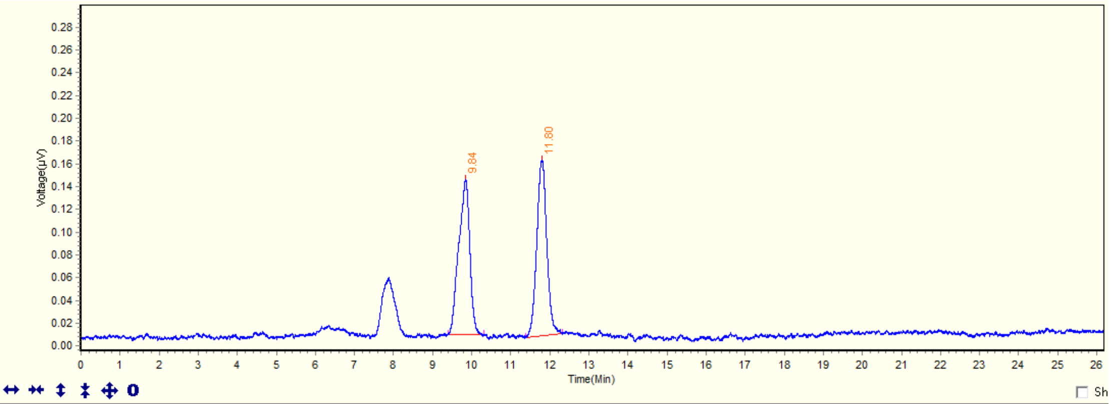

Integration Result | Calculation Result | TimeTable

No.	Retention Time	Peak Area	Peak Height	Peak Area(%)	Peak Width	Peak Type
1	9.84	1331774	67907	49.37%	0.919	BB
2	11.80	1365955	76978	50.63%	0.923	BB
Total		2,697,729	144,885	100.00%		

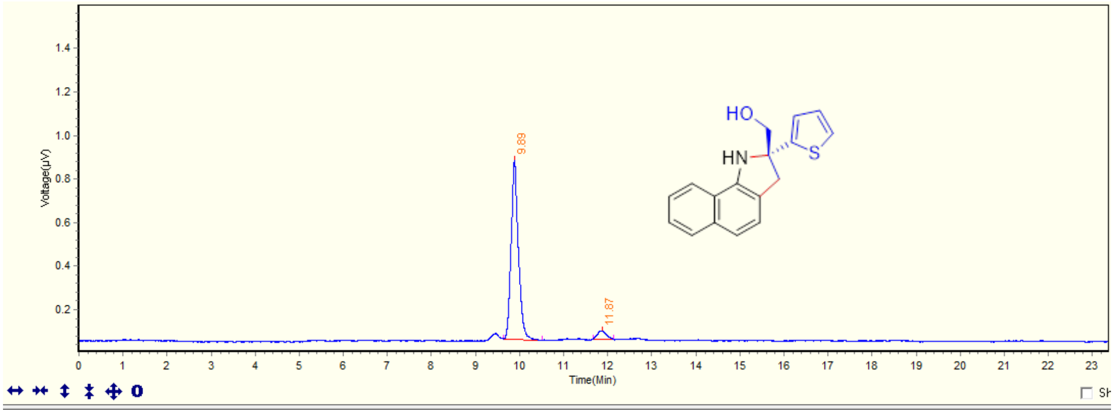

No.	Retention Time	Peak Area	Peak Height	Peak Area(%)	Peak Width	Peak Type
1	9.89	4705122	412016	94.78%	0.893	BB
2	11.87	258890	18582	5.22%	0.463	BB
Total		4,964,012	430,598	100.00%		

HPLC spectra of **9**

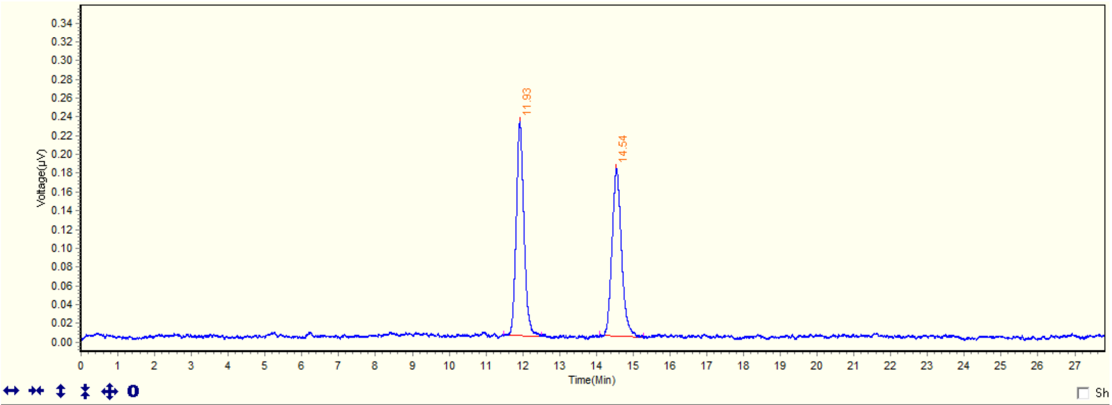

No.	Retention Time	Peak Area	Peak Height	Peak Area(%)	Peak Width	Peak Type
1	11.93	1666161	113999	50.77%	1.027	BB
2	14.54	1615434	89238	49.23%	1.193	BB
Total		3,281,595	203,237	100.00%		

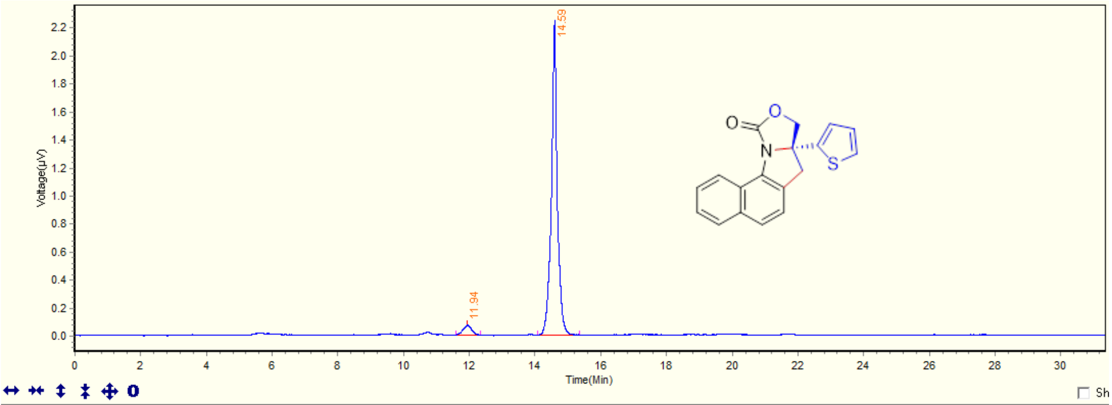

No.	Retention Time	Peak Area	Peak Height	Peak Area(%)	Peak Width	Peak Type
1	11.94	642509	34920	4.40%	0.754	BB
2	14.59	13958900	1120755	95.60%	1.254	BB
Total		14,601,409	1,155,675	100.00%		

HPLC spectra of **10**

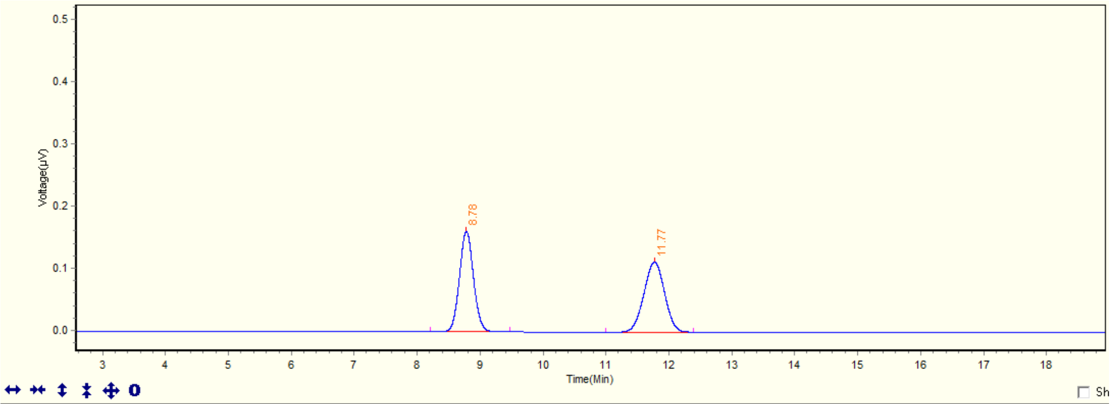

Integration Result | Calculation Result | TimeTable

No.	Retention Time	Peak Area	Peak Height	Peak Area(%)	Peak Width	Peak Type
1	8.78	1250484	80654	49.51%	1.256	BB
2	11.77	1275471	56354	50.49%	1.392	BB
Total		2,525,955	137,008	100.00%		

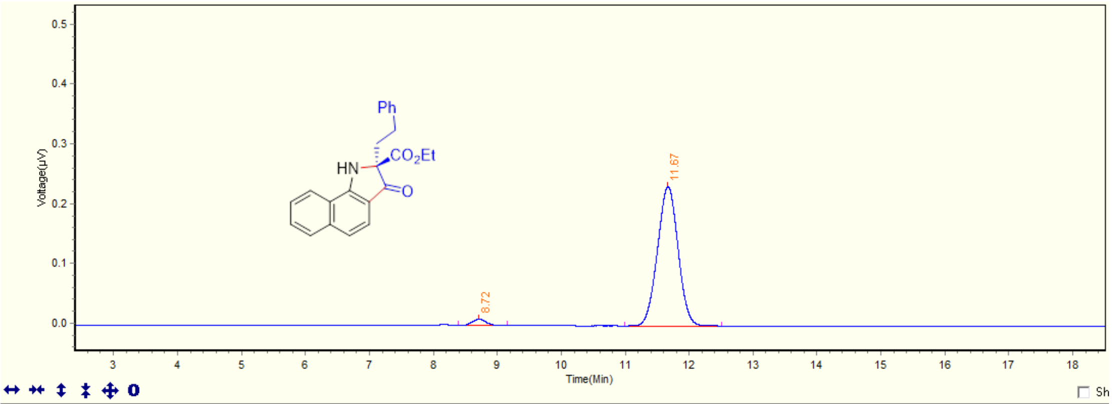

Integration Result | Calculation Result | TimeTable

No.	Retention Time	Peak Area	Peak Height	Peak Area(%)	Peak Width	Peak Type
1	8.72	81230	5312	3.03%	0.755	BB
2	11.67	2596701	116420	96.97%	1.528	BB
Total		2,677,931	121,732	100.00%		

HPLC spectra of **11**

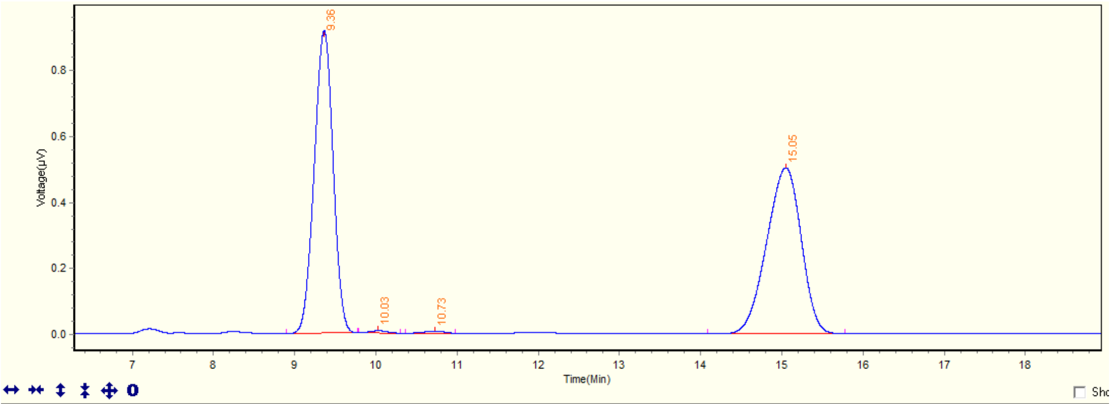

Integration Result | Calculation Result | TimeTable

No.	Retention Time	Peak Area	Peak Height	Peak Area(%)	Peak Width	Peak Type
1	9.36	7411634	458496	49.66%	0.877	BB
2	10.03	51010	3441	0.34%	0.514	BB
3	10.73	53489	3040	0.36%	0.615	BB
4	15.05	7408496	251122	49.64%	1.683	BB
Total		14,924,629	716,099	100.00%		

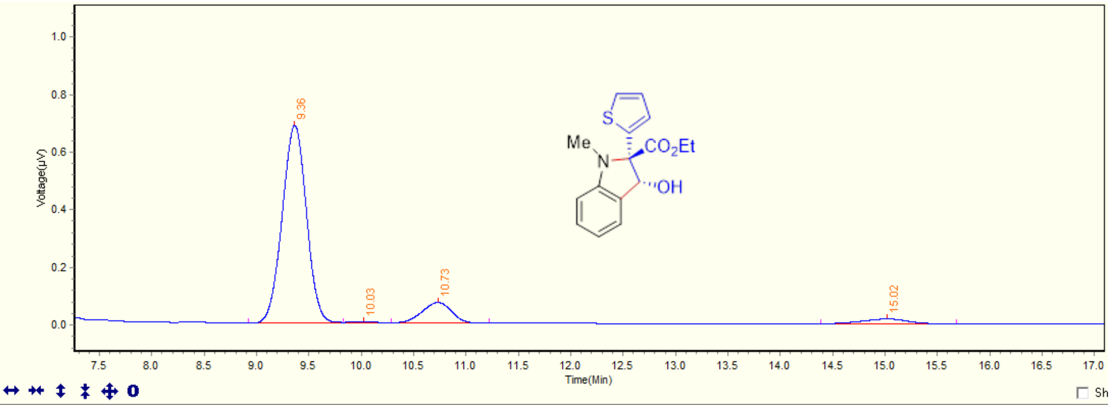

Integration Result | Calculation Result | TimeTable

No.	Retention Time	Peak Area	Peak Height	Peak Area(%)	Peak Width	Peak Type
1	9.36	5503685	343323	85.44%	0.901	BB
2	10.03	16073	1197	0.25%	0.452	BB
3	10.73	685941	35529	10.65%	0.938	BB
4	15.02	235641	8096	3.66%	1.291	BB
Total		6,441,340	388,145	100.00%		

HPLC spectra of **12**

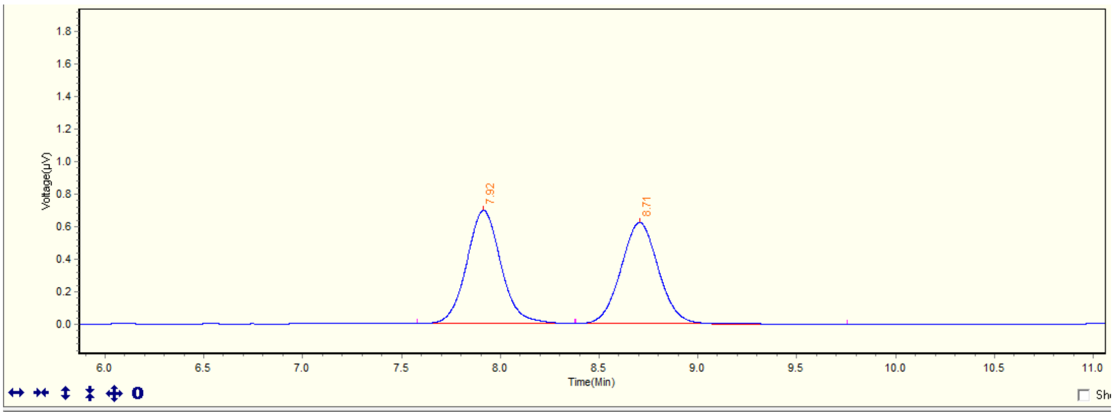

Integration Result | Calculation Result | TimeTable

No.	Retention Time	Peak Area	Peak Height	Peak Area(%)	Peak Width	Peak Type
1	7.92	4193120	347879	50.63%	0.799	BB
2	8.71	4088298	310767	49.37%	1.376	BB
Total		8,281,418	658,646	100.00%		

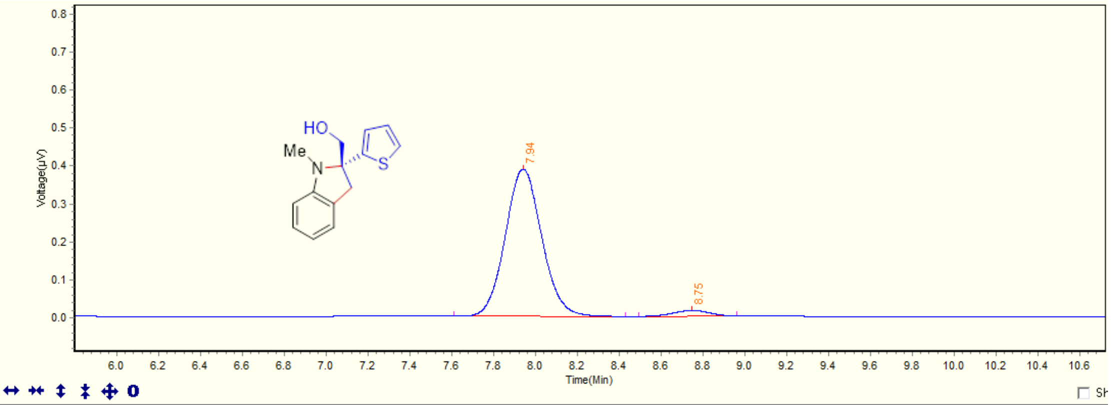

Integration Result | Calculation Result | TimeTable

No.	Retention Time	Peak Area	Peak Height	Peak Area(%)	Peak Width	Peak Type
1	7.94	2352908	194180	96.26%	0.816	BB
2	8.75	91372	7526	3.74%	0.47	BB
Total		2,444,280	201,706	100.00%		

HPLC spectra of **13**

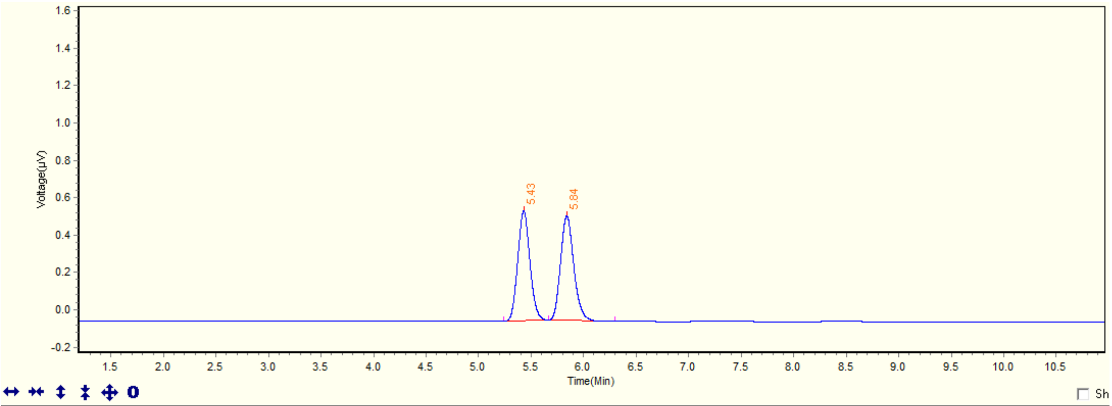

No.	Retention Time	Peak Area	Peak Height	Peak Area(%)	Peak Width	Peak Type
1	5.43	2373707	293985	49.20%	0.425	BB
2	5.84	2451013	279667	50.80%	0.637	BB
Total		4,824,720	573,652	100.00%		

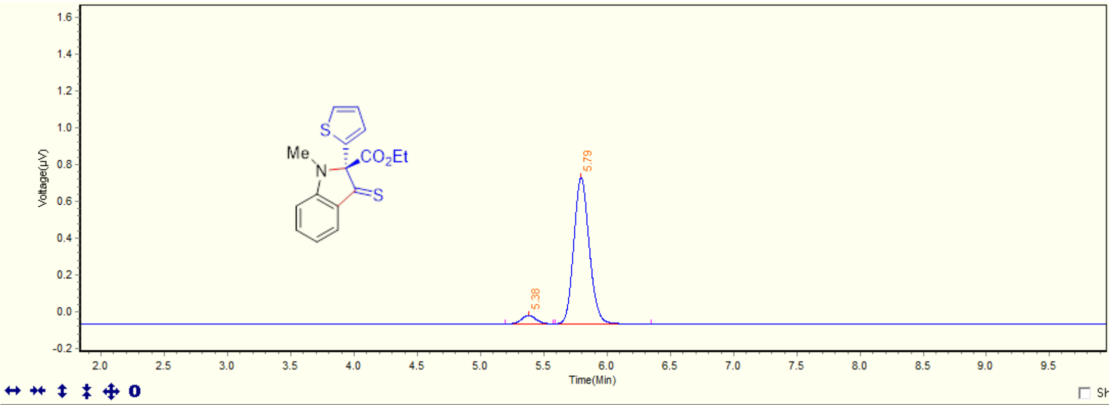

No.	Retention Time	Peak Area	Peak Height	Peak Area(%)	Peak Width	Peak Type
1	5.38	177460	22692	5.00%	0.381	BB
2	5.79	3370138	397175	95.00%	0.759	BB
Total		3,547,598	419,867	100.00%		

References

1. Li, X. *et al.* Arylazolyl(azinyl)thioacetanilides: Part 19: Discovery of Novel Substituted Imidazo[4,5-b]pyridin-2-ylthioacetanilides as Potent HIV NNRTIs Via a Structure-based Bioisosterism Approach. *Chem. Biol. Drug Des.* **88**, 241-253 (2016).
2. Nan, J. *et al.* Rhodium-Catalyzed C–H Annulation of Free Anilines with Vinylene Carbonate as a Bifunctional Synthon. *Org. Lett.* **23**, 8910-8915 (2021).
3. Wu, H., Wang, Q. & Zhu, J. Catalytic Enantioselective Benzilic Ester Rearrangement. *Angew. Chem. Int. Ed.* **59**, 7261-7265 (2020).
4. He, Y.-P. *et al.* Asymmetric Construction of α,α-Disubstituted Piperazinones Enabled by Benzilic Amide Rearrangement. *Angew. Chem. Int. Ed.* **62**, e202217954 (2023).
5. Wang, L., Zhong, J. & Lin, X. Atroposelective Phosphoric Acid Catalyzed Three-Component Cascade Reaction: Enantioselective Synthesis of Axially Chiral N-Arylindoles. *Angew. Chem. Int. Ed.* **58**, 15824-15828 (2019).
6. Cui, J., Duan, Y.-N., Yu, J. & Zhang, C. Iodosobenzene-mediated direct and efficient oxidation of β-dicarbonyls to vicinal tricarbonyls catalyzed by iron(III) salts. *Org. Chem. Front.* **3**, 1686-1690 (2016).
7. Gaussian 16, Revision A.03, M. J. Frisch, G. W. Trucks, H. B. Schlegel, G. E. Scuseria, M. A. Robb, J. R. Cheeseman, G. Scalmani, V. Barone, G. A. Petersson, H. Nakatsuji, X. Li, M. Caricato, A. V. Marenich, J. Bloino, B. G. Janesko, R. Gomperts, B. Mennucci, H. P. Hratchian, J. V. Ortiz, A. F. Izmaylov, J. L. Sonnenberg, D. Williams-Young, F. Ding, F. Lipparini, F. Egidi, J. Goings, B. Peng, A. Petrone, T. Henderson, D. Ranasinghe, V. G. Zakrzewski, J. Gao, N. Rega, G. Zheng, W. Liang, M. Hada, M. Ehara, K. Toyota, R. Fukuda, J. Hasegawa, M. Ishida, T. Nakajima, Y. Honda, O. Kitao, H. Nakai, T. Vreven, K. Throssell, J. A. Montgomery, Jr., J. E. Peralta, F. Ogliaro, M. J. Bearpark, J. J. Heyd, E. N. Brothers, K. N. Kudin, V. N. Staroverov, T. A. Keith, R. Kobayashi, J. Normand, K. Raghavachari, A. P. Rendell, J. C. Burant, S. S. Iyengar, J. Tomasi, M. Cossi, J. M. Millam, M. Klene, C. Adamo, R. Cammi, J. W. Ochterski, R. L. Martin, K. Morokuma, O. Farkas, J. B. Foresman, and D. J. Fox, Gaussian, Inc., Wallingford CT, 2016.
